# Risk Factors for Childhood Stunting in 137 Developing Countries: A Comparative Risk Assessment Analysis at Global, Regional, and Country Levels

**DOI:** 10.1371/journal.pmed.1002164

**Published:** 2016-11-01

**Authors:** Goodarz Danaei, Kathryn G. Andrews, Christopher R. Sudfeld, Günther Fink, Dana Charles McCoy, Evan Peet, Ayesha Sania, Mary C. Smith Fawzi, Majid Ezzati, Wafaie W. Fawzi

**Affiliations:** 1 Department of Global Health and Population, Harvard T.H. Chan School of Public Health, Boston, Massachusetts, United States of America; 2 Department of Epidemiology, Harvard T.H. Chan School of Public Health, Boston, Massachusetts, United States of America; 3 Harvard Graduate School of Education, Cambridge, Massachusetts, United States of America; 4 RAND Corporation, Pittsburgh, Pennsylvania, United States of America; 5 Department of Global Health and Social Medicine, Harvard Medical School, Boston, Massachusetts, United States of America; 6 MRC-PHE Centre for Environment and Health, School of Public Health, Imperial College London, London, United Kingdom; 7 Wellcome Trust Centre for Global Health Research, Imperial College London, London, United Kingdom; 8 Department of Nutrition, Harvard T.H. Chan School of Public Health, Boston, Massachusetts, United States of America; Makerere University Medical School, UGANDA

## Abstract

**Background:**

Stunting affects one-third of children under 5 y old in developing countries, and 14% of childhood deaths are attributable to it. A large number of risk factors for stunting have been identified in epidemiological studies. However, the relative contribution of these risk factors to stunting has not been examined across countries. We estimated the number of stunting cases among children aged 24–35 mo (i.e., at the end of the 1,000 days’ period of vulnerability) that are attributable to 18 risk factors in 137 developing countries.

**Methods and Findings:**

We classified risk factors into five clusters: maternal nutrition and infection, teenage motherhood and short birth intervals, fetal growth restriction (FGR) and preterm birth, child nutrition and infection, and environmental factors. We combined published estimates and individual-level data from population-based surveys to derive risk factor prevalence in each country in 2010 and identified the most recent meta-analysis or conducted de novo reviews to derive effect sizes. We estimated the prevalence of stunting and the number of stunting cases that were attributable to each risk factor and cluster of risk factors by country and region.

The leading risk worldwide was FGR, defined as being term and small for gestational age, and 10.8 million cases (95% CI 9.1 million–12.6 million) of stunting (out of 44.1 million) were attributable to it, followed by unimproved sanitation, with 7.2 million (95% CI 6.3 million–8.2 million), and diarrhea with 5.8 million (95% CI 2.4 million–9.2 million). FGR and preterm birth was the leading risk factor cluster in all regions. Environmental risks had the second largest estimated impact on stunting globally and in the South Asia, sub-Saharan Africa, and East Asia and Pacific regions, whereas child nutrition and infection was the second leading cluster of risk factors in other regions.

Although extensive, our analysis is limited to risk factors for which effect sizes and country-level exposure data were available. The global nature of the study required approximations (e.g., using exposures estimated among women of reproductive age as a proxy for maternal exposures, or estimating the impact of risk factors on stunting through a mediator rather than directly on stunting). Finally, as is standard in global risk factor analyses, we used the effect size of risk factors on stunting from meta-analyses of epidemiological studies and assumed that proportional effects were fairly similar across countries.

**Conclusions:**

FGR and unimproved sanitation are the leading risk factors for stunting in developing countries. Reducing the burden of stunting requires a paradigm shift from interventions focusing solely on children and infants to those that reach mothers and families and improve their living environment and nutrition.

## Abstract 2

Using risk factor and height data from population-based surveys, Goodarz Danaei estimate the greatest contributors to childhood stunting worldwide.

## Author Summary

**Why Was This Study Done?:**

- Even though child mortality is decreasing, children around the world are still suffering from delayed physical growth. In fact, 30% of children in developing countries are stunted (i.e., have heights more than two standard deviations below the global standard median height for their age).
- The first 1,000 days of life (up until a child turns two) are most important because development during this period impacts a child for the rest of his or her life. Stunting during this period is related to poor outcomes in health, cognitive development, and educational and economic attainment later in life.
- In order to reduce stunting, it is important to understand its determinants and their relative effect, to help priority-setting in designing policies to improve childhood growth.

**What Did the Researchers Do and Find?:**

- We identified 18 key risk factors for stunting and grouped them into five clusters (maternal nutrition and infection, teenage motherhood and short birth intervals, fetal growth restriction and preterm birth, child nutrition and infection, and environmental factors).
- We used data on the prevalence of each risk factor in each country and its effect on stunting. We then estimated the prevalence and number of cases of stunting among children aged 24 to 35 months in 2010 that were attributable to each of these risk factors, and to each cluster of risk factors combined, in 137 developing countries.
- We found that the leading risk for stunting worldwide was being “term, and small for gestational age” (that is, being born at or after 37 weeks of pregnancy, but being too small), to which 10.8 million cases of stunting among two-year-olds were attributable (out of 44.1 million). This was followed by poor sanitation (7.2 million cases) and diarrhea (5.8 million cases).
- When we grouped the risks together, fetal growth restriction and preterm birth was the leading risk factor cluster in all regions, but there were differences in the ranking of other risk factor clusters across regions. For example, environmental risk factors (i.e., poor water quality, poor sanitary conditions, and use of solid fuels) had the second largest impact on stunting globally and in South Asia, sub-Saharan Africa, and East Asia and Pacific, whereas risk factors related to child nutrition and infection were the second leading risk factors in other regions.

**What Do These Findings Mean?:**

- Efforts to further reduce stunting should be focused on fetal growth restriction and poor sanitation, and this will require refocusing prevention programs on interventions that reach mothers and families and improve their living environment and nutrition.

## Introduction

Child survival has improved substantially over the past fifty years. The annual number of child deaths under age 5 y declined from 17.6 million in 1970 to 6.3 million in 2013, and under-five mortality declined from 143 per 1,000 live births to 44 during the same period [1]. Global progress in improving childhood growth has been less impressive [2]. While the prevalence of stunting (height-for-age *z*-score less than two standard deviations below the global median, as defined by the 2006 World Health Organization Child Growth Standards [3]) among children under 5 y declined from 47% in 1985 to 30% in 2011 globally, only minor improvements have been achieved in some of the poorest regions of the world, especially South Asia and sub-Saharan Africa [2]. In recognition of the large disparities across the globe in the areas of early life nutrition and development, the World Health Assembly set a target to reduce by 40% the number of stunted children worldwide by 2025 [4].

To reach this target, information on ways to alleviate stunting in each country is essential. Randomized trials and observational studies have identified a large number of risk factors for poor childhood growth [5–7]. However, the impact of these risk factors on stunting at the population level (globally, regionally, and at the country level) is not known.

To address this gap, we conducted a global comparative risk assessment analysis of stunting risk factors. We used country-level data on the prevalence of risk factors from global pooling projects of population health surveys, in combination with effect sizes for each risk factor on stunting from recent meta-analyses of epidemiological studies. This report focuses on 18 risk factors for stunting, while a forthcoming paper uses similar methodology to examine four psychosocial risk factors.

## Methods

We estimated the burden of stunting among children 2 y (24–35 mo) of age (i.e., right at the end of the first 1,000 days of life) that is attributable to 18 risk factors in 137 developing countries. The selected countries included all countries designated as developing by the Global Burden of Disease Study [8], which closely correspond to the countries designated as developing by the United Nations for tracking progress towards the Millennium Development Goals [9]. These risk factors were selected from an extensive list of modifiable (i.e., behavioral or environmental; nongenetic) risk factors for stunting based on (i) the availability of high-quality exposure data (i.e., nationally representative data using standard measurements such as measured weight rather than self-report, and using appropriate statistical methods for pooling and imputing data [10]), (ii) strong evidence for an association with stunting, and (iii) the availability of evidence on the effect size on stunting from recent meta-analyses of epidemiological studies (criteria described in detail in S1 Text; see also [5–7] and S2 Table). Estimating the burden of stunting attributable to various risks does not in itself establish causality, but because we have included only risk factors for which there is convincing evidence of a causal relationship with stunting, the relationships examined here can be interpreted as our current best estimates of their causal effect. Stunting was defined as height-for-age *z*-score < −2 based on the World Health Organization Child Growth Standards [3]. We grouped risk factors into five clusters: maternal nutrition and infection, teenage motherhood and short birth intervals, fetal growth restriction (FGR) and preterm birth, child nutrition and infection, and environmental factors (i.e., unimproved water and sanitation and use of biomass fuels) (Table 1). These categories were based on the similarity of risk factors and of their corresponding interventions. We estimated the proportion of stunting that is attributable to each risk factor and cluster of risk factors in each country, as detailed below.

**Table 1:** Sources of data on the selected risk factors and their effect size for stunting.

Risk Factor	Definition	Evidence on Effect Size for Stunting	Effect Size^a^ (95% CI)	Source of Exposure Data
**Maternal nutrition and infection**				
Maternal short stature	Maternal height <160 cm	Pooled analysis of DHS [11]	Maternal height: <145 cm: 2.13 (2.10, 2.16); 145 to <150 cm: 1.78 (1.76, 1.80); 150 to <155 cm: 1.48 (1.46, 1.49); 155 to <160 cm: 1.24 (1.23, 1.26)	Height among women 18–49 y of age [12]^b^ ^,^ ^c^
Maternal underweight	Maternal BMI <18.5 kg/m^2^	Pooled analysis of population-based cohort studies and WHO perinatal facility-based data from 24 countries [13]	OR for LBW: 1.64 (1.38, 1.94)	Estimates of underweight among women of reproductive age [14]^b^ ^,^ ^d^
Maternal malaria	Malaria in pregnancy	Systematic review of IPTp RCTs [15]^e^	RR for LBW: 1.37 (1.13, 1.63)	Malaria Atlas Project estimates of *Plasmodium falciparum* parasite prevalence [16]^b^ ^,^ ^d^
Maternal anemia	Maternal hemoglobin <110 g/l	Systematic review of cohort studies [17]	OR for LBW: 1.29 (1.09, 1.53)	Estimates of hemoglobin concentration among pregnant women [18]^b^ ^,^ ^d^
**Teenage motherhood and short birth intervals**				
Teenage motherhood	Maternal age at delivery <20 y	Pooled analysis of DHS [19]	Maternal age: <18 y: 1.20 (1.19, 1.22); 18–19 y: 1.11 (1.10, 1.12)	DHS estimates of teenage motherhood [19]
Short birth intervals	<24 mo between consecutive births	Pooled analysis of DHS [19]	Birth spacing <12 mo: 1.14 (1.11, 1.67); 12–23 mo: 1.11 (1.10, 1.12)	DHS estimates of birth spacing [19]
**Fetal growth restriction and preterm birth**				
Preterm, small for gestational age	Birth before 37 wk of gestation and weight <10th percentile for gestational age	Meta-analysis of observational cohort studies [20]	4.51 (3.42, 5.93)	Estimates of prevalence of preterm, small for gestational age [21]^b^
Preterm, appropriate for gestational age	Birth before 37 wk of gestation and weight ≥10th percentile for gestational age	Meta-analysis of observational cohort studies [20]	1.93 (1.71, 2.18)	Estimates of prevalence of preterm, appropriate for gestational age [21] ^b^
Term, small for gestational age	Birth at or after 37 wk of gestation and weight <10th percentile for gestational age	Meta-analysis of observational cohort studies [20]	2.43 (2.22, 2.66)	Estimates of prevalence of term, small for gestational age [21]^b^
Low birth weight^f^	Birth weight <2,500 g	Meta-analysis of observational cohort studies [20]	2.92 (2.56, 3.33)	Estimates of LBW [21]^b^
**Child nutrition and infection**				
Childhood zinc deficiency	Deficient zinc intake during childhood based on age- and sex-specific zinc requirements	Systematic review of preventive zinc supplementation trials [22]	Mean decrease in HAZ: 0.06 (0.02, 0.10)^g^ ^,^ ^h^	Estimates of zinc deficiency [23]^b^
Childhood diarrhea	Mean number of diarrhea episodes per year during childhood	Pooled analysis of prospective cohort studies [24]	OR for stunting per one additional diarrhea episode: 1.025 (1.01, 1.04)	Estimates of mean number of diarrhea episodes per child per year [25]^b^
Nonexclusive breastfeeding	Nonexclusive breastfeeding of infants under 6 mo of age	Systematic review of observational studies [26]	RR for diarrhea: not breastfed: 2.65 (1.72, 4.07); partially breastfed: 1.69 (1.03, 2.76); predominantly breastfed: 1.26 (0.81, 1.95)	Estimates of prevalence of nonexclusive breastfeeding [27]^b^
Discontinued breastfeeding	Discontinued breastfeeding of children 6–24 mo of age	Systematic review of observational studies [26]	RR for diarrhea: 1.32 (1.06, 1.63)	Estimates of prevalence of discontinued breastfeeding [27]^b^
HIV infection without HAART before 2 y of age	Child HIV infection without initiation of HAART until after 2 y of age	Systematic review of observational studies [28–31] (described in S4 Text)	Mean decrease in HAZ: 0.63 (0.46, 0.80)^h^	UNAIDS estimates of prevalence of HIV infection and HAART coverage [32]^i^
**Environmental factors**				
Unimproved sanitation	Lack of access to safe sanitation in the community (based on WHO/UNICEF JMP definition of improved sanitation)^j^	Pooled analysis of DHS [33]	1.37 (1.33, 1.41)	Estimates of access to sanitation [34]^b^
Unimproved water	Lack of access to clean water in the community (based on WHO/UNICEF JMP definition of improved water source)^j^	Pooled analysis of DHS [33]	1.09 (1.06, 1.12)	Estimates of access to safe drinking water [34]^b^
Use of biomass fuels	Use of biomass fuels for cooking and heating	Systematic review of RCTs and observational cohorts [35]	OR for LBW: 1.40 (1.26, 1.54)	Estimates of proportion of households relying mainly on biomass fuel for cooking [36]^b^

^a^All effect sizes are reported as ORs for stunting unless otherwise stated.

^b^For these risk factors, exposure data were missing for six or fewer of the 137 developing countries (primarily small island nations), and these were imputed using sub-regional or regional averages.

^c^In order to generate estimates of maternal height in categories corresponding to the RR categories, we used estimates of mean height (and its uncertainty) and SD of height (and its uncertainty) for each country. Using data for women aged 18 to 49 y in 2010, incorporating the assumption that height declines linearly per year after age 18 y by 0.03562155 cm (as provided by the authors [12]), we calculated (population-weighted) estimates of the mean and SD of height of women of reproductive age in each country in 2010. Assuming that height follows a normal distribution, we calculated the fraction of women falling into each height category listed above using the mean and SD of height in each country. Using the uncertainty around the mean and SD of height, we propagated uncertainty at every step using 1,000 simulations. The SD used for this calculation is available in S1 Table, and the means are available from the NCD Risk Factor Collaboration website [37].

^d^Input exposure data for maternal underweight, anemia, and malaria are available in S1 Table.

^e^Given the lack of an available RR of malaria for childhood stunting, the inverse of the effect of IPTp on childhood stunting was used as a conservative approximation.

^f^For this analysis, LBW is used only as a mediator because the main effects are nearly entirely encompassed by the combination of the effects of term, small for gestational age; preterm, small for gestational age; and preterm, appropriate for gestational age.

^g^For zinc deficiency, the available effect size was a decrease in linear growth of 0.19 cm (95% CI 0.08–0.30 [22] among zinc-deficient children compared to those without zinc deficiency. We converted this effect size into an HAZ shift by dividing it by the SD of height among children aged 21 mo (the mean age of children in the zinc deficiency meta-analysis) from the WHO Child Growth Standards [3]. The estimated mean HAZ shift of 0.06 was then converted into a RR as described in footnote h below.

^h^For zinc deficiency and HIV infection without HAART initiation before 2 y of age (untreated HIV infection), the effect sizes were available as mean differences in HAZ between exposed and unexposed groups, but not as RRs. To convert HAZ shifts into RRs, we used the observed population mean HAZ and estimated a counterfactual HAZ had there been no zinc deficiency/late HAART initiation by subtracting off the HAZ shift attributable to each of these risks from each country’s observed mean HAZ. We converted observed country-level estimates of mean HAZ among children under 5 y to mean HAZ among children age 2 y as described in S2 Text [2,38]. For zinc deficiency and untreated HIV separately, we then translated the two mean HAZ levels for each country into stunting prevalence by using the linear regression crosswalk described in S3 Text [38] and shown in S1 Fig. We used the ratio of the counterfactual to the observed stunting prevalence generated from the crosswalk as a country-specific estimate of the RR.

^i^Using data available in the *UNAIDS Report on the Global AIDS Epidemic 2013* [32] on the number of HIV-infected children on HAART and not on HAART, and assuming that 75% of HIV-infected children on HAART initiate treatment before 2 y of age, we calculated the fraction of HIV-infected children age 2 y who are not yet on HAART (the exposure of interest) using this equation: HIV prevalence among children × (1 − HAART coverage among children) + HIV prevalence among children × HAART coverage among children × 25%. The data inputs (as shared by the authors of [32]) are available in S1 Table.

^j^The WHO/UNICEF Joint Monitoring Programme for Water Supply and Sanitation provides specific definitions of improved water and sanitation [39]. Improved water sources are piped water into dwelling, piped water into yard/plot, public tap or standpipe, tubewell or borehole, protected dug well, protected spring, and rainwater. Improved sanitation is flush toilet, piped sewer system, septic tank, flush/pour flush to pit latrine, ventilated improved pit latrine, pit latrine with slab, composting toilet, and flush/pour flush to unknown place [39]. This classification is used by Fink et al. [33] to create the RRs used for this analysis. The prevalences of exposure to improved water and sanitation used as inputs into this analysis (as shared by the authors of [33]) are available in S1 Table. We subtracted these prevalence values from 100 to calculate the prevalence of exposure to unimproved water and sanitation.

BMI, body mass index; DHS, Demographic and Health Surveys; HAART, highly active antiretroviral therapy; HAZ, height-for-age *z*-score; IPTp, intermittent preventive treatment of malaria in pregnancy; LBW, low birth weight; OR, odds ratio; RCT, randomized control trial; RR, relative risk; SD, standard deviation; WHO/UNICEF JMP, WHO/UNICEF Joint Monitoring Programme for Water Supply and Sanitation.

### Data Sources

We derived the prevalence of exposure to each risk factor for the year 2010 (or as close to 2010 as possible) from published literature and from available surveys such as Demographic and Health Surveys (DHS) (Table 1). Estimates of stunting prevalence for children under 5 y for each country for the year 2011 were provided by the Nutrition Impact Model Study [2], which provides regional and global levels similar to those estimated by WHO [40]. We chose 2010 as the index year for risk factor exposure and 2011 as the index year for stunting exposure to allow a temporal sequence such that risk factors are measured or estimated before stunting. To estimate the prevalence of stunting and number of stunted children at age 2 y (i.e., 24 to 35 mo of age), we calculated the ratio of stunting prevalence among children age 2 y to that among children under 5 y in 104 surveys available from the WHO Global Database on Child Growth and Malnutrition (available from the Nutrition Landscape Information System) [38]. For 33 countries without surveys, we used population-weighted sub-regional averages to generate a correction factor (see S2 Text for more detail and S3 Table for the country-specific ratios). Data on cohort size (population of children at age 2 y) were provided by the United Nations Population Division World Population Prospects 2015 Revision [41]. Data on prevalence of teenage motherhood and short birth spacing were available for 64 countries with recent DHS surveys (73 countries did not have a recent DHS survey). For countries without DHS data, we used a sub-regional average if estimates from at least one country in the region were available (or a regional average when no data were available within the sub-region; see S3 Fig for sub-region and region classifications). The analysis of child HIV infection without highly active antiretroviral therapy (HAART) before 2 y of age (untreated HIV infection) was conducted only for 45 countries with available HIV prevalence data. To inform the effect size of each risk factor on stunting, we identified the most recent meta-analyses of epidemiological studies or conducted de novo systematic reviews (S1 Text; S2 Table).

### Statistical Analyses

We calculated the population attributable fraction (PAF) of stunting for each risk factor using methods that have been described elsewhere [42]. The estimated PAF for each risk factor quantifies the independent effect of that specific risk factor on stunting (holding all other risk factors constant); the PAF is estimated using the following relationship:

$$\text{PAF}=\frac{\sum_{i}P_{i}\left({\text{RR}}_{i}-1\right)}{\sum_{i}P_{i}\left({\text{RR}}_{i}-1\right)+1}$$

where *RR*_*i*_ is the relative risk of stunting comparing category *i* with the reference (or optimal) category and *P*_*i*_ is the prevalence of exposure to the risk factor of interest.

For most risk factors, epidemiological studies have reported effect sizes on stunting. For maternal malaria, underweight, and anemia, as well as biomass fuel use, meta-analyses of effect sizes were available only on low birth weight (LBW), which is itself associated with stunting [20]. For these risk factors, we calculated the PAF of stunting by multiplying the proportion of LBW attributable to the risk factor (i.e., the PAF) by the proportion of stunting attributable to LBW in each country. For example, if in a particular country 20% of LBW is attributable to maternal underweight and 30% of stunting is attributable to LBW, then it can be easily inferred that 6% of stunting is attributable to maternal underweight. Similarly, the effects of nonexclusive and discontinued breastfeeding were reported only on diarrhea, which itself is associated with stunting [24]. We used these reported effect sizes to estimate the effect on stunting through diarrhea by multiplying the PAF of diarrhea attributable to these risk factors by the PAF of stunting attributable to diarrhea.

We further estimated the combined effect of several risk factors within each of the five clusters. The number of stunting cases attributable to multiple risk factors in one cluster is less than the sum of the number of stunting cases attributable to each risk factor because one case of stunting can be due to multiple risk factors (i.e., multicausality) and because one risk factor may affect stunting partly through another risk factor in the same cluster (i.e., mediation). To prevent “double counting” due to multicausality, we used a relationship to estimate the combined effects of multiple risk factors based on their individual PAFs [42]. The relationship is captured by the following formula, which assumes that there is no correlation and no effect modification for relative risks:

$${\text{PAF}}_{j}=1-\overset{R}{\underset{i=1}{\prod }}(1-{\text{PAF}}_{i})$$

where PAF_*i*_ is an individual PAF for a risk factor in cluster *j*, and all individual risk factors (from 1 to *R*) in cluster *j* are combined using the formula above to estimate PAF_*j*_, which is the fraction of stunting attributable to all the risk factors in cluster *j*.

To use this relationship in the child nutrition and infection cluster of risk factors, discontinued and nonexclusive breastfeeding were excluded because their effects on stunting are mediated through diarrhea, and we did not include childhood untreated HIV infection because data on this risk factor were not available for all countries. For this risk factor cluster, we had to make an additional modification because part of the effect of zinc deficiency on stunting is mediated by diarrhea. Therefore, using the above formula for this cluster would lead to overestimation if the effect of diarrhea and zinc deficiency were both included in the combined PAF (PAF_*j*_). To correct this, we replaced the overall effect of zinc on stunting with the part of the effect of zinc on stunting that is not mediated through diarrhea. We conducted a literature review but did not find any studies that quantified this relationship and therefore assumed that 50% of the excess relative risk of stunting due to zinc deficiency is mediated by diarrhea. For example, in Nigeria, the overall relative risk of zinc deficiency for stunting was estimated to be 1.04 (based on a 0.06 lower mean height-for-age *z*-score in zinc-deficient children; see notes below Table 1, and S3 Text, for more details), which corresponds to an excess relative risk of 0.04. Using the 50% proportion mentioned above, we calculated that the excess relative risk of zinc deficiency not mediated through diarrhea was 0.02, and that calculation in turn yields a “direct” relative risk of 1.02. This relative risk does not include the effect of zinc deficiency on diarrhea and can be combined with the effect of diarrhea on stunting to estimate the combined effect of both risk factors on stunting without creating a bias due to overestimation. We conducted a sensitivity analysis using 0% and 100% as the mediated proportion (S4 Table).

We calculated the attributable prevalence of stunting by multiplying the PAF by the prevalence of stunting in each country. We also calculated the attributable number of stunting cases in children aged 2 y by multiplying the PAF by the number of stunted children at age 2 y. To quantify uncertainty, we used the mean and standard error for each exposure prevalence and effect size, separately, to generate 1,000 simulations for the prevalence of the risk factor and its odds ratio for stunting, then calculated the PAF and attributable prevalence of stunting at age 2 y 1,000 times (once per simulation). The 95% confidence intervals of PAFs and numbers of attributable stunting cases were calculated by using the 2.5th and the 97.5th percentiles of draws. All analyses were conducted using STATA SE version 13.1. This study was conducted solely using secondary and existing datasets and therefore did not require institutional review board review.

## Results

**Fig 1:**
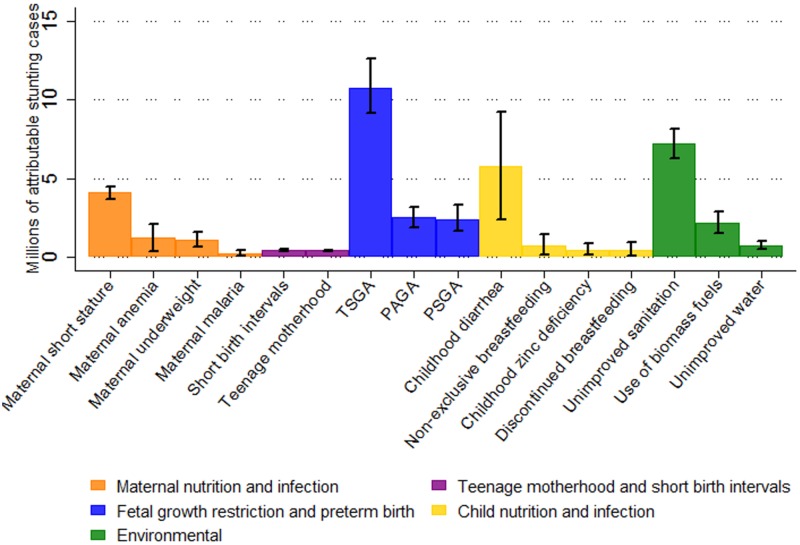
Risk factors ranked within each cluster by number of attributable stunting cases in children aged 2 y in 137 developing countries in 2011. Whiskers indicate 95% confidence intervals. Effects are not additive because each case of stunting can be attributed to more than one risk factor. Untreated HIV infection is not included because exposure data for all countries were not available. PAGA, preterm, appropriate for gestational age; PSGA, preterm, small for gestational age; TSGA, term, small for gestational age.

**Fig 2:**
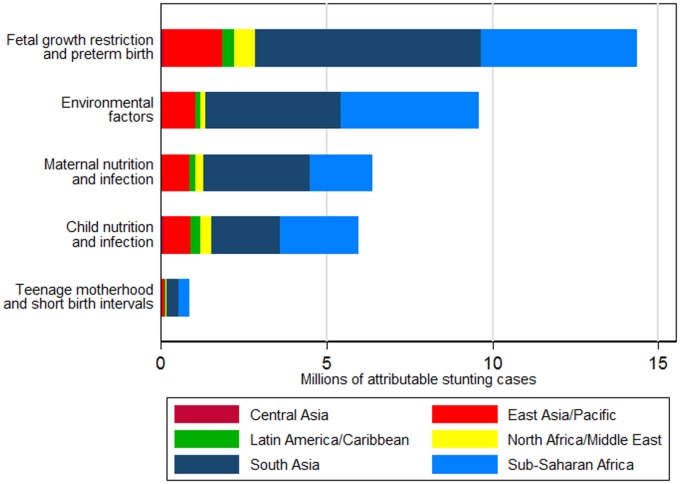
Number of stunting cases in children aged 2 y in 2011 attributable to risk factor clusters, stratified by region. Effects are not additive because each case of stunting can be attributed to more than one risk factor. Untreated HIV infection is not included because exposure data for all countries were not available.

In 2011, we estimated that 44.1 million children aged 2 y in the selected 137 developing countries were stunted, corresponding to 36% of the 2-y-old population. The most important individual risk factor for stunting was being term, small for gestational age (TSGA), with 10.8 million (95% CI 9.1 million–12.6 million) stunting cases attributable at age 2 y in 2011. Unimproved sanitation, with 7.2 million (95% CI 6.3 million–8.2 million) attributable cases of stunting, and diarrhea, with 5.8 million (95% CI 2.4 million–9.2 million) attributable cases, were the second and third most important risk factors for stunting worldwide, respectively (Fig 1). When clusters of risk factors were considered, FGR and preterm birth (preterm, small for gestational age; TSGA; preterm, appropriate for gestational age [PAGA]) were the leading risk factors for stunting prevalence, with 32.5% of stunting prevalence being attributed to these factors (14.4 million cases, 95% CI 12.6 million–16.2 million). This cluster of risk factors was followed by environmental factors (unimproved water, unimproved sanitation, and biomass fuel use), with 21.7% (9.6 million cases, 95% CI 8.4 million–10.8 million), maternal nutrition and infection risk factors, with 14.4% (6.4 million cases, 95% CI 5.3 million–7.5 million), and child nutrition and infection risk factors, with 13.5% (6.0 million cases, 95% CI 2.6 million–9.4 million) of attributable stunting cases. Teenage motherhood and short birth intervals had the fewest attributable stunting cases, with 1.9% (0.86 million cases, 95% CI 0.77 million–0.95 million) (Table 2; Fig 2).

**Table 2:** Population attributable fraction and number of stunting cases in children aged 2 y in 2011 attributable to risk factor clusters by region.

Region	Maternal Nutrition and Infection		Teenage Motherhood and Short Birth Intervals		Fetal Growth Restriction and Preterm Birth		Child Nutrition and Infection^a^		Environmental Factors	
	PAF (Percent)	Number Stunted (Thousands)	PAF (Percent)	Number Stunted (Thousands)	PAF (Percent)	Number Stunted (Thousands)	PAF (Percent)	Number Stunted (Thousands)	PAF (Percent)	Number Stunted (Thousands)
All developing countries	14.4 (12.5, 16.5)	6,374 (5,331, 7,514)	1.9 (1.9, 2.0)	858 (774, 945)	32.5 (30.0, 35.2)	14,366 (12,553, 16,209)	13.5 (6.0, 21.3)	5,962 (2,586, 9,444)	21.7 (19.9, 23.5)	9,584 (8,364, 10,783)
East Asia and Pacific	11.5 (10.4, 12.7)	842 (728, 955)	1.3 (1.2, 1.4)	96 (81, 110)	24.0 (21.0, 26.9)	1,758 (1,477, 2,041)	10.9 (4.7, 17.3)	802 (347, 1,328)	13.8 (11.3, 16.3)	1,014 (787, 1,282)
South Asia	19.2 (16.1, 22.3)	3,200 (2,370, 4,063)	2.2 (2.1, 2.3)	361 (287, 437)	40.9 (37.5, 44.2)	6,809 (5,363, 8,329)	12.3 (5.2, 20.1)	2,053 (880, 3,536)	24.5 (22.2, 26.9)	4,082 (3,163, 4,992)
Central Asia	6.5 (5.9, 7.2)	30 (24, 36)	1.6 (1.5, 1.7)	7 (6, 8)	22.6 (19.3, 25.5)	102 (83, 123)	18.9 (8.3, 29.1)	85 (36, 135)	4.2 (3.3, 5.1)	19 (14, 24)
North Africa and Middle East	9.2 (8.3, 10.3)	238 (201, 280)	1.8 (1.7, 2.0)	48 (41, 54)	24.6 (22.2, 27.3)	635 (533, 743)	12.9 (5.7, 20.6)	333 (148, 545)	6.2 (5.2, 7.3)	161 (129, 195)
Sub-Saharan Africa	12.2 (10.4, 14.1)	1,875 (1,572, 2,194)	2.0 (1.9, 2.1)	306 (285, 327)	30.6 (28.1, 33.3)	4,703 (4,257, 5,141)	15.4 (6.9, 24.1)	2,371 (1,066, 3,715)	27.0 (25.1, 29.1)	4,153 (3,777, 4,544)
Latin America and Caribbean	10.8 (10.0, 11.6)	189 (167, 213)	2.3 (2.2, 2.4)	41 (36, 46)	20.5 (18.4, 23.0)	359 (308, 417)	18.1 (7.9, 27.8)	318 (136, 495)	8.9 (7.9, 9.9)	156 (135, 178)

95% confidence intervals presented in parentheses.

^a^Untreated HIV infection is not included in the global analysis because estimates of its attributable burden were not available for all countries.

PAF, population attributable fraction.

The risk factor cluster FGR and preterm birth was associated with the largest attributable burden of stunting in all regions. The cluster of environmental factors, most importantly unimproved sanitation, was the second leading cluster of risk factors in South Asia, sub-Saharan Africa, and East Asia and Pacific, whereas in Central Asia, Latin America and Caribbean, and North Africa and Middle East, the second leading cluster was child nutrition and infection (mostly childhood diarrhea). Central Asia had the highest proportion of stunting attributable to child nutrition and infection risk factors across all regions, at 18.9%, and sub-Saharan Africa had the largest proportion of stunting attributable to environmental risk factors across all regions, at 27.0%. Of all stunting cases attributable to each risk factor cluster, South Asia and sub-Saharan Africa had the largest shares across all regions (ranging from 34% to 50%, and 29% to 43%, respectively, across risk factor clusters) due to having both high exposures to the selected risk factors and large numbers of stunted children.

Within these larger regions, there were important differences across sub-regions in the burden of stunting attributable to risk factors and risk factor clusters. Within the sub-Saharan Africa region, the attributable prevalence of stunting associated with unimproved sanitation in southern Africa was less than half that of central, east, and west Africa. Similarly, diarrhea was associated with almost three times the burden of stunting in Andean and central Latin America as in tropical and southern Latin America. The burden of diarrhea also differed substantially within Asia, with much smaller attributable prevalence of stunting in the East Asia sub-region (1.9 percentage points) than in the Central Asia, South Asia, and Southeast Asia sub-regions (all greater than 3.5 percentage points).

**Fig 3:**
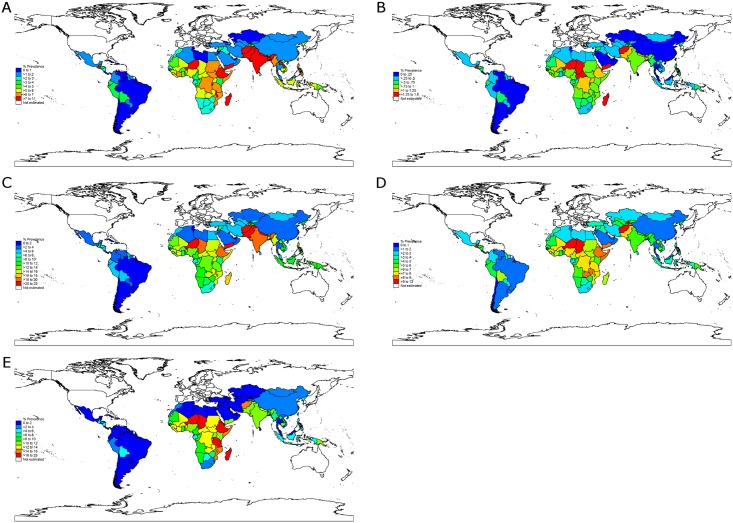
Stunting prevalence attributable to the selected risk factor clusters by country. (A) Maternal nutrition and infection. (B) Teenage motherhood and short birth intervals. (C) Fetal growth restriction and preterm birth. (D) Child nutrition and infection. (E) Environmental factors.

At the country level, nations with high stunting prevalence such as Niger, Burundi, Yemen, Eritrea, Ethiopia, Afghanistan, Timor-Leste, and Zambia (all with prevalence greater than 50%) had, as expected, a large burden of stunting attributable to all risk factor clusters (Fig 3; S4 Fig displays the PAFs). However, several countries with a relatively lower prevalence of stunting also had a relatively large attributable burden due to high exposure to specific risk factors. For example, Somalia had the largest prevalence of stunting attributable to discontinued breastfeeding and the second largest prevalence attributable to nonexclusive breastfeeding (0.9 and 1.4 percentage points, respectively). Malawi had the top rank for PAGA, at 4.9 percentage points, and Bangladesh had the top rank for teenage motherhood, at 0.9 percentage points (country-level PAFs and numbers of attributable stunting cases are available in S5 Text and on the study website: http://www.healthychilddev.sph.harvard.edu/).

The ranking of risk factors within each country was generally consistent with global and regional ranking of risk factors (S5 Table). Nevertheless, specific risk factors imposed a larger burden of stunting in particular countries irrespective of the burden of stunting in the country: unimproved sanitation surpassed TSGA as the leading risk factor in China and in many sub-Saharan African countries, and nonexclusive and discontinued breastfeeding had a substantially higher rank in middle-income countries (e.g., Mexico, South Africa, Iran, Turkey, and Argentina) than in low-income countries.

## Discussion

Our results suggest that a large proportion of childhood stunting in developing countries could be prevented if exposure to a few key risk factors could be eliminated. Globally, TSGA was associated with the largest stunting burden, followed by unimproved sanitation and childhood diarrhea. This pattern highlights the success of current clinical and public health interventions to prevent and manage childhood infections and improve childhood nutrition in many developing countries [43,44], but also calls for a new focus on interventions before and during pregnancy to address intergenerational effects of malnutrition among girls and women [45,46], as well as interventions to improve the environment in which mothers and families live, with specific attention to improving sanitation.

A previous analysis of FGR and preterm births in developing countries reported smaller fractions of stunting attributable to TSGA (e.g., a PAF of 16% for TSGA compared with 24% in our analysis) [20]. However, the value reported in this previous analysis may be an underestimation, as the regional and global effects were estimated using regional prevalence of stunting as opposed to estimating the attributable stunting in each country, as we did here.

The large burden of stunting attributable to FGR is perhaps unsurprising given that prenatal restricted growth is logically strongly related to postnatal restricted growth; nevertheless, our findings serve to further emphasize the importance of early intervention during pregnancy. Several recent reviews have identified maternal iron, balanced protein-energy, and multiple micronutrient supplementation as the most effective interventions to alleviate FGR [17,47]. However, providing these interventions before pregnancy or in its early months is logistically difficult because in many developing countries, the majority of pregnant women start attending antenatal clinics in their second or third trimester.

Environmental factors (i.e., unimproved water, unimproved sanitation, and biomass fuel use) had the second largest global attributable burden. Particularly, 7.2 million cases of stunting worldwide were attributable to unimproved sanitation. The attributable burden of unimproved sanitation for stunting was larger (though not significantly) than that of childhood diarrhea, as some of the effects of improved sanitation may be through prevention of other childhood infections and improvement of maternal health and nutrition in pregnancy [48,49]. This further underscores the importance of ongoing water, sanitation, and hygiene (WASH) programs [50] to increase access to, and use of, safe water and sanitation for children and families worldwide.

We found that one in seven cases of stunting was attributable to child nutrition and infection risk factors. Programs to promote adequate complementary feeding [51] and rotavirus and cholera vaccines [52] may reduce the attributable burden of this group of risks. Childhood HIV infection remains a significant contributor to child mortality [53] but was not a major risk for stunting at the population level. In Swaziland and Lesotho, the countries with the highest prevalence of this risk factor, the prevalence of stunting attributable to untreated HIV infection was estimated to be 0.7 and 0.8 percentage points, respectively. We may have slightly underestimated the fraction of stunting attributable to HIV as we were only able to estimate the effect of untreated child HIV infection, due to lack of data on the growth of HIV-infected children who receive HAART before 2 y of age compared with HIV-uninfected peers in developing countries (S2 Fig). Nevertheless, the contribution of HIV-infected children who receive HAART before 2 y to population-level stunting is likely small, given that young children initiating HAART exhibit rapid linear growth catch-up [54]. Teenage motherhood and short birth intervals had a fairly small effect on stunting at the population level due to both lower prevalence of exposure in many developing countries and smaller effect sizes for stunting compared with other risk factors.

The global scope of this analysis and the large number of risk factors included result in several limitations. Although we included only risk factors with strong evidence of an association with stunting, causality can never be guaranteed by observational studies (which were the sources of nearly all effect sizes available for the risk factors analyzed here). Although the list of risk factors is extensive, we had to exclude several risk factors because country-level data on exposure or effect size on stunting were not available. Examples are maternal smoking, prenatal alcohol use, and illicit drug use. Similarly, global data on environmental pollutant exposures in childhood, such as lead and arsenic exposure, were not available. In addition, we had to use approximate estimates for prevalence of use of biomass fuels, maternal malaria, maternal short stature, and maternal underweight. We used the prevalence of relying mainly on biomass fuels for cooking as a proxy for all exposure to biomass fuels, underweight among women of reproductive age as a proxy for maternal underweight, height among women aged 18 to 49 y as a proxy for maternal height, and prevalence of *P*. *falciparum* infection among children aged 2 to 10 y as a proxy for maternal malaria prevalence. Estimates of uncertainty in the exposures to maternal malaria, maternal short stature, childhood zinc deficiency, untreated HIV infection, LBW, and PAGA were unavailable, so uncertainty in the estimates of the effect of these risk factors is underestimated. Relatedly, we did not incorporate the uncertainty from converting the prevalence of stunting among children under 5 y to the prevalence of stunting among children age 2 y. Another limitation is that we estimated the effect of six risk factors through either LBW (maternal anemia, malaria, and underweight and biomass fuel use) or diarrhea (nonexclusive and discontinued breastfeeding), which may have led to underestimation of the effect of the maternal and childhood nutrition and infection risk factor clusters. As is standard in global risk factor analyses [42], we assumed that *proportional* effects are fairly similar across countries. Evidence from analyses of FGR and preterm birth supports this assumption as the odds ratios for stunting across different regions were fairly similar [20]. Multi-country studies are required to evaluate variation of effect sizes for other risk factors, but such studies are currently not available. Finally, the effect sizes for most risk factors were reported as odds ratios, which overestimate the relative risk when exposure is not rare and could lead to overestimating the effect of these risk factors on stunting. Correcting this bias requires information on the incidence of stunting among unexposed children for each risk factor and in each country [55], and these data are currently not available.

Our analysis also had several major strengths. We included all major risk factors for stunting after considering an extensive set and limiting our analysis to those with convincing evidence on their effect on stunting and high-quality data on country-level exposure. We reviewed and did not include risk factors with insufficient evidence on their effect on stunting, including child HIV infection with HAART [56], maternal and childhood iodine deficiency [57], child hookworm infection [58], acute lower respiratory infection [59], and childhood malaria [60]. We also excluded risk factors for which meta-analyses of observational studies and/or randomized trials identified no significant effect on childhood stunting. These were childhood anemia [61], maternal hookworm infection [62], and vitamin A deficiency [63]. For the included risk factors, we used the most recent and reliable meta-analyses of effect sizes on stunting. We quantified uncertainty at each step of estimation and reported overall uncertainty in the final results.

Our results represent a consistent and comparable set of global estimates of the impact of 18 risk factors on stunting. FGR, unimproved sanitation, and diarrhea are the leading risk factors for stunting globally, with larger estimated impacts on stunting in sub-Saharan Africa and South Asia compared with other regions. According to our findings, reducing the burden of stunting requires continuing the current efforts to diagnose and treat maternal and child infections, especially diarrhea, along with a new focus on clinical and public health interventions that focus on improving nutrition and sanitation among mothers and families.

## Supporting Information

S1 Data Data availability statement. (XLSX)

S1 Fig Scatter plot and regression line to model prevalence of stunting (height-for-age *z*-score less than −2) from height-for-age *z*-scores using survey data from the WHO Global Database on Child Growth and Malnutrition. (TIF)

S2 Fig Forest plot of observational studies comparing growth of HIV-infected children under 2 y of age who did not receive HAART versus HIV-exposed uninfected children. (TIF)

S3 Fig The 137 included developing countries. By sub-region (A) and by region (B).
(TIF)

S4 Fig Population attributable fraction of stunting attributable to the selected risk factor clusters by country. (A) Maternal nutrition and infection. (B) Teenage motherhood and short birth intervals. (C) FGR and preterm birth. (D) Child nutrition and infection. (E) Environmental factors.
(TIF)

S1 PRISMA Checklist (DOC)

S1 Table Prevalence data inputs (in percent, with 95% confidence intervals in parentheses where available) for exposure to untreated HIV, unimproved water and sanitation, maternal anemia, maternal underweight, maternal malaria, and standard deviations (in centimeters, with 95% confidence intervals) of maternal height. (XLSX)

S2 Table Categories of level of evidence on causal effects of risk factors on stunting. (DOCX)

S3 Table Ratios of stunting and mean height-for-age *z*-score among children age 2 y compared to children under age 5 y from 104 and 102 surveys, respectively, from the WHO Global Database on Child Growth and Malnutrition. (DOCX)

S4 Table Population attributable fraction and number of stunting cases by region attributable to the childhood nutrition and infection cluster of risk factors, based on differing assumptions about the proportion of zinc deficiency that is mediated through diarrhea (95% confidence intervals in parentheses). (DOCX)

S5 Table Ranking of risk factors within each country with respect to the attributable number of stunting cases. The leading risk factor is ranked one and colored bright red, and the risk factor with the smallest number of attributable cases is ranked 16 and colored dark green. Risk factors are ordered with respect to their global impact on stunting, and countries are ordered with respect to the number of stunted children at age 2 y in 2010. Untreated HIV infection is not included because exposure data were available for only 45 countries.
(DOCX)

S1 Text Methods used to identify sources of evidence on effect sizes. (DOCX)

S2 Text Description of conversion of stunting prevalence among children under 5 y to stunting prevalence among children age 2 y. (DOCX)

S3 Text Description of height-for-age *z*-score to stunting prevalence crosswalk. (DOCX)

S4 Text Systematic review of HAART and childhood growth. (DOCX)

S5 Text Country profiles showing country-specific results for all risk factors. (DOCX)

S6 Text Grant proposal methods. (DOCX)

## Abbreviations

DHS: Demographic and Health Surveys
FGR: fetal growth restriction
HAART: highly active antiretroviral therapy
LBW: low birth weight
PAF: population attributable fraction
PAGA: preterm, appropriate for gestational age
TSGA: term, small for gestational age

## References

[1] Wang H, Liddell CA, Coates MM, Mooney MD, Levitz CE, Schumacher AE, et al Global, regional, and national levels of neonatal, infant, and under-5 mortality during 1990–2013: a systematic analysis for the Global Burden of Disease Study 2013. Lancet. 2014;384:957–79. 10.1016/S0140-6736(14)60497-9 24797572 PMC4165626

[2] Stevens GA, Finucane MM, Paciorek CJ, Flaxman SR, White RA, Donner AJ, et al Trends in mild, moderate, and severe stunting and underweight, and progress towards MDG 1 in 141 developing countries: a systematic analysis of population representative data. Lancet. 2012;380:824–34. 10.1016/S0140-6736(12)60647-3 22770478 PMC3443900

[3] World Health Organization. Child growth standards: length/height-for-age. Geneva: World Health Organization; 2015 [cited 2015 Apr 8]. Available from: http://www.who.int/childgrowth/standards/height_for_age/en/.

[4] World Health Organization. WHA global nutrition targets 2025: stunting policy brief. Geneva: World Health Organization; 2014 [cited 2015 Jul 31]. Available from: http://www.who.int/nutrition/topics/globaltargets_stunting_policybrief.pdf.

[5] Black RE, Allen LH, Bhutta ZA, Caulfield LE, de Onis M, Ezzati M, et al Maternal and child undernutrition: global and regional exposures and health consequences. Lancet. 2008;371:243–60. 10.1016/S0140-6736(07)61690-0 18207566

[6] Black RE, Victora CG, Walker SP, Bhutta ZA, Christian P, de Onis M, et al Maternal and child undernutrition and overweight in low-income and middle-income countries. Lancet. 2013;382:427–51. 10.1016/S0140-6736(13)60937-X 23746772

[7] Bhutta ZA, Das JK, Rizvi A, Gaffey MF, Walker N, Horton S, et al Evidence-based interventions for improvement of maternal and child nutrition: what can be done and at what cost? Lancet. 2013;382:452–77. 10.1016/S0140-6736(13)60996-4 23746776

[8] Global Burden of Disease Cancer Collaboration, Fitzmaurice C, Dicker D, Pain A, Hamavid H, Moradi-Lakeh M, et al The global burden of cancer 2013. JAMA Oncol. 2015;1:505–27. 10.1001/jamaoncol.2015.0735 26181261 PMC4500822

[9] United Nations. Millennium Development indicators: world and regional groupings. 2014 [cited 2016 Apr 24]. Available from: http://mdgs.un.org/unsd/mdg/Host.aspx?Content=Data/RegionalGroupings.

[10] Finucane MM, Paciorek CJ, Danaei G, Ezzati M. Bayesian estimation of population-level trends in measures of health status. Stat Sci. 2014;29:18–25.

[11] Özaltin E, Hill K, Subramanian SV. Association of maternal stature with offspring mortality, underweight, and stunting in low- to middle-income countries. JAMA. 2010;303:1507–16. 10.1001/jama.2010.450 20407060 PMC3100588

[12] NCD Risk Factor Collaboration. A century of trends in adult human height. Elife. 2016;5:e13410 10.7554/eLife.13410 27458798 PMC4961475

[13] Han Z, Mulla S, Beyene J, Liao G, McDonald SD, Knowledge Synthesis Group. Maternal underweight and the risk of preterm birth and low birth weight: a systematic review and meta-analyses. Int J Epidemiol. 2011;40:65–101. 10.1093/ije/dyq195 21097954

[14] NCD Risk Factor Collaboration. Trends in adult body-mass index in 200 countries from 1975 to 2014: a pooled analysis of 1698 population-based measurement studies with 19·2 million participants. Lancet. 2016;387:1377–96. 10.1016/S0140-6736(16)30054-X 27115820 PMC7615134

[15] Radeva-Petrova D, Kayentao K, ter Kuile FO, Sinclair D, Garner P. Drugs for preventing malaria in pregnant women in endemic areas: any drug regimen versus placebo or no treatment. Cochrane Database Syst Rev. 2014;(10):CD000169 10.1002/14651858.CD000169.pub3 25300703 PMC4498495

[16] Gething PW, Patil AP, Smith DL, Guerra CA, Elyazar IR, Johnston GL, et al A new world malaria map: Plasmodium falciparum endemicity in 2010. Malar J. 2011;10:378 10.1186/1475-2875-10-378 22185615 PMC3274487

[17] Haider BA, Olofin I, Wang M, Spiegelman D, Ezzati M, Fawzi WW. Anaemia, prenatal iron use, and risk of adverse pregnancy outcomes: systematic review and meta-analysis. BMJ. 2013;346:f3443 10.1136/bmj.f3443 23794316 PMC3689887

[18] Stevens GA, Finucane MM, De-Regil LM, Paciorek CJ, Flaxman SR, Branca F, et al Global, regional, and national trends in haemoglobin concentration and prevalence of total and severe anaemia in children and pregnant and non-pregnant women for 1995–2011: a systematic analysis of population-representative data. Lancet Glob Health. 2013;1:e16–25. 10.1016/S2214-109X(13)70001-9 25103581 PMC4547326

[19] Fink G, Sudfeld CR, Danaei G, Ezzati M, Fawzi WW. Scaling-up access to family planning may improve linear growth and child development in low and middle income countries. PLoS ONE. 2014;9:e102391 10.1371/journal.pone.0102391 25020132 PMC4096753

[20] Christian P, Lee SE, Donahue Angel M, Adair LS, Arifeen SE, Ashorn P, et al Risk of childhood undernutrition related to small-for-gestational age and preterm birth in low- and middle-income countries. Int J Epidemiol. 2013;42:1340–55. 10.1093/ije/dyt109 23920141 PMC3816349

[21] Lee AC, Katz J, Blencowe H, Cousens S, Kozuki N, Vogel JP, et al National and regional estimates of term and preterm babies born small for gestational age in 138 low-income and middle-income countries in 2010. Lancet Glob Health. 2013;1:e26–36. 10.1016/S2214-109X(13)70006-8 25103583 PMC4221634

[22] Imdad A, Bhutta ZA. Effect of preventive zinc supplementation on linear growth in children under 5 years of age in developing countries: a meta-analysis of studies for input to the lives saved tool. BMC Public Health. 2011;11:S22 10.1186/1471-2458-11-S3-S22 21501440 PMC3231896

[23] Wessells KR, Brown KH. Estimating the global prevalence of zinc deficiency: results based on zinc availability in national food supplies and the prevalence of stunting. PLoS ONE. 2012;7:e50568 10.1371/journal.pone.0050568 23209782 PMC3510072

[24] Checkley W, Buckley G, Gilman RH, Assis AM, Guerrant RL, Morris SS, et al Multi-country analysis of the effects of diarrhoea on childhood stunting. Int J Epidemiol. 2008;37:816–30. 10.1093/ije/dyn099 18567626 PMC2734063

[25] Walker CLF, Rudan I, Liu L, Nair H, Theodoratou E, Bhutta ZA, et al Global burden of childhood pneumonia and diarrhoea. Lancet. 2013;381:1405–16. 10.1016/S0140-6736(13)60222-6 23582727 PMC7159282

[26] Lamberti LM, Walker CLF, Noiman A, Victora C, Black RE. Breastfeeding and the risk for diarrhea morbidity and mortality. BMC Public Health. 2011;11:S15. 10.1186/1471-2458-11-S3-S15 PMC3231888 21501432

[27] Roberts TJ, Carnahan E, Gakidou E. Can breastfeeding promote child health equity? A comprehensive analysis of breastfeeding patterns across the developing world and what we can learn from them. BMC Med. 2013;11:254 10.1186/1741-7015-11-254 24305597 PMC3896843

[28] Bailey RC, Kamenga MC, Nsuami MJ, Nieburg P, St Louis ME. Growth of children according to maternal and child HIV, immunological and disease characteristics: a prospective cohort study in Kinshasa, Democratic Republic of Congo. Int J Epidemiol. 1999;28:532–40. 10405861 10.1093/ije/28.3.532

[29] McDonald CM, Manji KP, Kupka R, Bellinger DC, Spiegelman D, Kisenge R, et al Stunting and wasting are associated with poorer psychomotor and mental development in HIV-exposed Tanzanian infants. J Nutr. 2013;143:204–14. 10.3945/jn.112.168682 23256148 PMC3542911

[30] Taha T, Nour S, Li Q, Kumwenda N, Kafulafula G, Nkhoma C, et al The effect of human immunodeficiency virus and breastfeeding on the nutritional status of African children. Pediatr Infect Dis J. 2010;29:514–8. 10.1097/INF.0b013e3181cda531 20054287

[31] Webb AL, Manji K, Fawzi WW, Villamor E. Time-independent maternal and infant factors and time-dependent infant morbidities including HIV infection, contribute to infant growth faltering during the first 2 years of life. J Trop Pediatr. 2009;55:83–90. 10.1093/tropej/fmn068 18723575 PMC2734313

[32] Joint United Nations Programme on HIV/AIDS. Global report: UNAIDS report on the global AIDS epidemic 2013. 2013 Nov [cited 2016 Sep 29]. Available from: http://www.unaids.org/sites/default/files/media_asset/UNAIDS_Global_Report_2013_en_1.pdf.

[33] Fink G, Günther I, Hill K. The effect of water and sanitation on child health: evidence from the demographic and health surveys 1986–2007. Int J Epidemiol. 2011;40:1196–204. 10.1093/ije/dyr102 21724576

[34] Wolf J, Bonjour S, Prüss-Ustün A. An exploration of multilevel modeling for estimating access to drinking-water and sanitation. J Water Health. 2013;11:64–77. 10.2166/wh.2012.107 23428550

[35] Bruce NG, Dherani MK, Das JK, Balakrishnan K, Adair-Rohani H, Bhutta ZA, et al Control of household air pollution for child survival: estimates for intervention impacts. BMC Public Health. 2013;13:S8. 10.1186/1471-2458-13-S3-S8 PMC3847681 24564764

[36] Bonjour S, Adair-Rohani H, Wolf J, Bruce NG, Mehta S, Prüss-Ustün A, et al Solid fuel use for household cooking: country and regional estimates for 1980–2010. Environ. Health Perspect. 2013;121:784–90. 10.1289/ehp.1205987 23674502 PMC3701999

[37] NCD Risk Factor Collaboration. Height: evolution of adult height over time. 2016 [cited 2016 Jul 29]. Available from: http://www.ncdrisc.org/d-height.html.

[38] World Health Organization. Nutrition Landscape Information System (NLiS). 2016 [cited 2016 Apr 17]. Available from: http://apps.who.int/nutrition/landscape/search.aspx.

[39] WHO/UNICEF Joint Monitoring Programme (JMP) for Water Supply and Sanitation. Improved and unimproved water and sanitation facilities. 2014 [cited 2014 Dec 19]. Available from: http://www.wssinfo.org/definitions-methods/watsan-categories/.

[40] de Onis M, Blössner M, Borghi E. Prevalence and trends of stunting among pre-school children, 1990–2020. Public Health Nutr. 2012;15:142–8. 10.1017/S1368980011001315 21752311

[41] United Nations Population Division. World population prospects: 2015 revision. 2015 [cited 2016 Apr 17]. Available from: http://www.un.org/en/development/desa/population/events/other/10/index.shtml.

[42] Ezzati M, Lopez AD, Rodgers A, Vander Hoorn S, Murray CJL, Comparative Risk Assessment Collaborating Group. Selected major risk factors and global and regional burden of disease. Lancet. 2002;360:1347–60. 12423980 10.1016/S0140-6736(02)11403-6

[43] Gordon JE, Béhar M, Scrimshaw NS. Acute diarrhoeal disease in less developed countries. Bull World Health Organ. 1964;31:1–7. 14230890 PMC2555148

[44] Arifeen SE, Hoque DME, Akter T, Rahman M, Hoque ME, Begum K, et al Effect of the Integrated Management of Childhood Illness strategy on childhood mortality and nutrition in a rural area in Bangladesh: a cluster randomised trial. Lancet. 2009;374:393–403. 10.1016/S0140-6736(09)60828-X 19647607

[45] Stein AD, Barnhart HX, Wang M, Hoshen MB, Ologoudou K, Ramakrishnan U, et al Comparison of linear growth patterns in the first three years of life across two generations in Guatemala. Pediatrics. 2004;113:e270–5. 14993588 10.1542/peds.113.3.e270

[46] Prentice AM, Ward KA, Goldberg GR, Jarjou LM, Moore SE, Fulford AJ, et al Critical windows for nutritional interventions against stunting. Am J Clin Nutr. 2013;97:911–8. 10.3945/ajcn.112.052332 23553163 PMC3628381

[47] Morris RK, Oliver EA, Malin G, Khan KS, Meads C. Effectiveness of interventions for the prevention of small-for-gestational age fetuses and perinatal mortality: a review of systematic reviews. Acta Obstet Gynecol Scand. 2013;92:143–51. 10.1111/aogs.12029 23066728

[48] Strunz EC, Addiss DG, Stocks ME, Ogden S, Utzinger J, Freeman MC. Water, sanitation, hygiene, and soil-transmitted helminth infection: a systematic review and meta-analysis. PLoS Med. 2014;11:e1001620 10.1371/journal.pmed.1001620 24667810 PMC3965411

[49] Dangour AD, Watson L, Cumming O, Boisson S, Che Y, Velleman Y, et al Interventions to improve water quality and supply, sanitation and hygiene practices, and their effects on the nutritional status of children. Cochrane Database Syst Rev. 2013;(8):CD009382 10.1002/14651858.CD009382.pub2 23904195 PMC11608819

[50] UNICEF. Water, sanitation and hygiene: UNICEF in action. New York: UNICEF; 2014 1 10 [cited 2016 Mar 25]. Available from: http://www.unicef.org/wash/index_action.html.

[51] Lassi ZS, Das JK, Zahid G, Imdad A, Bhutta ZA. Impact of education and provision of complementary feeding on growth and morbidity in children less than 2 y of age in developing countries: a systematic review. BMC Public Health. 2013;13:S13 10.1186/1471-2458-13-S3-S13 24564534 PMC3847349

[52] Das JK, Tripathi A, Ali A, Hassan A, Dojosoeandy C, Bhutta ZA. Vaccines for the prevention of diarrhea due to cholera, shigella, ETEC and rotavirus. BMC Public Health. 2013;13(Suppl 3):S11. 24564510 10.1186/1471-2458-13-S3-S11 PMC3847224

[53] Liu L, Oza S, Hogan D, Perin J, Rudan I, Lawn JE, et al Global, regional, and national causes of child mortality in 2000–13, with projections to inform post-2015 priorities: an updated systematic analysis. Lancet. 2015;385:430–40. 10.1016/S0140-6736(14)61698-6 25280870

[54] Shiau S, Arpadi S, Strehlau R, Martens L, Patel F, Coovadia A, et al Initiation of antiretroviral therapy before 6 months of age is associated with faster growth recovery in South African children perinatally infected with human immunodeficiency virus. J Pediatr. 2013;162:1138–45. 10.1016/j.jpeds.2012.11.025 23312691 PMC3640753

[55] Zhang J, Yu KF. What’s the relative risk?: a method of correcting the odds ratio in cohort studies of common outcomes. JAMA. 1998;280:1690–1. 9832001 10.1001/jama.280.19.1690

[56] Isanaka S, Duggan C, Fawzi WW. Patterns of postnatal growth in HIV-infected and HIV-exposed children. Nutr Rev. 2009;67:343–59. 10.1111/j.1753-4887.2009.00207.x 19519675 PMC2771338

[57] Zimmermann MB. The role of iodine in human growth and development. Semin Cell Dev Biol. 2011;22:645–52. 10.1016/j.semcdb.2011.07.009 21802524

[58] Taylor-Robinson DC, Maayan N, Soares-Weiser K, Donegan S, Garner P. Deworming drugs for soil-transmitted intestinal worms in children: effects on nutritional indicators, haemoglobin and school performance. Cochrane Database Syst Rev. 2012;(7):CD000371 10.1002/14651858.CD000371.pub4 22786473

[59] Victora CG, Barros FC, Kirkwood BR, Vaughan JP. Pneumonia, diarrhea, and growth in the first 4 y of life: a longitudinal study of 5914 urban Brazilian children. Am J Clin Nutr. 1990;52:391–6. 2375306 10.1093/ajcn/52.2.391

[60] Dicko A, Barry A, Dicko M, Diallo AI, Tembine I, Dicko Y, et al Malaria morbidity in children in the year after they had received intermittent preventive treatment of malaria in Mali: a randomized control trial. PLoS ONE. 2011;6:e23390 10.1371/journal.pone.0023390 21858096 PMC3155530

[61] Pasricha S-R, Hayes E, Kalumba K, Biggs B-A. Effect of daily iron supplementation on health in children aged 4–23 months: a systematic review and meta-analysis of randomised controlled trials. Lancet Glob Health. 2013;1:e77–86. 10.1016/S2214-109X(13)70046-9 25104162

[62] Imhoff-Kunsch B, Briggs V. Antihelminthics in pregnancy and maternal, newborn and child health. Paediatr Perinat Epidemiol. 2012;26(Suppl 1):223–38. 10.1111/j.1365-3016.2012.01280.x 22742613

[63] Ramakrishnan U, Aburto N, McCabe G, Martorell R. Multimicronutrient interventions but not vitamin A or iron interventions alone improve child growth: results of 3 meta-analyses. J Nutr. 2004;134:2592–602. 15465753 10.1093/jn/134.10.2592

