# Supplementary figures and images for: Risk Factors for Childhood Stunting in 137 Developing Countries: A Comparative Risk Assessment Analysis at Global, Regional, and Country Levels
Source: PLoS Med. 2016 Nov 1;13(11):e1002164. doi: 10.1371/journal.pmed.1002164 (PMC5089547; doi:10.1371/journal.pmed.1002164)

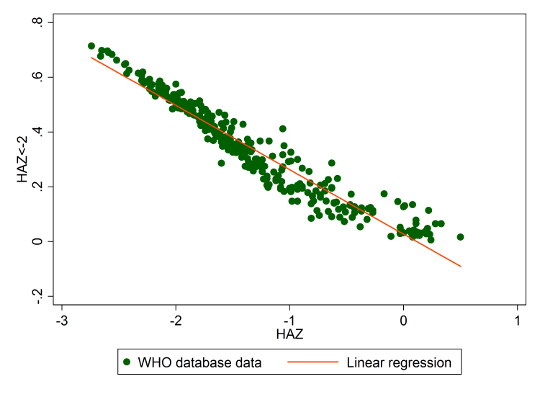

Supplement: S1 Fig — (TIF) [file pmed.1002164.s002.tif]

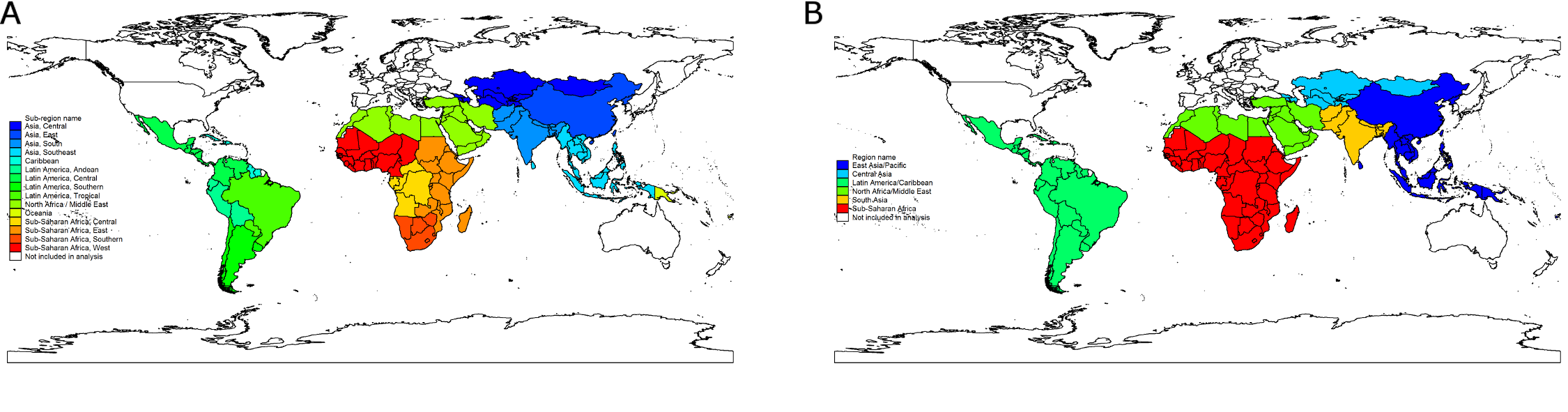

Supplement: S3 Fig — By sub-region (A) and by region (B). (TIF) [file pmed.1002164.s004.tif]

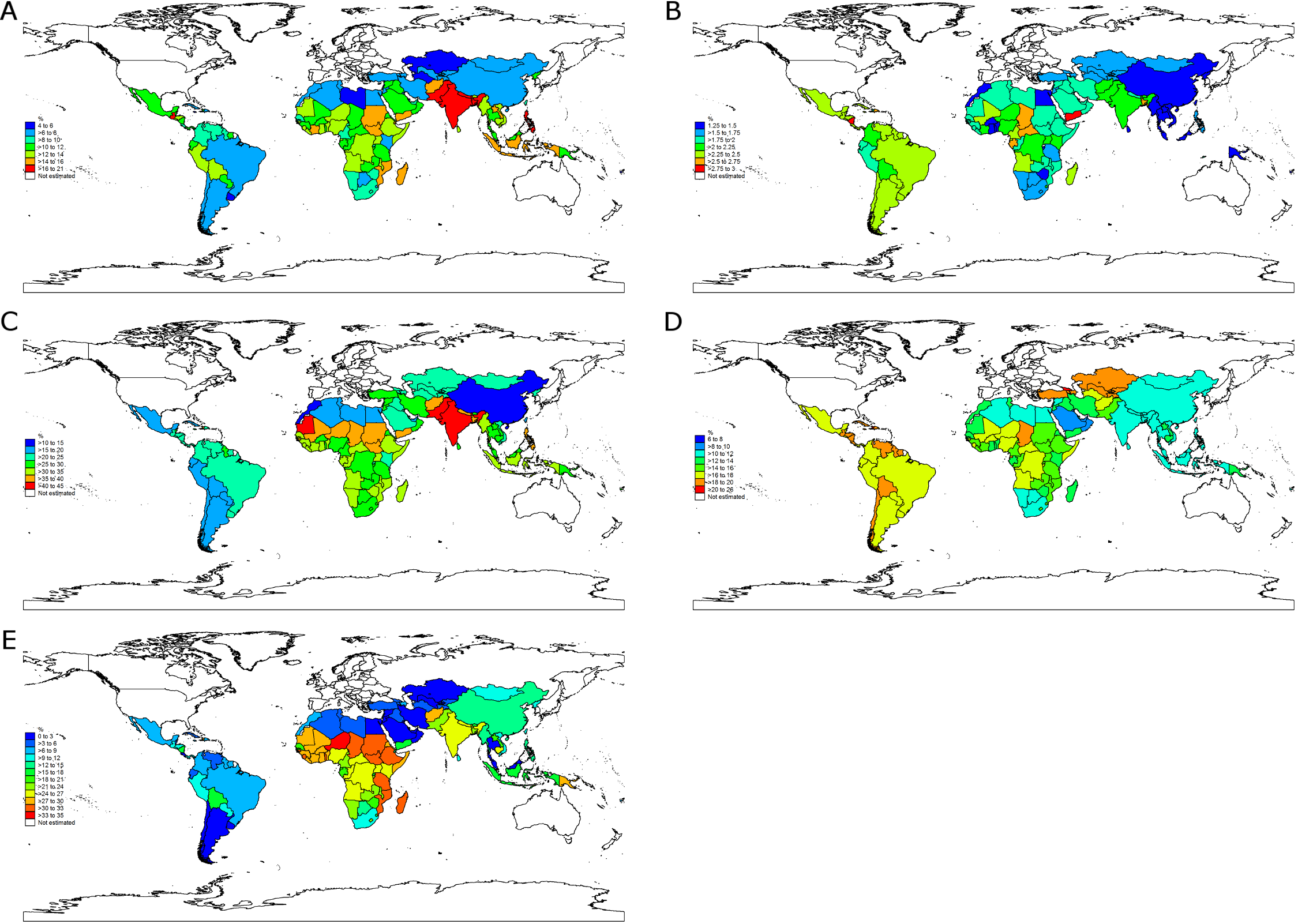

Supplement: S4 Fig — (A) Maternal nutrition and infection. (B) Teenage motherhood and short birth intervals. (C) FGR and preterm birth. (D) Child nutrition and infection. (E) Environmental factors. (TIF) [file pmed.1002164.s005.tif]
