# Supplementary material for: Risk Factors for Childhood Stunting in 137 Developing Countries: A Comparative Risk Assessment Analysis at Global, Regional, and Country Levels
Source: PLoS Med. 2016 Nov 1;13(11):e1002164. doi: 10.1371/journal.pmed.1002164 (PMC5089547; doi:10.1371/journal.pmed.1002164)
Supplement: S2 Table — (DOCX) [file pmed.1002164.s008.docx]

| **Level of evidence** | **Empirical evidence** |
| --- | --- |
| Convincing | Evidence from more than one study type (e.g. randomized trials and cohort studies).  • Evidence from at least two independent randomized trials or cohort studies  • No substantial unexplained heterogeneity  • Good quality studies to exclude with confidence the possibility of random or systematic error, including confounding, measurement error, and selection bias  • Presence of a plausible biological gradient (‘dose response’)  • Strong and plausible experimental evidence (human studies or relevant animal models) |
| Probable | Evidence from at least two independent randomized trials, cohort studies, or at least five case-control or cross sectional studies.  • Evidence from case-control studies should only be considered if there is consensus among the panel^a^ that potential for bias is reasonably low.  • Evidence from cross-sectional studies should only be considered if there is consensus among the panel^a^ that exposure could not possibly have been affected by the outcome.  • No substantial unexplained heterogeneity between or within study types in the presence or absence of an association, or direction of effect  • Good quality studies to exclude with confidence the possibility that the observed association results from random or systematic error, including confounding, measurement error, and selection bias  • Evidence for biological plausibility |
| Limited-suggestive | Evidence from at least two independent randomized trials or cohort studies or at least five case-control or cross-sectional studies.  • Evidence from case-control studies should only be considered if there is consensus among the panel^a^ that potential for bias is reasonably low.  • Evidence from cross-sectional studies should only be considered if there is consensus among the panel^a^ that exposure could not possibly have been affected by the outcome.  • The direction of effect is generally consistent though some unexplained heterogeneity may be present.  • Evidence for biological plausibility |
| Limited-no conclusion | Evidence is so limited that no firm conclusion can be made. |

# ^a^ The panel consisted of the members of our core Saving Brains Research Team.
