# Supplementary material for: Risk Factors for Childhood Stunting in 137 Developing Countries: A Comparative Risk Assessment Analysis at Global, Regional, and Country Levels
Source: PLoS Med. 2016 Nov 1;13(11):e1002164. doi: 10.1371/journal.pmed.1002164 (PMC5089547; doi:10.1371/journal.pmed.1002164)
Supplement: S3 Table — (DOCX) [file pmed.1002164.s009.docx]

| **Country** | **Stunting survey year** | **Age 2 : under 5 stunting ratio** | **HAZ survey year** | **Age 2 : under 5 HAZ ratio** |
| --- | --- | --- | --- | --- |
| Algeria | 2012 | 1.16 | 2005 | 1.12 |
| Armenia | 2010 | 1.03 | 2010 | 1.00 |
| Azerbaijan | 2013 | 0.99 | 2006 | 1.24 |
| Bangladesh | 2011 | 1.16 | 2011 | 1.11 |
| Belize | 2011 | 1.01 | 2011 | 1.12 |
| Benin | 2006 | 1.12 | 2006 | 1.08 |
| Bhutan | 2010 | 1.12 | 2010 | 1.23 |
| Bolivia | 2008 | 1.27 | 2008 | 1.20 |
| Botswana | 2007 | 1.27 | 2007 | 1.25 |
| Brazil | 2006 | 1.03 | 2006 | 1.68 |
| Burkina Faso | 2010 | 1.33 | 2010 | 1.34 |
| Burundi | 2010 | 1.13 | 2010 | 1.11 |
| Cambodia | 2010 | 1.13 | 2010 | 1.11 |
| Cameroon | 2011 | 1.23 | 2011 | 1.31 |
| Central African Republic | 2010 | 1.21 | 2006 | 1.26 |
| Chad | 2010 | 1.37 | 2004 | 1.48 |
| Chile | 2014 | 1.00 |  |  |
| China | 2002 | 1.38 | 2002 | 1.51 |
| Colombia | 2009 | 1.16 | 2009 | 1.15 |
| Comoros | 2012 | 1.04 | 2012 | 1.12 |
| Congo | 2011 | 1.17 | 2011 | 1.20 |
| Congo, the Democratic Republic of the | 2013 | 1.15 | 2013 | 1.16 |
| Côte d'Ivoire | 2011 | 1.18 | 2011 | 1.24 |
| Djibouti | 2012 | 1.10 | 2012 | 1.16 |
| Dominican Republic | 2013 | 1.14 | 2013 | 1.07 |
| Ecuador | 2012 | 1.15 | 2012 | 1.17 |
| Egypt | 2014 | 1.01 | 2014 | 1.25 |
| El Salvador | 2008 | 1.11 | 2008 | 1.12 |
| Equatorial Guinea | 2004 | 1.14 | 2004 | 1.27 |
| Eritrea | 2010 | 1.41 | 2002 | 1.38 |
| Ethiopia | 2014 | 1.28 | 2010 | 1.26 |
| Fiji | 2004 | 1.69 | 2004 | -3.07 |
| Gabon | 2012 | 1.21 | 2012 | 1.23 |
| Gambia | 2010 | 1.41 | 2005 | 1.32 |
| Georgia | 2009 | 1.15 | 2005 | 1.10 |
| Ghana | 2011 | 1.24 | 2008 | 1.34 |
| Guatemala | 2008 | 1.15 | 2008 | 1.10 |
| Guinea | 2012 | 1.28 | 2012 | 1.39 |
| Guinea-Bissau | 2010 | 1.29 | 2008 | 1.34 |
| Guyana | 2009 | 1.02 | 2009 | 1.10 |
| Haiti | 2012 | 1.25 | 2012 | 1.29 |
| Honduras | 2011 | 1.21 | 2011 | 1.16 |
| India | 2005 | 1.14 | 2005 | 1.14 |
| Indonesia | 2013 | 1.14 | 2013 | 1.16 |
| Iraq | 2011 | 1.09 | 2006 | 1.12 |
| Jamaica | 2012 | 0.74 | 2004 | 1.31 |
| Jordan | 2012 | 1.14 | 2012 | 1.40 |
| Kazakhstan | 2010 | 1.10 | 2010 | 1.59 |
| Kenya | 2008 | 1.19 | 2008 | 1.19 |
| Korea, Democratic People's Republic of | 2012 | 1.32 | 2009 | 1.00 |
| Kuwait | 2014 | 0.53 | 2014 | -15.00 |
| Kyrgyzstan | 2012 | 1.28 | 2012 | 1.38 |
| Lao People's Democratic Republic | 2011 | 1.15 | 2011 | 1.14 |
| Lebanon | 2004 | 1.05 | 2004 | 0.85 |
| Lesotho | 2009 | 1.14 | 2009 | 1.14 |
| Liberia | 2013 | 1.13 | 2013 | 1.29 |
| Libyan Arab Jamahiriya | 2007 | 1.01 | 2007 | 0.91 |
| Madagascar | 2003 | 1.09 | 2003 | 1.09 |
| Malawi | 2010 | 1.13 | 2010 | 1.09 |
| Maldives | 2009 | 0.94 | 2009 | 0.99 |
| Mali | 2006 | 1.27 | 2006 | 1.32 |
| Mauritania | 2012 | 1.25 | 2012 | 1.28 |
| Mexico | 2011 | 1.09 | 2011 | 1.04 |
| Mongolia | 2010 | 1.33 | 2010 | 1.53 |
| Morocco | 2010 | 1.38 | 2010 | 1.52 |
| Mozambique | 2011 | 1.14 | 2011 | 1.09 |
| Myanmar | 2009 | 1.25 | 2003 | 1.16 |
| Namibia | 2013 | 1.41 | 2013 | 1.46 |
| Nepal | 2011 | 1.27 | 2011 | 1.20 |
| Nicaragua | 2006 | 1.18 | 2006 | 1.18 |
| Niger | 2012 | 1.29 | 2012 | 1.26 |
| Nigeria | 2014 | 1.16 | 2014 | 1.22 |
| Oman | 2009 | 1.15 | 2009 | 1.15 |
| Pakistan | 2012 | 1.18 | 2012 | 1.21 |
| Panama | 2008 | 1.04 |  |  |
| Papua New Guinea | 2009 | 1.04 | 2009 | 1.09 |
| Paraguay | 2011 | 1.01 | 2011 | 1.35 |
| Peru | 2012 | 1.04 | 2012 | 1.03 |
| Philippines | 2011 | 1.17 | 2011 | 1.15 |
| Rwanda | 2010 | 1.14 | 2010 | 1.13 |
| Sao Tome and Principe | 2008 | 0.91 | 2008 | 1.33 |
| Saudi Arabia | 2004 | 1.29 | 2004 | 1.82 |
| Senegal | 2012 | 1.17 | 2012 | 1.18 |
| Sierra Leone | 2013 | 1.22 | 2013 | 1.16 |
| Somalia | 2006 | 1.30 | 2006 | 1.41 |
| South Africa | 2003 | 1.11 | 2003 | 1.07 |
| Sri Lanka | 2009 | 1.07 | 2009 | 1.11 |
| Sudan | 2006 | 1.22 | 2006 | 1.21 |
| Suriname | 2010 | 1.03 | 2010 | 1.47 |
| Swaziland | 2010 | 1.26 | 2010 | 1.16 |
| Syrian Arab Republic | 2009 | 1.19 | 2009 | 1.25 |
| Tajikistan | 2012 | 1.22 | 2012 | 1.24 |
| Tanzania, United Republic of | 2010 | 1.34 | 2009 | 1.19 |
| Thailand | 2012 | 1.00 | 2005 | 0.97 |
| Timor-Leste | 2009 | 1.07 | 2009 | 1.09 |
| Togo | 2013 | 1.26 | 2013 | 1.26 |
| Trinidad and Tobago | 2000 | 1.25 | 2000 | 0.48 |
| Tunisia | 2011 | 1.00 | 2006 | 1.34 |
| Turkey | 2013 | 1.39 | 2003 | 1.35 |
| Uganda | 2011 | 1.26 | 2011 | 1.27 |
| Uruguay | 2004 | 0.97 | 2004 | 0.78 |
| Uzbekistan | 2006 | 1.33 | 2006 | 1.49 |
| Vanuatu | 2007 | 1.33 | 2007 | 1.29 |
| Viet Nam | 2010 | 1.22 | 2010 | 1.20 |
