# Supplementary material for: Risk Factors for Childhood Stunting in 137 Developing Countries: A Comparative Risk Assessment Analysis at Global, Regional, and Country Levels
Source: PLoS Med. 2016 Nov 1;13(11):e1002164. doi: 10.1371/journal.pmed.1002164 (PMC5089547; doi:10.1371/journal.pmed.1002164)
Supplement: S4 Table — (DOCX) [file pmed.1002164.s010.docx]

|  | **50% mediation** | | **No mediation** | | **100% mediation** | |
| --- | --- | --- | --- | --- | --- | --- |
| **Region** | **PAF (Percent)** | **Number Stunted (Thousands)** | **PAF (Percent)** | **Number Stunted (Thousands)** | **PAF (Percent)** | **Number Stunted (Thousands)** |
| All developing countries | 13.5  (6.0, 21.3) | 5962  (2586, 9444) | 14.0  (6.5, 21.7) | 6163  (2799, 9659) | 13.1  (5.3, 20.9) | 5765  (2396, 9212) |
| East Asia and Pacific | 10.9  (4.7, 17.3) | 802  (347, 1328) | 11.4  (5.2, 17.9) | 837  (376, 1397) | 10.5  (4.3, 16.7) | 768  (304, 1285) |
| South Asia | 12.3  (5.2, 20.1) | 2053  (880, 3536) | 12.8  (5.8, 20.4) | 2130  (967, 3608) | 11.9  (4.6, 19.7) | 1975  (805, 3426) |
| Central Asia | 18.9  (8.3, 29.1) | 85  (36, 135) | 19.2  (8.7, 29.7) | 87  (38, 136) | 18.6  (8.1, 28.8) | 84  (35, 133) |
| North Africa and Middle East | 12.9  (5.7, 20.6) | 333  (148, 545) | 13.6  (6.4, 21.3) | 350  (161, 562) | 12.3  (5.2, 20.2) | 318  (131, 528) |
| Sub-Saharan Africa | 15.4  (6.9, 24.1) | 2371  (1066, 3715) | 15.9  (7.2, 24.5) | 2433  (1105, 3768) | 15.0  (6.4, 23.8) | 2307  (996, 3668) |
| Latin America and Caribbean | 18.1  (7.9, 27.8) | 318  (136, 495) | 18.6  (8.5, 28.2) | 327  (146, 508) | 17.8  (7.4, 27.7) | 312  (130, 490) |
