# Supplementary material for: Risk Factors for Childhood Stunting in 137 Developing Countries: A Comparative Risk Assessment Analysis at Global, Regional, and Country Levels
Source: PLoS Med. 2016 Nov 1;13(11):e1002164. doi: 10.1371/journal.pmed.1002164 (PMC5089547; doi:10.1371/journal.pmed.1002164)
Supplement: S5 Table — The leading risk factor is ranked one and colored bright red, and the risk factor with the smallest number of attributable cases is ranked 16 and colored dark green. Risk factors are ordered with respect to their global impact on stunting, and countries are ordered with respect to the number of stunted children at age 2 y in 2010. Untreated HIV infection is not included because exposure data were available for only 45 countries. (DOCX) [file pmed.1002164.s011.docx]

| Country | TSGA | Unim-proved sanitation | Child-hood diarrhea | Maternal short stature | PAGA | PSGA | Biomass fuel use | Maternal anemia | Maternal under-weight | Unim-proved water | Non-exclusive breast-feeding | Child-hood zinc deficiency | Short birth intervals | Teenage mother-hood | Dis-continued breast-feeding | Maternal malaria |
| --- | --- | --- | --- | --- | --- | --- | --- | --- | --- | --- | --- | --- | --- | --- | --- | --- |
| India | 1 | 2 | 4 | 3 | 7 | 6 | 5 | 9 | 8 | 14 | 11 | 10 | 12 | 13 | 15 | 16 |
| Nigeria | 1 | 2 | 3 | 4 | 5 | 6 | 7 | 9 | 12 | 8 | 11 | 16 | 13 | 14 | 15 | 10 |
| Pakistan | 1 | 2 | 3 | 4 | 7 | 5 | 6 | 8 | 9 | 13 | 10 | 14 | 11 | 15 | 12 | 16 |
| Ethiopia | 1 | 2 | 3 | 5 | 6 | 8 | 4 | 10 | 9 | 7 | 11 | 15 | 12 | 14 | 13 | 16 |
| Indonesia | 1 | 3 | 4 | 2 | 6 | 5 | 7 | 10 | 9 | 12 | 11 | 8 | 15 | 14 | 13 | 16 |
| China | 4 | 1 | 2 | 3 | 6 | 5 | 11 | 14 | 15 | 12 | 7 | 9 | 10 | 13 | 8 | 16 |
| Bangladesh | 1 | 2 | 4 | 3 | 7 | 6 | 5 | 9 | 8 | 11 | 12 | 13 | 14 | 10 | 15 | 16 |
| Democratic Republic of the Congo | 2 | 1 | 3 | 4 | 5 | 6 | 7 | 10 | 13 | 8 | 12 | 11 | 14 | 15 | 16 | 9 |
| United Republic of Tanzania | 2 | 1 | 3 | 4 | 5 | 6 | 7 | 9 | 12 | 8 | 10 | 11 | 14 | 13 | 15 | 16 |
| Philippines | 1 | 4 | 3 | 2 | 6 | 5 | 7 | 8 | 9 | 13 | 10 | 15 | 12 | 14 | 11 | 16 |
| Afghanistan | 1 | 2 | 3 | 4 | 7 | 6 | 5 | 9 | 10 | 8 | 11 | 15 | 14 | 12 | 13 | 16 |
| Uganda | 1 | 2 | 3 | 5 | 4 | 7 | 6 | 10 | 11 | 9 | 12 | 16 | 13 | 14 | 15 | 8 |
| Niger | 1 | 2 | 3 | 6 | 7 | 9 | 4 | 5 | 10 | 8 | 11 | 16 | 14 | 13 | 15 | 12 |
| Sudan | 1 | 2 | 3 | 4 | 6 | 7 | 5 | 8 | 10 | 9 | 11 | 16 | 13 | 14 | 12 | 15 |
| Mozambique | 2 | 1 | 3 | 4 | 5 | 7 | 6 | 9 | 11 | 8 | 12 | 15 | 16 | 13 | 14 | 10 |
| Yemen | 1 | 3 | 2 | 4 | 6 | 5 | 7 | 8 | 12 | 9 | 10 | 14 | 11 | 15 | 13 | 16 |
| Kenya | 2 | 1 | 3 | 5 | 4 | 6 | 7 | 10 | 14 | 8 | 9 | 12 | 13 | 11 | 15 | 16 |
| Madagascar | 2 | 1 | 3 | 4 | 5 | 7 | 6 | 10 | 9 | 8 | 12 | 15 | 13 | 11 | 14 | 16 |
| Myanmar | 1 | 4 | 2 | 3 | 7 | 5 | 6 | 9 | 8 | 10 | 11 | 13 | 12 | 15 | 14 | 16 |
| Angola | 1 | 2 | 3 | 4 | 5 | 6 | 8 | 9 | 11 | 7 | 10 | 13 | 14 | 12 | 15 | 16 |
| Nepal | 1 | 2 | 4 | 3 | 7 | 6 | 5 | 8 | 9 | 11 | 12 | 14 | 13 | 10 | 15 | 16 |
| Cote d’Ivoire | 1 | 2 | 3 | 7 | 4 | 8 | 5 | 9 | 13 | 11 | 10 | 14 | 16 | 12 | 15 | 6 |
| Chad | 1 | 2 | 3 | 7 | 5 | 6 | 4 | 8 | 10 | 9 | 11 | 14 | 13 | 12 | 16 | 15 |
| Mali | 1 | 2 | 3 | 8 | 5 | 6 | 4 | 7 | 10 | 9 | 12 | 15 | 14 | 13 | 16 | 11 |
| Viet Nam | 1 | 4 | 3 | 2 | 6 | 5 | 7 | 12 | 9 | 11 | 8 | 14 | 10 | 15 | 13 | 16 |
| Malawi | 1 | 2 | 3 | 5 | 4 | 6 | 7 | 9 | 13 | 10 | 12 | 11 | 15 | 14 | 16 | 8 |
| Burkina Faso | 2 | 1 | 3 | 8 | 4 | 6 | 5 | 9 | 13 | 10 | 11 | 12 | 15 | 14 | 16 | 7 |
| Cameroon | 1 | 2 | 3 | 6 | 4 | 5 | 7 | 8 | 16 | 9 | 11 | 12 | 14 | 13 | 15 | 10 |
| Zambia | 1 | 2 | 3 | 5 | 4 | 6 | 7 | 9 | 12 | 8 | 10 | 11 | 15 | 13 | 16 | 14 |
| Mexico | 2 | 4 | 1 | 3 | 5 | 6 | 13 | 12 | 15 | 14 | 7 | 9 | 11 | 10 | 8 | 16 |
| Ghana | 2 | 1 | 3 | 5 | 4 | 6 | 7 | 9 | 12 | 10 | 11 | 13 | 15 | 14 | 16 | 8 |
| Burundi | 1 | 3 | 2 | 4 | 5 | 7 | 6 | 9 | 10 | 8 | 13 | 11 | 12 | 14 | 16 | 15 |
| Egypt | 2 | 6 | 1 | 3 | 4 | 5 | 16 | 9 | 15 | 14 | 7 | 8 | 10 | 11 | 12 | 13 |
| Somalia | 1 | 2 | 3 | 4 | 5 | 8 | 7 | 10 | 12 | 6 | 9 | 15 | 13 | 14 | 11 | 16 |
| Guinea | 2 | 1 | 3 | 5 | 4 | 7 | 6 | 8 | 12 | 10 | 11 | 14 | 15 | 13 | 16 | 9 |
| South Africa | 1 | 3 | 2 | 4 | 5 | 6 | 12 | 8 | 14 | 13 | 7 | 10 | 15 | 11 | 9 | 16 |
| Brazil | 2 | 3 | 1 | 4 | 6 | 5 | 14 | 10 | 13 | 15 | 8 | 11 | 12 | 9 | 7 | 16 |
| Turkey | 1 | 6 | 2 | 5 | 4 | 3 | 14 | 10 | 13 | 15 | 9 | 7 | 11 | 12 | 8 | 16 |
| Iraq | 1 | 5 | 2 | 3 | 6 | 4 | 15 | 12 | 14 | 10 | 7 | 11 | 8 | 13 | 9 | 16 |
| Guatemala | 3 | 4 | 1 | 2 | 6 | 5 | 7 | 12 | 15 | 14 | 9 | 13 | 10 | 11 | 8 | 16 |
| Benin | 2 | 1 | 3 | 4 | 6 | 7 | 5 | 9 | 11 | 10 | 12 | 14 | 15 | 13 | 16 | 8 |
| Zimbabwe | 1 | 2 | 3 | 6 | 4 | 5 | 7 | 10 | 13 | 11 | 9 | 8 | 16 | 12 | 14 | 15 |
| Iran (Islamic Republic of) | 1 | 7 | 2 | 5 | 4 | 3 | 16 | 10 | 13 | 14 | 9 | 6 | 8 | 12 | 11 | 15 |
| Cambodia | 2 | 1 | 3 | 4 | 6 | 5 | 7 | 9 | 10 | 8 | 11 | 14 | 13 | 15 | 12 | 16 |
| Senegal | 1 | 2 | 3 | 8 | 4 | 7 | 5 | 6 | 10 | 9 | 11 | 12 | 15 | 13 | 16 | 14 |
| Rwanda | 2 | 3 | 1 | 4 | 5 | 6 | 7 | 13 | 12 | 8 | 11 | 9 | 10 | 15 | 14 | 16 |
| Sierra Leone | 1 | 2 | 3 | 4 | 6 | 7 | 5 | 10 | 12 | 8 | 11 | 13 | 16 | 14 | 15 | 9 |
| Peru | 3 | 4 | 1 | 2 | 5 | 6 | 7 | 14 | 15 | 10 | 8 | 13 | 12 | 11 | 9 | 16 |
| Democratic People’s Republic of Korea | 1 | 4 | 2 | 3 | 6 | 5 | 7 | 11 | 12 | 15 | 8 | 9 | 10 | 14 | 13 | 16 |
| Morocco | 2 | 4 | 1 | 3 | 5 | 6 | 16 | 12 | 14 | 9 | 7 | 10 | 11 | 13 | 8 | 15 |
| Eritrea | 2 | 1 | 3 | 4 | 5 | 6 | 7 | 10 | 9 | 8 | 13 | 15 | 11 | 12 | 14 | 16 |
| Uzbekistan | 2 | 7 | 1 | 3 | 5 | 4 | 14 | 10 | 15 | 11 | 6 | 12 | 9 | 13 | 8 | 16 |
| Haiti | 1 | 3 | 2 | 7 | 6 | 5 | 4 | 8 | 12 | 10 | 9 | 13 | 14 | 15 | 11 | 16 |
| Syrian Arab Republic | 1 | 6 | 2 | 3 | 5 | 4 | 16 | 9 | 14 | 12 | 7 | 10 | 8 | 13 | 11 | 15 |
| Colombia | 2 | 4 | 1 | 3 | 5 | 6 | 11 | 10 | 15 | 14 | 7 | 12 | 13 | 9 | 8 | 16 |
| Algeria | 1 | 6 | 2 | 3 | 4 | 5 | 16 | 11 | 15 | 12 | 8 | 7 | 10 | 13 | 9 | 14 |
| Togo | 2 | 1 | 3 | 5 | 4 | 6 | 7 | 10 | 12 | 8 | 11 | 13 | 15 | 14 | 16 | 9 |
| Central African Republic | 1 | 2 | 3 | 5 | 4 | 7 | 6 | 8 | 12 | 9 | 10 | 16 | 14 | 13 | 15 | 11 |
| Papua New Guinea | 2 | 1 | 3 | 4 | 8 | 6 | 7 | 9 | 13 | 5 | 10 | 15 | 11 | 14 | 12 | 16 |
| Lao People’s Democratic Republic | 1 | 4 | 2 | 3 | 6 | 5 | 7 | 10 | 11 | 8 | 9 | 14 | 12 | 15 | 13 | 16 |
| Thailand | 1 | 11 | 2 | 3 | 5 | 4 | 9 | 10 | 12 | 15 | 7 | 6 | 13 | 14 | 8 | 16 |
| Tajikistan | 2 | 8 | 1 | 4 | 5 | 3 | 10 | 12 | 15 | 6 | 7 | 13 | 11 | 14 | 9 | 16 |
| Liberia | 2 | 1 | 3 | 4 | 5 | 7 | 6 | 8 | 12 | 10 | 11 | 13 | 16 | 14 | 15 | 9 |
| Bolivia | 4 | 2 | 1 | 3 | 6 | 5 | 13 | 12 | 15 | 10 | 7 | 14 | 11 | 9 | 8 | 16 |
| Malaysia | 1 | 6 | 2 | 3 | 5 | 4 | 16 | 10 | 11 | 14 | 8 | 7 | 12 | 13 | 9 | 15 |
| Ecuador | 3 | 4 | 1 | 2 | 6 | 5 | 14 | 11 | 15 | 12 | 8 | 13 | 10 | 9 | 7 | 16 |
| Sri Lanka | 1 | 7 | 3 | 2 | 5 | 4 | 6 | 10 | 9 | 11 | 13 | 8 | 12 | 15 | 14 | 16 |
| Mauritania | 1 | 2 | 3 | 6 | 5 | 7 | 4 | 8 | 10 | 9 | 11 | 16 | 14 | 13 | 15 | 12 |
| Congo | 2 | 1 | 3 | 6 | 4 | 5 | 7 | 8 | 12 | 9 | 10 | 14 | 16 | 13 | 15 | 11 |
| Honduras | 2 | 4 | 1 | 3 | 6 | 5 | 7 | 12 | 15 | 13 | 8 | 11 | 14 | 10 | 9 | 16 |
| Venezuela | 2 | 6 | 1 | 3 | 5 | 4 | 16 | 11 | 14 | 13 | 7 | 8 | 12 | 10 | 9 | 15 |
| Saudi Arabia | 1 | 12 | 3 | 2 | 6 | 4 | 16 | 7 | 14 | 13 | 9 | 5 | 8 | 11 | 10 | 15 |
| Kazakhstan | 2 | 8 | 1 | 5 | 4 | 3 | 13 | 10 | 14 | 15 | 6 | 12 | 9 | 11 | 7 | 16 |
| Argentina | 2 | 8 | 1 | 3 | 5 | 4 | 15 | 12 | 13 | 14 | 7 | 10 | 11 | 9 | 6 | 16 |
| Guinea-Bissau | 1 | 2 | 3 | 5 | 6 | 8 | 4 | 7 | 11 | 9 | 10 | 14 | 15 | 13 | 16 | 12 |
| Azerbaijan | 2 | 3 | 1 | 4 | 6 | 5 | 14 | 12 | 15 | 10 | 7 | 9 | 11 | 13 | 8 | 16 |
| Nicaragua | 2 | 3 | 1 | 4 | 5 | 6 | 7 | 14 | 15 | 13 | 8 | 11 | 12 | 10 | 9 | 16 |
| Taiwan | 1 | 3 | 2 | 4 | 6 | 5 | 9 | 12 | 14 | 15 | 8 | 7 | 11 | 13 | 10 | 16 |
| Gambia | 1 | 3 | 2 | 7 | 5 | 6 | 4 | 8 | 9 | 13 | 12 | 10 | 14 | 11 | 16 | 15 |
| Kyrgyzstan | 2 | 6 | 1 | 5 | 4 | 3 | 9 | 12 | 15 | 11 | 7 | 14 | 10 | 13 | 8 | 16 |
| Timore Leste | 1 | 2 | 4 | 3 | 6 | 5 | 7 | 11 | 9 | 8 | 13 | 14 | 10 | 15 | 12 | 16 |
| Lesotho | 2 | 1 | 3 | 4 | 5 | 6 | 7 | 11 | 14 | 9 | 8 | 10 | 15 | 12 | 13 | 16 |
| Namibia | 1 | 2 | 3 | 6 | 4 | 5 | 7 | 8 | 10 | 14 | 9 | 13 | 15 | 12 | 11 | 16 |
| El Salvador | 2 | 4 | 1 | 3 | 6 | 5 | 11 | 12 | 15 | 14 | 7 | 10 | 13 | 9 | 8 | 16 |
| Dominican Republic | 2 | 6 | 1 | 3 | 5 | 4 | 15 | 11 | 14 | 12 | 7 | 8 | 13 | 10 | 9 | 16 |
| Turkmenistan | 2 | 8 | 1 | 5 | 4 | 3 | 16 | 11 | 14 | 9 | 6 | 13 | 10 | 12 | 7 | 15 |
| Paraguay | 2 | 4 | 1 | 3 | 6 | 5 | 9 | 13 | 15 | 12 | 7 | 14 | 11 | 10 | 8 | 16 |
| State of Palestine | 2 | 7 | 1 | 3 | 5 | 4 | 15 | 11 | 14 | 12 | 8 | 6 | 10 | 13 | 9 | 16 |
| Botswana | 1 | 3 | 2 | 6 | 4 | 5 | 7 | 9 | 13 | 16 | 8 | 11 | 14 | 12 | 10 | 15 |
| Jordan | 1 | 11 | 2 | 5 | 4 | 3 | 15 | 10 | 14 | 12 | 7 | 6 | 9 | 13 | 8 | 16 |
| Swaziland | 1 | 3 | 2 | 6 | 4 | 5 | 7 | 11 | 14 | 8 | 9 | 13 | 15 | 10 | 12 | 16 |
| Libyan Arab Jamahiriya | 2 | 7 | 1 | 4 | 3 | 5 | 16 | 13 | 15 | 9 | 8 | 6 | 11 | 12 | 10 | 14 |
| Gabon | 1 | 2 | 3 | 6 | 4 | 5 | 10 | 7 | 12 | 13 | 8 | 16 | 15 | 9 | 11 | 14 |
| Comoros | 1 | 2 | 3 | 4 | 5 | 7 | 6 | 8 | 9 | 15 | 10 | 12 | 11 | 14 | 13 | 16 |
| Tunisia | 2 | 7 | 1 | 4 | 3 | 6 | 16 | 11 | 15 | 13 | 8 | 5 | 10 | 12 | 9 | 14 |
| Mongolia | 3 | 2 | 1 | 6 | 5 | 4 | 7 | 13 | 15 | 8 | 10 | 14 | 9 | 12 | 11 | 16 |
| Equatorial Guinea | 1 | 3 | 2 | 4 | 5 | 6 | 7 | 9 | 14 | 8 | 10 | 13 | 16 | 12 | 11 | 15 |
| Panama | 2 | 4 | 1 | 3 | 5 | 6 | 13 | 11 | 15 | 14 | 7 | 9 | 12 | 10 | 8 | 16 |
| Cuba | 2 | 7 | 1 | 3 | 5 | 6 | 15 | 12 | 14 | 13 | 9 | 4 | 11 | 10 | 8 | 16 |
| Djibouti | 1 | 3 | 2 | 4 | 5 | 6 | 12 | 8 | 9 | 15 | 7 | 11 | 13 | 14 | 10 | 16 |
| Georgia | 2 | 9 | 1 | 7 | 5 | 4 | 10 | 12 | 15 | 14 | 6 | 3 | 11 | 13 | 8 | 16 |
| Oman | 1 | 8 | 4 | 2 | 5 | 3 | 16 | 7 | 12 | 10 | 11 | 6 | 9 | 13 | 14 | 15 |
| Solomon Islands | 2 | 1 | 3 | 4 | 6 | 5 | 7 | 8 | 14 | 9 | 12 | 10 | 11 | 15 | 13 | 16 |
| Armenia | 2 | 6 | 1 | 4 | 5 | 3 | 13 | 10 | 14 | 15 | 9 | 8 | 11 | 12 | 7 | 16 |
| Costa Rica | 2 | 9 | 1 | 3 | 5 | 4 | 14 | 12 | 15 | 13 | 7 | 6 | 11 | 10 | 8 | 16 |
| Bhutan | 1 | 2 | 3 | 4 | 6 | 5 | 7 | 8 | 10 | 15 | 12 | 11 | 14 | 9 | 13 | 16 |
| Lebanon | 1 | 8 | 2 | 6 | 5 | 4 | 16 | 11 | 13 | 14 | 7 | 3 | 10 | 12 | 9 | 15 |
| United Arab Emirates | 3 | 10 | 2 | 1 | 6 | 5 | 14 | 12 | 13 | 15 | 7 | 4 | 8 | 11 | 9 | 16 |
| Chile | 2 | 10 | 1 | 3 | 6 | 5 | 13 | 12 | 15 | 14 | 8 | 4 | 11 | 9 | 7 | 16 |
| Uruguay | 2 | 12 | 1 | 5 | 4 | 3 | 14 | 10 | 13 | 16 | 8 | 7 | 11 | 9 | 6 | 15 |
| Fiji | 1 | 5 | 2 | 6 | 4 | 3 | 8 | 9 | 15 | 14 | 10 | 7 | 12 | 13 | 11 | 16 |
| Guyana | 2 | 6 | 1 | 3 | 5 | 4 | 15 | 9 | 13 | 14 | 7 | 12 | 11 | 10 | 8 | 16 |
| Mauritius | 1 | 6 | 2 | 3 | 4 | 5 | 14 | 8 | 11 | 16 | 9 | 7 | 12 | 13 | 10 | 15 |
| Cape Verde | 3 | 1 | 2 | 6 | 4 | 5 | 9 | 10 | 15 | 12 | 8 | 7 | 14 | 11 | 13 | 16 |
| Vanuatu | 1 | 2 | 3 | 6 | 5 | 4 | 7 | 9 | 15 | 10 | 8 | 12 | 11 | 14 | 13 | 16 |
| Jamaica | 2 | 4 | 1 | 7 | 5 | 6 | 13 | 10 | 14 | 15 | 8 | 3 | 12 | 11 | 9 | 16 |
| Bahrain | 1 | 13 | 4 | 3 | 5 | 2 | 16 | 7 | 12 | 14 | 9 | 6 | 8 | 11 | 10 | 15 |
| Belize | 1 | 6 | 2 | 3 | 5 | 4 | 11 | 9 | 14 | 15 | 7 | 12 | 13 | 10 | 8 | 16 |
| Sao Tome and Principe | 2 | 1 | 3 | 4 | 5 | 6 | 7 | 8 | 13 | 12 | 10 | 9 | 15 | 11 | 14 | 16 |
| Qatar | 1 | 14 | 3 | 6 | 5 | 4 | 13 | 10 | 12 | 16 | 7 | 2 | 9 | 11 | 8 | 15 |
| Trinidad and Tobago | 1 | 7 | 3 | 6 | 5 | 4 | 15 | 10 | 14 | 13 | 8 | 2 | 12 | 9 | 11 | 16 |
| Suriname | 2 | 4 | 1 | 3 | 5 | 6 | 13 | 10 | 15 | 14 | 7 | 9 | 12 | 11 | 8 | 16 |
| Maldives | 1 | 6 | 3 | 2 | 5 | 4 | 13 | 7 | 8 | 15 | 9 | 10 | 12 | 14 | 11 | 16 |
| Kuwait | 1 | 15 | 2 | 5 | 4 | 3 | 14 | 10 | 12 | 13 | 7 | 6 | 9 | 11 | 8 | 16 |
| Kiribati | 2 | 1 | 3 | 4 | 6 | 5 | 7 | 11 | 15 | 8 | 10 | 9 | 12 | 14 | 13 | 16 |
| Micronesia (Federated States of) | 1 | 2 | 3 | 4 | 6 | 5 | 7 | 8 | 15 | 13 | 9 | 10 | 11 | 14 | 12 | 16 |
| St. Lucia | 2 | 3 | 1 | 6 | 4 | 5 | 15 | 9 | 13 | 12 | 7 | 14 | 11 | 10 | 8 | 16 |
| Bahamas | 1 | 6 | 3 | 2 | 5 | 4 | 15 | 7 | 13 | 14 | 8 | 12 | 11 | 9 | 10 | 16 |
| Barbados | 1 | 5 | 2 | 6 | 4 | 3 | 16 | 7 | 13 | 14 | 8 | 11 | 12 | 9 | 10 | 15 |
| Samoa | 2 | 3 | 1 | 5 | 6 | 4 | 12 | 14 | 15 | 13 | 8 | 7 | 10 | 11 | 9 | 16 |
| St. Vincent | 2 | 4 | 1 | 3 | 6 | 5 | 16 | 10 | 13 | 14 | 7 | 12 | 11 | 9 | 8 | 15 |
| Grenada | 2 | 6 | 1 | 8 | 4 | 3 | 16 | 10 | 13 | 14 | 5 | 12 | 11 | 9 | 7 | 15 |
| Tonga | 2 | 3 | 1 | 7 | 5 | 4 | 11 | 13 | 15 | 14 | 8 | 6 | 10 | 12 | 9 | 16 |
| Marshall Islands | 1 | 3 | 2 | 4 | 6 | 5 | 7 | 8 | 14 | 15 | 9 | 10 | 11 | 13 | 12 | 16 |
| Antigua and Barbuda | 2 | 6 | 1 | 3 | 5 | 4 | 15 | 11 | 13 | 14 | 7 | 12 | 10 | 9 | 8 | 16 |
| Seychelles | 1 | 5 | 2 | 6 | 3 | 4 | 16 | 10 | 12 | 14 | 8 | 7 | 11 | 13 | 9 | 15 |
| Dominica | 2 | 3 | 1 | 4 | 5 | 6 | 14 | 10 | 13 | 15 | 7 | 11 | 12 | 9 | 8 | 16 |
