# Supplementary material for: Risk Factors for Childhood Stunting in 137 Developing Countries: A Comparative Risk Assessment Analysis at Global, Regional, and Country Levels
Source: PLoS Med. 2016 Nov 1;13(11):e1002164. doi: 10.1371/journal.pmed.1002164 (PMC5089547; doi:10.1371/journal.pmed.1002164)
Supplement: S1 Text — (DOCX) [file pmed.1002164.s012.docx]

# **Methods used to identify source of evidence on effect sizes**

We utilized published systematic reviews to identify maternal and child risk factors with potential effects on child height-for-age z-scores (HAZ), stunting (HAZ<-2) or poor birth outcomes (low birth weight and small-for-gestational age) [1–3]. Systematic literature searches of PubMed, Cochrane Libraries, and all WHO Regional Databases were then conducted to identify the most up-to-date evidence (systematic reviews, meta-analyses, and pooling studies) for each of the identified risk factors utilizing relevant MeSH terms and keywords. The level of evidence for each risk factor was then classified by the study team to be ‘convincing’, ‘probable’, ‘limited-suggestive’, or ‘limited-no conclusion’ based on predetermined criteria (see S2 Table). Evidence defined as ‘convincing’ or ‘probable’ was then selected for inclusion in the model if country-level data on prevalence of the exposure were available globally. Risk factors with ‘convincing’ and ‘probable’ evidence of an effect but lacking global exposure or coverage data included child lead exposure, child arsenic exposure, and parental drug use.

**References**

1. Black RE, Allen LH, Bhutta ZA, Caulfield LE, de Onis M, Ezzati M, et al. Maternal and child undernutrition: global and regional exposures and health consequences. The Lancet. 2008;371:243–60.

2. Black RE, Victora CG, Walker SP, Bhutta ZA, Christian P, de Onis M, et al. Maternal and child undernutrition and overweight in low-income and middle-income countries. The Lancet. 2013;382:427–51.

3. Bhutta ZA, Das JK, Rizvi A, Gaffey MF, Walker N, Horton S, et al. Evidence-based interventions for improvement of maternal and child nutrition: what can be done and at what cost? Lancet. 2013;382:452–77.
