# Supplementary material for: Risk Factors for Childhood Stunting in 137 Developing Countries: A Comparative Risk Assessment Analysis at Global, Regional, and Country Levels
Source: PLoS Med. 2016 Nov 1;13(11):e1002164. doi: 10.1371/journal.pmed.1002164 (PMC5089547; doi:10.1371/journal.pmed.1002164)
Supplement: S2 Text — (DOCX) [file pmed.1002164.s013.docx]

**Description of conversion of stunting prevalence among children under 5 to stunting prevalence among children age 2**

To create the conversion factors, we used data from the WHO Database on Child Growth and Malnutrition [1]. Specifically, we used the latest nationally-representative survey since 2000 (for both sexes) from each country, for which both stunting prevalence among children age 0 to 59 months and among children age 24 to 35 months were available. This resulted in a dataset of 104 observations (from the same number of countries) from the 137 countries on our analysis list, from 2000 to 2014. We calculated country-specific ratios of stunting prevalence among children age 2 to stunting prevalence among children under 5. For those 33 countries (of the 137 developing countries in this analysis) without country-specific ratios, we used population-weighted means by sub-region. We then multiplied the country-specific stunting prevalence among children under 5 from the Nutrition Impact Model Study (NIMS) [2] by these ratios to convert the prevalence values to prevalence of stunting among children age 2. We used the same methodology to convert mean HAZ among children under 5 from NIMS to mean HAZ among children age 2 (using data from 102 surveys from the WHO Database on Child Growth and Malnutrition that had both stunting prevalence among children age 0 to 59 months and among children age 24 to 35 months). The estimates of mean HAZ among children age 2 were used in estimating the burden of stunting attributable to HIV and zinc deficiency, as described in the main paper. The country-specific ratios used (either directly or in population-weighted averages by sub-region for missing countries) are visible in S3 Table.

**References**

1. World Health Organization. Nutrition Landscape Information System (NLiS). 2016 [cited 2016 Apr 17]. Available from: http://apps.who.int/nutrition/landscape/search.aspx.

2. Stevens GA, Finucane MM, Paciorek CJ, Flaxman SR, White RA, Donner AJ, et al. Trends in mild, moderate, and severe stunting and underweight, and progress towards MDG 1 in 141 developing countries: a systematic analysis of population representative data. The Lancet. 2012;380:824–34.
