# Supplementary material for: Risk Factors for Childhood Stunting in 137 Developing Countries: A Comparative Risk Assessment Analysis at Global, Regional, and Country Levels
Source: PLoS Med. 2016 Nov 1;13(11):e1002164. doi: 10.1371/journal.pmed.1002164 (PMC5089547; doi:10.1371/journal.pmed.1002164)
Supplement: S3 Text — (DOCX) [file pmed.1002164.s014.docx]

# **Description of HAZ to stunting prevalence crosswalk**

In order to translate changes in HAZ to changes in stunting prevalence (which was necessary to estimate the effect of the risk factors of zinc deficiency and HIV), we used a linear regression model that allowed us to capture the relationship between HAZ and stunting prevalence. We used 309 (nationally representative, for both sexes) data points from the WHO Database on Child Growth and Malnutrition [1] from 120 countries and 15 years (2000-2014) where mean HAZ and stunting prevalence were estimated for the same population. The raw data and the model fit are presented in S1 Fig. We applied the regression parameters to estimates of HAZ in order to generate estimated stunting prevalence. Both the parameter and model uncertainty were incorporated into the uncertainty (1000 draws) associated with the analysis estimates.

**References**

1. World Health Organization. Nutrition Landscape Information System (NLiS). 2016 [cited 2016 Apr 17]. Available from: http://apps.who.int/nutrition/landscape/search.aspx.
