# Supplementary material for: Risk Factors for Childhood Stunting in 137 Developing Countries: A Comparative Risk Assessment Analysis at Global, Regional, and Country Levels
Source: PLoS Med. 2016 Nov 1;13(11):e1002164. doi: 10.1371/journal.pmed.1002164 (PMC5089547; doi:10.1371/journal.pmed.1002164)
Supplement: S4 Text — (DOCX) [file pmed.1002164.s015.docx]

**Systematic review of HAART and childhood growth**

No quantitative systematic review or meta-analysis was identified for the effect of child HIV on linear growth and as a result a full systematic review of individual studies was conducted. The effect size was estimated by a meta-analysis of 4 observational studies [1–4] during the pre-ART era in developing country settings of HIV-infected children under 2 years of age as compared to HIV-exposed uninfected children (HIV+ mother but infant not infected). This comparison was selected to minimize confounding, also only 1 or 2 low quality studies compare growth of HIV-infected children to children of HIV-negative mothers. As displayed in S2 Fig, we estimated the effect on HAZ of late HAART initiation for HIV-positive children age 2 to be -0.63.

**References**

1. Bailey RC, Kamenga MC, Nsuami MJ, Nieburg P, St Louis ME. Growth of children according to maternal and child HIV, immunological and disease characteristics: a prospective cohort study in Kinshasa, Democratic Republic of Congo. Int. J. Epidemiol. 1999;28:532–40.

2. McDonald CM, Manji KP, Kupka R, Bellinger DC, Spiegelman D, Kisenge R, et al. Stunting and wasting are associated with poorer psychomotor and mental development in HIV-exposed Tanzanian infants. J. Nutr. 2013;143:204–14.

3. Taha T, Nour S, Li Q, Kumwenda N, Kafulafula G, Nkhoma C, et al. The effect of human immunodeficiency virus and breastfeeding on the nutritional status of African children. Pediatr. Infect. Dis. J. 2010;29:514–8.

4. Webb AL, Manji K, Fawzi WW, Villamor E. Time-independent maternal and infant factors and time-dependent infant morbidities including HIV infection, contribute to infant growth faltering during the first 2 years of life. J. Trop. Pediatr. 2009;55:83–90.
