# Supplementary material for: Risk Factors for Childhood Stunting in 137 Developing Countries: A Comparative Risk Assessment Analysis at Global, Regional, and Country Levels
Source: PLoS Med. 2016 Nov 1;13(11):e1002164. doi: 10.1371/journal.pmed.1002164 (PMC5089547; doi:10.1371/journal.pmed.1002164)
Supplement: S5 Text — (DOCX) [file pmed.1002164.s016.docx]

Afghanistan

**Region: South Asia; Sub-region: Asia, South**

**Stunting prevalence among children age 2: 58.0%**

**Stunting cases among children age 2: 634,041**

**Population of children age 2: 1,092,454**

This country profile provides results for 2011 from the analysis presented in *Risk factors for childhood stunting in 137 developing countries: a comparative risk assessment analysis at global, regional, and country levels.*

**Table 1: Population attributable fraction (PAF in %), attributable stunting prevalence (percentage points) and number of stunting cases among 2-year olds attributable to individual risks and risk factor clusters (95% confidence intervals presented in parentheses)**

| **Description** | **PAF** | **Attributable stunting prevalence** | **Attributable stunting cases (thousands)** |
| --- | --- | --- | --- |
| **Maternal nutrition and infection** | **14.0 (11.5, 17.0)** | **8.1 (5.9, 10.8)** | **89.0 (64.8, 118.4)** |
| Maternal short stature | 8.4 (8.1, 8.8) | 4.9 (3.8, 6.1) | 53.5 (41.0, 67.0) |
| Maternal underweight | 3.1 (1.5, 4.9) | 1.8 (0.8, 3.0) | 19.6 (8.9, 33.2) |
| Maternal malaria | 0.0 (0.0, 0.0) | 0.0 (0.0, 0.0) | 0.0 (0.0, 0.0) |
| Maternal anemia | 3.1 (0.8, 5.8) | 1.8 (0.5, 3.5) | 19.7 (5.5, 38.6) |
| **Teenage motherhood and short birth intervals** | **2.2 (2.1, 2.3)** | **1.3 (1.0, 1.6)** | **14.1 (10.7, 17.6)** |
| Teenage motherhood | 1.3 (1.2, 1.3) | 0.7 (0.6, 0.9) | 8.0 (6.1, 10.1) |
| Short birth intervals | 1.0 (0.9, 1.0) | 0.6 (0.4, 0.7) | 6.1 (4.6, 7.7) |
| **Fetal growth restriction and preterm birth** | **38.1 (33.7, 42.1)** | **22.1 (16.4, 28.2)** | **241.5 (179.4, 307.9)** |
| Preterm, small-for-gestational age | 5.4 (0.8, 10.6) | 3.1 (0.5, 6.6) | 34.2 (5.2, 72.2) |
| Preterm, appropriate-for-gestational age | 4.8 (1.0, 8.9) | 2.8 (0.5, 5.4) | 30.5 (5.9, 59.2) |
| Term, small-for-gestational age | 31.2 (26.5, 36.5) | 18.1 (13.2, 23.9) | 197.8 (144.6, 261.3) |
| Low birth weight | 27.8 (23.8, 31.7) | 16.1 (11.8, 20.6) | 176 (129.4, 225.5) |
| **Child nutrition and infection** | **17.2 (6.5, 29.5)** | **9.9 (3.7, 17.5)** | **108.7 (40.2, 191.1)** |
| Childhood zinc deficiency | 0.6 (0.2, 1.1) | 0.3 (0.1, 0.7) | 3.8 (1.4, 7.1) |
| Childhood diarrhea | 16.9 (6.2, 29.3) | 9.8 (3.5, 17.3) | 107.1 (38.3, 189.2) |
| Non-exclusive breastfeeding | 2.1 (-0.6, 5.5) | 1.2 (-0.4, 3.1) | 13.3 (-3.9, 33.4) |
| HIV infection without HAART before 2 years | No data | No data | No data |
| Discontinued breastfeeding | 1.1 (0.1, 3.1) | 0.7 (0.1, 1.8) | 7.1 (0.7, 19.9) |
| **Water, sanitation and biomass fuel use** | **27.1 (23.9, 30.5)** | **15.7 (11.7, 20.3)** | **172 (128.3, 221.7)** |
| Unimproved sanitation | 19.0 (15.9, 22.0) | 11.0 (8.0, 14.5) | 120.3 (87.0, 158.7) |
| Unimproved water | 3.5 (2.0, 5.1) | 2.0 (1.1, 3.2) | 22.4 (12.2, 35.5) |
| Use of biomass fuels | 6.8 (4.8, 8.9) | 3.9 (2.6, 5.4) | 43.0 (28.2, 59.3) |

**Fig 1: Stunting cases among 2-year olds attributable to individual risk factors**


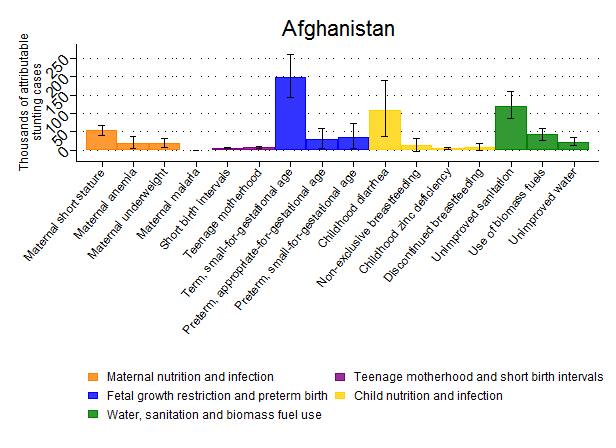


Algeria

**Region: North Africa/Middle East; Sub-region: North Africa / Middle East**

**Stunting prevalence among children age 2: 19.2%**

**Stunting cases among children age 2: 168,165**

**Population of children age 2: 874,884**

This country profile provides results for 2011 from the analysis presented in *Risk factors for childhood stunting in 137 developing countries: a comparative risk assessment analysis at global, regional, and country levels.*

**Table 1: Population attributable fraction (PAF in %), attributable stunting prevalence (percentage points) and number of stunting cases among 2-year olds attributable to individual risks and risk factor clusters (95% confidence intervals presented in parentheses)**

| **Description** | **PAF** | **Attributable stunting prevalence** | **Attributable stunting cases (thousands)** |
| --- | --- | --- | --- |
| **Maternal nutrition and infection** | **6.8 (6.0, 7.7)** | **1.3 (0.8, 1.8)** | **11.4 (7.1, 15.9)** |
| Maternal short stature | 5.3 (5.0, 5.6) | 1.0 (0.6, 1.4) | 8.9 (5.6, 12.2) |
| Maternal underweight | 0.4 (0.1, 0.7) | 0.1 (0.0, 0.1) | 0.6 (0.2, 1.2) |
| Maternal malaria | 0.4 (0.1, 0.6) | 0.1 (0.0, 0.1) | 0.6 (0.2, 1.1) |
| Maternal anemia | 0.8 (0.2, 1.7) | 0.2 (0.0, 0.3) | 1.4 (0.3, 3.0) |
| **Teenage motherhood and short birth intervals** | **1.8 (1.7, 1.9)** | **0.3 (0.2, 0.5)** | **3.0 (1.9, 4.1)** |
| Teenage motherhood | 0.5 (0.5, 0.6) | 0.1 (0.1, 0.1) | 0.9 (0.6, 1.2) |
| Short birth intervals | 1.2 (1.1, 1.3) | 0.2 (0.2, 0.3) | 2.1 (1.3, 2.9) |
| **Fetal growth restriction and preterm birth** | **17.9 (13.4, 22.1)** | **3.4 (2.0, 5.1)** | **30.1 (17.8, 44.4)** |
| Preterm, small-for-gestational age | 3.4 (1.0, 6.4) | 0.7 (0.2, 1.3) | 5.7 (1.4, 11.0) |
| Preterm, appropriate-for-gestational age | 4.7 (1.4, 8.1) | 0.9 (0.3, 1.7) | 7.9 (2.4, 15.0) |
| Term, small-for-gestational age | 10.8 (7.5, 14.6) | 2.1 (1.1, 3.2) | 18.2 (10.0, 27.9) |
| Low birth weight | 10.0 (8.3, 11.9) | 1.9 (1.2, 2.8) | 16.9 (10.4, 24.5) |
| **Child nutrition and infection** | **10.9 (3.9, 21.2)** | **2.1 (0.7, 4.1)** | **18.3 (6.1, 36.1)** |
| Childhood zinc deficiency | 2.2 (0.5, 8.8) | 0.4 (0.1, 1.7) | 3.7 (0.8, 14.8) |
| Childhood diarrhea | 9.9 (3.5, 19.7) | 1.9 (0.6, 3.8) | 16.7 (5.3, 33.1) |
| Non-exclusive breastfeeding | 1.9 (0.4, 4.2) | 0.4 (0.1, 0.9) | 3.1 (0.6, 7.5) |
| HIV infection without HAART before 2 years | No data | No data | No data |
| Discontinued breastfeeding | 1.3 (0.2, 3.2) | 0.3 (0.0, 0.7) | 2.2 (0.3, 5.7) |
| **Water, sanitation and biomass fuel use** | **3.6 (2.6, 4.7)** | **0.7 (0.4, 1.1)** | **6.1 (3.5, 9.3)** |
| Unimproved sanitation | 2.9 (2.0, 3.9) | 0.6 (0.3, 0.9) | 4.9 (2.7, 7.6) |
| Unimproved water | 0.8 (0.3, 1.4) | 0.1 (0.0, 0.3) | 1.3 (0.4, 2.5) |
| Use of biomass fuels | 0.0 (-0.1, 0.1) | 0.0 (0.0, 0.0) | 0.0 (-0.2, 0.2) |

**Fig 1: Stunting cases among 2-year olds attributable to individual risk factors**


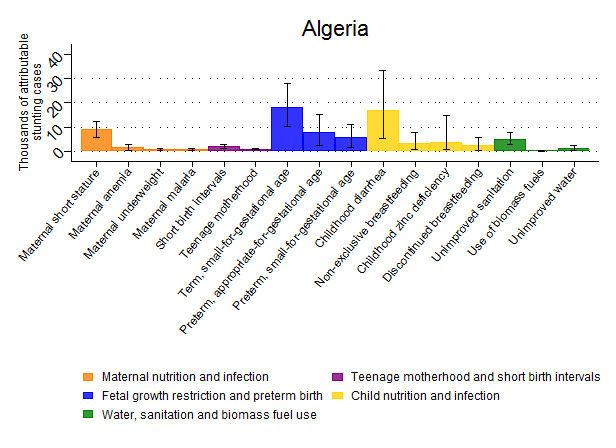


Angola

**Region: Sub-Saharan Africa; Sub-region: Sub-Saharan Africa, Central**

**Stunting prevalence among children age 2: 36.8%**

**Stunting cases among children age 2: 372,406**

**Population of children age 2: 1,010,837**

This country profile provides results for 2011 from the analysis presented in *Risk factors for childhood stunting in 137 developing countries: a comparative risk assessment analysis at global, regional, and country levels.*

**Table 1: Population attributable fraction (PAF in %), attributable stunting prevalence (percentage points) and number of stunting cases among 2-year olds attributable to individual risks and risk factor clusters (95% confidence intervals presented in parentheses)**

| **Description** | **PAF** | **Attributable stunting prevalence** | **Attributable stunting cases (thousands)** |
| --- | --- | --- | --- |
| **Maternal nutrition and infection** | **13.7 (11.6, 15.9)** | **5.1 (3.3, 7.2)** | **51.1 (33.0, 72.5)** |
| Maternal short stature | 9.6 (8.7, 10.4) | 3.6 (2.3, 4.8) | 35.9 (23.6, 48.4) |
| Maternal underweight | 1.5 (0.5, 2.7) | 0.5 (0.2, 1.1) | 5.5 (1.7, 11.1) |
| Maternal malaria | 0.8 (0.3, 1.3) | 0.3 (0.1, 0.5) | 2.8 (1.0, 5.2) |
| Maternal anemia | 2.3 (0.6, 4.1) | 0.9 (0.2, 1.7) | 8.7 (2.3, 17.4) |
| **Teenage motherhood and short birth intervals** | **2.3 (2.2, 2.4)** | **0.9 (0.6, 1.2)** | **8.7 (5.7, 11.7)** |
| Teenage motherhood | 1.4 (1.3, 1.5) | 0.5 (0.3, 0.7) | 5.2 (3.3, 7.0) |
| Short birth intervals | 1.0 (0.9, 1.0) | 0.4 (0.2, 0.5) | 3.6 (2.4, 4.8) |
| **Fetal growth restriction and preterm birth** | **30.7 (26.3, 34.8)** | **11.3 (7.4, 15.5)** | **114.5 (75.1, 156.7)** |
| Preterm, small-for-gestational age | 4.8 (0.8, 9.1) | 1.8 (0.3, 3.7) | 17.8 (3.0, 37.0) |
| Preterm, appropriate-for-gestational age | 6.4 (1.5, 11.4) | 2.4 (0.5, 4.5) | 23.8 (5.2, 45.4) |
| Term, small-for-gestational age | 22.3 (18.4, 26.7) | 8.2 (5.4, 11.6) | 83.0 (54.2, 116.9) |
| Low birth weight | 19.3 (16.2, 22.3) | 7.1 (4.6, 9.9) | 71.7 (46.4, 100.2) |
| **Child nutrition and infection** | **16.2 (6.1, 27.4)** | **6.0 (2.1, 11.2)** | **60.4 (21.4, 112.9)** |
| Childhood zinc deficiency | 1.1 (0.4, 2.3) | 0.4 (0.2, 0.9) | 4.2 (1.5, 9.0) |
| Childhood diarrhea | 15.7 (5.5, 27.0) | 5.8 (1.9, 11.0) | 58.6 (19.6, 111.4) |
| Non-exclusive breastfeeding | 2.2 (-0.3, 5.4) | 0.8 (-0.1, 2.1) | 8.2 (-1.1, 21.3) |
| HIV infection without HAART before 2 years | 0.4 (0.1, 1.0) | 0.1 (0.0, 0.4) | 1.4 (0.5, 3.9) |
| Discontinued breastfeeding | 0.9 (0.0, 2.5) | 0.3 (0.0, 0.9) | 3.2 (0.2, 9.4) |
| **Water, sanitation and biomass fuel use** | **25.1 (22.5, 27.6)** | **9.2 (6.0, 12.7)** | **93.4 (60.2, 128.4)** |
| Unimproved sanitation | 19.4 (17.4, 21.5) | 7.2 (4.6, 9.9) | 72.3 (46.0, 100.3) |
| Unimproved water | 3.6 (2.1, 5.4) | 1.3 (0.7, 2.1) | 13.5 (7.1, 21.7) |
| Use of biomass fuels | 3.5 (1.8, 5.4) | 1.3 (0.6, 2.2) | 13.1 (6.1, 21.8) |

**Fig 1: Stunting cases among 2-year olds attributable to individual risk factors**


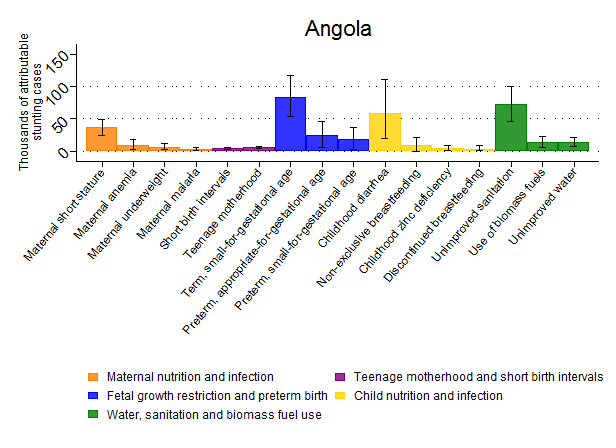


Antigua and Barbuda

**Region: Latin America/Caribbean; Sub-region: Caribbean**

**Stunting prevalence among children age 2: 17.0%**

**Stunting cases among children age 2: 253**

**Population of children age 2: 1,487**

This country profile provides results for 2011 from the analysis presented in *Risk factors for childhood stunting in 137 developing countries: a comparative risk assessment analysis at global, regional, and country levels.*

**Table 1: Population attributable fraction (PAF in %), attributable stunting prevalence (percentage points) and number of stunting cases among 2-year olds attributable to individual risks and risk factor clusters (95% confidence intervals presented in parentheses)**

| **Description** | **PAF** | **Attributable stunting prevalence** | **Attributable stunting cases (thousands)** |
| --- | --- | --- | --- |
| **Maternal nutrition and infection** | **9.6 (8.4, 10.8)** | **1.6 (0.6, 2.7)** | **0.0 (0.0, 0.0)** |
| Maternal short stature | 8.7 (7.6, 9.5) | 1.5 (0.5, 2.5) | 0.0 (0.0, 0.0) |
| Maternal underweight | 0.2 (0.0, 0.4) | 0.0 (0.0, 0.1) | 0.0 (0.0, 0.0) |
| Maternal malaria | 0.0 (0.0, 0.0) | 0.0 (0.0, 0.0) | 0.0 (0.0, 0.0) |
| Maternal anemia | 0.8 (0.2, 1.7) | 0.1 (0.0, 0.3) | 0.0 (0.0, 0.0) |
| **Teenage motherhood and short birth intervals** | **2.3 (2.2, 2.4)** | **0.4 (0.1, 0.6)** | **0.0 (0.0, 0.0)** |
| Teenage motherhood | 1.3 (1.3, 1.4) | 0.2 (0.1, 0.4) | 0.0 (0.0, 0.0) |
| Short birth intervals | 0.9 (0.9, 1.0) | 0.2 (0.1, 0.3) | 0.0 (0.0, 0.0) |
| **Fetal growth restriction and preterm birth** | **15.9 (11.9, 20.0)** | **2.7 (0.9, 4.8)** | **0.0 (0.0, 0.1)** |
| Preterm, small-for-gestational age | 3.9 (1.3, 6.8) | 0.7 (0.2, 1.5) | 0.0 (0.0, 0.0) |
| Preterm, appropriate-for-gestational age | 3.7 (1.2, 6.4) | 0.6 (0.1, 1.3) | 0.0 (0.0, 0.0) |
| Term, small-for-gestational age | 9.1 (5.7, 12.6) | 1.6 (0.5, 2.9) | 0.0 (0.0, 0.0) |
| Low birth weight | 10.0 (8.3, 11.9) | 1.7 (0.6, 2.9) | 0.0 (0.0, 0.0) |
| **Child nutrition and infection** | **16.1 (6.9, 24.9)** | **2.7 (0.8, 5.3)** | **0.0 (0.0, 0.1)** |
| Childhood zinc deficiency | 0.7 (0.2, 2.7) | 0.1 (0.0, 0.5) | 0.0 (0.0, 0.0) |
| Childhood diarrhea | 15.8 (6.5, 24.6) | 2.7 (0.8, 5.2) | 0.0 (0.0, 0.1) |
| Non-exclusive breastfeeding | 2.9 (0.4, 6.5) | 0.5 (0.1, 1.3) | 0.0 (0.0, 0.0) |
| HIV infection without HAART before 2 years | No data | No data | No data |
| Discontinued breastfeeding | 2.5 (0.4, 5.6) | 0.4 (0.0, 1.1) | 0.0 (0.0, 0.0) |
| **Water, sanitation and biomass fuel use** | **3.4 (1.7, 4.9)** | **0.6 (0.2, 1.1)** | **0.0 (0.0, 0.0)** |
| Unimproved sanitation | 3.2 (1.6, 4.7) | 0.5 (0.1, 1.1) | 0.0 (0.0, 0.0) |
| Unimproved water | 0.2 (0.1, 0.3) | 0.0 (0.0, 0.1) | 0.0 (0.0, 0.0) |
| Use of biomass fuels | 0.0 (-0.2, 0.2) | 0.0 (0.0, 0.0) | 0.0 (0.0, 0.0) |

**Fig 1: Stunting cases among 2-year olds attributable to individual risk factors**


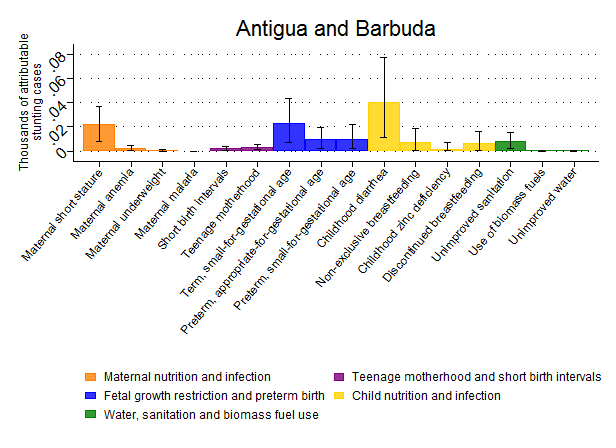


Argentina

**Region: High Income Latin America/Caribbean; Sub-region: Latin America, Southern**

**Stunting prevalence among children age 2: 8.0%**

**Stunting cases among children age 2: 59,456**

**Population of children age 2: 747,797**

This country profile provides results for 2011 from the analysis presented in *Risk factors for childhood stunting in 137 developing countries: a comparative risk assessment analysis at global, regional, and country levels.*

**Table 1: Population attributable fraction (PAF in %), attributable stunting prevalence (percentage points) and number of stunting cases among 2-year olds attributable to individual risks and risk factor clusters (95% confidence intervals presented in parentheses)**

| **Description** | **PAF** | **Attributable stunting prevalence** | **Attributable stunting cases (thousands)** |
| --- | --- | --- | --- |
| **Maternal nutrition and infection** | **6.5 (5.7, 7.5)** | **0.5 (0.2, 0.8)** | **3.8 (1.7, 6.0)** |
| Maternal short stature | 5.5 (5.3, 5.7) | 0.4 (0.2, 0.7) | 3.3 (1.5, 5.1) |
| Maternal underweight | 0.2 (0.0, 0.3) | 0.0 (0.0, 0.0) | 0.1 (0.0, 0.2) |
| Maternal malaria | 0.0 (0.0, 0.0) | 0.0 (0.0, 0.0) | 0.0 (0.0, 0.0) |
| Maternal anemia | 0.9 (0.1, 1.9) | 0.1 (0.0, 0.2) | 0.5 (0.1, 1.2) |
| **Teenage motherhood and short birth intervals** | **2.3 (2.2, 2.4)** | **0.2 (0.1, 0.3)** | **1.4 (0.6, 2.2)** |
| Teenage motherhood | 1.4 (1.3, 1.4) | 0.1 (0.1, 0.2) | 0.8 (0.4, 1.3) |
| Short birth intervals | 1.0 (0.9, 1.0) | 0.1 (0.0, 0.1) | 0.6 (0.3, 0.9) |
| **Fetal growth restriction and preterm birth** | **19.2 (15.9, 22.7)** | **1.5 (0.7, 2.4)** | **11.4 (5.1, 17.6)** |
| Preterm, small-for-gestational age | 4.8 (3.4, 6.8) | 0.4 (0.2, 0.7) | 2.9 (1.2, 4.9) |
| Preterm, appropriate-for-gestational age | 4.7 (3.6, 6.0) | 0.4 (0.2, 0.6) | 2.8 (1.2, 4.7) |
| Term, small-for-gestational age | 10.9 (7.4, 14.7) | 0.9 (0.4, 1.5) | 6.4 (2.8, 11.0) |
| Low birth weight | 12.0 (10.0, 14.1) | 1.0 (0.4, 1.5) | 7.1 (3.2, 11.3) |
| **Child nutrition and infection** | **17.3 (7.2, 28.3)** | **1.4 (0.4, 2.8)** | **10.4 (3.0, 20.7)** |
| Childhood zinc deficiency | 1.1 (0.1, 5.2) | 0.1 (0.0, 0.4) | 0.7 (0.0, 3.2) |
| Childhood diarrhea | 16.9 (6.9, 27.7) | 1.3 (0.4, 2.7) | 10.1 (3.0, 20.2) |
| Non-exclusive breastfeeding | 1.6 (-1.8, 5.1) | 0.1 (-0.1, 0.5) | 0.9 (-1.1, 3.6) |
| Discontinued breastfeeding | 2.1 (0.4, 5.0) | 0.2 (0.0, 0.4) | 1.2 (0.2, 3.3) |
| HIV infection without HAART before 2 years | No data | No data | No data |
| **Water, sanitation and biomass fuel use** | **1.6 (0.3, 2.9)** | **0.1 (0.0, 0.3)** | **0.9 (0.2, 2.0)** |
| Unimproved sanitation | 1.4 (0.2, 2.8) | 0.1 (0.0, 0.3) | 0.9 (0.1, 1.9) |
| Unimproved water | 0.1 (0.0, 0.2) | 0.0 (0.0, 0.0) | 0.1 (0.0, 0.1) |
| Use of biomass fuels | 0.0 (-0.2, 0.3) | 0.0 (0.0, 0.0) | 0.0 (-0.1, 0.2) |

**Fig 1: Stunting cases among 2-year olds attributable to individual risk factors**


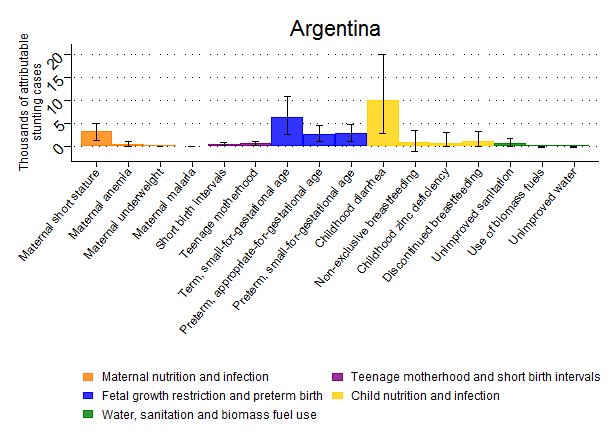


Armenia

**Region: Central Asia Eastern Europe/Central Asia; Sub-region: Asia, Central**

**Stunting prevalence among children age 2: 17.1%**

**Stunting cases among children age 2: 7,040**

**Population of children age 2: 41,097**

This country profile provides results for 2011 from the analysis presented in *Risk factors for childhood stunting in 137 developing countries: a comparative risk assessment analysis at global, regional, and country levels.*

**Table 1: Population attributable fraction (PAF in %), attributable stunting prevalence (percentage points) and number of stunting cases among 2-year olds attributable to individual risks and risk factor clusters (95% confidence intervals presented in parentheses)**

| **Description** | **PAF** | **Attributable stunting prevalence** | **Attributable stunting cases (thousands)** |
| --- | --- | --- | --- |
| **Maternal nutrition and infection** | **7.3 (6.6, 8.1)** | **1.2 (0.9, 1.6)** | **0.5 (0.4, 0.7)** |
| Maternal short stature | 6.1 (5.9, 6.3) | 1.1 (0.8, 1.4) | 0.4 (0.3, 0.6) |
| Maternal underweight | 0.3 (0.1, 0.7) | 0.1 (0.0, 0.1) | 0.0 (0.0, 0.0) |
| Maternal malaria | 0.0 (0.0, 0.0) | 0.0 (0.0, 0.0) | 0.0 (0.0, 0.0) |
| Maternal anemia | 0.9 (0.2, 1.8) | 0.2 (0.0, 0.3) | 0.1 (0.0, 0.1) |
| **Teenage motherhood and short birth intervals** | **1.2 (1.1, 1.3)** | **0.2 (0.1, 0.3)** | **0.1 (0.1, 0.1)** |
| Teenage motherhood | 0.5 (0.4, 0.5) | 0.1 (0.1, 0.1) | 0.0 (0.0, 0.0) |
| Short birth intervals | 0.8 (0.7, 0.9) | 0.1 (0.1, 0.2) | 0.1 (0.0, 0.1) |
| **Fetal growth restriction and preterm birth** | **24.6 (17.6, 30.7)** | **4.2 (2.7, 5.9)** | **1.7 (1.1, 2.4)** |
| Preterm, small-for-gestational age | 6.4 (1.1, 12.6) | 1.1 (0.2, 2.3) | 0.5 (0.1, 0.9) |
| Preterm, appropriate-for-gestational age | 5.7 (0.3, 10.8) | 1.0 (0.0, 2.0) | 0.4 (0.0, 0.8) |
| Term, small-for-gestational age | 14.5 (9.3, 19.8) | 2.5 (1.4, 3.7) | 1.0 (0.6, 1.5) |
| Low birth weight | 12.5 (10.3, 14.7) | 2.1 (1.4, 2.9) | 0.9 (0.6, 1.2) |
| **Child nutrition and infection** | **18.1 (7.8, 28.3)** | **3.1 (1.3, 5.3)** | **1.3 (0.5, 2.2)** |
| Childhood zinc deficiency | 1.9 (0.5, 5.0) | 0.3 (0.1, 0.8) | 0.1 (0.0, 0.3) |
| Childhood diarrhea | 17.3 (7.2, 27.2) | 3.0 (1.2, 5.1) | 1.2 (0.5, 2.1) |
| Non-exclusive breastfeeding | 1.8 (-1.5, 5.1) | 0.3 (-0.3, 0.9) | 0.1 (-0.1, 0.4) |
| Discontinued breastfeeding | 2.6 (0.4, 5.9) | 0.4 (0.1, 1.0) | 0.2 (0.0, 0.4) |
| HIV infection without HAART before 2 years | No data | No data | No data |
| **Water, sanitation and biomass fuel use** | **3.3 (2.1, 4.4)** | **0.6 (0.3, 0.8)** | **0.2 (0.1, 0.3)** |
| Unimproved sanitation | 2.9 (2.0, 3.8) | 0.5 (0.3, 0.7) | 0.2 (0.1, 0.3) |
| Unimproved water | 0.1 (-0.1, 0.3) | 0.0 (0.0, 0.0) | 0.0 (0.0, 0.0) |
| Use of biomass fuels | 0.3 (-0.4, 1.0) | 0.1 (-0.1, 0.2) | 0.0 (0.0, 0.1) |

**Fig 1: Stunting cases among 2-year olds attributable to individual risk factors**


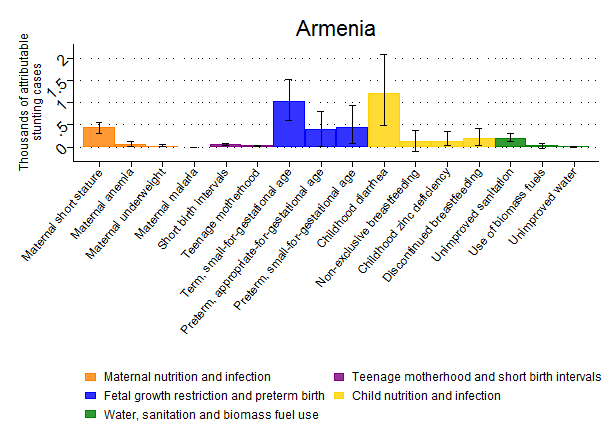


Azerbaijan

**Region: Central Asia Eastern Europe/Central Asia; Sub-region: Asia, Central**

**Stunting prevalence among children age 2: 19.8%**

**Stunting cases among children age 2: 38,132**

**Population of children age 2: 192,940**

This country profile provides results for 2011 from the analysis presented in *Risk factors for childhood stunting in 137 developing countries: a comparative risk assessment analysis at global, regional, and country levels.*

**Table 1: Population attributable fraction (PAF in %), attributable stunting prevalence (percentage points) and number of stunting cases among 2-year olds attributable to individual risks and risk factor clusters (95% confidence intervals presented in parentheses)**

| **Description** | **PAF** | **Attributable stunting prevalence** | **Attributable stunting cases (thousands)** |
| --- | --- | --- | --- |
| **Maternal nutrition and infection** | **6.9 (6.1, 7.8)** | **1.4 (0.8, 1.9)** | **2.6 (1.6, 3.8)** |
| Maternal short stature | 5.7 (5.5, 5.9) | 1.1 (0.7, 1.6) | 2.2 (1.3, 3.1) |
| Maternal underweight | 0.3 (0.1, 0.6) | 0.1 (0.0, 0.1) | 0.1 (0.0, 0.2) |
| Maternal malaria | 0.0 (0.0, 0.0) | 0.0 (0.0, 0.0) | 0.0 (0.0, 0.0) |
| Maternal anemia | 1.0 (0.3, 1.9) | 0.2 (0.0, 0.4) | 0.4 (0.1, 0.8) |
| **Teenage motherhood and short birth intervals** | **1.7 (1.6, 1.9)** | **0.3 (0.2, 0.5)** | **0.7 (0.4, 0.9)** |
| Teenage motherhood | 0.6 (0.5, 0.7) | 0.1 (0.1, 0.2) | 0.2 (0.1, 0.3) |
| Short birth intervals | 1.1 (1.0, 1.3) | 0.2 (0.1, 0.3) | 0.4 (0.3, 0.6) |
| **Fetal growth restriction and preterm birth** | **23.2 (17.3, 28.8)** | **4.6 (2.6, 6.7)** | **8.9 (5.0, 13.0)** |
| Preterm, small-for-gestational age | 5.0 (1.0, 9.8) | 1.0 (0.2, 2.1) | 1.9 (0.4, 4.1) |
| Preterm, appropriate-for-gestational age | 4.6 (0.8, 8.5) | 0.9 (0.2, 1.8) | 1.7 (0.3, 3.5) |
| Term, small-for-gestational age | 15.3 (9.3, 21.4) | 3.0 (1.5, 4.8) | 5.8 (2.9, 9.3) |
| Low birth weight | 11.7 (9.7, 13.8) | 2.3 (1.4, 3.4) | 4.5 (2.6, 6.6) |
| **Child nutrition and infection** | **19.0 (8.0, 30.6)** | **3.8 (1.5, 7.0)** | **7.3 (2.9, 13.4)** |
| Childhood zinc deficiency | 1.7 (0.5, 4.3) | 0.3 (0.1, 0.9) | 0.6 (0.2, 1.7) |
| Childhood diarrhea | 18.4 (7.6, 29.4) | 3.6 (1.4, 6.6) | 7.0 (2.8, 12.7) |
| Non-exclusive breastfeeding | 2.9 (0.4, 6.3) | 0.6 (0.1, 1.3) | 1.1 (0.2, 2.6) |
| HIV infection without HAART before 2 years | No data | No data | No data |
| Discontinued breastfeeding | 2.8 (0.4, 6.1) | 0.6 (0.1, 1.3) | 1.1 (0.2, 2.5) |
| **Water, sanitation and biomass fuel use** | **7.7 (5.2, 10.1)** | **1.5 (0.8, 2.3)** | **2.9 (1.6, 4.5)** |
| Unimproved sanitation | 5.9 (3.7, 8.1) | 1.2 (0.6, 1.9) | 2.3 (1.2, 3.6) |
| Unimproved water | 1.4 (0.7, 2.2) | 0.3 (0.1, 0.5) | 0.5 (0.2, 0.9) |
| Use of biomass fuels | 0.5 (-0.4, 1.3) | 0.1 (-0.1, 0.3) | 0.2 (-0.2, 0.5) |

**Fig 1: Stunting cases among 2-year olds attributable to individual risk factors**


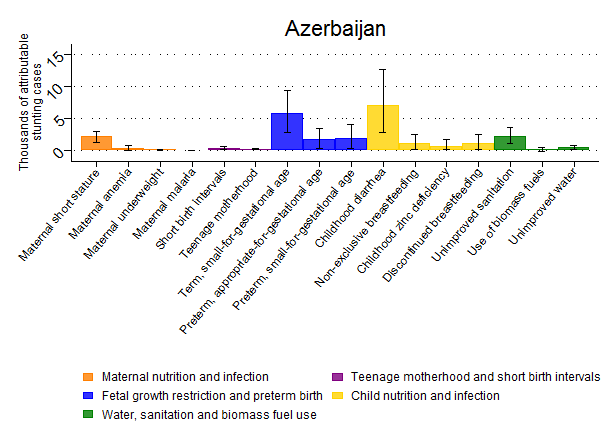


Bahamas

**Region: Latin America/Caribbean; Sub-region: Caribbean**

**Stunting prevalence among children age 2: 12.0%**

**Stunting cases among children age 2: 675**

**Population of children age 2: 5,603**

This country profile provides results for 2011 from the analysis presented in *Risk factors for childhood stunting in 137 developing countries: a comparative risk assessment analysis at global, regional, and country levels.*

**Table 1: Population attributable fraction (PAF in %), attributable stunting prevalence (percentage points) and number of stunting cases among 2-year olds attributable to individual risks and risk factor clusters (95% confidence intervals presented in parentheses)**

| **Description** | **PAF** | **Attributable stunting prevalence** | **Attributable stunting cases (thousands)** |
| --- | --- | --- | --- |
| **Maternal nutrition and infection** | **10.2 (8.7, 11.9)** | **1.2 (0.4, 2.2)** | **0.1 (0.0, 0.1)** |
| Maternal short stature | 8.6 (7.5, 9.4) | 1.0 (0.3, 1.8) | 0.1 (0.0, 0.1) |
| Maternal underweight | 0.4 (0.0, 0.8) | 0.0 (0.0, 0.1) | 0.0 (0.0, 0.0) |
| Maternal malaria | 0.0 (0.0, 0.0) | 0.0 (0.0, 0.0) | 0.0 (0.0, 0.0) |
| Maternal anemia | 1.4 (0.3, 3.0) | 0.2 (0.0, 0.4) | 0.0 (0.0, 0.0) |
| **Teenage motherhood and short birth intervals** | **2.3 (2.2, 2.4)** | **0.3 (0.1, 0.5)** | **0.0 (0.0, 0.0)** |
| Teenage motherhood | 1.3 (1.3, 1.4) | 0.2 (0.0, 0.3) | 0.0 (0.0, 0.0) |
| Short birth intervals | 0.9 (0.9, 1.0) | 0.1 (0.0, 0.2) | 0.0 (0.0, 0.0) |
| **Fetal growth restriction and preterm birth** | **24.1 (19.6, 28.5)** | **2.9 (0.9, 5.1)** | **0.2 (0.0, 0.3)** |
| Preterm, small-for-gestational age | 5.3 (2.4, 8.7) | 0.6 (0.2, 1.3) | 0.0 (0.0, 0.1) |
| Preterm, appropriate-for-gestational age | 5.2 (2.2, 8.6) | 0.6 (0.1, 1.3) | 0.0 (0.0, 0.1) |
| Term, small-for-gestational age | 15.4 (11.2, 20.1) | 1.9 (0.6, 3.4) | 0.1 (0.0, 0.2) |
| Low birth weight | 18.2 (15.3, 21.2) | 2.2 (0.7, 3.8) | 0.1 (0.0, 0.2) |
| **Child nutrition and infection** | **7.7 (3.2, 12.9)** | **0.9 (0.2, 1.9)** | **0.1 (0.0, 0.1)** |
| Childhood zinc deficiency | 1.0 (0.2, 4.5) | 0.1 (0.0, 0.5) | 0.0 (0.0, 0.0) |
| Childhood diarrhea | 7.2 (2.9, 12.2) | 0.9 (0.2, 1.9) | 0.0 (0.0, 0.1) |
| Non-exclusive breastfeeding | 1.4 (0.2, 3.1) | 0.2 (0.0, 0.4) | 0.0 (0.0, 0.0) |
| HIV infection without HAART before 2 years | No data | No data | No data |
| Discontinued breastfeeding | 1.2 (0.2, 2.8) | 0.1 (0.0, 0.4) | 0.0 (0.0, 0.0) |
| **Water, sanitation and biomass fuel use** | **3.1 (2.0, 4.4)** | **0.4 (0.1, 0.7)** | **0.0 (0.0, 0.0)** |
| Unimproved sanitation | 2.9 (1.8, 4.2) | 0.3 (0.1, 0.7) | 0.0 (0.0, 0.0) |
| Unimproved water | 0.3 (0.1, 0.4) | 0.0 (0.0, 0.1) | 0.0 (0.0, 0.0) |
| Use of biomass fuels | 0.0 (0.0, 0.0) | 0.0 (0.0, 0.0) | 0.0 (0.0, 0.0) |

**Fig 1: Stunting cases among 2-year olds attributable to individual risk factors**


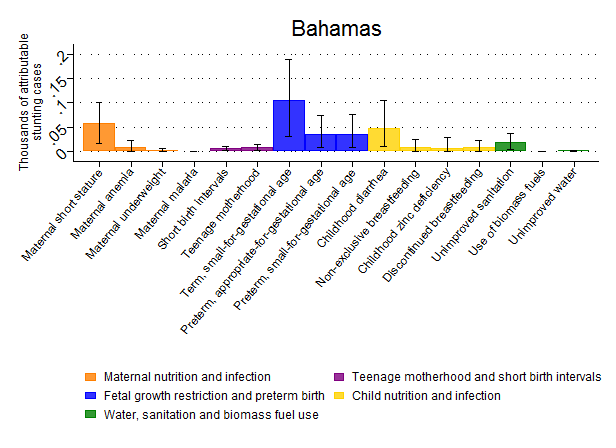


Bahrain

**Region: North Africa/Middle East; Sub-region: North Africa / Middle East**

**Stunting prevalence among children age 2: 10.8%**

**Stunting cases among children age 2: 2,066**

**Population of children age 2: 19,163**

This country profile provides results for 2011 from the analysis presented in *Risk factors for childhood stunting in 137 developing countries: a comparative risk assessment analysis at global, regional, and country levels.*

**Table 1: Population attributable fraction (PAF in %), attributable stunting prevalence (percentage points) and number of stunting cases among 2-year olds attributable to individual risks and risk factor clusters (95% confidence intervals presented in parentheses)**

| **Description** | **PAF** | **Attributable stunting prevalence** | **Attributable stunting cases (thousands)** |
| --- | --- | --- | --- |
| **Maternal nutrition and infection** | **9.4 (8.2, 10.7)** | **1.0 (0.4, 1.6)** | **0.2 (0.1, 0.3)** |
| Maternal short stature | 7.7 (7.3, 8.0) | 0.8 (0.3, 1.3) | 0.2 (0.1, 0.3) |
| Maternal underweight | 0.4 (0.1, 0.8) | 0.0 (0.0, 0.1) | 0.0 (0.0, 0.0) |
| Maternal malaria | 0.0 (0.0, 0.0) | 0.0 (0.0, 0.0) | 0.0 (0.0, 0.0) |
| Maternal anemia | 1.4 (0.3, 2.8) | 0.2 (0.0, 0.4) | 0.0 (0.0, 0.1) |
| **Teenage motherhood and short birth intervals** | **1.8 (1.7, 1.9)** | **0.2 (0.1, 0.3)** | **0.0 (0.0, 0.1)** |
| Teenage motherhood | 0.5 (0.5, 0.6) | 0.1 (0.0, 0.1) | 0.0 (0.0, 0.0) |
| Short birth intervals | 1.2 (1.1, 1.3) | 0.1 (0.1, 0.2) | 0.0 (0.0, 0.0) |
| **Fetal growth restriction and preterm birth** | **28.1 (23.3, 32.4)** | **3.0 (1.3, 4.9)** | **0.6 (0.2, 0.9)** |
| Preterm, small-for-gestational age | 7.7 (4.4, 11.4) | 0.8 (0.3, 1.5) | 0.2 (0.1, 0.3) |
| Preterm, appropriate-for-gestational age | 7.0 (4.2, 9.9) | 0.7 (0.3, 1.3) | 0.1 (0.0, 0.3) |
| Term, small-for-gestational age | 16.3 (11.2, 21.4) | 1.8 (0.7, 3.0) | 0.3 (0.1, 0.6) |
| Low birth weight | 16.0 (13.4, 18.7) | 1.7 (0.7, 2.8) | 0.3 (0.1, 0.5) |
| **Child nutrition and infection** | **10.0 (3.6, 23.2)** | **1.1 (0.3, 2.7)** | **0.2 (0.1, 0.5)** |
| Childhood zinc deficiency | 6.0 (1.0, 29.5) | 0.6 (0.1, 3.0) | 0.1 (0.0, 0.6) |
| Childhood diarrhea | 7.2 (2.9, 12.3) | 0.8 (0.2, 1.6) | 0.1 (0.0, 0.3) |
| Non-exclusive breastfeeding | 1.2 (0.0, 2.9) | 0.1 (0.0, 0.3) | 0.0 (0.0, 0.1) |
| HIV infection without HAART before 2 years | No data | No data | No data |
| Discontinued breastfeeding | 0.9 (0.1, 2.3) | 0.1 (0.0, 0.3) | 0.0 (0.0, 0.1) |
| **Water, sanitation and biomass fuel use** | **0.5 (0.2, 0.7)** | **0.1 (0.0, 0.1)** | **0.0 (0.0, 0.0)** |
| Unimproved sanitation | 0.4 (0.2, 0.6) | 0.0 (0.0, 0.1) | 0.0 (0.0, 0.0) |
| Unimproved water | 0.1 (-0.1, 0.3) | 0.0 (0.0, 0.0) | 0.0 (0.0, 0.0) |
| Use of biomass fuels | 0.0 (0.0, 0.0) | 0.0 (0.0, 0.0) | 0.0 (0.0, 0.0) |

**Fig 1: Stunting cases among 2-year olds attributable to individual risk factors**


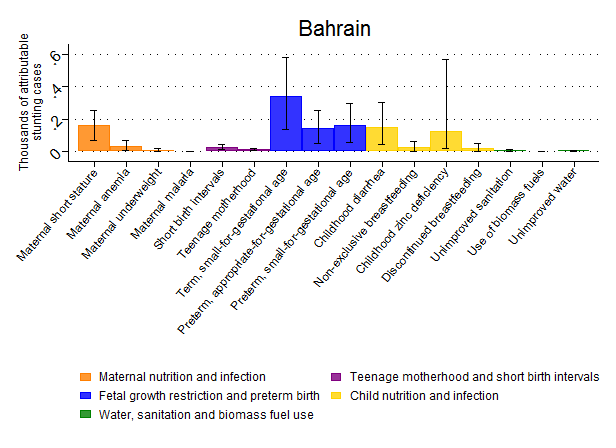


Bangladesh

**Region: South Asia; Sub-region: Asia, South**

**Stunting prevalence among children age 2: 43.8%**

**Stunting cases among children age 2: 1,422,758**

**Population of children age 2: 3,248,246**

This country profile provides results for 2011 from the analysis presented in *Risk factors for childhood stunting in 137 developing countries: a comparative risk assessment analysis at global, regional, and country levels.*

**Table 1: Population attributable fraction (PAF in %), attributable stunting prevalence (percentage points) and number of stunting cases among 2-year olds attributable to individual risks and risk factor clusters (95% confidence intervals presented in parentheses)**

| **Description** | **PAF** | **Attributable stunting prevalence** | **Attributable stunting cases (thousands)** |
| --- | --- | --- | --- |
| **Maternal nutrition and infection** | **19.8 (17.0, 22.5)** | **8.7 (6.5, 10.9)** | **281.4 (209.6, 353.5)** |
| Maternal short stature | 13.1 (12.9, 13.3) | 5.7 (4.4, 7.0) | 186.6 (144.5, 227.3) |
| Maternal underweight | 4.2 (2.5, 6.1) | 1.8 (1.0, 2.8) | 60.0 (32.9, 92.1) |
| Maternal malaria | 0.0 (0.0, 0.0) | 0.0 (0.0, 0.0) | 0.0 (0.0, 0.0) |
| Maternal anemia | 3.6 (1.1, 6.1) | 1.6 (0.4, 2.8) | 51.2 (14.4, 92.5) |
| **Teenage motherhood and short birth intervals** | **2.6 (2.5, 2.7)** | **1.1 (0.9, 1.4)** | **36.9 (28.8, 45.2)** |
| Teenage motherhood | 2.1 (2.0, 2.3) | 0.9 (0.7, 1.2) | 30.4 (23.7, 37.5) |
| Short birth intervals | 0.5 (0.4, 0.5) | 0.2 (0.2, 0.2) | 6.6 (5.0, 8.1) |
| **Fetal growth restriction and preterm birth** | **38.3 (34.9, 41.8)** | **16.8 (13.1, 20.8)** | **545.4 (426.5, 676)** |
| Preterm, small-for-gestational age | 6.4 (3.9, 9.7) | 2.8 (1.6, 4.5) | 91.3 (51.3, 146) |
| Preterm, appropriate-for-gestational age | 5.8 (3.8, 8.3) | 2.5 (1.6, 3.8) | 82.3 (50.4, 122.9) |
| Term, small-for-gestational age | 30.0 (25.7, 34.4) | 13.2 (10.0, 16.7) | 427.4 (325, 543.5) |
| Low birth weight | 29.3 (25.2, 33.4) | 12.9 (9.8, 16.3) | 417.5 (317.2, 528) |
| **Child nutrition and infection** | **11.4 (4.7, 19.9)** | **5.0 (2.0, 8.9)** | **161.8 (65.2, 289)** |
| Childhood zinc deficiency | 1.1 (0.4, 1.8) | 0.5 (0.2, 0.8) | 15.0 (5.8, 26.7) |
| Childhood diarrhea | 10.9 (4.2, 19.4) | 4.8 (1.8, 8.7) | 155.1 (58.9, 282.1) |
| Non-exclusive breastfeeding | 1.1 (-1.0, 3.3) | 0.5 (-0.5, 1.6) | 15.3 (-14.9, 51.0) |
| Discontinued breastfeeding | 0.4 (0.0, 1.3) | 0.2 (0.0, 0.5) | 6.0 (0.5, 17.3) |
| HIV infection without HAART before 2 years | No data | No data | No data |
| **Water, sanitation and biomass fuel use** | **21.4 (19.0, 23.9)** | **9.4 (7.1, 11.7)** | **304.5 (229.6, 380)** |
| Unimproved sanitation | 13.7 (11.7, 15.7) | 6.0 (4.5, 7.6) | 194.7 (146.1, 247.3) |
| Unimproved water | 1.4 (0.8, 2.0) | 0.6 (0.3, 0.9) | 19.5 (11.2, 30.4) |
| Use of biomass fuels | 7.7 (5.6, 9.9) | 3.4 (2.3, 4.6) | 109.3 (73.3, 150.4) |

**Fig 1: Stunting cases among 2-year olds attributable to individual risk factors**


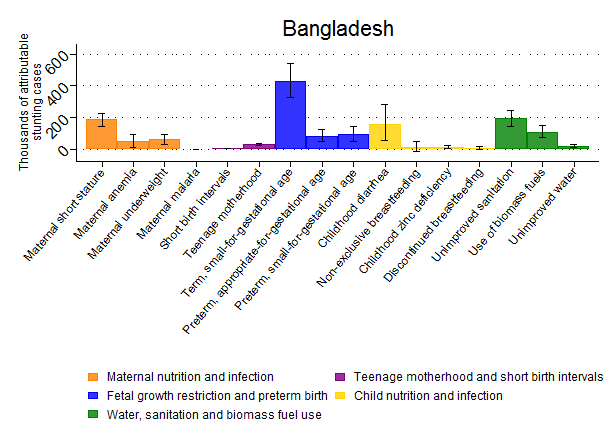


Barbados

**Region: Latin America/Caribbean; Sub-region: Caribbean**

**Stunting prevalence among children age 2: 16.9%**

**Stunting cases among children age 2: 590**

**Population of children age 2: 3,488**

This country profile provides results for 2011 from the analysis presented in *Risk factors for childhood stunting in 137 developing countries: a comparative risk assessment analysis at global, regional, and country levels.*

**Table 1: Population attributable fraction (PAF in %), attributable stunting prevalence (percentage points) and number of stunting cases among 2-year olds attributable to individual risks and risk factor clusters (95% confidence intervals presented in parentheses)**

| **Description** | **PAF** | **Attributable stunting prevalence** | **Attributable stunting cases (thousands)** |
| --- | --- | --- | --- |
| **Maternal nutrition and infection** | **4.5 (3.3, 6.1)** | **0.8 (0.2, 1.4)** | **0.0 (0.0, 0.0)** |
| Maternal short stature | 2.7 (2.5, 2.9) | 0.5 (0.2, 0.8) | 0.0 (0.0, 0.0) |
| Maternal underweight | 0.4 (0.1, 0.8) | 0.1 (0.0, 0.2) | 0.0 (0.0, 0.0) |
| Maternal malaria | 0.0 (0.0, 0.0) | 0.0 (0.0, 0.0) | 0.0 (0.0, 0.0) |
| Maternal anemia | 1.4 (0.2, 3.1) | 0.2 (0.0, 0.6) | 0.0 (0.0, 0.0) |
| **Teenage motherhood and short birth intervals** | **2.3 (2.2, 2.4)** | **0.4 (0.1, 0.6)** | **0.0 (0.0, 0.0)** |
| Teenage motherhood | 1.3 (1.3, 1.4) | 0.2 (0.1, 0.4) | 0.0 (0.0, 0.0) |
| Short birth intervals | 0.9 (0.9, 1.0) | 0.2 (0.1, 0.3) | 0.0 (0.0, 0.0) |
| **Fetal growth restriction and preterm birth** | **24.7 (20.1, 29.1)** | **4.2 (1.5, 7.2)** | **0.1 (0.1, 0.3)** |
| Preterm, small-for-gestational age | 5.4 (2.5, 8.8) | 0.9 (0.3, 1.9) | 0.0 (0.0, 0.1) |
| Preterm, appropriate-for-gestational age | 5.4 (2.9, 8.4) | 0.9 (0.3, 1.8) | 0.0 (0.0, 0.1) |
| Term, small-for-gestational age | 15.8 (11.5, 20.2) | 2.7 (0.9, 4.7) | 0.1 (0.0, 0.2) |
| Low birth weight | 18.4 (15.4, 21.4) | 3.1 (1.1, 5.5) | 0.1 (0.0, 0.2) |
| **Child nutrition and infection** | **7.6 (3.1, 12.9)** | **1.3 (0.3, 2.8)** | **0.0 (0.0, 0.1)** |
| Childhood zinc deficiency | 1.0 (0.2, 3.3) | 0.2 (0.0, 0.6) | 0.0 (0.0, 0.0) |
| Childhood diarrhea | 7.2 (2.7, 12.1) | 1.2 (0.3, 2.6) | 0.0 (0.0, 0.1) |
| Non-exclusive breastfeeding | 1.3 (0.1, 3.2) | 0.2 (0.0, 0.6) | 0.0 (0.0, 0.0) |
| Discontinued breastfeeding | 1.2 (0.2, 2.7) | 0.2 (0.0, 0.5) | 0.0 (0.0, 0.0) |
| HIV infection without HAART before 2 years | No data | No data | No data |
| **Water, sanitation and biomass fuel use** | **2.9 (1.2, 4.6)** | **0.5 (0.1, 1.1)** | **0.0 (0.0, 0.0)** |
| Unimproved sanitation | 2.9 (1.2, 4.5) | 0.5 (0.1, 1.0) | 0.0 (0.0, 0.0) |
| Unimproved water | 0.1 (-0.1, 0.3) | 0.0 (0.0, 0.1) | 0.0 (0.0, 0.0) |
| Use of biomass fuels | 0.0 (-0.5, 0.5) | 0.0 (-0.1, 0.1) | 0.0 (0.0, 0.0) |

**Fig 1: Stunting cases among 2-year olds attributable to individual risk factors**


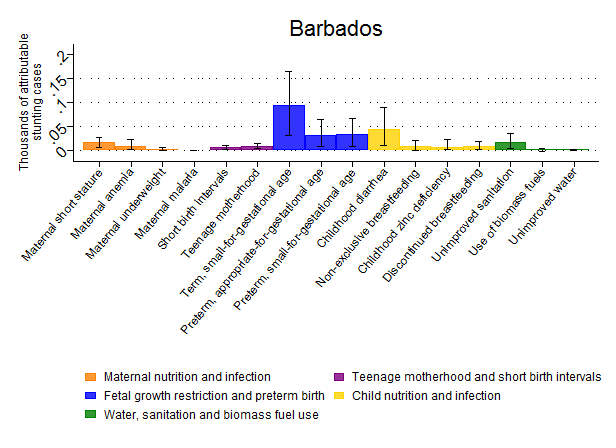


Belize

**Region: Latin America/Caribbean; Sub-region: Caribbean**

**Stunting prevalence among children age 2: 19.7%**

**Stunting cases among children age 2: 1,514**

**Population of children age 2: 7,664**

This country profile provides results for 2011 from the analysis presented in *Risk factors for childhood stunting in 137 developing countries: a comparative risk assessment analysis at global, regional, and country levels.*

**Table 1: Population attributable fraction (PAF in %), attributable stunting prevalence (percentage points) and number of stunting cases among 2-year olds attributable to individual risks and risk factor clusters (95% confidence intervals presented in parentheses)**

| **Description** | **PAF** | **Attributable stunting prevalence** | **Attributable stunting cases (thousands)** |
| --- | --- | --- | --- |
| **Maternal nutrition and infection** | **10.5 (9.1, 12.1)** | **2.1 (1.3, 2.9)** | **0.2 (0.1, 0.2)** |
| Maternal short stature | 8.5 (8.2, 8.9) | 1.7 (1.1, 2.3) | 0.1 (0.1, 0.2) |
| Maternal underweight | 0.6 (0.1, 1.2) | 0.1 (0.0, 0.3) | 0.0 (0.0, 0.0) |
| Maternal malaria | 0.0 (0.0, 0.0) | 0.0 (0.0, 0.0) | 0.0 (0.0, 0.0) |
| Maternal anemia | 1.6 (0.3, 3.3) | 0.3 (0.1, 0.7) | 0.0 (0.0, 0.1) |
| **Teenage motherhood and short birth intervals** | **2.3 (2.2, 2.4)** | **0.4 (0.3, 0.6)** | **0.0 (0.0, 0.0)** |
| Teenage motherhood | 1.3 (1.3, 1.4) | 0.3 (0.2, 0.4) | 0.0 (0.0, 0.0) |
| Short birth intervals | 0.9 (0.9, 1.0) | 0.2 (0.1, 0.3) | 0.0 (0.0, 0.0) |
| **Fetal growth restriction and preterm birth** | **26.8 (22.0, 30.9)** | **5.3 (3.3, 7.5)** | **0.4 (0.3, 0.6)** |
| Preterm, small-for-gestational age | 5.5 (2.1, 9.4) | 1.1 (0.4, 2.0) | 0.1 (0.0, 0.2) |
| Preterm, appropriate-for-gestational age | 5.4 (2.4, 8.8) | 1.1 (0.4, 1.9) | 0.1 (0.0, 0.1) |
| Term, small-for-gestational age | 18.1 (12.8, 23.1) | 3.6 (2.1, 5.3) | 0.3 (0.2, 0.4) |
| Low birth weight | 21.7 (18.3, 25.0) | 4.3 (2.7, 6.0) | 0.3 (0.2, 0.5) |
| **Child nutrition and infection** | **18.0 (7.7, 28.6)** | **3.5 (1.4, 6.3)** | **0.3 (0.1, 0.5)** |
| Childhood zinc deficiency | 1.0 (0.3, 2.1) | 0.2 (0.1, 0.4) | 0.0 (0.0, 0.0) |
| Childhood diarrhea | 17.6 (7.1, 28.2) | 3.5 (1.4, 6.2) | 0.3 (0.1, 0.5) |
| Non-exclusive breastfeeding | 3.5 (1.0, 7.1) | 0.7 (0.2, 1.5) | 0.1 (0.0, 0.1) |
| HIV infection without HAART before 2 years | No data | No data | No data |
| Discontinued breastfeeding | 2.6 (0.3, 5.7) | 0.5 (0.1, 1.2) | 0.0 (0.0, 0.1) |
| **Water, sanitation and biomass fuel use** | **5.7 (4.6, 7.0)** | **1.1 (0.7, 1.6)** | **0.1 (0.1, 0.1)** |
| Unimproved sanitation | 4.2 (3.2, 5.3) | 0.8 (0.5, 1.2) | 0.1 (0.0, 0.1) |
| Unimproved water | 0.4 (0.1, 0.8) | 0.1 (0.0, 0.2) | 0.0 (0.0, 0.0) |
| Use of biomass fuels | 1.1 (0.5, 1.9) | 0.2 (0.1, 0.4) | 0.0 (0.0, 0.0) |

**Fig 1: Stunting cases among 2-year olds attributable to individual risk factors**


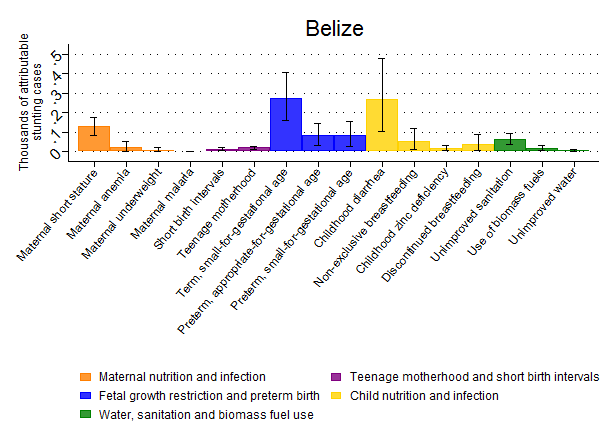


Benin

**Region: Sub-Saharan Africa; Sub-region: Sub-Saharan Africa, West**

**Stunting prevalence among children age 2: 42.9%**

**Stunting cases among children age 2: 154,346**

**Population of children age 2: 359,908**

This country profile provides results for 2011 from the analysis presented in *Risk factors for childhood stunting in 137 developing countries: a comparative risk assessment analysis at global, regional, and country levels.*

**Table 1: Population attributable fraction (PAF in %), attributable stunting prevalence (percentage points) and number of stunting cases among 2-year olds attributable to individual risks and risk factor clusters (95% confidence intervals presented in parentheses)**

| **Description** | **PAF** | **Attributable stunting prevalence** | **Attributable stunting cases (thousands)** |
| --- | --- | --- | --- |
| **Maternal nutrition and infection** | **13.9 (11.0, 17.0)** | **6.0 (4.2, 8.2)** | **21.5 (15.0, 29.6)** |
| Maternal short stature | 6.1 (6.0, 6.3) | 2.6 (2.0, 3.3) | 9.5 (7.2, 11.8) |
| Maternal underweight | 1.4 (0.6, 2.4) | 0.6 (0.3, 1.1) | 2.2 (0.9, 3.9) |
| Maternal malaria | 3.6 (1.5, 5.8) | 1.6 (0.6, 2.7) | 5.6 (2.3, 9.6) |
| Maternal anemia | 3.5 (1.0, 5.7) | 1.5 (0.4, 2.6) | 5.4 (1.6, 9.3) |
| **Teenage motherhood and short birth intervals** | **1.4 (1.4, 1.5)** | **0.6 (0.5, 0.8)** | **2.2 (1.7, 2.9)** |
| Teenage motherhood | 0.8 (0.7, 0.8) | 0.3 (0.2, 0.4) | 1.2 (0.9, 1.5) |
| Short birth intervals | 0.7 (0.6, 0.8) | 0.3 (0.2, 0.4) | 1.1 (0.8, 1.4) |
| **Fetal growth restriction and preterm birth** | **30.4 (25.9, 34.4)** | **13.0 (9.7, 17.2)** | **46.9 (35.0, 61.8)** |
| Preterm, small-for-gestational age | 4.1 (1.0, 7.6) | 1.8 (0.4, 3.4) | 6.3 (1.5, 12.1) |
| Preterm, appropriate-for-gestational age | 5.6 (1.5, 9.9) | 2.4 (0.6, 4.5) | 8.7 (2.3, 16.2) |
| Term, small-for-gestational age | 23.1 (19.0, 27.1) | 9.9 (7.2, 13.1) | 35.6 (25.8, 47.2) |
| Low birth weight | 22.3 (18.8, 25.7) | 9.6 (6.8, 12.6) | 34.4 (24.4, 45.3) |
| **Child nutrition and infection** | **13.8 (5.3, 24.7)** | **5.9 (2.2, 10.8)** | **21.3 (7.9, 38.7)** |
| Childhood zinc deficiency | 0.7 (0.3, 1.2) | 0.3 (0.1, 0.6) | 1.1 (0.4, 2.0) |
| Childhood diarrhea | 13.5 (5.1, 24.6) | 5.8 (2.1, 10.6) | 20.8 (7.6, 38.1) |
| Non-exclusive breastfeeding | 1.3 (-1.7, 4.2) | 0.6 (-0.7, 1.9) | 2.0 (-2.6, 6.8) |
| Discontinued breastfeeding | 0.5 (0.0, 1.5) | 0.2 (0.0, 0.7) | 0.8 (0.0, 2.4) |
| HIV infection without HAART before 2 years | 0.1 (0.0, 0.2) | 0.0 (0.0, 0.1) | 0.1 (0.1, 0.3) |
| **Water, sanitation and biomass fuel use** | **29.8 (27.6, 32.1)** | **12.8 (9.6, 16.4)** | **46.0 (34.6, 58.9)** |
| Unimproved sanitation | 23.7 (21.8, 25.6) | 10.2 (7.6, 13.1) | 36.6 (27.5, 47.2) |
| Unimproved water | 2.0 (1.2, 3.2) | 0.9 (0.5, 1.4) | 3.2 (1.6, 5.0) |
| Use of biomass fuels | 6.1 (4.3, 7.8) | 2.6 (1.7, 3.7) | 9.4 (6.1, 13.3) |

**Fig 1: Stunting cases among 2-year olds attributable to individual risk factors**


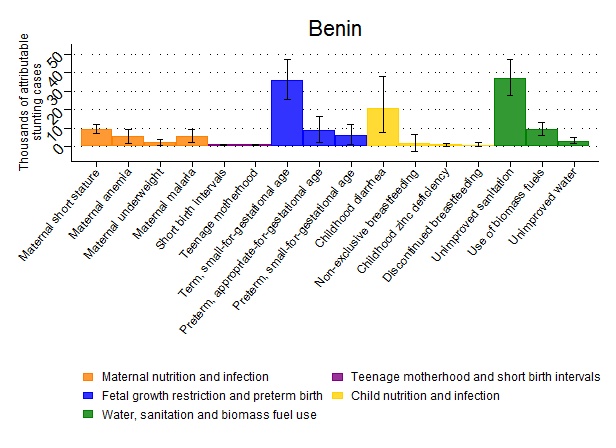


Bhutan

**Region: South Asia; Sub-region: Asia, South**

**Stunting prevalence among children age 2: 36.7%**

**Stunting cases among children age 2: 5,253**

**Population of children age 2: 14,315**

This country profile provides results for 2011 from the analysis presented in *Risk factors for childhood stunting in 137 developing countries: a comparative risk assessment analysis at global, regional, and country levels.*

**Table 1: Population attributable fraction (PAF in %), attributable stunting prevalence (percentage points) and number of stunting cases among 2-year olds attributable to individual risks and risk factor clusters (95% confidence intervals presented in parentheses)**

| **Description** | **PAF** | **Attributable stunting prevalence** | **Attributable stunting cases (thousands)** |
| --- | --- | --- | --- |
| **Maternal nutrition and infection** | **13.2 (11.8, 14.8)** | **4.8 (3.9, 5.8)** | **0.7 (0.6, 0.8)** |
| Maternal short stature | 10.5 (10.2, 10.8) | 3.9 (3.2, 4.5) | 0.6 (0.5, 0.6) |
| Maternal underweight | 1.2 (0.5, 2.0) | 0.4 (0.2, 0.8) | 0.1 (0.0, 0.1) |
| Maternal malaria | 0.0 (0.0, 0.0) | 0.0 (0.0, 0.0) | 0.0 (0.0, 0.0) |
| Maternal anemia | 1.8 (0.5, 3.3) | 0.7 (0.2, 1.3) | 0.1 (0.0, 0.2) |
| **Teenage motherhood and short birth intervals** | **2.2 (2.1, 2.3)** | **0.8 (0.7, 0.9)** | **0.1 (0.1, 0.1)** |
| Teenage motherhood | 1.3 (1.2, 1.3) | 0.5 (0.4, 0.5) | 0.1 (0.1, 0.1) |
| Short birth intervals | 1.0 (0.9, 1.0) | 0.4 (0.3, 0.4) | 0.1 (0.0, 0.1) |
| **Fetal growth restriction and preterm birth** | **27.9 (21.7, 33.1)** | **10.2 (7.6, 12.9)** | **1.5 (1.1, 1.8)** |
| Preterm, small-for-gestational age | 5.4 (0.6, 10.7) | 2.0 (0.2, 4.0) | 0.3 (0.0, 0.6) |
| Preterm, appropriate-for-gestational age | 5.2 (0.6, 10.1) | 1.9 (0.2, 3.8) | 0.3 (0.0, 0.5) |
| Term, small-for-gestational age | 19.5 (13.9, 24.8) | 7.1 (5.0, 9.7) | 1.0 (0.7, 1.4) |
| Low birth weight | 15.2 (12.7, 17.7) | 5.6 (4.4, 6.9) | 0.8 (0.6, 1.0) |
| **Child nutrition and infection** | **11.3 (4.4, 19.5)** | **4.1 (1.6, 7.2)** | **0.6 (0.2, 1.0)** |
| Childhood zinc deficiency | 1.2 (0.5, 2.0) | 0.4 (0.2, 0.8) | 0.1 (0.0, 0.1) |
| Childhood diarrhea | 10.7 (3.9, 18.6) | 3.9 (1.4, 7.0) | 0.6 (0.2, 1.0) |
| Non-exclusive breastfeeding | 1.2 (-0.6, 3.4) | 0.4 (-0.2, 1.2) | 0.1 (0.0, 0.2) |
| HIV infection without HAART before 2 years | No data | No data | No data |
| Discontinued breastfeeding | 1.1 (0.1, 2.7) | 0.4 (0.0, 1.0) | 0.1 (0.0, 0.1) |
| **Water, sanitation and biomass fuel use** | **17.6 (15.2, 20.2)** | **6.5 (5.1, 7.9)** | **0.9 (0.7, 1.1)** |
| Unimproved sanitation | 15.6 (13.4, 17.9) | 5.7 (4.5, 7.1) | 0.8 (0.6, 1.0) |
| Unimproved water | 0.4 (0.1, 0.8) | 0.2 (0.0, 0.3) | 0.0 (0.0, 0.0) |
| Use of biomass fuels | 1.9 (1.0, 3.0) | 0.7 (0.4, 1.1) | 0.1 (0.1, 0.2) |

**Fig 1: Stunting cases among 2-year olds attributable to individual risk factors**


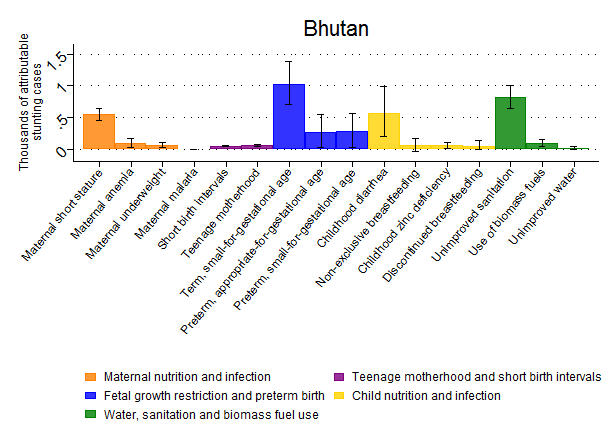


Bolivia

**Region: Latin America/Caribbean; Sub-region: Latin America, Andean**

**Stunting prevalence among children age 2: 32.0%**

**Stunting cases among children age 2: 80,985**

**Population of children age 2: 253,439**

This country profile provides results for 2011 from the analysis presented in *Risk factors for childhood stunting in 137 developing countries: a comparative risk assessment analysis at global, regional, and country levels.*

**Table 1: Population attributable fraction (PAF in %), attributable stunting prevalence (percentage points) and number of stunting cases among 2-year olds attributable to individual risks and risk factor clusters (95% confidence intervals presented in parentheses)**

| **Description** | **PAF** | **Attributable stunting prevalence** | **Attributable stunting cases (thousands)** |
| --- | --- | --- | --- |
| **Maternal nutrition and infection** | **12.4 (11.8, 13.1)** | **4.0 (3.1, 4.8)** | **10.1 (7.9, 12.1)** |
| Maternal short stature | 11.6 (11.3, 11.8) | 3.7 (2.9, 4.4) | 9.4 (7.4, 11.2) |
| Maternal underweight | 0.1 (0.0, 0.3) | 0.0 (0.0, 0.1) | 0.1 (0.0, 0.2) |
| Maternal malaria | 0.0 (0.0, 0.0) | 0.0 (0.0, 0.0) | 0.0 (0.0, 0.0) |
| Maternal anemia | 0.8 (0.2, 1.6) | 0.3 (0.1, 0.5) | 0.7 (0.2, 1.2) |
| **Teenage motherhood and short birth intervals** | **2.0 (1.9, 2.2)** | **0.7 (0.5, 0.8)** | **1.7 (1.3, 2.0)** |
| Teenage motherhood | 1.1 (1.1, 1.2) | 0.4 (0.3, 0.4) | 0.9 (0.7, 1.1) |
| Short birth intervals | 0.9 (0.8, 1.0) | 0.3 (0.2, 0.3) | 0.7 (0.6, 0.9) |
| **Fetal growth restriction and preterm birth** | **17.9 (13.9, 21.5)** | **5.7 (4.3, 7.5)** | **14.5 (10.9, 18.9)** |
| Preterm, small-for-gestational age | 4.9 (2.7, 7.6) | 1.6 (0.8, 2.5) | 3.9 (2.1, 6.3) |
| Preterm, appropriate-for-gestational age | 4.8 (3.0, 6.9) | 1.5 (0.9, 2.3) | 3.9 (2.3, 5.9) |
| Term, small-for-gestational age | 9.4 (5.8, 12.7) | 3.0 (1.8, 4.3) | 7.6 (4.4, 10.8) |
| Low birth weight | 8.6 (7.1, 10.2) | 2.8 (2.0, 3.5) | 7.0 (5.1, 9.0) |
| **Child nutrition and infection** | **19.5 (8.2, 30.3)** | **6.2 (2.5, 10.1)** | **15.8 (6.3, 25.5)** |
| Childhood zinc deficiency | 0.5 (0.2, 0.9) | 0.2 (0.1, 0.3) | 0.4 (0.2, 0.8) |
| Childhood diarrhea | 19.3 (8.0, 30.1) | 6.2 (2.4, 10.0) | 15.6 (6.2, 25.4) |
| Non-exclusive breastfeeding | 1.8 (-2.0, 5.5) | 0.6 (-0.7, 1.8) | 1.4 (-1.7, 4.5) |
| HIV infection without HAART before 2 years | No data | No data | No data |
| Discontinued breastfeeding | 1.6 (0.2, 4.2) | 0.5 (0.1, 1.3) | 1.3 (0.1, 3.2) |
| **Water, sanitation and biomass fuel use** | **16.9 (14.6, 19.2)** | **5.4 (4.1, 6.7)** | **13.7 (10.4, 17.0)** |
| Unimproved sanitation | 15.3 (13.1, 17.7) | 4.9 (3.7, 6.1) | 12.4 (9.5, 15.5) |
| Unimproved water | 1.0 (0.6, 1.6) | 0.3 (0.2, 0.5) | 0.8 (0.5, 1.3) |
| Use of biomass fuels | 0.8 (0.5, 1.2) | 0.2 (0.1, 0.4) | 0.6 (0.4, 1.0) |

**Fig 1: Stunting cases among 2-year olds attributable to individual risk factors**


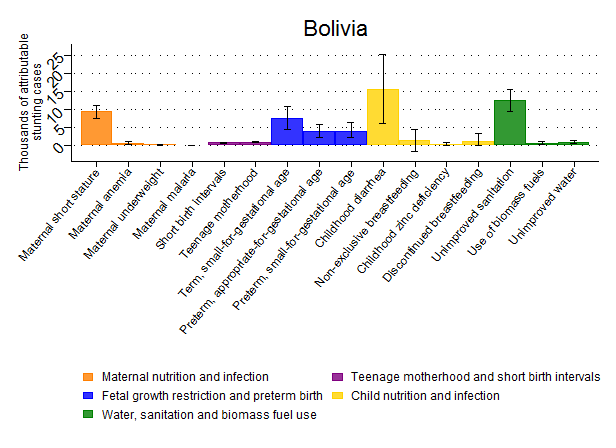


Botswana

**Region: Sub-Saharan Africa; Sub-region: Sub-Saharan Africa, Southern**

**Stunting prevalence among children age 2: 32.7%**

**Stunting cases among children age 2: 17,101**

**Population of children age 2: 52,298**

This country profile provides results for 2011 from the analysis presented in *Risk factors for childhood stunting in 137 developing countries: a comparative risk assessment analysis at global, regional, and country levels.*

**Table 1: Population attributable fraction (PAF in %), attributable stunting prevalence (percentage points) and number of stunting cases among 2-year olds attributable to individual risks and risk factor clusters (95% confidence intervals presented in parentheses)**

| **Description** | **PAF** | **Attributable stunting prevalence** | **Attributable stunting cases (thousands)** |
| --- | --- | --- | --- |
| **Maternal nutrition and infection** | **7.2 (5.6, 9.1)** | **2.3 (1.6, 3.3)** | **1.2 (0.8, 1.7)** |
| Maternal short stature | 4.3 (4.1, 4.5) | 1.4 (1.0, 1.8) | 0.7 (0.5, 1.0) |
| Maternal underweight | 1.0 (0.3, 1.9) | 0.3 (0.1, 0.7) | 0.2 (0.0, 0.3) |
| Maternal malaria | 0.3 (0.1, 0.5) | 0.1 (0.0, 0.2) | 0.1 (0.0, 0.1) |
| Maternal anemia | 1.7 (0.4, 3.6) | 0.6 (0.1, 1.2) | 0.3 (0.1, 0.6) |
| **Teenage motherhood and short birth intervals** | **1.7 (1.6, 1.8)** | **0.6 (0.4, 0.7)** | **0.3 (0.2, 0.4)** |
| Teenage motherhood | 1.2 (1.1, 1.3) | 0.4 (0.3, 0.5) | 0.2 (0.2, 0.3) |
| Short birth intervals | 0.5 (0.4, 0.5) | 0.2 (0.1, 0.2) | 0.1 (0.1, 0.1) |
| **Fetal growth restriction and preterm birth** | **28.6 (23.7, 33.3)** | **9.4 (6.4, 12.7)** | **4.9 (3.4, 6.6)** |
| Preterm, small-for-gestational age | 5.8 (2.1, 10.0) | 1.9 (0.7, 3.5) | 1.0 (0.4, 1.9) |
| Preterm, appropriate-for-gestational age | 8.0 (3.4, 13.3) | 2.6 (1.1, 4.8) | 1.4 (0.6, 2.5) |
| Term, small-for-gestational age | 17.6 (12.7, 22.4) | 5.7 (3.7, 8.2) | 3.0 (1.9, 4.3) |
| Low birth weight | 20.1 (17.0, 23.3) | 6.6 (4.6, 8.8) | 3.4 (2.4, 4.6) |
| **Child nutrition and infection** | **11.8 (4.4, 21.3)** | **3.9 (1.4, 7.4)** | **2.0 (0.7, 3.9)** |
| Childhood zinc deficiency | 1.5 (0.6, 2.7) | 0.5 (0.2, 0.9) | 0.3 (0.1, 0.5) |
| Childhood diarrhea | 11.2 (3.9, 20.4) | 3.6 (1.2, 7.1) | 1.9 (0.6, 3.7) |
| Non-exclusive breastfeeding | 1.9 (0.1, 4.6) | 0.6 (0.1, 1.5) | 0.3 (0.0, 0.8) |
| HIV infection without HAART before 2 years | 0.3 (0.1, 0.8) | 0.1 (0.0, 0.3) | 0.1 (0.0, 0.1) |
| Discontinued breastfeeding | 1.5 (0.2, 3.8) | 0.5 (0.1, 1.2) | 0.3 (0.0, 0.7) |
| **Water, sanitation and biomass fuel use** | **13.4 (11.0, 15.9)** | **4.4 (3.0, 6.0)** | **2.3 (1.6, 3.1)** |
| Unimproved sanitation | 10.9 (8.6, 13.3) | 3.6 (2.4, 4.9) | 1.9 (1.2, 2.6) |
| Unimproved water | 0.3 (0.1, 0.4) | 0.1 (0.0, 0.1) | 0.0 (0.0, 0.1) |
| Use of biomass fuels | 2.6 (1.6, 3.8) | 0.8 (0.5, 1.3) | 0.4 (0.2, 0.7) |

**Fig 1: Stunting cases among 2-year olds attributable to individual risk factors**


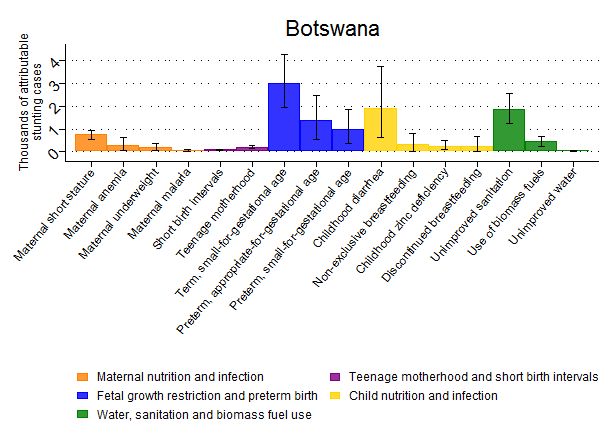


Brazil

**Region: Latin America/Caribbean; Sub-region: Latin America, Tropical**

**Stunting prevalence among children age 2: 9.2%**

**Stunting cases among children age 2: 288,224**

**Population of children age 2: 3,123,488**

This country profile provides results for 2011 from the analysis presented in *Risk factors for childhood stunting in 137 developing countries: a comparative risk assessment analysis at global, regional, and country levels.*

**Table 1: Population attributable fraction (PAF in %), attributable stunting prevalence (percentage points) and number of stunting cases among 2-year olds attributable to individual risks and risk factor clusters (95% confidence intervals presented in parentheses)**

| **Description** | **PAF** | **Attributable stunting prevalence** | **Attributable stunting cases (thousands)** |
| --- | --- | --- | --- |
| **Maternal nutrition and infection** | **7.4 (6.4, 8.7)** | **0.7 (0.4, 1.0)** | **21.4 (12.9, 30.7)** |
| Maternal short stature | 5.9 (5.8, 6.1) | 0.5 (0.3, 0.8) | 17.1 (10.5, 23.6) |
| Maternal underweight | 0.4 (0.2, 0.7) | 0.0 (0.0, 0.1) | 1.2 (0.5, 2.2) |
| Maternal malaria | 0.0 (0.0, 0.0) | 0.0 (0.0, 0.0) | 0.0 (0.0, 0.0) |
| Maternal anemia | 1.2 (0.2, 2.6) | 0.1 (0.0, 0.2) | 3.4 (0.5, 7.8) |
| **Teenage motherhood and short birth intervals** | **2.5 (2.3, 2.6)** | **0.2 (0.1, 0.3)** | **7.1 (4.3, 9.9)** |
| Teenage motherhood | 1.3 (1.2, 1.5) | 0.1 (0.1, 0.2) | 3.9 (2.4, 5.4) |
| Short birth intervals | 1.1 (1.0, 1.2) | 0.1 (0.1, 0.1) | 3.3 (2.0, 4.6) |
| **Fetal growth restriction and preterm birth** | **21.5 (17.4, 25.6)** | **2.0 (1.2, 2.9)** | **62.0 (37.3, 90.7)** |
| Preterm, small-for-gestational age | 5.2 (2.5, 8.7) | 0.5 (0.2, 0.9) | 15.1 (6.1, 27.5) |
| Preterm, appropriate-for-gestational age | 5.2 (2.8, 8.0) | 0.5 (0.2, 0.9) | 15.0 (7.0, 27.4) |
| Term, small-for-gestational age | 12.6 (9.0, 16.5) | 1.2 (0.7, 1.8) | 36.4 (20.6, 57.4) |
| Low birth weight | 13.9 (11.6, 16.3) | 1.3 (0.7, 1.9) | 40.1 (23.3, 58.5) |
| **Child nutrition and infection** | **17.9 (7.3, 28.6)** | **1.6 (0.6, 3.0)** | **51.4 (19.4, 92.4)** |
| Childhood zinc deficiency | 1.1 (0.2, 4.4) | 0.1 (0.0, 0.5) | 3.3 (0.6, 14.3) |
| Childhood diarrhea | 17.4 (7.0, 28.1) | 1.6 (0.6, 2.9) | 50.0 (18.5, 90.4) |
| Non-exclusive breastfeeding | 2.9 (0.2, 6.4) | 0.3 (0.0, 0.6) | 8.4 (0.7, 19.7) |
| Discontinued breastfeeding | 3.1 (0.5, 6.5) | 0.3 (0.0, 0.6) | 8.8 (1.4, 19.4) |
| HIV infection without HAART before 2 years | No data | No data | No data |
| **Water, sanitation and biomass fuel use** | **6.5 (5.2, 7.6)** | **0.6 (0.4, 0.9)** | **18.6 (11.3, 26.6)** |
| Unimproved sanitation | 5.9 (4.9, 7.0) | 0.5 (0.3, 0.8) | 17.1 (10.3, 24.7) |
| Unimproved water | 0.3 (0.1, 0.4) | 0.0 (0.0, 0.0) | 0.7 (0.4, 1.3) |
| Use of biomass fuels | 0.3 (-0.1, 0.8) | 0.0 (0.0, 0.1) | 0.9 (-0.4, 2.4) |

**Fig 1: Stunting cases among 2-year olds attributable to individual risk factors**


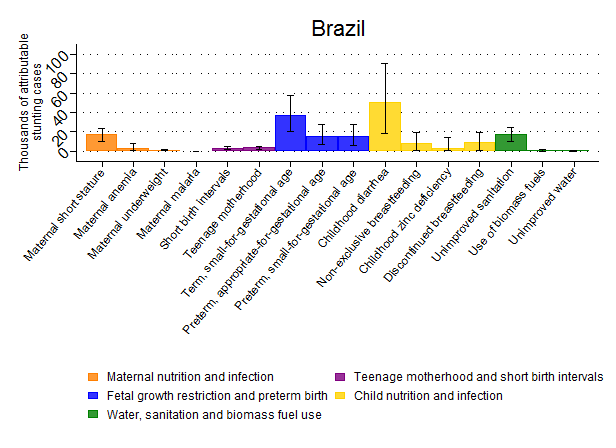


Burkina Faso

**Region: Sub-Saharan Africa; Sub-region: Sub-Saharan Africa, West**

**Stunting prevalence among children age 2: 46.6%**

**Stunting cases among children age 2: 308,377**

**Population of children age 2: 661,146**

This country profile provides results for 2011 from the analysis presented in *Risk factors for childhood stunting in 137 developing countries: a comparative risk assessment analysis at global, regional, and country levels.*

**Table 1: Population attributable fraction (PAF in %), attributable stunting prevalence (percentage points) and number of stunting cases among 2-year olds attributable to individual risks and risk factor clusters (95% confidence intervals presented in parentheses)**

| **Description** | **PAF** | **Attributable stunting prevalence** | **Attributable stunting cases (thousands)** |
| --- | --- | --- | --- |
| **Maternal nutrition and infection** | **10.7 (8.1, 13.4)** | **5.0 (3.6, 6.5)** | **33.0 (23.6, 42.8)** |
| Maternal short stature | 3.6 (3.5, 3.7) | 1.7 (1.4, 1.9) | 11.1 (9.3, 12.9) |
| Maternal underweight | 1.5 (0.7, 2.4) | 0.7 (0.3, 1.1) | 4.5 (2.3, 7.3) |
| Maternal malaria | 3.6 (1.5, 5.6) | 1.7 (0.7, 2.7) | 11.3 (4.7, 17.8) |
| Maternal anemia | 2.4 (0.8, 4.1) | 1.1 (0.4, 1.9) | 7.5 (2.4, 12.7) |
| **Teenage motherhood and short birth intervals** | **1.5 (1.4, 1.6)** | **0.7 (0.6, 0.8)** | **4.6 (3.9, 5.3)** |
| Teenage motherhood | 0.9 (0.8, 0.9) | 0.4 (0.3, 0.5) | 2.7 (2.3, 3.2) |
| Short birth intervals | 0.6 (0.6, 0.7) | 0.3 (0.2, 0.3) | 1.9 (1.6, 2.2) |
| **Fetal growth restriction and preterm birth** | **27.3 (23.9, 30.9)** | **12.7 (10.4, 15.2)** | **84.3 (68.5, 100.7)** |
| Preterm, small-for-gestational age | 4.3 (2.0, 7.0) | 2.0 (1.0, 3.3) | 13.1 (6.4, 22.0) |
| Preterm, appropriate-for-gestational age | 6.0 (3.6, 8.9) | 2.8 (1.6, 4.2) | 18.6 (10.8, 27.9) |
| Term, small-for-gestational age | 19.2 (15.8, 23.0) | 9.0 (6.9, 11.3) | 59.2 (45.7, 74.5) |
| Low birth weight | 16.9 (14.2, 19.7) | 7.9 (6.1, 9.7) | 52.3 (40.3, 63.8) |
| **Child nutrition and infection** | **16.3 (6.6, 27.7)** | **7.6 (3.0, 13.0)** | **50.1 (19.8, 85.9)** |
| Childhood zinc deficiency | 1.5 (0.6, 2.6) | 0.7 (0.3, 1.2) | 4.7 (1.9, 8.1) |
| Childhood diarrhea | 15.6 (6.1, 27.2) | 7.3 (2.8, 12.6) | 48.1 (18.4, 83.6) |
| Non-exclusive breastfeeding | 1.8 (-0.5, 4.7) | 0.8 (-0.2, 2.2) | 5.5 (-1.4, 14.7) |
| Discontinued breastfeeding | 0.5 (0.0, 1.4) | 0.2 (0.0, 0.6) | 1.5 (0.1, 4.3) |
| HIV infection without HAART before 2 years | 0.1 (0.0, 0.2) | 0.0 (0.0, 0.1) | 0.2 (0.1, 0.5) |
| **Water, sanitation and biomass fuel use** | **28.6 (26.6, 30.6)** | **13.4 (11.1, 15.6)** | **88.3 (73.3, 103)** |
| Unimproved sanitation | 23.7 (21.8, 25.6) | 11.1 (9.1, 13.0) | 73.1 (60.5, 86.1) |
| Unimproved water | 1.9 (1.1, 2.7) | 0.9 (0.5, 1.3) | 5.8 (3.4, 8.7) |
| Use of biomass fuels | 4.7 (3.3, 6.0) | 2.2 (1.5, 2.9) | 14.4 (9.7, 19.1) |

**Fig 1: Stunting cases among 2-year olds attributable to individual risk factors**


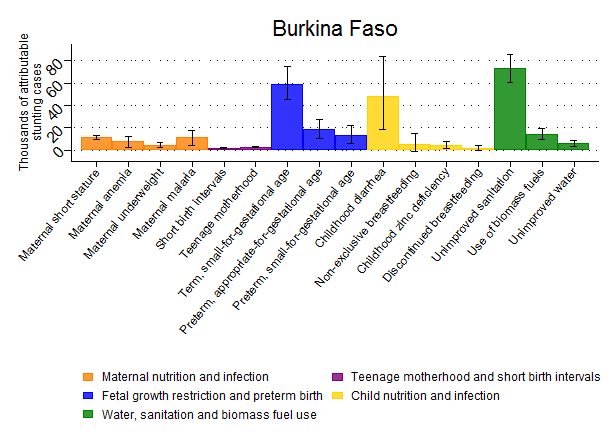


Burundi

**Region: Sub-Saharan Africa; Sub-region: Sub-Saharan Africa, East**

**Stunting prevalence among children age 2: 63.1%**

**Stunting cases among children age 2: 265,260**

**Population of children age 2: 420,634**

This country profile provides results for 2011 from the analysis presented in *Risk factors for childhood stunting in 137 developing countries: a comparative risk assessment analysis at global, regional, and country levels.*

**Table 1: Population attributable fraction (PAF in %), attributable stunting prevalence (percentage points) and number of stunting cases among 2-year olds attributable to individual risks and risk factor clusters (95% confidence intervals presented in parentheses)**

| **Description** | **PAF** | **Attributable stunting prevalence** | **Attributable stunting cases (thousands)** |
| --- | --- | --- | --- |
| **Maternal nutrition and infection** | **11.8 (10.4, 13.3)** | **7.5 (6.3, 8.8)** | **31.4 (26.4, 36.8)** |
| Maternal short stature | 8.8 (8.5, 9.1) | 5.5 (4.9, 6.2) | 23.3 (20.7, 26.0) |
| Maternal underweight | 1.4 (0.5, 2.5) | 0.9 (0.3, 1.6) | 3.7 (1.4, 6.8) |
| Maternal malaria | 0.5 (0.2, 0.8) | 0.3 (0.1, 0.5) | 1.2 (0.4, 2.1) |
| Maternal anemia | 1.5 (0.4, 2.7) | 1.0 (0.2, 1.8) | 4.0 (1.0, 7.4) |
| **Teenage motherhood and short birth intervals** | **1.4 (1.4, 1.5)** | **0.9 (0.8, 1.0)** | **3.8 (3.4, 4.3)** |
| Teenage motherhood | 0.5 (0.4, 0.5) | 0.3 (0.3, 0.4) | 1.3 (1.1, 1.5) |
| Short birth intervals | 1.0 (0.9, 1.0) | 0.6 (0.5, 0.7) | 2.6 (2.2, 2.9) |
| **Fetal growth restriction and preterm birth** | **29.2 (24.7, 33.4)** | **18.4 (15.0, 22.2)** | **77.5 (63.1, 93.4)** |
| Preterm, small-for-gestational age | 4.3 (0.6, 8.6) | 2.7 (0.4, 5.5) | 11.4 (1.7, 23.0) |
| Preterm, appropriate-for-gestational age | 6.1 (1.0, 11.1) | 3.9 (0.6, 7.1) | 16.2 (2.5, 29.8) |
| Term, small-for-gestational age | 21.2 (17.3, 25.1) | 13.4 (10.6, 16.3) | 56.2 (44.7, 68.5) |
| Low birth weight | 17.7 (14.9, 20.6) | 11.2 (9.0, 13.5) | 47.0 (37.8, 56.6) |
| **Child nutrition and infection** | **16.0 (5.9, 27.2)** | **10.1 (3.7, 17.0)** | **42.4 (15.5, 71.5)** |
| Childhood zinc deficiency | 1.0 (0.4, 1.7) | 0.7 (0.3, 1.1) | 2.8 (1.1, 4.6) |
| Childhood diarrhea | 15.6 (5.4, 26.9) | 9.8 (3.4, 16.6) | 41.3 (14.1, 69.9) |
| Non-exclusive breastfeeding | 0.8 (-2.3, 3.6) | 0.5 (-1.5, 2.2) | 2.2 (-6.5, 9.3) |
| Discontinued breastfeeding | 0.3 (0.0, 0.9) | 0.2 (0.0, 0.6) | 0.8 (0.0, 2.4) |
| HIV infection without HAART before 2 years | 0.1 (0.0, 0.1) | 0.0 (0.0, 0.1) | 0.2 (0.1, 0.3) |
| **Water, sanitation and biomass fuel use** | **21.3 (17.7, 24.7)** | **13.5 (10.9, 16.0)** | **56.6 (45.8, 67.5)** |
| Unimproved sanitation | 15.5 (12.0, 18.8) | 9.8 (7.4, 12.3) | 41.2 (31.2, 51.7) |
| Unimproved water | 2.0 (0.9, 3.3) | 1.2 (0.6, 2.0) | 5.2 (2.3, 8.6) |
| Use of biomass fuels | 5.0 (3.6, 6.4) | 3.1 (2.2, 4.2) | 13.2 (9.2, 17.7) |

**Fig 1: Stunting cases among 2-year olds attributable to individual risk factors**


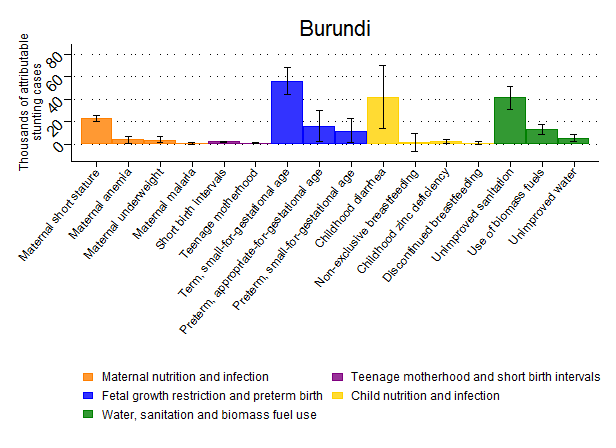


Cambodia

**Region: East Asia/Pacific; Sub-region: Asia, Southeast**

**Stunting prevalence among children age 2: 42.5%**

**Stunting cases among children age 2: 155,421**

**Population of children age 2: 365,299**

This country profile provides results for 2011 from the analysis presented in *Risk factors for childhood stunting in 137 developing countries: a comparative risk assessment analysis at global, regional, and country levels.*

**Table 1: Population attributable fraction (PAF in %), attributable stunting prevalence (percentage points) and number of stunting cases among 2-year olds attributable to individual risks and risk factor clusters (95% confidence intervals presented in parentheses)**

| **Description** | **PAF** | **Attributable stunting prevalence** | **Attributable stunting cases (thousands)** |
| --- | --- | --- | --- |
| **Maternal nutrition and infection** | **13.7 (12.4, 15.1)** | **5.8 (4.9, 6.8)** | **21.3 (17.8, 24.9)** |
| Maternal short stature | 11.0 (10.8, 11.3) | 4.7 (4.1, 5.3) | 17.1 (14.8, 19.5) |
| Maternal underweight | 1.3 (0.6, 2.1) | 0.5 (0.3, 0.9) | 2.0 (1.0, 3.2) |
| Maternal malaria | 0.0 (0.0, 0.0) | 0.0 (0.0, 0.0) | 0.0 (0.0, 0.0) |
| Maternal anemia | 1.7 (0.5, 3.0) | 0.7 (0.2, 1.3) | 2.7 (0.8, 4.7) |
| **Teenage motherhood and short birth intervals** | **1.1 (1.1, 1.2)** | **0.5 (0.4, 0.6)** | **1.8 (1.5, 2.0)** |
| Teenage motherhood | 0.5 (0.5, 0.6) | 0.2 (0.2, 0.2) | 0.8 (0.7, 0.9) |
| Short birth intervals | 0.6 (0.6, 0.7) | 0.3 (0.2, 0.3) | 1.0 (0.9, 1.2) |
| **Fetal growth restriction and preterm birth** | **26.0 (19.9, 31.7)** | **11.0 (8.2, 13.9)** | **40.4 (29.9, 50.9)** |
| Preterm, small-for-gestational age | 6.0 (0.7, 12.2) | 2.5 (0.3, 5.1) | 9.3 (1.1, 18.7) |
| Preterm, appropriate-for-gestational age | 5.5 (0.7, 10.5) | 2.3 (0.3, 4.5) | 8.5 (1.1, 16.3) |
| Term, small-for-gestational age | 16.6 (11.5, 22.3) | 7.1 (4.8, 9.8) | 25.9 (17.6, 35.7) |
| Low birth weight | 13.3 (11.1, 15.7) | 5.7 (4.5, 7.1) | 20.7 (16.6, 25.9) |
| **Child nutrition and infection** | **12.6 (5.4, 20.5)** | **5.4 (2.2, 8.8)** | **19.5 (8.1, 32.2)** |
| Childhood zinc deficiency | 0.6 (0.2, 1.0) | 0.3 (0.1, 0.4) | 0.9 (0.4, 1.6) |
| Childhood diarrhea | 12.3 (5.2, 20.3) | 5.2 (2.1, 8.7) | 19.1 (7.7, 31.7) |
| Non-exclusive breastfeeding | 1.1 (-1.3, 3.6) | 0.5 (-0.5, 1.6) | 1.7 (-1.9, 5.7) |
| HIV infection without HAART before 2 years | No data | No data | No data |
| Discontinued breastfeeding | 0.9 (0.1, 2.3) | 0.4 (0.0, 1.0) | 1.3 (0.1, 3.5) |
| **Water, sanitation and biomass fuel use** | **24.1 (21.8, 26.3)** | **10.3 (8.6, 12.1)** | **37.5 (31.5, 44.2)** |
| Unimproved sanitation | 19.1 (17.2, 21.2) | 8.1 (6.7, 9.6) | 29.7 (24.4, 35.0) |
| Unimproved water | 2.8 (1.7, 4.1) | 1.2 (0.7, 1.8) | 4.3 (2.7, 6.4) |
| Use of biomass fuels | 3.5 (2.5, 4.6) | 1.5 (1.0, 2.0) | 5.4 (3.7, 7.3) |

**Fig 1: Stunting cases among 2-year olds attributable to individual risk factors**


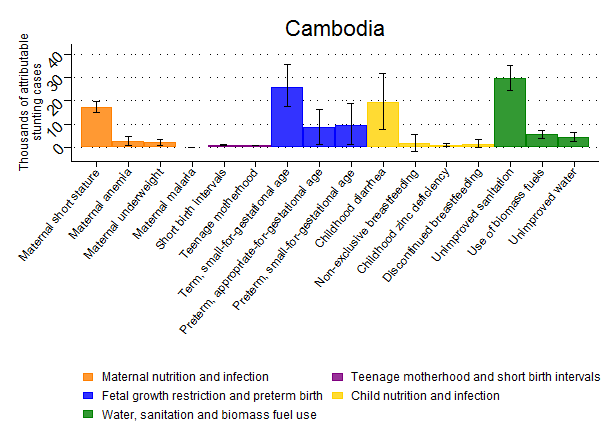


Cameroon

**Region: Sub-Saharan Africa; Sub-region: Sub-Saharan Africa, West**

**Stunting prevalence among children age 2: 41.0%**

**Stunting cases among children age 2: 326,275**

**Population of children age 2: 795,095**

This country profile provides results for 2011 from the analysis presented in *Risk factors for childhood stunting in 137 developing countries: a comparative risk assessment analysis at global, regional, and country levels.*

**Table 1: Population attributable fraction (PAF in %), attributable stunting prevalence (percentage points) and number of stunting cases among 2-year olds attributable to individual risks and risk factor clusters (95% confidence intervals presented in parentheses)**

| **Description** | **PAF** | **Attributable stunting prevalence** | **Attributable stunting cases (thousands)** |
| --- | --- | --- | --- |
| **Maternal nutrition and infection** | **9.4 (7.5, 11.5)** | **3.9 (2.9, 5.0)** | **30.7 (22.8, 40.0)** |
| Maternal short stature | 4.7 (4.5, 4.8) | 1.9 (1.6, 2.3) | 15.3 (12.6, 18.3) |
| Maternal underweight | 0.8 (0.4, 1.4) | 0.3 (0.1, 0.6) | 2.6 (1.2, 4.7) |
| Maternal malaria | 2.1 (0.8, 3.4) | 0.9 (0.3, 1.4) | 6.8 (2.7, 11.4) |
| Maternal anemia | 2.2 (0.7, 3.7) | 0.9 (0.3, 1.6) | 7.0 (2.1, 12.5) |
| **Teenage motherhood and short birth intervals** | **2.3 (2.2, 2.4)** | **0.9 (0.8, 1.1)** | **7.5 (6.2, 9.0)** |
| Teenage motherhood | 1.4 (1.3, 1.4) | 0.6 (0.5, 0.7) | 4.4 (3.7, 5.3) |
| Short birth intervals | 0.9 (0.9, 1.0) | 0.4 (0.3, 0.5) | 3.1 (2.5, 3.7) |
| **Fetal growth restriction and preterm birth** | **28.1 (23.5, 32.2)** | **11.6 (8.9, 14.3)** | **91.9 (70.7, 113.7)** |
| Preterm, small-for-gestational age | 4.9 (1.7, 8.9) | 2.0 (0.6, 3.7) | 16.0 (5.1, 29.7) |
| Preterm, appropriate-for-gestational age | 6.9 (2.6, 11.1) | 2.8 (1.0, 4.6) | 22.4 (8.3, 36.9) |
| Term, small-for-gestational age | 18.8 (14.9, 22.7) | 7.7 (5.7, 10.0) | 61.5 (45.4, 79.6) |
| Low birth weight | 17.2 (14.4, 20.0) | 7.1 (5.5, 8.9) | 56.2 (43.4, 70.9) |
| **Child nutrition and infection** | **15.2 (5.9, 26.0)** | **6.2 (2.5, 10.9)** | **49.5 (19.6, 87.0)** |
| Childhood zinc deficiency | 1.4 (0.5, 2.5) | 0.6 (0.2, 1.0) | 4.4 (1.6, 8.2) |
| Childhood diarrhea | 14.6 (5.4, 25.4) | 6.0 (2.2, 10.8) | 47.6 (17.4, 85.5) |
| Non-exclusive breastfeeding | 1.8 (-0.9, 5.0) | 0.7 (-0.3, 2.1) | 5.9 (-2.7, 16.3) |
| Discontinued breastfeeding | 0.9 (0.1, 2.5) | 0.4 (0.0, 1.0) | 3.0 (0.2, 8.3) |
| HIV infection without HAART before 2 years | 0.3 (0.2, 0.8) | 0.1 (0.1, 0.3) | 1.1 (0.5, 2.5) |
| **Water, sanitation and biomass fuel use** | **21.1 (18.4, 23.8)** | **8.6 (6.9, 10.6)** | **68.7 (55.1, 84.0)** |
| Unimproved sanitation | 15.9 (13.4, 18.5) | 6.5 (5.1, 8.1) | 51.9 (40.4, 64.5) |
| Unimproved water | 2.1 (1.2, 3.2) | 0.9 (0.5, 1.4) | 6.9 (3.7, 10.9) |
| Use of biomass fuels | 4.1 (2.9, 5.4) | 1.7 (1.1, 2.3) | 13.3 (8.9, 18.4) |

**Fig 1: Stunting cases among 2-year olds attributable to individual risk factors**


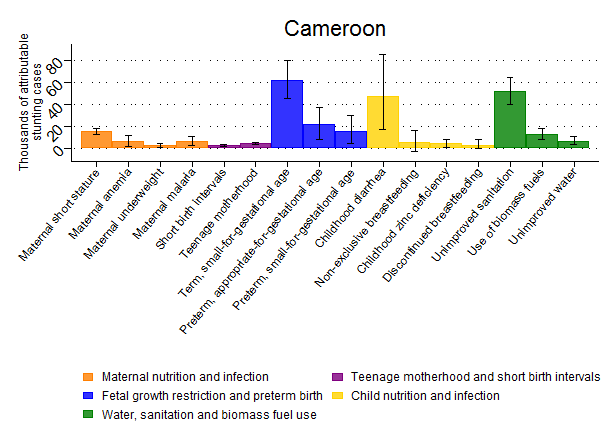


Cape Verde

**Region: Sub-Saharan Africa; Sub-region: Sub-Saharan Africa, West**

**Stunting prevalence among children age 2: 22.0%**

**Stunting cases among children age 2: 2,423**

**Population of children age 2: 11,011**

This country profile provides results for 2011 from the analysis presented in *Risk factors for childhood stunting in 137 developing countries: a comparative risk assessment analysis at global, regional, and country levels.*

**Table 1: Population attributable fraction (PAF in %), attributable stunting prevalence (percentage points) and number of stunting cases among 2-year olds attributable to individual risks and risk factor clusters (95% confidence intervals presented in parentheses)**

| **Description** | **PAF** | **Attributable stunting prevalence** | **Attributable stunting cases (thousands)** |
| --- | --- | --- | --- |
| **Maternal nutrition and infection** | **4.9 (3.9, 6.0)** | **1.1 (0.5, 1.7)** | **0.1 (0.1, 0.2)** |
| Maternal short stature | 3.2 (3.0, 3.5) | 0.7 (0.3, 1.1) | 0.1 (0.0, 0.1) |
| Maternal underweight | 0.6 (0.2, 1.1) | 0.1 (0.0, 0.3) | 0.0 (0.0, 0.0) |
| Maternal malaria | 0.0 (0.0, 0.0) | 0.0 (0.0, 0.0) | 0.0 (0.0, 0.0) |
| Maternal anemia | 1.1 (0.3, 2.1) | 0.2 (0.1, 0.6) | 0.0 (0.0, 0.1) |
| **Teenage motherhood and short birth intervals** | **1.9 (1.8, 2.0)** | **0.4 (0.2, 0.7)** | **0.0 (0.0, 0.1)** |
| Teenage motherhood | 1.1 (1.0, 1.1) | 0.2 (0.1, 0.4) | 0.0 (0.0, 0.0) |
| Short birth intervals | 0.8 (0.8, 0.9) | 0.2 (0.1, 0.3) | 0.0 (0.0, 0.0) |
| **Fetal growth restriction and preterm birth** | **20.9 (16.3, 25.4)** | **4.6 (2.2, 7.3)** | **0.5 (0.2, 0.8)** |
| Preterm, small-for-gestational age | 5.3 (2.2, 9.0) | 1.2 (0.4, 2.4) | 0.1 (0.0, 0.3) |
| Preterm, appropriate-for-gestational age | 7.1 (3.6, 11.2) | 1.6 (0.5, 2.9) | 0.2 (0.1, 0.3) |
| Term, small-for-gestational age | 10.0 (6.7, 13.6) | 2.2 (0.9, 3.8) | 0.2 (0.1, 0.4) |
| Low birth weight | 10.4 (8.6, 12.2) | 2.3 (1.0, 3.7) | 0.3 (0.1, 0.4) |
| **Child nutrition and infection** | **11.2 (3.8, 20.4)** | **2.4 (0.7, 5.0)** | **0.3 (0.1, 0.5)** |
| Childhood zinc deficiency | 1.6 (0.4, 5.5) | 0.3 (0.1, 1.2) | 0.0 (0.0, 0.1) |
| Childhood diarrhea | 10.5 (3.4, 19.0) | 2.3 (0.6, 4.7) | 0.3 (0.1, 0.5) |
| Non-exclusive breastfeeding | 1.4 (-0.4, 4.0) | 0.3 (-0.1, 0.9) | 0.0 (0.0, 0.1) |
| Discontinued breastfeeding | 0.9 (0.1, 2.5) | 0.2 (0.0, 0.6) | 0.0 (0.0, 0.1) |
| HIV infection without HAART before 2 years | 0.0 (0.0, 0.1) | 0.0 (0.0, 0.0) | 0.0 (0.0, 0.0) |
| **Water, sanitation and biomass fuel use** | **20.7 (18.5, 22.7)** | **4.5 (2.1, 7.1)** | **0.5 (0.2, 0.8)** |
| Unimproved sanitation | 18.9 (16.7, 21.0) | 4.2 (1.9, 6.5) | 0.5 (0.2, 0.7) |
| Unimproved water | 1.0 (0.5, 1.7) | 0.2 (0.1, 0.4) | 0.0 (0.0, 0.0) |
| Use of biomass fuels | 1.1 (0.6, 1.7) | 0.3 (0.1, 0.5) | 0.0 (0.0, 0.1) |

**Fig 1: Stunting cases among 2-year olds attributable to individual risk factors**


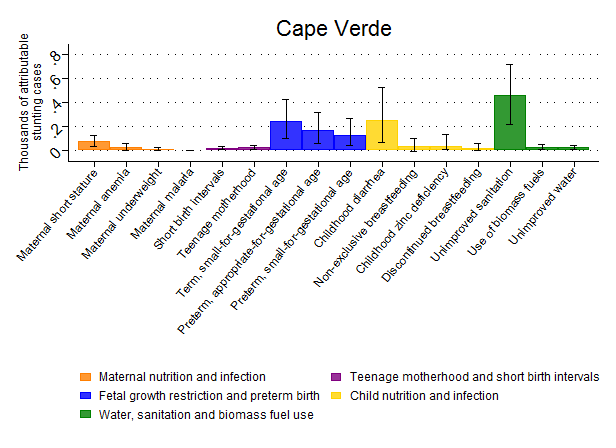


Central African Republic

**Region: Sub-Saharan Africa; Sub-region: Sub-Saharan Africa, Central**

**Stunting prevalence among children age 2: 49.3%**

**Stunting cases among children age 2: 76,792**

**Population of children age 2: 155,740**

This country profile provides results for 2011 from the analysis presented in *Risk factors for childhood stunting in 137 developing countries: a comparative risk assessment analysis at global, regional, and country levels.*

**Table 1: Population attributable fraction (PAF in %), attributable stunting prevalence (percentage points) and number of stunting cases among 2-year olds attributable to individual risks and risk factor clusters (95% confidence intervals presented in parentheses)**

| **Description** | **PAF** | **Attributable stunting prevalence** | **Attributable stunting cases (thousands)** |
| --- | --- | --- | --- |
| **Maternal nutrition and infection** | **11.7 (9.5, 14.2)** | **5.8 (4.0, 7.8)** | **9.0 (6.3, 12.2)** |
| Maternal short stature | 5.9 (5.6, 6.2) | 2.9 (2.3, 3.6) | 4.5 (3.5, 5.6) |
| Maternal underweight | 1.7 (0.6, 2.9) | 0.8 (0.3, 1.5) | 1.3 (0.4, 2.3) |
| Maternal malaria | 2.1 (0.8, 3.4) | 1.0 (0.4, 1.8) | 1.6 (0.6, 2.7) |
| Maternal anemia | 2.6 (0.8, 4.5) | 1.3 (0.4, 2.4) | 2.0 (0.6, 3.7) |
| **Teenage motherhood and short birth intervals** | **2.6 (2.4, 2.7)** | **1.3 (1.0, 1.6)** | **2.0 (1.5, 2.5)** |
| Teenage motherhood | 1.4 (1.3, 1.5) | 0.7 (0.5, 0.9) | 1.1 (0.8, 1.3) |
| Short birth intervals | 1.2 (1.1, 1.3) | 0.6 (0.4, 0.7) | 0.9 (0.7, 1.2) |
| **Fetal growth restriction and preterm birth** | **31.6 (26.9, 36.0)** | **15.6 (11.7, 19.9)** | **24.3 (18.2, 31.0)** |
| Preterm, small-for-gestational age | 4.6 (0.4, 9.5) | 2.3 (0.2, 4.7) | 3.6 (0.3, 7.4) |
| Preterm, appropriate-for-gestational age | 6.3 (0.4, 12.0) | 3.1 (0.2, 6.3) | 4.8 (0.3, 9.9) |
| Term, small-for-gestational age | 23.4 (19.2, 27.8) | 11.6 (8.3, 15.0) | 18.0 (13.0, 23.4) |
| Low birth weight | 20.0 (16.8, 23.2) | 9.9 (7.2, 12.8) | 15.4 (11.2, 20.0) |
| **Child nutrition and infection** | **16.1 (5.8, 28.7)** | **7.9 (2.7, 14.9)** | **12.3 (4.2, 23.1)** |
| Childhood zinc deficiency | 0.6 (0.2, 1.0) | 0.3 (0.1, 0.5) | 0.4 (0.2, 0.8) |
| Childhood diarrhea | 15.9 (5.6, 28.4) | 7.8 (2.6, 14.7) | 12.2 (4.0, 23.0) |
| Non-exclusive breastfeeding | 2.1 (-0.6, 5.6) | 1.0 (-0.3, 2.8) | 1.6 (-0.4, 4.4) |
| Discontinued breastfeeding | 0.6 (0.0, 1.7) | 0.3 (0.0, 0.9) | 0.5 (0.0, 1.3) |
| HIV infection without HAART before 2 years | 0.5 (0.2, 0.9) | 0.2 (0.1, 0.4) | 0.3 (0.2, 0.7) |
| **Water, sanitation and biomass fuel use** | **26.1 (22.3, 30.0)** | **12.9 (9.5, 16.6)** | **20.0 (14.8, 25.8)** |
| Unimproved sanitation | 19.9 (16.0, 23.6) | 9.8 (6.9, 13.0) | 15.3 (10.8, 20.3) |
| Unimproved water | 2.3 (1.1, 3.8) | 1.1 (0.5, 1.9) | 1.8 (0.8, 3.0) |
| Use of biomass fuels | 5.6 (4.0, 7.3) | 2.8 (1.8, 3.9) | 4.3 (2.9, 6.2) |

**Fig 1: Stunting cases among 2-year olds attributable to individual risk factors**


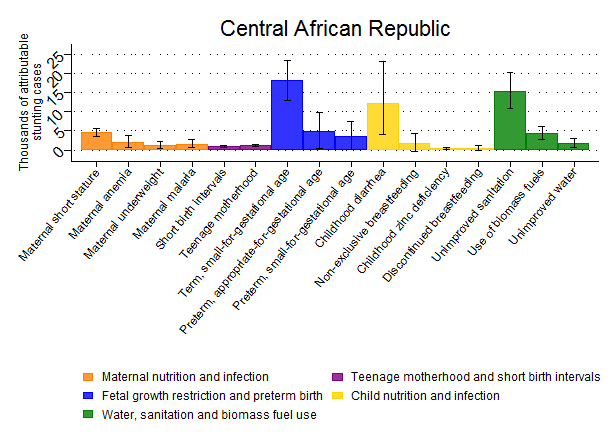


Chad

**Region: Sub-Saharan Africa; Sub-region: Sub-Saharan Africa, West**

**Stunting prevalence among children age 2: 51.9%**

**Stunting cases among children age 2: 292,555**

**Population of children age 2: 563,924**

This country profile provides results for 2011 from the analysis presented in *Risk factors for childhood stunting in 137 developing countries: a comparative risk assessment analysis at global, regional, and country levels.*

**Table 1: Population attributable fraction (PAF in %), attributable stunting prevalence (percentage points) and number of stunting cases among 2-year olds attributable to individual risks and risk factor clusters (95% confidence intervals presented in parentheses)**

| **Description** | **PAF** | **Attributable stunting prevalence** | **Attributable stunting cases (thousands)** |
| --- | --- | --- | --- |
| **Maternal nutrition and infection** | **10.8 (7.8, 14.1)** | **5.6 (3.6, 8.2)** | **31.7 (20.2, 46.1)** |
| Maternal short stature | 3.9 (3.7, 4.1) | 2.0 (1.4, 2.7) | 11.5 (7.8, 15.0) |
| Maternal underweight | 2.6 (1.3, 4.3) | 1.3 (0.6, 2.3) | 7.6 (3.6, 13.0) |
| Maternal malaria | 1.0 (0.4, 1.6) | 0.5 (0.2, 0.9) | 2.8 (1.0, 5.0) |
| Maternal anemia | 3.8 (1.1, 6.9) | 2.0 (0.5, 3.8) | 11.1 (2.9, 21.5) |
| **Teenage motherhood and short birth intervals** | **2.6 (2.5, 2.8)** | **1.4 (0.9, 1.8)** | **7.7 (5.3, 10.1)** |
| Teenage motherhood | 1.4 (1.3, 1.5) | 0.7 (0.5, 1.0) | 4.2 (2.8, 5.4) |
| Short birth intervals | 1.2 (1.1, 1.3) | 0.6 (0.4, 0.8) | 3.6 (2.5, 4.8) |
| **Fetal growth restriction and preterm birth** | **36.8 (32.3, 41.0)** | **19.1 (12.9, 25.6)** | **107.7 (72.9, 144.6)** |
| Preterm, small-for-gestational age | 4.4 (0.2, 8.6) | 2.3 (0.1, 4.8) | 12.9 (0.7, 26.8) |
| Preterm, appropriate-for-gestational age | 6.0 (0.6, 11.8) | 3.1 (0.3, 6.3) | 17.5 (1.8, 35.5) |
| Term, small-for-gestational age | 29.6 (25.0, 34.9) | 15.4 (10.0, 21.0) | 86.7 (56.5, 118.5) |
| Low birth weight | 29.4 (25.3, 33.5) | 15.3 (10.0, 20.6) | 86.2 (56.6, 116.3) |
| **Child nutrition and infection** | **18.5 (7.2, 32.6)** | **9.6 (3.4, 18.2)** | **54.2 (19.4, 102.9)** |
| Childhood zinc deficiency | 1.1 (0.4, 2.0) | 0.6 (0.2, 1.1) | 3.2 (1.1, 6.2) |
| Childhood diarrhea | 18.0 (6.8, 32.2) | 9.4 (3.3, 17.9) | 52.9 (18.8, 101.2) |
| Non-exclusive breastfeeding | 2.4 (0.0, 5.9) | 1.2 (0.0, 3.2) | 7.0 (0.0, 18.3) |
| HIV infection without HAART before 2 years | 0.2 (0.1, 0.4) | 0.1 (0.0, 0.2) | 0.6 (0.3, 1.3) |
| Discontinued breastfeeding | 0.9 (0.0, 2.6) | 0.5 (0.0, 1.3) | 2.6 (0.0, 7.4) |
| **Water, sanitation and biomass fuel use** | **32.8 (29.9, 35.5)** | **17.0 (11.5, 22.8)** | **96.0 (65.0, 128.6)** |
| Unimproved sanitation | 24.1 (22.0, 26.4) | 12.5 (8.4, 16.7) | 70.6 (47.3, 94.1) |
| Unimproved water | 3.8 (2.4, 5.4) | 2.0 (1.1, 3.1) | 11.1 (6.2, 17.3) |
| Use of biomass fuels | 8.0 (5.7, 10.4) | 4.1 (2.5, 6.0) | 23.3 (14.2, 33.8) |

**Fig 1: Stunting cases among 2-year olds attributable to individual risk factors**


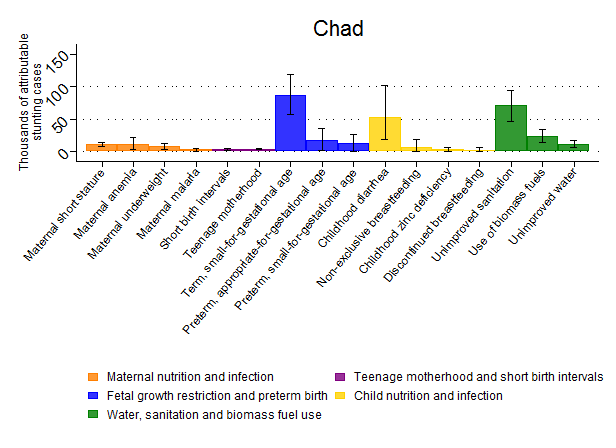


Chile

**Region: High Income Latin America/Caribbean; Sub-region: Latin America, Southern**

**Stunting prevalence among children age 2: 2.4%**

**Stunting cases among children age 2: 5,687**

**Population of children age 2: 238,068**

This country profile provides results for 2011 from the analysis presented in *Risk factors for childhood stunting in 137 developing countries: a comparative risk assessment analysis at global, regional, and country levels.*

**Table 1: Population attributable fraction (PAF in %), attributable stunting prevalence (percentage points) and number of stunting cases among 2-year olds attributable to individual risks and risk factor clusters (95% confidence intervals presented in parentheses)**

| **Description** | **PAF** | **Attributable stunting prevalence** | **Attributable stunting cases (thousands)** |
| --- | --- | --- | --- |
| **Maternal nutrition and infection** | **7.5 (6.8, 8.4)** | **0.2 (0.1, 0.2)** | **0.4 (0.3, 0.5)** |
| Maternal short stature | 6.8 (6.6, 7.0) | 0.2 (0.1, 0.2) | 0.4 (0.3, 0.5) |
| Maternal underweight | 0.1 (0.0, 0.2) | 0.0 (0.0, 0.0) | 0.0 (0.0, 0.0) |
| Maternal malaria | 0.0 (0.0, 0.0) | 0.0 (0.0, 0.0) | 0.0 (0.0, 0.0) |
| Maternal anemia | 0.7 (0.0, 1.6) | 0.0 (0.0, 0.0) | 0.0 (0.0, 0.1) |
| **Teenage motherhood and short birth intervals** | **2.3 (2.2, 2.4)** | **0.1 (0.0, 0.1)** | **0.1 (0.1, 0.2)** |
| Teenage motherhood | 1.4 (1.3, 1.4) | 0.0 (0.0, 0.0) | 0.1 (0.1, 0.1) |
| Short birth intervals | 1.0 (0.9, 1.0) | 0.0 (0.0, 0.0) | 0.1 (0.0, 0.1) |
| **Fetal growth restriction and preterm birth** | **17.1 (13.6, 20.5)** | **0.4 (0.3, 0.5)** | **1.0 (0.7, 1.3)** |
| Preterm, small-for-gestational age | 4.4 (3.0, 6.2) | 0.1 (0.1, 0.2) | 0.2 (0.2, 0.4) |
| Preterm, appropriate-for-gestational age | 4.3 (3.2, 5.4) | 0.1 (0.1, 0.1) | 0.2 (0.2, 0.3) |
| Term, small-for-gestational age | 9.4 (5.4, 12.9) | 0.2 (0.1, 0.3) | 0.5 (0.3, 0.8) |
| Low birth weight | 10.5 (8.7, 12.4) | 0.3 (0.2, 0.3) | 0.6 (0.4, 0.8) |
| **Child nutrition and infection** | **18.5 (7.4, 32.0)** | **0.4 (0.2, 0.8)** | **1.1 (0.4, 1.9)** |
| Childhood zinc deficiency | 5.4 (0.5, 29.7) | 0.1 (0.0, 0.8) | 0.3 (0.0, 1.9) |
| Childhood diarrhea | 16.5 (7.1, 26.3) | 0.4 (0.2, 0.6) | 0.9 (0.4, 1.5) |
| Non-exclusive breastfeeding | 1.4 (-2.1, 4.6) | 0.0 (0.0, 0.1) | 0.1 (-0.1, 0.3) |
| Discontinued breastfeeding | 2.2 (0.3, 5.2) | 0.1 (0.0, 0.1) | 0.1 (0.0, 0.3) |
| HIV infection without HAART before 2 years | No data | No data | No data |
| **Water, sanitation and biomass fuel use** | **1.6 (1.0, 2.1)** | **0.0 (0.0, 0.1)** | **0.1 (0.1, 0.1)** |
| Unimproved sanitation | 1.1 (0.7, 1.5) | 0.0 (0.0, 0.0) | 0.1 (0.0, 0.1) |
| Unimproved water | 0.2 (0.0, 0.3) | 0.0 (0.0, 0.0) | 0.0 (0.0, 0.0) |
| Use of biomass fuels | 0.3 (-0.1, 0.6) | 0.0 (0.0, 0.0) | 0.0 (0.0, 0.0) |

**Fig 1: Stunting cases among 2-year olds attributable to individual risk factors**


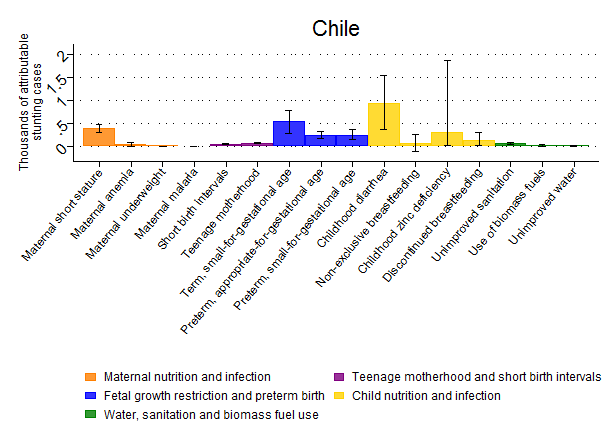


China

**Region: East Asia/Pacific; Sub-region: Asia, East**

**Stunting prevalence among children age 2: 17.4%**

**Stunting cases among children age 2: 2,868,327**

**Population of children age 2: 16,475,482**

This country profile provides results for 2011 from the analysis presented in *Risk factors for childhood stunting in 137 developing countries: a comparative risk assessment analysis at global, regional, and country levels.*

**Table 1: Population attributable fraction (PAF in %), attributable stunting prevalence (percentage points) and number of stunting cases among 2-year olds attributable to individual risks and risk factor clusters (95% confidence intervals presented in parentheses)**

| **Description** | **PAF** | **Attributable stunting prevalence** | **Attributable stunting cases (thousands)** |
| --- | --- | --- | --- |
| **Maternal nutrition and infection** | **6.4 (6.1, 6.7)** | **1.1 (0.7, 1.5)** | **184.1 (120.1, 248.7)** |
| Maternal short stature | 6.0 (5.8, 6.1) | 1.0 (0.7, 1.4) | 171.6 (112.1, 232.5) |
| Maternal underweight | 0.2 (0.1, 0.3) | 0.0 (0.0, 0.1) | 5.7 (2.7, 9.9) |
| Maternal malaria | 0.0 (0.0, 0.0) | 0.0 (0.0, 0.0) | 0.0 (0.0, 0.0) |
| Maternal anemia | 0.3 (0.1, 0.5) | 0.0 (0.0, 0.1) | 7.6 (1.8, 17.6) |
| **Teenage motherhood and short birth intervals** | **1.3 (1.2, 1.4)** | **0.2 (0.1, 0.3)** | **37.0 (24.3, 50.2)** |
| Teenage motherhood | 0.5 (0.4, 0.5) | 0.1 (0.1, 0.1) | 13.0 (8.5, 17.5) |
| Short birth intervals | 0.8 (0.8, 0.9) | 0.1 (0.1, 0.2) | 24.2 (15.9, 32.9) |
| **Fetal growth restriction and preterm birth** | **14.3 (10.3, 18.0)** | **2.5 (1.5, 3.7)** | **409.7 (250.3, 607.6)** |
| Preterm, small-for-gestational age | 4.8 (3.0, 7.1) | 0.8 (0.4, 1.4) | 138.2 (72.7, 231.6) |
| Preterm, appropriate-for-gestational age | 4.4 (3.0, 5.9) | 0.8 (0.4, 1.2) | 124.8 (70.2, 191.4) |
| Term, small-for-gestational age | 5.8 (1.7, 9.9) | 1.0 (0.3, 1.9) | 167.6 (46.1, 317.7) |
| Low birth weight | 4.4 (3.6, 5.3) | 0.8 (0.5, 1.1) | 126.9 (79.2, 177.9) |
| **Child nutrition and infection** | **10.9 (4.5, 18.1)** | **1.9 (0.7, 3.5)** | **313.1 (112.2, 573)** |
| Childhood zinc deficiency | 1.1 (0.2, 3.7) | 0.2 (0.0, 0.7) | 32.3 (5.4, 109.1) |
| Childhood diarrhea | 10.4 (4.3, 17.3) | 1.8 (0.6, 3.3) | 298.5 (106.3, 547.1) |
| Non-exclusive breastfeeding | 1.7 (0.1, 4.1) | 0.3 (0.0, 0.8) | 50.0 (1.6, 129) |
| Discontinued breastfeeding | 1.5 (0.2, 3.5) | 0.3 (0.0, 0.6) | 44.0 (5.9, 101.9) |
| HIV infection without HAART before 2 years | No data | No data | No data |
| **Water, sanitation and biomass fuel use** | **13.4 (7.7, 18.6)** | **2.3 (1.1, 3.7)** | **384.3 (183.4, 611.8)** |
| Unimproved sanitation | 12.3 (6.4, 17.5) | 2.1 (0.9, 3.5) | 352.5 (156.5, 577.6) |
| Unimproved water | 0.6 (0.2, 1.1) | 0.1 (0.0, 0.2) | 17.1 (4.5, 33.5) |
| Use of biomass fuels | 0.7 (0.4, 1.0) | 0.1 (0.1, 0.2) | 19.3 (10.5, 29.3) |

**Fig 1: Stunting cases among 2-year olds attributable to individual risk factors**


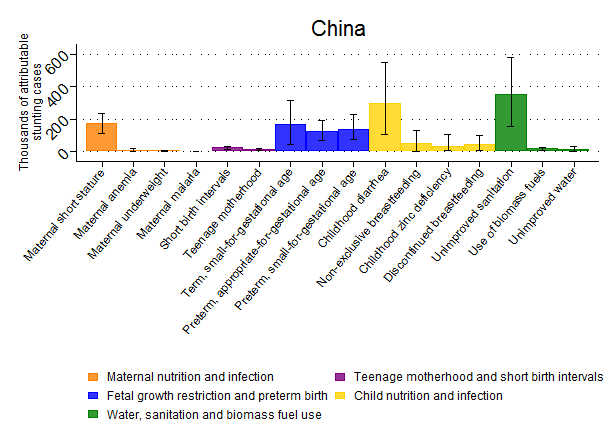


Colombia

**Region: Latin America/Caribbean; Sub-region: Latin America, Central**

**Stunting prevalence among children age 2: 16.3%**

**Stunting cases among children age 2: 128,026**

**Population of children age 2: 784,144**

This country profile provides results for 2011 from the analysis presented in *Risk factors for childhood stunting in 137 developing countries: a comparative risk assessment analysis at global, regional, and country levels.*

**Table 1: Population attributable fraction (PAF in %), attributable stunting prevalence (percentage points) and number of stunting cases among 2-year olds attributable to individual risks and risk factor clusters (95% confidence intervals presented in parentheses)**

| **Description** | **PAF** | **Attributable stunting prevalence** | **Attributable stunting cases (thousands)** |
| --- | --- | --- | --- |
| **Maternal nutrition and infection** | **10.0 (9.1, 11.0)** | **1.6 (1.3, 2.0)** | **12.8 (10.0, 15.8)** |
| Maternal short stature | 8.5 (8.4, 8.7) | 1.4 (1.1, 1.7) | 10.9 (8.7, 13.1) |
| Maternal underweight | 0.3 (0.1, 0.6) | 0.1 (0.0, 0.1) | 0.4 (0.2, 0.8) |
| Maternal malaria | 0.0 (0.0, 0.0) | 0.0 (0.0, 0.0) | 0.0 (0.0, 0.0) |
| Maternal anemia | 1.2 (0.3, 2.3) | 0.2 (0.1, 0.4) | 1.6 (0.4, 3.1) |
| **Teenage motherhood and short birth intervals** | **2.2 (2.1, 2.4)** | **0.4 (0.3, 0.4)** | **2.9 (2.3, 3.5)** |
| Teenage motherhood | 1.6 (1.5, 1.7) | 0.3 (0.2, 0.3) | 2.1 (1.7, 2.5) |
| Short birth intervals | 0.6 (0.6, 0.7) | 0.1 (0.1, 0.1) | 0.8 (0.6, 1.0) |
| **Fetal growth restriction and preterm birth** | **21.9 (18.2, 25.5)** | **3.6 (2.7, 4.5)** | **28.0 (21.2, 35.3)** |
| Preterm, small-for-gestational age | 4.9 (3.1, 7.0) | 0.8 (0.5, 1.2) | 6.3 (3.9, 9.5) |
| Preterm, appropriate-for-gestational age | 5.0 (3.4, 6.8) | 0.8 (0.5, 1.1) | 6.4 (4.0, 9.0) |
| Term, small-for-gestational age | 13.5 (9.6, 17.4) | 2.2 (1.5, 3.0) | 17.3 (11.4, 23.9) |
| Low birth weight | 14.8 (12.3, 17.3) | 2.4 (1.8, 3.1) | 18.9 (14.2, 24.1) |
| **Child nutrition and infection** | **17.3 (7.4, 27.6)** | **2.8 (1.2, 4.6)** | **22.2 (9.4, 36.2)** |
| Childhood zinc deficiency | 0.7 (0.3, 1.5) | 0.1 (0.0, 0.3) | 1.0 (0.3, 2.0) |
| Childhood diarrhea | 17.0 (7.1, 27.3) | 2.8 (1.2, 4.5) | 21.8 (9.0, 35.6) |
| Non-exclusive breastfeeding | 2.3 (-0.7, 5.8) | 0.4 (-0.1, 1.0) | 3.0 (-1.0, 7.8) |
| HIV infection without HAART before 2 years | No data | No data | No data |
| Discontinued breastfeeding | 2.0 (0.3, 4.6) | 0.3 (0.0, 0.8) | 2.5 (0.4, 5.9) |
| **Water, sanitation and biomass fuel use** | **7.8 (6.2, 9.6)** | **1.3 (0.9, 1.7)** | **10.0 (7.2, 13.0)** |
| Unimproved sanitation | 6.6 (5.0, 8.3) | 1.1 (0.8, 1.4) | 8.4 (6.0, 11.3) |
| Unimproved water | 0.5 (0.2, 0.9) | 0.1 (0.0, 0.2) | 0.7 (0.3, 1.2) |
| Use of biomass fuels | 0.8 (0.2, 1.4) | 0.1 (0.0, 0.2) | 1.0 (0.3, 1.9) |

**Fig 1: Stunting cases among 2-year olds attributable to individual risk factors**


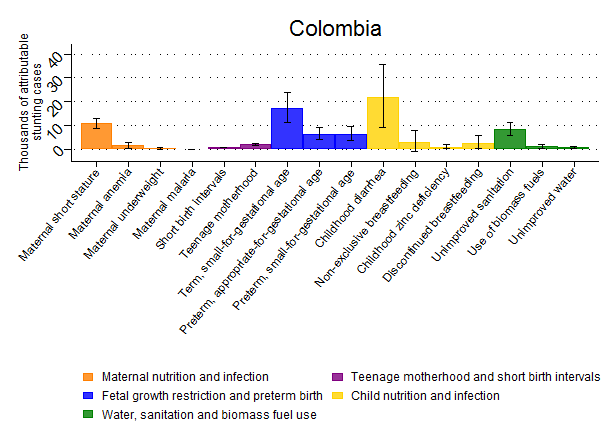


Comoros

**Region: Sub-Saharan Africa; Sub-region: Sub-Saharan Africa, East**

**Stunting prevalence among children age 2: 39.3%**

**Stunting cases among children age 2: 9,710**

**Population of children age 2: 24,703**

This country profile provides results for 2011 from the analysis presented in *Risk factors for childhood stunting in 137 developing countries: a comparative risk assessment analysis at global, regional, and country levels.*

**Table 1: Population attributable fraction (PAF in %), attributable stunting prevalence (percentage points) and number of stunting cases among 2-year olds attributable to individual risks and risk factor clusters (95% confidence intervals presented in parentheses)**

| **Description** | **PAF** | **Attributable stunting prevalence** | **Attributable stunting cases (thousands)** |
| --- | --- | --- | --- |
| **Maternal nutrition and infection** | **12.9 (10.4, 15.7)** | **5.1 (3.1, 7.3)** | **1.2 (0.8, 1.8)** |
| Maternal short stature | 8.3 (8.1, 8.6) | 3.3 (2.2, 4.4) | 0.8 (0.5, 1.1) |
| Maternal underweight | 2.0 (0.8, 3.5) | 0.8 (0.3, 1.5) | 0.2 (0.1, 0.4) |
| Maternal malaria | 0.0 (0.0, 0.0) | 0.0 (0.0, 0.0) | 0.0 (0.0, 0.0) |
| Maternal anemia | 3.0 (0.6, 5.9) | 1.2 (0.2, 2.5) | 0.3 (0.1, 0.6) |
| **Teenage motherhood and short birth intervals** | **2.4 (2.2, 2.6)** | **0.9 (0.6, 1.3)** | **0.2 (0.2, 0.3)** |
| Teenage motherhood | 0.8 (0.7, 0.9) | 0.3 (0.2, 0.4) | 0.1 (0.0, 0.1) |
| Short birth intervals | 1.6 (1.5, 1.8) | 0.6 (0.4, 0.9) | 0.2 (0.1, 0.2) |
| **Fetal growth restriction and preterm birth** | **37.9 (33.2, 41.9)** | **14.9 (9.8, 20.4)** | **3.7 (2.4, 5.0)** |
| Preterm, small-for-gestational age | 5.6 (1.6, 10.2) | 2.2 (0.5, 4.1) | 0.5 (0.1, 1.0) |
| Preterm, appropriate-for-gestational age | 7.3 (2.2, 12.3) | 2.9 (0.7, 5.5) | 0.7 (0.2, 1.3) |
| Term, small-for-gestational age | 28.9 (23.9, 34.2) | 11.4 (7.4, 15.8) | 2.8 (1.8, 3.9) |
| Low birth weight | 32.5 (28.0, 36.7) | 12.7 (8.5, 17.5) | 3.1 (2.1, 4.3) |
| **Child nutrition and infection** | **12.3 (4.6, 21.7)** | **4.8 (1.7, 9.0)** | **1.2 (0.4, 2.2)** |
| Childhood zinc deficiency | 1.3 (0.5, 2.5) | 0.5 (0.2, 1.0) | 0.1 (0.0, 0.3) |
| Childhood diarrhea | 11.7 (4.1, 21.0) | 4.6 (1.6, 8.8) | 1.1 (0.4, 2.2) |
| Non-exclusive breastfeeding | 2.0 (0.1, 4.8) | 0.8 (0.0, 2.0) | 0.2 (0.0, 0.5) |
| HIV infection without HAART before 2 years | 0.0 (0.0, 0.1) | 0.0 (0.0, 0.0) | 0.0 (0.0, 0.0) |
| Discontinued breastfeeding | 1.1 (0.1, 2.9) | 0.4 (0.0, 1.2) | 0.1 (0.0, 0.3) |
| **Water, sanitation and biomass fuel use** | **24.4 (21.4, 27.7)** | **9.6 (6.2, 13.2)** | **2.4 (1.5, 3.3)** |
| Unimproved sanitation | 18.1 (15.3, 21.1) | 7.1 (4.5, 10.0) | 1.8 (1.1, 2.5) |
| Unimproved water | 0.6 (0.1, 1.2) | 0.2 (0.1, 0.5) | 0.1 (0.0, 0.1) |
| Use of biomass fuels | 7.2 (5.0, 9.4) | 2.8 (1.7, 4.2) | 0.7 (0.4, 1.0) |

**Fig 1: Stunting cases among 2-year olds attributable to individual risk factors**


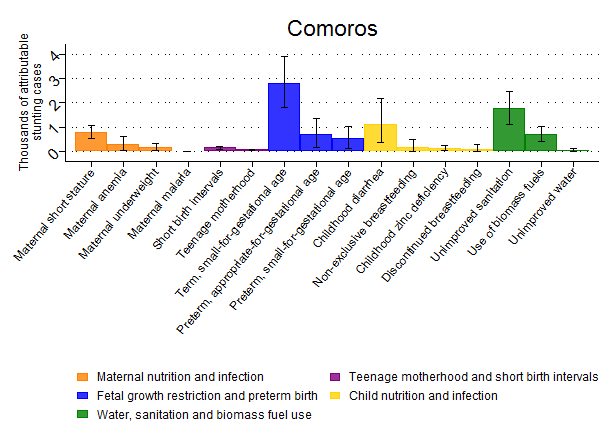


Congo

**Region: Sub-Saharan Africa; Sub-region: Sub-Saharan Africa, Central**

**Stunting prevalence among children age 2: 30.1%**

**Stunting cases among children age 2: 46,402**

**Population of children age 2: 153,936**

This country profile provides results for 2011 from the analysis presented in *Risk factors for childhood stunting in 137 developing countries: a comparative risk assessment analysis at global, regional, and country levels.*

**Table 1: Population attributable fraction (PAF in %), attributable stunting prevalence (percentage points) and number of stunting cases among 2-year olds attributable to individual risks and risk factor clusters (95% confidence intervals presented in parentheses)**

| **Description** | **PAF** | **Attributable stunting prevalence** | **Attributable stunting cases (thousands)** |
| --- | --- | --- | --- |
| **Maternal nutrition and infection** | **11.9 (9.7, 14.2)** | **3.6 (2.2, 5.1)** | **5.5 (3.4, 7.9)** |
| Maternal short stature | 6.1 (5.9, 6.3) | 1.8 (1.2, 2.5) | 2.8 (1.8, 3.8) |
| Maternal underweight | 1.6 (0.7, 2.6) | 0.5 (0.2, 0.9) | 0.7 (0.3, 1.3) |
| Maternal malaria | 1.8 (0.7, 2.9) | 0.5 (0.2, 1.0) | 0.8 (0.3, 1.5) |
| Maternal anemia | 3.0 (0.9, 4.9) | 0.9 (0.3, 1.6) | 1.4 (0.4, 2.5) |
| **Teenage motherhood and short birth intervals** | **1.9 (1.8, 2.0)** | **0.6 (0.4, 0.8)** | **0.9 (0.6, 1.2)** |
| Teenage motherhood | 1.3 (1.2, 1.5) | 0.4 (0.3, 0.6) | 0.6 (0.4, 0.8) |
| Short birth intervals | 0.5 (0.5, 0.6) | 0.2 (0.1, 0.2) | 0.3 (0.2, 0.3) |
| **Fetal growth restriction and preterm birth** | **31.7 (27.7, 35.3)** | **9.5 (6.0, 13.0)** | **14.7 (9.3, 20.0)** |
| Preterm, small-for-gestational age | 6.3 (2.7, 10.3) | 1.9 (0.8, 3.4) | 2.9 (1.2, 5.3) |
| Preterm, appropriate-for-gestational age | 8.5 (4.0, 12.9) | 2.6 (1.0, 4.4) | 3.9 (1.6, 6.7) |
| Term, small-for-gestational age | 20.3 (16.7, 24.2) | 6.1 (3.7, 8.8) | 9.4 (5.7, 13.5) |
| Low birth weight | 20.1 (17.0, 23.3) | 6.1 (3.7, 8.5) | 9.4 (5.7, 13.2) |
| **Child nutrition and infection** | **14.8 (5.4, 25.8)** | **4.4 (1.6, 8.4)** | **6.8 (2.4, 12.9)** |
| Childhood zinc deficiency | 1.2 (0.4, 2.7) | 0.4 (0.1, 0.8) | 0.6 (0.2, 1.3) |
| Childhood diarrhea | 14.2 (5.0, 25.4) | 4.3 (1.5, 8.2) | 6.6 (2.3, 12.7) |
| Non-exclusive breastfeeding | 2.0 (-0.3, 5.2) | 0.6 (-0.1, 1.6) | 0.9 (-0.2, 2.4) |
| Discontinued breastfeeding | 1.1 (0.1, 2.7) | 0.3 (0.0, 0.9) | 0.5 (0.0, 1.4) |
| HIV infection without HAART before 2 years | 1.0 (0.3, 4.2) | 0.3 (0.1, 1.3) | 0.5 (0.1, 2.0) |
| **Water, sanitation and biomass fuel use** | **26.9 (24.2, 29.6)** | **8.1 (5.1, 11.1)** | **12.5 (7.9, 17.1)** |
| Unimproved sanitation | 21.7 (19.2, 24.3) | 6.5 (4.1, 9.0) | 10.1 (6.4, 13.8) |
| Unimproved water | 2.1 (1.2, 3.1) | 0.6 (0.3, 1.1) | 1.0 (0.5, 1.6) |
| Use of biomass fuels | 4.7 (3.3, 6.2) | 1.4 (0.8, 2.2) | 2.2 (1.3, 3.3) |

**Fig 1: Stunting cases among 2-year olds attributable to individual risk factors**


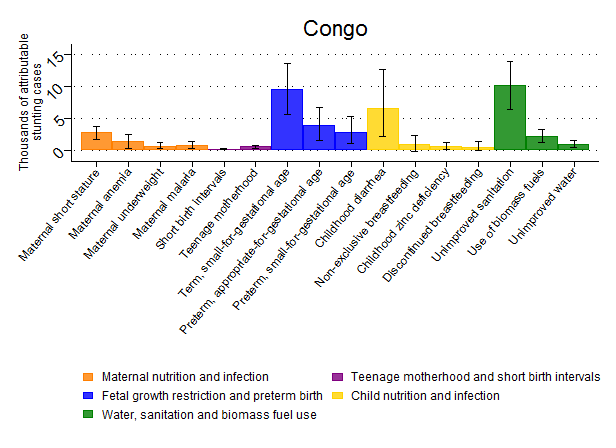


Costa Rica

**Region: Latin America/Caribbean; Sub-region: Latin America, Central**

**Stunting prevalence among children age 2: 9.2%**

**Stunting cases among children age 2: 6,579**

**Population of children age 2: 71,832**

This country profile provides results for 2011 from the analysis presented in *Risk factors for childhood stunting in 137 developing countries: a comparative risk assessment analysis at global, regional, and country levels.*

**Table 1: Population attributable fraction (PAF in %), attributable stunting prevalence (percentage points) and number of stunting cases among 2-year olds attributable to individual risks and risk factor clusters (95% confidence intervals presented in parentheses)**

| **Description** | **PAF** | **Attributable stunting prevalence** | **Attributable stunting cases (thousands)** |
| --- | --- | --- | --- |
| **Maternal nutrition and infection** | **9.2 (8.6, 10.1)** | **0.8 (0.4, 1.3)** | **0.6 (0.3, 1.0)** |
| Maternal short stature | 8.4 (8.0, 8.7) | 0.8 (0.4, 1.2) | 0.6 (0.3, 0.9) |
| Maternal underweight | 0.2 (0.1, 0.4) | 0.0 (0.0, 0.0) | 0.0 (0.0, 0.0) |
| Maternal malaria | 0.0 (0.0, 0.0) | 0.0 (0.0, 0.0) | 0.0 (0.0, 0.0) |
| Maternal anemia | 0.7 (0.1, 1.6) | 0.1 (0.0, 0.2) | 0.0 (0.0, 0.1) |
| **Teenage motherhood and short birth intervals** | **2.5 (2.4, 2.6)** | **0.2 (0.1, 0.3)** | **0.2 (0.1, 0.3)** |
| Teenage motherhood | 1.5 (1.4, 1.6) | 0.1 (0.1, 0.2) | 0.1 (0.0, 0.2) |
| Short birth intervals | 1.0 (0.9, 1.1) | 0.1 (0.0, 0.1) | 0.1 (0.0, 0.1) |
| **Fetal growth restriction and preterm birth** | **22.3 (15.9, 27.8)** | **2.0 (0.9, 3.3)** | **1.5 (0.7, 2.4)** |
| Preterm, small-for-gestational age | 7.5 (2.0, 13.1) | 0.7 (0.2, 1.4) | 0.5 (0.1, 1.0) |
| Preterm, appropriate-for-gestational age | 7.5 (2.3, 12.8) | 0.7 (0.2, 1.4) | 0.5 (0.1, 1.0) |
| Term, small-for-gestational age | 9.2 (5.7, 13.0) | 0.8 (0.4, 1.6) | 0.6 (0.3, 1.1) |
| Low birth weight | 10.8 (8.9, 12.8) | 1.0 (0.5, 1.6) | 0.7 (0.3, 1.1) |
| **Child nutrition and infection** | **18.1 (7.6, 29.4)** | **1.7 (0.5, 3.3)** | **1.2 (0.4, 2.3)** |
| Childhood zinc deficiency | 3.4 (0.7, 14.6) | 0.3 (0.0, 1.4) | 0.2 (0.0, 1.0) |
| Childhood diarrhea | 16.6 (6.5, 26.5) | 1.5 (0.5, 2.9) | 1.1 (0.4, 2.1) |
| Non-exclusive breastfeeding | 2.8 (0.2, 6.7) | 0.3 (0.0, 0.6) | 0.2 (0.0, 0.4) |
| HIV infection without HAART before 2 years | No data | No data | No data |
| Discontinued breastfeeding | 2.3 (0.4, 5.2) | 0.2 (0.0, 0.5) | 0.2 (0.0, 0.4) |
| **Water, sanitation and biomass fuel use** | **2.7 (1.8, 3.4)** | **0.2 (0.1, 0.4)** | **0.2 (0.1, 0.3)** |
| Unimproved sanitation | 2.2 (1.4, 2.9) | 0.2 (0.1, 0.3) | 0.1 (0.1, 0.2) |
| Unimproved water | 0.3 (0.1, 0.4) | 0.0 (0.0, 0.0) | 0.0 (0.0, 0.0) |
| Use of biomass fuels | 0.2 (0.1, 0.4) | 0.0 (0.0, 0.0) | 0.0 (0.0, 0.0) |

**Fig 1: Stunting cases among 2-year olds attributable to individual risk factors**


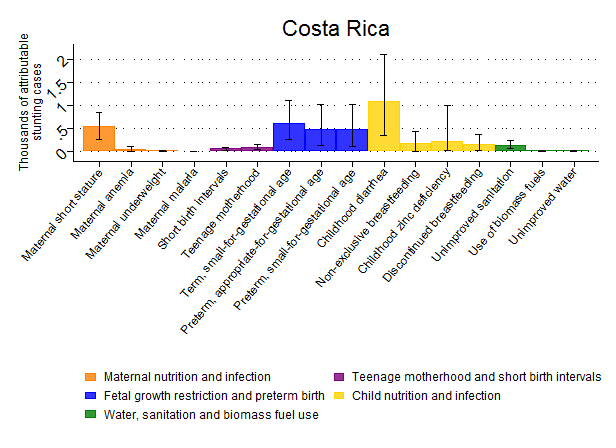


Cote d’Ivoire

**Region: Sub-Saharan Africa; Sub-region: Sub-Saharan Africa, West**

**Stunting prevalence among children age 2: 43.6%**

**Stunting cases among children age 2: 334,750**

**Population of children age 2: 768,059**

This country profile provides results for 2011 from the analysis presented in *Risk factors for childhood stunting in 137 developing countries: a comparative risk assessment analysis at global, regional, and country levels.*

**Table 1: Population attributable fraction (PAF in %), attributable stunting prevalence (percentage points) and number of stunting cases among 2-year olds attributable to individual risks and risk factor clusters (95% confidence intervals presented in parentheses)**

| **Description** | **PAF** | **Attributable stunting prevalence** | **Attributable stunting cases (thousands)** |
| --- | --- | --- | --- |
| **Maternal nutrition and infection** | **14.7 (11.2, 18.3)** | **6.4 (4.2, 8.7)** | **49.2 (32.2, 67.0)** |
| Maternal short stature | 5.3 (5.1, 5.5) | 2.3 (1.7, 2.9) | 17.8 (13.1, 22.4) |
| Maternal underweight | 1.3 (0.6, 2.2) | 0.6 (0.3, 1.0) | 4.4 (1.9, 7.8) |
| Maternal malaria | 5.5 (2.3, 8.4) | 2.4 (1.0, 3.9) | 18.4 (7.4, 29.9) |
| Maternal anemia | 3.4 (1.0, 5.9) | 1.5 (0.4, 2.7) | 11.5 (3.4, 20.6) |
| **Teenage motherhood and short birth intervals** | **2.0 (1.9, 2.2)** | **0.9 (0.6, 1.1)** | **6.8 (5.0, 8.7)** |
| Teenage motherhood | 1.4 (1.2, 1.5) | 0.6 (0.4, 0.8) | 4.6 (3.4, 6.0) |
| Short birth intervals | 0.7 (0.6, 0.7) | 0.3 (0.2, 0.4) | 2.2 (1.6, 2.9) |
| **Fetal growth restriction and preterm birth** | **34.4 (29.8, 38.4)** | **15.0 (10.7, 19.4)** | **115.1 (82.3, 148.6)** |
| Preterm, small-for-gestational age | 5.2 (1.1, 9.8) | 2.3 (0.4, 4.3) | 17.3 (3.4, 32.7) |
| Preterm, appropriate-for-gestational age | 6.9 (1.7, 12.5) | 3.0 (0.7, 5.8) | 23.1 (5.3, 44.7) |
| Term, small-for-gestational age | 25.6 (21.2, 30.1) | 11.2 (7.9, 14.7) | 85.8 (60.3, 113.2) |
| Low birth weight | 24.3 (20.7, 27.9) | 10.6 (7.6, 13.8) | 81.4 (58.3, 105.9) |
| **Child nutrition and infection** | **14.7 (5.9, 25.3)** | **6.4 (2.4, 11.1)** | **49.3 (18.4, 85.5)** |
| Childhood zinc deficiency | 1.2 (0.5, 2.1) | 0.5 (0.2, 1.0) | 4.0 (1.6, 7.4) |
| Childhood diarrhea | 14.2 (5.4, 24.6) | 6.2 (2.2, 11.0) | 47.6 (17.0, 84.2) |
| Non-exclusive breastfeeding | 1.9 (0.0, 4.7) | 0.8 (0.0, 2.1) | 6.3 (-0.1, 16.4) |
| HIV infection without HAART before 2 years | 0.3 (0.1, 0.5) | 0.1 (0.1, 0.2) | 0.8 (0.4, 1.7) |
| Discontinued breastfeeding | 0.7 (0.0, 2.0) | 0.3 (0.0, 0.9) | 2.3 (0.1, 6.9) |
| **Water, sanitation and biomass fuel use** | **27.7 (25.3, 30.2)** | **12.1 (8.8, 15.4)** | **92.7 (67.7, 118.3)** |
| Unimproved sanitation | 21.9 (19.7, 24.0) | 9.6 (6.9, 12.3) | 73.4 (53.2, 94.4) |
| Unimproved water | 1.6 (0.8, 2.6) | 0.7 (0.3, 1.2) | 5.4 (2.5, 9.4) |
| Use of biomass fuels | 5.8 (4.0, 7.8) | 2.5 (1.6, 3.7) | 19.5 (12.2, 28.7) |

**Fig 1: Stunting cases among 2-year olds attributable to individual risk factors**


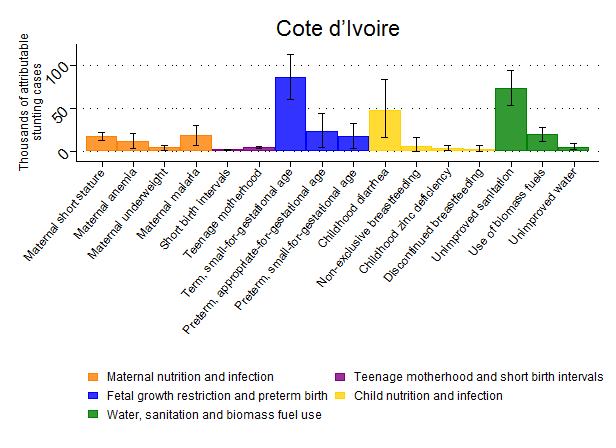


Cuba

**Region: Latin America/Caribbean; Sub-region: Caribbean**

**Stunting prevalence among children age 2: 9.7%**

**Stunting cases among children age 2: 11,907**

**Population of children age 2: 123,169**

This country profile provides results for 2011 from the analysis presented in *Risk factors for childhood stunting in 137 developing countries: a comparative risk assessment analysis at global, regional, and country levels.*

**Table 1: Population attributable fraction (PAF in %), attributable stunting prevalence (percentage points) and number of stunting cases among 2-year olds attributable to individual risks and risk factor clusters (95% confidence intervals presented in parentheses)**

| **Description** | **PAF** | **Attributable stunting prevalence** | **Attributable stunting cases (thousands)** |
| --- | --- | --- | --- |
| **Maternal nutrition and infection** | **7.0 (6.3, 7.7)** | **0.7 (0.3, 1.1)** | **0.8 (0.3, 1.4)** |
| Maternal short stature | 6.0 (5.8, 6.2) | 0.6 (0.2, 1.0) | 0.7 (0.3, 1.2) |
| Maternal underweight | 0.3 (0.1, 0.6) | 0.0 (0.0, 0.1) | 0.0 (0.0, 0.1) |
| Maternal malaria | 0.0 (0.0, 0.0) | 0.0 (0.0, 0.0) | 0.0 (0.0, 0.0) |
| Maternal anemia | 0.7 (0.1, 1.5) | 0.1 (0.0, 0.2) | 0.1 (0.0, 0.2) |
| **Teenage motherhood and short birth intervals** | **2.3 (2.2, 2.4)** | **0.2 (0.1, 0.4)** | **0.3 (0.1, 0.4)** |
| Teenage motherhood | 1.3 (1.3, 1.4) | 0.1 (0.0, 0.2) | 0.2 (0.1, 0.3) |
| Short birth intervals | 0.9 (0.9, 1.0) | 0.1 (0.0, 0.2) | 0.1 (0.0, 0.2) |
| **Fetal growth restriction and preterm birth** | **15.4 (11.9, 18.9)** | **1.5 (0.6, 2.5)** | **1.8 (0.7, 3.1)** |
| Preterm, small-for-gestational age | 3.9 (2.6, 5.6) | 0.4 (0.1, 0.7) | 0.5 (0.2, 0.8) |
| Preterm, appropriate-for-gestational age | 3.9 (2.8, 5.1) | 0.4 (0.1, 0.7) | 0.5 (0.2, 0.8) |
| Term, small-for-gestational age | 8.4 (4.6, 12.1) | 0.8 (0.3, 1.5) | 1.0 (0.3, 1.8) |
| Low birth weight | 9.4 (7.8, 11.1) | 0.9 (0.3, 1.5) | 1.1 (0.4, 1.8) |
| **Child nutrition and infection** | **18.1 (7.5, 30.1)** | **1.8 (0.5, 3.6)** | **2.2 (0.6, 4.4)** |
| Childhood zinc deficiency | 4.1 (0.8, 19.9) | 0.4 (0.0, 2.0) | 0.5 (0.1, 2.4) |
| Childhood diarrhea | 16.4 (6.4, 26.3) | 1.6 (0.4, 3.2) | 1.9 (0.5, 4.0) |
| Non-exclusive breastfeeding | 2.7 (0.1, 6.2) | 0.3 (0.0, 0.7) | 0.3 (0.0, 0.8) |
| HIV infection without HAART before 2 years | No data | No data | No data |
| Discontinued breastfeeding | 2.9 (0.5, 6.1) | 0.3 (0.0, 0.7) | 0.3 (0.0, 0.9) |
| **Water, sanitation and biomass fuel use** | **4.0 (2.6, 5.4)** | **0.4 (0.1, 0.7)** | **0.5 (0.2, 0.8)** |
| Unimproved sanitation | 3.2 (1.9, 4.6) | 0.3 (0.1, 0.6) | 0.4 (0.1, 0.7) |
| Unimproved water | 0.5 (0.3, 0.8) | 0.1 (0.0, 0.1) | 0.1 (0.0, 0.1) |
| Use of biomass fuels | 0.2 (-0.3, 0.8) | 0.0 (0.0, 0.1) | 0.0 (0.0, 0.1) |

**Fig 1: Stunting cases among 2-year olds attributable to individual risk factors**


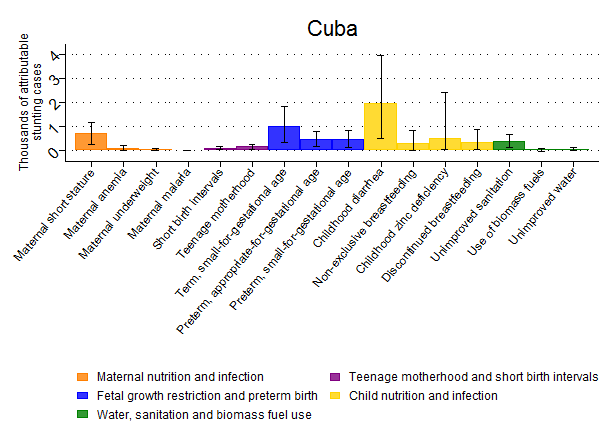


Democratic People’s Republic of Korea

**Region: East Asia/Pacific; Sub-region: Asia, East**

**Stunting prevalence among children age 2: 44.9%**

**Stunting cases among children age 2: 160,064**

**Population of children age 2: 356,363**

This country profile provides results for 2011 from the analysis presented in *Risk factors for childhood stunting in 137 developing countries: a comparative risk assessment analysis at global, regional, and country levels.*

**Table 1: Population attributable fraction (PAF in %), attributable stunting prevalence (percentage points) and number of stunting cases among 2-year olds attributable to individual risks and risk factor clusters (95% confidence intervals presented in parentheses)**

| **Description** | **PAF** | **Attributable stunting prevalence** | **Attributable stunting cases (thousands)** |
| --- | --- | --- | --- |
| **Maternal nutrition and infection** | **11.2 (10.0, 12.3)** | **5.0 (3.5, 6.6)** | **17.9 (12.3, 23.4)** |
| Maternal short stature | 9.8 (8.9, 10.5) | 4.4 (3.0, 5.6) | 15.7 (10.6, 20.1) |
| Maternal underweight | 0.7 (0.1, 1.4) | 0.3 (0.1, 0.7) | 1.1 (0.2, 2.4) |
| Maternal malaria | 0.0 (0.0, 0.0) | 0.0 (0.0, 0.0) | 0.0 (0.0, 0.0) |
| Maternal anemia | 0.8 (0.2, 1.6) | 0.4 (0.1, 0.8) | 1.3 (0.3, 2.7) |
| **Teenage motherhood and short birth intervals** | **1.3 (1.2, 1.4)** | **0.6 (0.4, 0.7)** | **2.1 (1.4, 2.7)** |
| Teenage motherhood | 0.5 (0.4, 0.5) | 0.2 (0.1, 0.3) | 0.7 (0.5, 0.9) |
| Short birth intervals | 0.8 (0.8, 0.9) | 0.4 (0.3, 0.5) | 1.3 (0.9, 1.7) |
| **Fetal growth restriction and preterm birth** | **24.3 (17.8, 29.9)** | **10.9 (7.0, 14.8)** | **38.9 (25.0, 52.9)** |
| Preterm, small-for-gestational age | 6.4 (1.3, 11.9) | 2.9 (0.6, 5.9) | 10.2 (2.1, 20.9) |
| Preterm, appropriate-for-gestational age | 5.7 (0.9, 10.7) | 2.6 (0.4, 4.9) | 9.1 (1.4, 17.6) |
| Term, small-for-gestational age | 14.2 (8.9, 19.0) | 6.4 (3.8, 9.4) | 22.7 (13.4, 33.6) |
| Low birth weight | 11.4 (9.5, 13.5) | 5.1 (3.4, 7.0) | 18.3 (12.1, 24.8) |
| **Child nutrition and infection** | **10.6 (4.1, 18.8)** | **4.8 (1.7, 9.0)** | **16.9 (6.0, 32.2)** |
| Childhood zinc deficiency | 1.3 (0.5, 2.4) | 0.6 (0.2, 1.1) | 2.2 (0.8, 4.0) |
| Childhood diarrhea | 10.0 (3.7, 18.3) | 4.5 (1.4, 8.7) | 16.0 (5.1, 30.8) |
| Non-exclusive breastfeeding | 1.4 (-0.2, 3.6) | 0.6 (-0.1, 1.7) | 2.3 (-0.3, 6.1) |
| HIV infection without HAART before 2 years | No data | No data | No data |
| Discontinued breastfeeding | 0.6 (0.0, 1.7) | 0.3 (0.0, 0.8) | 1.0 (0.1, 2.8) |
| **Water, sanitation and biomass fuel use** | **10.3 (7.4, 13.0)** | **4.6 (2.8, 6.5)** | **16.5 (9.9, 23.0)** |
| Unimproved sanitation | 7.4 (4.7, 10.1) | 3.3 (1.8, 5.0) | 11.9 (6.4, 17.7) |
| Unimproved water | 0.0 (-0.2, 0.2) | 0.0 (-0.1, 0.1) | 0.0 (-0.3, 0.3) |
| Use of biomass fuels | 3.1 (2.2, 4.1) | 1.4 (0.8, 2.0) | 4.9 (2.9, 7.1) |

**Fig 1: Stunting cases among 2-year olds attributable to individual risk factors**


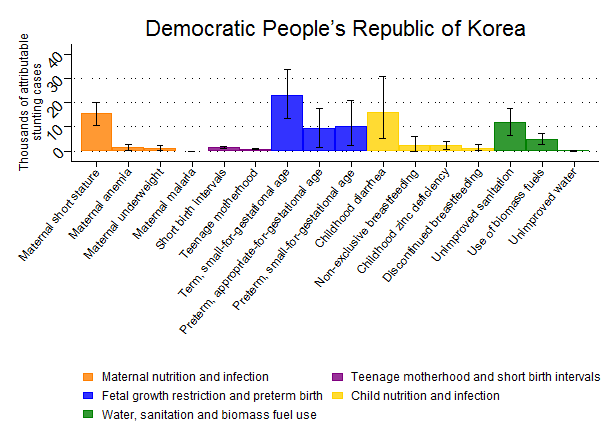


Democratic Republic of the Congo

**Region: Sub-Saharan Africa; Sub-region: Sub-Saharan Africa, Central**

**Stunting prevalence among children age 2: 47.6%**

**Stunting cases among children age 2: 1,376,896**

**Population of children age 2: 2,890,163**

This country profile provides results for 2011 from the analysis presented in *Risk factors for childhood stunting in 137 developing countries: a comparative risk assessment analysis at global, regional, and country levels.*

**Table 1: Population attributable fraction (PAF in %), attributable stunting prevalence (percentage points) and number of stunting cases among 2-year olds attributable to individual risks and risk factor clusters (95% confidence intervals presented in parentheses)**

| **Description** | **PAF** | **Attributable stunting prevalence** | **Attributable stunting cases (thousands)** |
| --- | --- | --- | --- |
| **Maternal nutrition and infection** | **13.3 (11.3, 15.4)** | **6.3 (5.0, 7.8)** | **182.9 (144, 225.3)** |
| Maternal short stature | 7.9 (7.8, 8.1) | 3.8 (3.2, 4.4) | 109.4 (93.5, 125.8) |
| Maternal underweight | 1.4 (0.7, 2.2) | 0.6 (0.3, 1.1) | 18.7 (8.8, 30.6) |
| Maternal malaria | 2.6 (1.0, 4.1) | 1.2 (0.5, 2.0) | 35.6 (13.8, 58.7) |
| Maternal anemia | 2.0 (0.6, 3.5) | 0.9 (0.3, 1.7) | 27.0 (7.5, 48.7) |
| **Teenage motherhood and short birth intervals** | **2.2 (2.1, 2.3)** | **1.0 (0.9, 1.2)** | **29.8 (25.2, 34.4)** |
| Teenage motherhood | 1.0 (0.9, 1.0) | 0.5 (0.4, 0.5) | 13.4 (11.3, 15.6) |
| Short birth intervals | 1.2 (1.1, 1.3) | 0.6 (0.5, 0.7) | 16.6 (13.9, 19.2) |
| **Fetal growth restriction and preterm birth** | **28.4 (23.4, 32.8)** | **13.5 (10.6, 16.6)** | **391.2 (306.9, 480.4)** |
| Preterm, small-for-gestational age | 4.8 (1.1, 9.1) | 2.3 (0.5, 4.5) | 65.7 (14.2, 128.6) |
| Preterm, appropriate-for-gestational age | 6.3 (0.5, 11.8) | 3.0 (0.2, 5.6) | 86.6 (7.1, 163) |
| Term, small-for-gestational age | 19.7 (15.6, 24.0) | 9.4 (7.2, 11.9) | 271.7 (208, 344.1) |
| Low birth weight | 15.6 (13.0, 18.2) | 7.4 (5.8, 9.2) | 214.8 (166.5, 265.5) |
| **Child nutrition and infection** | **16.5 (6.6, 28.2)** | **7.8 (3.1, 13.6)** | **226.6 (90.2, 394.2)** |
| Childhood zinc deficiency | 1.9 (0.8, 3.4) | 0.9 (0.4, 1.7) | 26.8 (10.6, 48.1) |
| Childhood diarrhea | 15.6 (5.8, 27.4) | 7.4 (2.8, 13.2) | 215.2 (80.2, 382.2) |
| Non-exclusive breastfeeding | 1.8 (-1.0, 5.1) | 0.9 (-0.4, 2.5) | 25.1 (-12.8, 72.7) |
| Discontinued breastfeeding | 0.8 (0.1, 2.2) | 0.4 (0.0, 1.0) | 11.0 (0.8, 29.8) |
| HIV infection without HAART before 2 years | 0.1 (0.1, 0.2) | 0.1 (0.0, 0.1) | 1.7 (0.9, 3.3) |
| **Water, sanitation and biomass fuel use** | **26.3 (22.6, 30.2)** | **12.5 (10.1, 15.2)** | **362.6 (291.8, 438.8)** |
| Unimproved sanitation | 19.8 (15.9, 23.5) | 9.4 (7.2, 11.9) | 272.7 (208, 343.1) |
| Unimproved water | 4.1 (2.5, 5.9) | 2.0 (1.2, 2.8) | 56.4 (34.2, 82.1) |
| Use of biomass fuels | 4.2 (3.0, 5.5) | 2.0 (1.4, 2.7) | 58.0 (39.6, 78.3) |

**Fig 1: Stunting cases among 2-year olds attributable to individual risk factors**


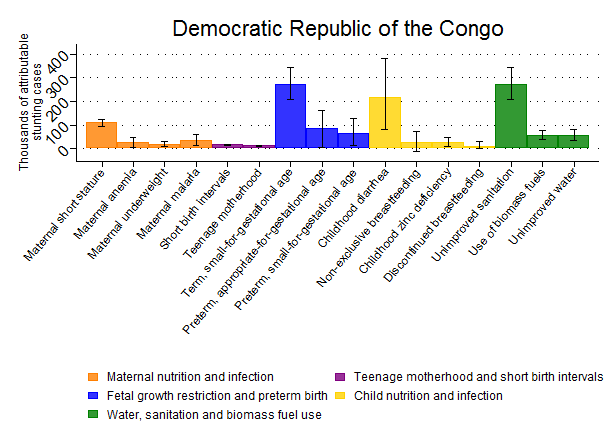


Djibouti

**Region: Sub-Saharan Africa; Sub-region: Sub-Saharan Africa, East**

**Stunting prevalence among children age 2: 27.4%**

**Stunting cases among children age 2: 6,061**

**Population of children age 2: 22,139**

This country profile provides results for 2011 from the analysis presented in *Risk factors for childhood stunting in 137 developing countries: a comparative risk assessment analysis at global, regional, and country levels.*

**Table 1: Population attributable fraction (PAF in %), attributable stunting prevalence (percentage points) and number of stunting cases among 2-year olds attributable to individual risks and risk factor clusters (95% confidence intervals presented in parentheses)**

| **Description** | **PAF** | **Attributable stunting prevalence** | **Attributable stunting cases (thousands)** |
| --- | --- | --- | --- |
| **Maternal nutrition and infection** | **14.0 (11.8, 16.8)** | **3.8 (2.5, 5.3)** | **0.9 (0.5, 1.2)** |
| Maternal short stature | 10.0 (9.1, 10.6) | 2.7 (1.8, 3.6) | 0.6 (0.4, 0.8) |
| Maternal underweight | 2.0 (0.7, 3.7) | 0.6 (0.2, 1.1) | 0.1 (0.0, 0.2) |
| Maternal malaria | 0.2 (0.1, 0.4) | 0.1 (0.0, 0.1) | 0.0 (0.0, 0.0) |
| Maternal anemia | 2.3 (0.5, 4.7) | 0.6 (0.1, 1.4) | 0.1 (0.0, 0.3) |
| **Teenage motherhood and short birth intervals** | **1.9 (1.8, 2.0)** | **0.5 (0.3, 0.7)** | **0.1 (0.1, 0.2)** |
| Teenage motherhood | 1.0 (0.9, 1.0) | 0.3 (0.2, 0.3) | 0.1 (0.0, 0.1) |
| Short birth intervals | 1.0 (0.9, 1.0) | 0.3 (0.2, 0.3) | 0.1 (0.0, 0.1) |
| **Fetal growth restriction and preterm birth** | **34.3 (29.7, 38.5)** | **9.4 (6.1, 12.8)** | **2.1 (1.3, 2.8)** |
| Preterm, small-for-gestational age | 4.1 (0.6, 8.3) | 1.1 (0.1, 2.4) | 0.3 (0.0, 0.5) |
| Preterm, appropriate-for-gestational age | 5.9 (1.0, 10.6) | 1.6 (0.3, 3.2) | 0.4 (0.1, 0.7) |
| Term, small-for-gestational age | 27.2 (22.4, 31.9) | 7.4 (4.8, 10.5) | 1.6 (1.1, 2.3) |
| Low birth weight | 27.8 (23.8, 31.7) | 7.6 (5.0, 10.6) | 1.7 (1.1, 2.3) |
| **Child nutrition and infection** | **14.5 (5.5, 25.5)** | **4.0 (1.4, 7.5)** | **0.9 (0.3, 1.7)** |
| Childhood zinc deficiency | 1.8 (0.3, 6.3) | 0.5 (0.1, 1.7) | 0.1 (0.0, 0.4) |
| Childhood diarrhea | 13.7 (5.3, 24.3) | 3.7 (1.3, 7.2) | 0.8 (0.3, 1.6) |
| Non-exclusive breastfeeding | 2.5 (0.5, 5.8) | 0.7 (0.1, 1.6) | 0.2 (0.0, 0.4) |
| HIV infection without HAART before 2 years | No data | No data | No data |
| Discontinued breastfeeding | 1.8 (0.3, 4.6) | 0.5 (0.1, 1.2) | 0.1 (0.0, 0.3) |
| **Water, sanitation and biomass fuel use** | **14.9 (10.6, 18.7)** | **4.1 (2.4, 5.9)** | **0.9 (0.5, 1.3)** |
| Unimproved sanitation | 12.9 (9.1, 16.7) | 3.5 (2.0, 5.2) | 0.8 (0.4, 1.1) |
| Unimproved water | 0.9 (0.3, 1.5) | 0.2 (0.1, 0.5) | 0.1 (0.0, 0.1) |
| Use of biomass fuels | 1.4 (-0.1, 3.0) | 0.4 (0.0, 0.9) | 0.1 (0.0, 0.2) |

**Fig 1: Stunting cases among 2-year olds attributable to individual risk factors**


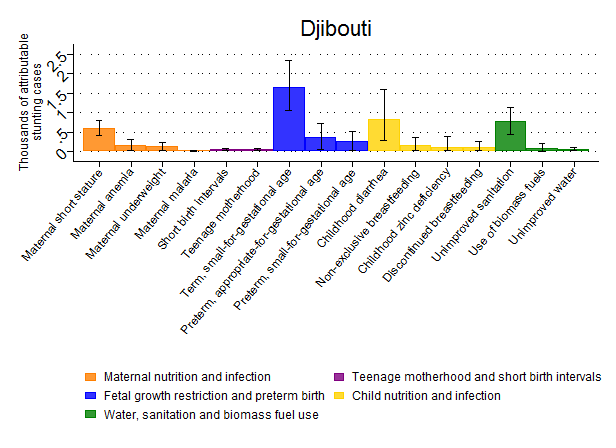


Dominica

**Region: Latin America/Caribbean; Sub-region: Caribbean**

**Stunting prevalence among children age 2: 16.8%**

**Stunting cases among children age 2: 171**

**Population of children age 2: 1,020**

This country profile provides results for 2011 from the analysis presented in *Risk factors for childhood stunting in 137 developing countries: a comparative risk assessment analysis at global, regional, and country levels.*

**Table 1: Population attributable fraction (PAF in %), attributable stunting prevalence (percentage points) and number of stunting cases among 2-year olds attributable to individual risks and risk factor clusters (95% confidence intervals presented in parentheses)**

| **Description** | **PAF** | **Attributable stunting prevalence** | **Attributable stunting cases (thousands)** |
| --- | --- | --- | --- |
| **Maternal nutrition and infection** | **7.7 (6.6, 9.2)** | **1.3 (0.4, 2.2)** | **0.0 (0.0, 0.0)** |
| Maternal short stature | 6.0 (5.8, 6.2) | 1.0 (0.4, 1.7) | 0.0 (0.0, 0.0) |
| Maternal underweight | 0.5 (0.3, 0.7) | 0.1 (0.0, 0.2) | 0.0 (0.0, 0.0) |
| Maternal malaria | 0.0 (0.0, 0.0) | 0.0 (0.0, 0.0) | 0.0 (0.0, 0.0) |
| Maternal anemia | 1.3 (0.3, 2.9) | 0.2 (0.0, 0.5) | 0.0 (0.0, 0.0) |
| **Teenage motherhood and short birth intervals** | **2.3 (2.2, 2.4)** | **0.4 (0.1, 0.6)** | **0.0 (0.0, 0.0)** |
| Teenage motherhood | 1.3 (1.3, 1.4) | 0.2 (0.1, 0.4) | 0.0 (0.0, 0.0) |
| Short birth intervals | 0.9 (0.9, 1.0) | 0.2 (0.1, 0.3) | 0.0 (0.0, 0.0) |
| **Fetal growth restriction and preterm birth** | **21.8 (15.1, 27.7)** | **3.6 (1.2, 6.6)** | **0.0 (0.0, 0.1)** |
| Preterm, small-for-gestational age | 4.4 (-1.0, 10.2) | 0.7 (-0.2, 2.0) | 0.0 (0.0, 0.0) |
| Preterm, appropriate-for-gestational age | 4.8 (-0.9, 10.7) | 0.8 (-0.1, 2.2) | 0.0 (0.0, 0.0) |
| Term, small-for-gestational age | 14.0 (9.7, 18.5) | 2.3 (0.8, 4.2) | 0.0 (0.0, 0.0) |
| Low birth weight | 15.7 (13.1, 18.4) | 2.6 (0.8, 4.6) | 0.0 (0.0, 0.0) |
| **Child nutrition and infection** | **16.3 (6.9, 25.1)** | **2.7 (0.7, 5.6)** | **0.0 (0.0, 0.1)** |
| Childhood zinc deficiency | 1.0 (0.2, 3.0) | 0.2 (0.0, 0.5) | 0.0 (0.0, 0.0) |
| Childhood diarrhea | 15.8 (6.5, 24.6) | 2.7 (0.6, 5.5) | 0.0 (0.0, 0.1) |
| Non-exclusive breastfeeding | 2.8 (0.4, 6.1) | 0.5 (0.0, 1.2) | 0.0 (0.0, 0.0) |
| HIV infection without HAART before 2 years | No data | No data | No data |
| Discontinued breastfeeding | 2.4 (0.4, 5.0) | 0.4 (0.0, 1.0) | 0.0 (0.0, 0.0) |
| **Water, sanitation and biomass fuel use** | **7.3 (5.6, 9.0)** | **1.2 (0.4, 2.2)** | **0.0 (0.0, 0.0)** |
| Unimproved sanitation | 6.6 (5.0, 8.2) | 1.1 (0.3, 2.0) | 0.0 (0.0, 0.0) |
| Unimproved water | 0.3 (0.1, 0.6) | 0.1 (0.0, 0.1) | 0.0 (0.0, 0.0) |
| Use of biomass fuels | 0.4 (-0.3, 1.2) | 0.1 (-0.1, 0.2) | 0.0 (0.0, 0.0) |

**Fig 1: Stunting cases among 2-year olds attributable to individual risk factors**


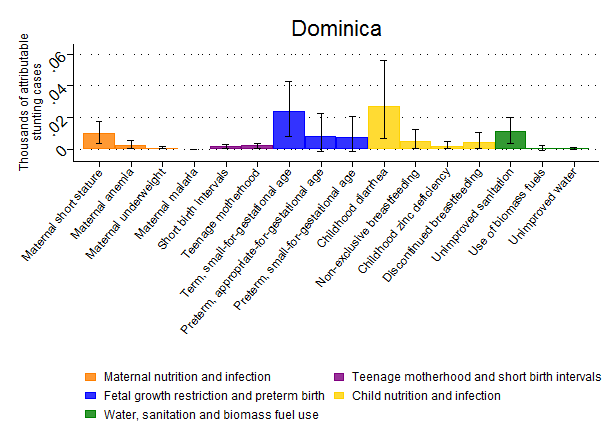


Dominican Republic

**Region: Latin America/Caribbean; Sub-region: Caribbean**

**Stunting prevalence among children age 2: 10.7%**

**Stunting cases among children age 2: 23,445**

**Population of children age 2: 219,248**

This country profile provides results for 2011 from the analysis presented in *Risk factors for childhood stunting in 137 developing countries: a comparative risk assessment analysis at global, regional, and country levels.*

**Table 1: Population attributable fraction (PAF in %), attributable stunting prevalence (percentage points) and number of stunting cases among 2-year olds attributable to individual risks and risk factor clusters (95% confidence intervals presented in parentheses)**

| **Description** | **PAF** | **Attributable stunting prevalence** | **Attributable stunting cases (thousands)** |
| --- | --- | --- | --- |
| **Maternal nutrition and infection** | **7.8 (6.9, 9.0)** | **0.8 (0.6, 1.2)** | **1.8 (1.2, 2.5)** |
| Maternal short stature | 6.4 (6.2, 6.6) | 0.7 (0.5, 0.9) | 1.5 (1.0, 2.0) |
| Maternal underweight | 0.4 (0.1, 0.8) | 0.0 (0.0, 0.1) | 0.1 (0.0, 0.2) |
| Maternal malaria | 0.0 (0.0, 0.0) | 0.0 (0.0, 0.0) | 0.0 (0.0, 0.0) |
| Maternal anemia | 1.1 (0.2, 2.2) | 0.1 (0.0, 0.2) | 0.3 (0.0, 0.5) |
| **Teenage motherhood and short birth intervals** | **2.7 (2.5, 2.8)** | **0.3 (0.2, 0.4)** | **0.6 (0.4, 0.8)** |
| Teenage motherhood | 1.7 (1.6, 1.8) | 0.2 (0.1, 0.2) | 0.4 (0.3, 0.5) |
| Short birth intervals | 1.0 (0.9, 1.0) | 0.1 (0.1, 0.1) | 0.2 (0.1, 0.3) |
| **Fetal growth restriction and preterm birth** | **22.3 (17.0, 27.1)** | **2.4 (1.5, 3.4)** | **5.2 (3.2, 7.4)** |
| Preterm, small-for-gestational age | 6.1 (1.5, 11.3) | 0.7 (0.2, 1.3) | 1.4 (0.3, 2.8) |
| Preterm, appropriate-for-gestational age | 6.0 (1.4, 10.2) | 0.6 (0.1, 1.2) | 1.4 (0.3, 2.6) |
| Term, small-for-gestational age | 12.0 (8.2, 15.6) | 1.3 (0.7, 1.9) | 2.8 (1.6, 4.1) |
| Low birth weight | 12.9 (10.7, 15.2) | 1.4 (0.9, 1.9) | 3.0 (2.0, 4.2) |
| **Child nutrition and infection** | **19.4 (8.6, 30.7)** | **2.1 (0.8, 3.6)** | **4.6 (1.8, 7.8)** |
| Childhood zinc deficiency | 3.5 (0.9, 11.3) | 0.4 (0.1, 1.4) | 0.8 (0.2, 3.0) |
| Childhood diarrhea | 17.9 (7.4, 28.5) | 1.9 (0.8, 3.3) | 4.2 (1.7, 7.2) |
| Non-exclusive breastfeeding | 3.6 (0.9, 7.2) | 0.4 (0.1, 0.8) | 0.9 (0.2, 1.8) |
| Discontinued breastfeeding | 3.0 (0.5, 6.5) | 0.3 (0.1, 0.7) | 0.7 (0.1, 1.6) |
| HIV infection without HAART before 2 years | No data | No data | No data |
| **Water, sanitation and biomass fuel use** | **7.3 (5.6, 8.8)** | **0.8 (0.5, 1.1)** | **1.7 (1.1, 2.4)** |
| Unimproved sanitation | 5.9 (4.5, 7.3) | 0.6 (0.4, 0.9) | 1.4 (0.9, 2.0) |
| Unimproved water | 1.1 (0.2, 2.0) | 0.1 (0.0, 0.2) | 0.2 (0.1, 0.5) |
| Use of biomass fuels | 0.4 (0.1, 0.8) | 0.0 (0.0, 0.1) | 0.1 (0.0, 0.2) |

**Fig 1: Stunting cases among 2-year olds attributable to individual risk factors**


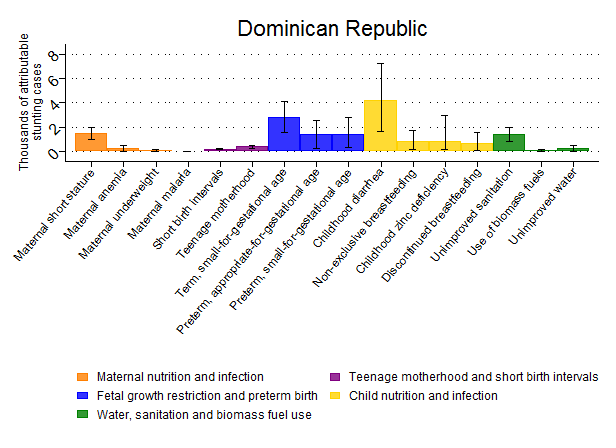


Ecuador

**Region: Latin America/Caribbean; Sub-region: Latin America, Andean**

**Stunting prevalence among children age 2: 26.6%**

**Stunting cases among children age 2: 86,963**

**Population of children age 2: 326,445**

This country profile provides results for 2011 from the analysis presented in *Risk factors for childhood stunting in 137 developing countries: a comparative risk assessment analysis at global, regional, and country levels.*

**Table 1: Population attributable fraction (PAF in %), attributable stunting prevalence (percentage points) and number of stunting cases among 2-year olds attributable to individual risks and risk factor clusters (95% confidence intervals presented in parentheses)**

| **Description** | **PAF** | **Attributable stunting prevalence** | **Attributable stunting cases (thousands)** |
| --- | --- | --- | --- |
| **Maternal nutrition and infection** | **11.9 (11.2, 12.7)** | **3.2 (2.2, 4.1)** | **10.4 (7.2, 13.5)** |
| Maternal short stature | 11.1 (10.8, 11.3) | 3.0 (2.0, 3.9) | 9.6 (6.7, 12.7) |
| Maternal underweight | 0.1 (0.0, 0.3) | 0.0 (0.0, 0.1) | 0.1 (0.0, 0.3) |
| Maternal malaria | 0.0 (0.0, 0.0) | 0.0 (0.0, 0.0) | 0.0 (0.0, 0.0) |
| Maternal anemia | 0.8 (0.2, 1.6) | 0.2 (0.0, 0.5) | 0.7 (0.1, 1.5) |
| **Teenage motherhood and short birth intervals** | **2.0 (1.9, 2.0)** | **0.5 (0.4, 0.7)** | **1.7 (1.2, 2.2)** |
| Teenage motherhood | 1.1 (1.0, 1.2) | 0.3 (0.2, 0.4) | 0.9 (0.6, 1.2) |
| Short birth intervals | 0.9 (0.8, 1.0) | 0.2 (0.2, 0.3) | 0.8 (0.5, 1.0) |
| **Fetal growth restriction and preterm birth** | **15.8 (11.3, 19.9)** | **4.2 (2.6, 6.1)** | **13.8 (8.5, 19.8)** |
| Preterm, small-for-gestational age | 3.2 (0.2, 6.5) | 0.9 (0.0, 1.8) | 2.8 (0.1, 5.9) |
| Preterm, appropriate-for-gestational age | 3.2 (0.1, 6.1) | 0.8 (0.0, 1.7) | 2.8 (0.1, 5.7) |
| Term, small-for-gestational age | 10.2 (6.8, 14.0) | 2.7 (1.5, 4.0) | 8.9 (5.0, 13.2) |
| Low birth weight | 10.5 (8.7, 12.4) | 2.8 (1.8, 3.8) | 9.1 (6.0, 12.5) |
| **Child nutrition and infection** | **17.6 (7.1, 28.1)** | **4.7 (1.8, 8.1)** | **15.3 (5.9, 26.5)** |
| Childhood zinc deficiency | 0.4 (0.1, 0.8) | 0.1 (0.0, 0.2) | 0.3 (0.1, 0.7) |
| Childhood diarrhea | 17.5 (7.0, 27.9) | 4.6 (1.8, 8.1) | 15.2 (5.7, 26.3) |
| Non-exclusive breastfeeding | 1.6 (-1.6, 4.9) | 0.4 (-0.4, 1.4) | 1.4 (-1.4, 4.6) |
| HIV infection without HAART before 2 years | No data | No data | No data |
| Discontinued breastfeeding | 1.7 (0.2, 4.3) | 0.4 (0.0, 1.1) | 1.4 (0.2, 3.6) |
| **Water, sanitation and biomass fuel use** | **4.5 (2.8, 6.1)** | **1.2 (0.6, 1.8)** | **3.9 (2.1, 5.9)** |
| Unimproved sanitation | 3.6 (2.0, 5.1) | 0.9 (0.5, 1.5) | 3.1 (1.5, 4.9) |
| Unimproved water | 0.8 (0.2, 1.4) | 0.2 (0.1, 0.4) | 0.7 (0.2, 1.3) |
| Use of biomass fuels | 0.2 (-0.1, 0.4) | 0.0 (0.0, 0.1) | 0.1 (-0.1, 0.4) |

**Fig 1: Stunting cases among 2-year olds attributable to individual risk factors**


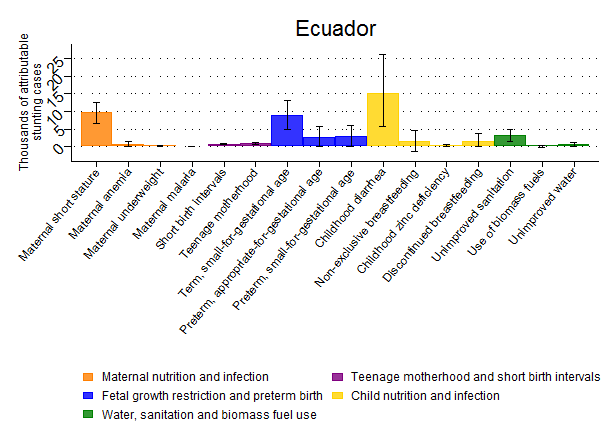


Egypt

**Region: North Africa/Middle East; Sub-region: North Africa / Middle East**

**Stunting prevalence among children age 2: 23.4%**

**Stunting cases among children age 2: 522,390**

**Population of children age 2: 2,228,587**

This country profile provides results for 2011 from the analysis presented in *Risk factors for childhood stunting in 137 developing countries: a comparative risk assessment analysis at global, regional, and country levels.*

**Table 1: Population attributable fraction (PAF in %), attributable stunting prevalence (percentage points) and number of stunting cases among 2-year olds attributable to individual risks and risk factor clusters (95% confidence intervals presented in parentheses)**

| **Description** | **PAF** | **Attributable stunting prevalence** | **Attributable stunting cases (thousands)** |
| --- | --- | --- | --- |
| **Maternal nutrition and infection** | **7.5 (6.8, 8.3)** | **1.8 (1.2, 2.4)** | **39.0 (26.4, 52.4)** |
| Maternal short stature | 6.2 (6.1, 6.4) | 1.5 (1.0, 1.9) | 32.6 (22.3, 42.9) |
| Maternal underweight | 0.1 (0.0, 0.2) | 0.0 (0.0, 0.1) | 0.5 (0.1, 1.1) |
| Maternal malaria | 0.4 (0.1, 0.6) | 0.1 (0.0, 0.2) | 2.0 (0.6, 3.6) |
| Maternal anemia | 0.8 (0.2, 1.6) | 0.2 (0.0, 0.4) | 4.4 (1.0, 9.0) |
| **Teenage motherhood and short birth intervals** | **1.3 (1.3, 1.4)** | **0.3 (0.2, 0.4)** | **6.9 (4.7, 9.2)** |
| Teenage motherhood | 0.6 (0.5, 0.6) | 0.1 (0.1, 0.2) | 3.0 (2.0, 4.1) |
| Short birth intervals | 0.8 (0.7, 0.8) | 0.2 (0.1, 0.2) | 4.0 (2.7, 5.3) |
| **Fetal growth restriction and preterm birth** | **17.2 (13.1, 21.0)** | **4.0 (2.5, 5.7)** | **89.8 (56.1, 127.1)** |
| Preterm, small-for-gestational age | 3.5 (1.3, 5.9) | 0.8 (0.3, 1.5) | 18.1 (6.7, 33.2) |
| Preterm, appropriate-for-gestational age | 4.6 (1.9, 7.8) | 1.1 (0.4, 1.9) | 24.1 (8.4, 43.3) |
| Term, small-for-gestational age | 10.1 (6.5, 13.6) | 2.4 (1.3, 3.5) | 52.6 (29.7, 79.0) |
| Low birth weight | 10.4 (8.6, 12.2) | 2.4 (1.6, 3.4) | 54.2 (35.0, 74.9) |
| **Child nutrition and infection** | **11.8 (4.1, 21.2)** | **2.8 (0.9, 5.6)** | **61.9 (21.0, 125.4)** |
| Childhood zinc deficiency | 0.9 (0.3, 2.1) | 0.2 (0.1, 0.5) | 4.6 (1.4, 11.9) |
| Childhood diarrhea | 11.5 (3.7, 20.6) | 2.7 (0.9, 5.4) | 59.9 (19.2, 120.6) |
| Non-exclusive breastfeeding | 1.1 (-1.0, 3.7) | 0.3 (-0.2, 0.9) | 6.0 (-5.1, 19.0) |
| Discontinued breastfeeding | 0.6 (0.0, 1.6) | 0.1 (0.0, 0.4) | 2.9 (0.1, 8.4) |
| HIV infection without HAART before 2 years | No data | No data | No data |
| **Water, sanitation and biomass fuel use** | **1.6 (1.1, 2.2)** | **0.4 (0.2, 0.6)** | **8.5 (4.8, 12.7)** |
| Unimproved sanitation | 1.5 (0.9, 2.1) | 0.3 (0.2, 0.5) | 7.6 (4.1, 11.5) |
| Unimproved water | 0.2 (0.1, 0.3) | 0.0 (0.0, 0.1) | 0.9 (0.4, 1.7) |
| Use of biomass fuels | 0.0 (-0.2, 0.2) | 0.0 (0.0, 0.0) | 0.0 (-1.0, 0.8) |

**Fig 1: Stunting cases among 2-year olds attributable to individual risk factors**


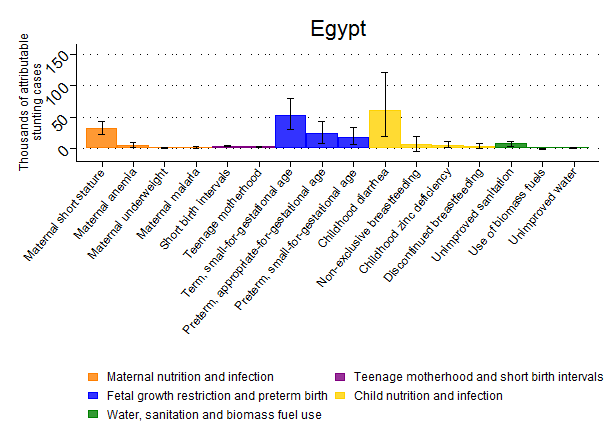


El Salvador

**Region: Latin America/Caribbean; Sub-region: Latin America, Central**

**Stunting prevalence among children age 2: 22.5%**

**Stunting cases among children age 2: 24,919**

**Population of children age 2: 110,987**

This country profile provides results for 2011 from the analysis presented in *Risk factors for childhood stunting in 137 developing countries: a comparative risk assessment analysis at global, regional, and country levels.*

**Table 1: Population attributable fraction (PAF in %), attributable stunting prevalence (percentage points) and number of stunting cases among 2-year olds attributable to individual risks and risk factor clusters (95% confidence intervals presented in parentheses)**

| **Description** | **PAF** | **Attributable stunting prevalence** | **Attributable stunting cases (thousands)** |
| --- | --- | --- | --- |
| **Maternal nutrition and infection** | **12.0 (11.1, 13.3)** | **2.7 (2.1, 3.3)** | **3.0 (2.4, 3.6)** |
| Maternal short stature | 10.7 (10.4, 10.9) | 2.4 (1.9, 2.8) | 2.7 (2.2, 3.2) |
| Maternal underweight | 0.3 (0.1, 0.7) | 0.1 (0.0, 0.2) | 0.1 (0.0, 0.2) |
| Maternal malaria | 0.0 (0.0, 0.0) | 0.0 (0.0, 0.0) | 0.0 (0.0, 0.0) |
| Maternal anemia | 1.2 (0.2, 2.5) | 0.3 (0.1, 0.6) | 0.3 (0.1, 0.6) |
| **Teenage motherhood and short birth intervals** | **2.5 (2.4, 2.6)** | **0.6 (0.4, 0.7)** | **0.6 (0.5, 0.7)** |
| Teenage motherhood | 1.5 (1.4, 1.6) | 0.3 (0.3, 0.4) | 0.4 (0.3, 0.4) |
| Short birth intervals | 1.0 (0.9, 1.1) | 0.2 (0.2, 0.3) | 0.2 (0.2, 0.3) |
| **Fetal growth restriction and preterm birth** | **24.6 (18.0, 30.5)** | **5.5 (3.8, 7.4)** | **6.1 (4.2, 8.2)** |
| Preterm, small-for-gestational age | 7.0 (0.4, 14.0) | 1.6 (0.1, 3.2) | 1.7 (0.1, 3.6) |
| Preterm, appropriate-for-gestational age | 6.7 (0.0, 13.1) | 1.5 (0.0, 3.0) | 1.7 (0.0, 3.3) |
| Term, small-for-gestational age | 13.1 (9.1, 17.4) | 2.9 (1.9, 4.1) | 3.3 (2.2, 4.6) |
| Low birth weight | 15.9 (13.3, 18.5) | 3.6 (2.7, 4.5) | 4.0 (3.0, 5.0) |
| **Child nutrition and infection** | **17.9 (8.0, 28.2)** | **4.0 (1.8, 6.5)** | **4.5 (2.0, 7.2)** |
| Childhood zinc deficiency | 1.5 (0.6, 2.6) | 0.3 (0.1, 0.6) | 0.4 (0.1, 0.7) |
| Childhood diarrhea | 17.3 (7.6, 27.7) | 3.9 (1.7, 6.3) | 4.3 (1.9, 7.0) |
| Non-exclusive breastfeeding | 2.8 (-0.1, 6.3) | 0.6 (0.0, 1.4) | 0.7 (0.0, 1.6) |
| HIV infection without HAART before 2 years | No data | No data | No data |
| Discontinued breastfeeding | 1.7 (0.2, 3.9) | 0.4 (0.0, 0.9) | 0.4 (0.1, 1.0) |
| **Water, sanitation and biomass fuel use** | **10.9 (8.6, 13.1)** | **2.4 (1.8, 3.2)** | **2.7 (2.0, 3.5)** |
| Unimproved sanitation | 9.1 (6.8, 11.1) | 2.0 (1.4, 2.7) | 2.3 (1.6, 3.0) |
| Unimproved water | 0.8 (0.3, 1.3) | 0.2 (0.1, 0.3) | 0.2 (0.1, 0.3) |
| Use of biomass fuels | 1.2 (0.5, 2.1) | 0.3 (0.1, 0.5) | 0.3 (0.1, 0.5) |

**Fig 1: Stunting cases among 2-year olds attributable to individual risk factors**


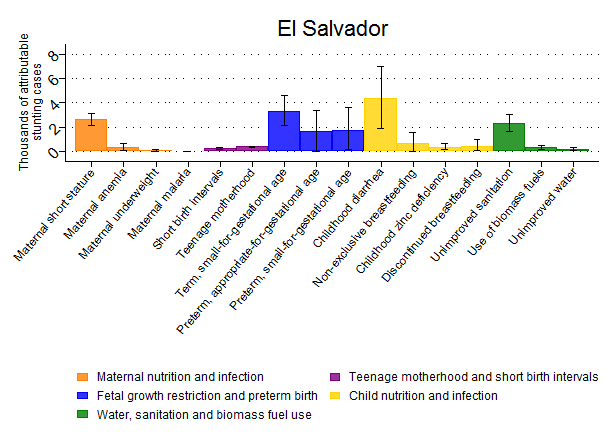


Equatorial Guinea

**Region: Sub-Saharan Africa; Sub-region: Sub-Saharan Africa, Central**

**Stunting prevalence among children age 2: 30.8%**

**Stunting cases among children age 2: 8,163**

**Population of children age 2: 26,486**

This country profile provides results for 2011 from the analysis presented in *Risk factors for childhood stunting in 137 developing countries: a comparative risk assessment analysis at global, regional, and country levels.*

**Table 1: Population attributable fraction (PAF in %), attributable stunting prevalence (percentage points) and number of stunting cases among 2-year olds attributable to individual risks and risk factor clusters (95% confidence intervals presented in parentheses)**

| **Description** | **PAF** | **Attributable stunting prevalence** | **Attributable stunting cases (thousands)** |
| --- | --- | --- | --- |
| **Maternal nutrition and infection** | **14.0 (11.9, 16.1)** | **4.3 (2.8, 5.9)** | **1.1 (0.7, 1.6)** |
| Maternal short stature | 9.6 (8.7, 10.3) | 3.0 (1.9, 3.9) | 0.8 (0.5, 1.0) |
| Maternal underweight | 1.2 (0.3, 2.2) | 0.4 (0.1, 0.8) | 0.1 (0.0, 0.2) |
| Maternal malaria | 1.1 (0.4, 1.9) | 0.3 (0.1, 0.6) | 0.1 (0.0, 0.2) |
| Maternal anemia | 2.7 (0.8, 4.6) | 0.8 (0.2, 1.5) | 0.2 (0.1, 0.4) |
| **Teenage motherhood and short birth intervals** | **2.3 (2.2, 2.4)** | **0.7 (0.5, 0.9)** | **0.2 (0.1, 0.3)** |
| Teenage motherhood | 1.4 (1.3, 1.5) | 0.4 (0.3, 0.6) | 0.1 (0.1, 0.2) |
| Short birth intervals | 1.0 (0.9, 1.0) | 0.3 (0.2, 0.4) | 0.1 (0.1, 0.1) |
| **Fetal growth restriction and preterm birth** | **31.8 (27.1, 36.3)** | **9.8 (6.2, 13.6)** | **2.6 (1.6, 3.6)** |
| Preterm, small-for-gestational age | 5.9 (1.3, 10.8) | 1.8 (0.4, 3.6) | 0.5 (0.1, 1.0) |
| Preterm, appropriate-for-gestational age | 8.1 (2.6, 13.7) | 2.5 (0.7, 4.6) | 0.7 (0.2, 1.2) |
| Term, small-for-gestational age | 21.1 (17.4, 25.1) | 6.5 (4.1, 9.1) | 1.7 (1.1, 2.4) |
| Low birth weight | 19.8 (16.6, 22.9) | 6.1 (3.8, 8.4) | 1.6 (1.0, 2.2) |
| **Child nutrition and infection** | **19.4 (7.6, 33.3)** | **6.0 (2.2, 11.1)** | **1.6 (0.6, 2.9)** |
| Childhood zinc deficiency | 1.4 (0.5, 2.8) | 0.4 (0.1, 0.9) | 0.1 (0.0, 0.2) |
| Childhood diarrhea | 18.8 (7.0, 32.6) | 5.8 (2.0, 10.9) | 1.5 (0.5, 2.9) |
| Non-exclusive breastfeeding | 2.5 (-0.9, 6.6) | 0.8 (-0.3, 2.1) | 0.2 (-0.1, 0.6) |
| HIV infection without HAART before 2 years | 1.5 (0.5, 5.5) | 0.5 (0.1, 1.8) | 0.1 (0.0, 0.5) |
| Discontinued breastfeeding | 1.9 (0.2, 5.1) | 0.6 (0.1, 1.6) | 0.2 (0.0, 0.4) |
| **Water, sanitation and biomass fuel use** | **20.2 (17.5, 22.9)** | **6.2 (3.9, 8.5)** | **1.7 (1.0, 2.3)** |
| Unimproved sanitation | 13.7 (11.3, 16.0) | 4.2 (2.6, 5.9) | 1.1 (0.7, 1.6) |
| Unimproved water | 3.0 (1.6, 4.5) | 0.9 (0.4, 1.5) | 0.2 (0.1, 0.4) |
| Use of biomass fuels | 4.7 (3.3, 6.1) | 1.4 (0.8, 2.2) | 0.4 (0.2, 0.6) |

**Fig 1: Stunting cases among 2-year olds attributable to individual risk factors**


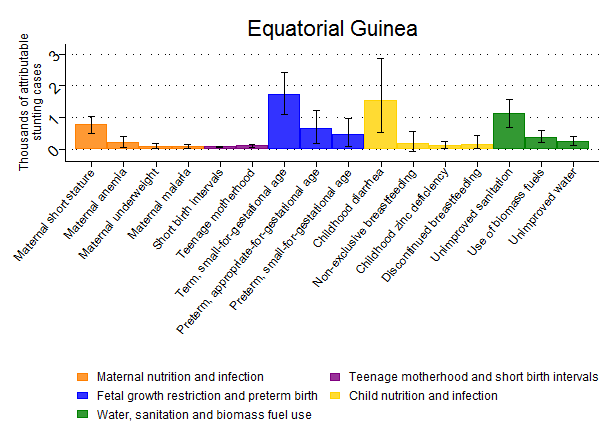


Eritrea

**Region: Sub-Saharan Africa; Sub-region: Sub-Saharan Africa, East**

**Stunting prevalence among children age 2: 60.2%**

**Stunting cases among children age 2: 102,948**

**Population of children age 2: 171,014**

This country profile provides results for 2011 from the analysis presented in *Risk factors for childhood stunting in 137 developing countries: a comparative risk assessment analysis at global, regional, and country levels.*

**Table 1: Population attributable fraction (PAF in %), attributable stunting prevalence (percentage points) and number of stunting cases among 2-year olds attributable to individual risks and risk factor clusters (95% confidence intervals presented in parentheses)**

| **Description** | **PAF** | **Attributable stunting prevalence** | **Attributable stunting cases (thousands)** |
| --- | --- | --- | --- |
| **Maternal nutrition and infection** | **11.6 (9.7, 13.6)** | **7.0 (4.7, 9.5)** | **11.9 (8.1, 16.2)** |
| Maternal short stature | 7.2 (7.0, 7.5) | 4.4 (3.1, 5.6) | 7.4 (5.2, 9.5) |
| Maternal underweight | 2.4 (1.3, 3.8) | 1.4 (0.7, 2.4) | 2.4 (1.2, 4.1) |
| Maternal malaria | 0.3 (0.1, 0.5) | 0.2 (0.1, 0.3) | 0.3 (0.1, 0.6) |
| Maternal anemia | 2.0 (0.5, 4.0) | 1.2 (0.3, 2.6) | 2.1 (0.5, 4.4) |
| **Teenage motherhood and short birth intervals** | **1.9 (1.8, 2.0)** | **1.1 (0.8, 1.5)** | **2.0 (1.4, 2.5)** |
| Teenage motherhood | 1.0 (0.9, 1.0) | 0.6 (0.4, 0.7) | 1.0 (0.7, 1.3) |
| Short birth intervals | 1.0 (0.9, 1.0) | 0.6 (0.4, 0.7) | 1.0 (0.7, 1.3) |
| **Fetal growth restriction and preterm birth** | **29.2 (24.1, 34.0)** | **17.6 (11.9, 23.7)** | **30.1 (20.4, 40.5)** |
| Preterm, small-for-gestational age | 4.7 (1.2, 9.2) | 2.9 (0.7, 5.7) | 4.9 (1.2, 9.8) |
| Preterm, appropriate-for-gestational age | 6.4 (0.9, 11.9) | 3.8 (0.5, 7.8) | 6.6 (0.9, 13.3) |
| Term, small-for-gestational age | 20.6 (16.4, 25.2) | 12.4 (8.1, 17.2) | 21.2 (13.8, 29.3) |
| Low birth weight | 21.3 (18.0, 24.7) | 12.8 (8.5, 17.1) | 22.0 (14.6, 29.2) |
| **Child nutrition and infection** | **12.8 (4.8, 22.5)** | **7.7 (2.7, 14.7)** | **13.2 (4.7, 25.1)** |
| Childhood zinc deficiency | 0.6 (0.2, 1.0) | 0.3 (0.1, 0.6) | 0.6 (0.2, 1.1) |
| Childhood diarrhea | 12.6 (4.7, 22.3) | 7.6 (2.6, 14.5) | 13.0 (4.5, 24.8) |
| Non-exclusive breastfeeding | 0.9 (-1.6, 3.5) | 0.6 (-1.0, 2.3) | 1.0 (-1.7, 3.9) |
| Discontinued breastfeeding | 0.6 (0.0, 1.7) | 0.4 (0.0, 1.1) | 0.6 (0.0, 1.8) |
| HIV infection without HAART before 2 years | 0.0 (0.0, 0.1) | 0.0 (0.0, 0.0) | 0.0 (0.0, 0.1) |
| **Water, sanitation and biomass fuel use** | **29.0 (26.6, 31.5)** | **17.5 (12.1, 22.8)** | **29.9 (20.7, 38.9)** |
| Unimproved sanitation | 23.7 (21.5, 26.1) | 14.3 (9.7, 18.7) | 24.4 (16.6, 31.9) |
| Unimproved water | 2.8 (1.5, 4.2) | 1.7 (0.9, 2.8) | 2.9 (1.5, 4.7) |
| Use of biomass fuels | 4.3 (2.8, 5.8) | 2.6 (1.5, 3.8) | 4.4 (2.6, 6.6) |

**Fig 1: Stunting cases among 2-year olds attributable to individual risk factors**


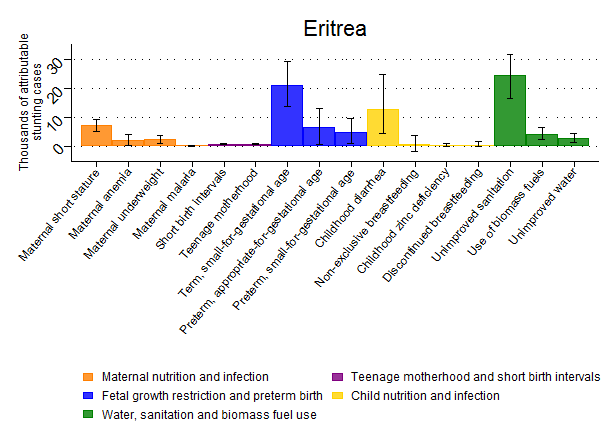


Ethiopia

**Region: Sub-Saharan Africa; Sub-region: Sub-Saharan Africa, East**

**Stunting prevalence among children age 2: 59.2%**

**Stunting cases among children age 2: 1,802,418**

**Population of children age 2: 3,046,317**

This country profile provides results for 2011 from the analysis presented in *Risk factors for childhood stunting in 137 developing countries: a comparative risk assessment analysis at global, regional, and country levels.*

**Table 1: Population attributable fraction (PAF in %), attributable stunting prevalence (percentage points) and number of stunting cases among 2-year olds attributable to individual risks and risk factor clusters (95% confidence intervals presented in parentheses)**

| **Description** | **PAF** | **Attributable stunting prevalence** | **Attributable stunting cases (thousands)** |
| --- | --- | --- | --- |
| **Maternal nutrition and infection** | **12.4 (10.6, 14.5)** | **7.4 (6.0, 9.1)** | **224.2 (182.2, 276)** |
| Maternal short stature | 7.9 (7.7, 8.1) | 4.7 (4.0, 5.3) | 142.2 (122.9, 162.1) |
| Maternal underweight | 2.8 (1.6, 4.4) | 1.7 (0.9, 2.6) | 51.1 (26.8, 80.0) |
| Maternal malaria | 0.3 (0.1, 0.5) | 0.2 (0.1, 0.3) | 5.6 (2.0, 9.9) |
| Maternal anemia | 1.9 (0.5, 3.3) | 1.1 (0.3, 2.0) | 33.5 (9.3, 60.8) |
| **Teenage motherhood and short birth intervals** | **2.0 (1.9, 2.1)** | **1.2 (1.0, 1.3)** | **35.4 (30.4, 40.7)** |
| Teenage motherhood | 0.9 (0.8, 1.0) | 0.5 (0.5, 0.6) | 16.3 (13.9, 19.1) |
| Short birth intervals | 1.1 (1.0, 1.1) | 0.6 (0.5, 0.7) | 19.3 (16.4, 22.4) |
| **Fetal growth restriction and preterm birth** | **33.8 (29.9, 37.7)** | **20.0 (16.8, 23.4)** | **609.2 (510.4, 712.7)** |
| Preterm, small-for-gestational age | 3.6 (1.9, 5.7) | 2.2 (1.1, 3.5) | 65.6 (34.1, 106) |
| Preterm, appropriate-for-gestational age | 5.0 (3.1, 7.4) | 3.0 (1.9, 4.4) | 90.8 (57.1, 133.1) |
| Term, small-for-gestational age | 27.7 (23.3, 32.2) | 16.4 (13.2, 19.7) | 498.2 (403.4, 600.5) |
| Low birth weight | 28.1 (24.0, 32.0) | 16.6 (13.5, 20.3) | 506 (411.5, 617.4) |
| **Child nutrition and infection** | **14.0 (5.5, 24.6)** | **8.3 (3.2, 15.0)** | **253.1 (97.6, 457.5)** |
| Childhood zinc deficiency | 0.3 (0.1, 0.5) | 0.2 (0.1, 0.3) | 5.8 (2.3, 9.8) |
| Childhood diarrhea | 13.9 (5.4, 24.6) | 8.2 (3.1, 15.0) | 250.6 (95.9, 455.6) |
| Non-exclusive breastfeeding | 1.3 (-1.5, 4.2) | 0.8 (-0.8, 2.5) | 23.9 (-24.7, 77.6) |
| HIV infection without HAART before 2 years | 0.1 (0.1, 0.2) | 0.1 (0.0, 0.1) | 2.2 (1.3, 3.8) |
| Discontinued breastfeeding | 0.9 (0.1, 2.5) | 0.6 (0.0, 1.5) | 16.8 (1.1, 45.9) |
| **Water, sanitation and biomass fuel use** | **31.0 (28.2, 33.9)** | **18.3 (15.5, 21.3)** | **558.4 (473, 650)** |
| Unimproved sanitation | 21.7 (19.4, 24.0) | 12.8 (10.8, 15.1) | 390.4 (328.7, 460.5) |
| Unimproved water | 4.3 (2.8, 6.1) | 2.6 (1.6, 3.7) | 78.3 (49.3, 111.7) |
| Use of biomass fuels | 7.9 (5.7, 10.1) | 4.7 (3.3, 6.2) | 142.3 (101, 189.2) |

**Fig 1: Stunting cases among 2-year olds attributable to individual risk factors**


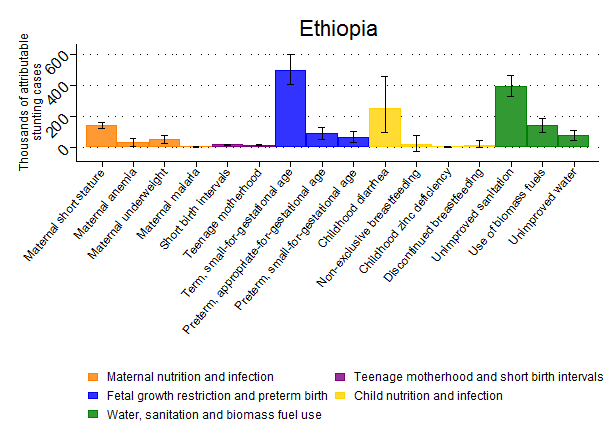


Fiji

**Region: East Asia/Pacific; Sub-region: Oceania**

**Stunting prevalence among children age 2: 21.7%**

**Stunting cases among children age 2: 4,011**

**Population of children age 2: 18,489**

This country profile provides results for 2011 from the analysis presented in *Risk factors for childhood stunting in 137 developing countries: a comparative risk assessment analysis at global, regional, and country levels.*

**Table 1: Population attributable fraction (PAF in %), attributable stunting prevalence (percentage points) and number of stunting cases among 2-year olds attributable to individual risks and risk factor clusters (95% confidence intervals presented in parentheses)**

| **Description** | **PAF** | **Attributable stunting prevalence** | **Attributable stunting cases (thousands)** |
| --- | --- | --- | --- |
| **Maternal nutrition and infection** | **5.1 (4.1, 6.5)** | **1.1 (0.3, 2.1)** | **0.2 (0.1, 0.4)** |
| Maternal short stature | 3.5 (3.4, 3.7) | 0.8 (0.2, 1.3) | 0.1 (0.0, 0.2) |
| Maternal underweight | 0.3 (0.1, 0.6) | 0.1 (0.0, 0.2) | 0.0 (0.0, 0.0) |
| Maternal malaria | 0.0 (0.0, 0.0) | 0.0 (0.0, 0.0) | 0.0 (0.0, 0.0) |
| Maternal anemia | 1.4 (0.4, 2.7) | 0.3 (0.0, 0.7) | 0.1 (0.0, 0.1) |
| **Teenage motherhood and short birth intervals** | **1.3 (1.2, 1.4)** | **0.3 (0.1, 0.5)** | **0.1 (0.0, 0.1)** |
| Teenage motherhood | 0.5 (0.4, 0.5) | 0.1 (0.0, 0.2) | 0.0 (0.0, 0.0) |
| Short birth intervals | 0.8 (0.8, 0.9) | 0.2 (0.0, 0.3) | 0.0 (0.0, 0.1) |
| **Fetal growth restriction and preterm birth** | **25.2 (19.4, 30.5)** | **5.5 (1.4, 10.1)** | **1.0 (0.3, 1.9)** |
| Preterm, small-for-gestational age | 5.6 (1.0, 10.4) | 1.2 (0.1, 2.8) | 0.2 (0.0, 0.5) |
| Preterm, appropriate-for-gestational age | 5.1 (1.3, 9.3) | 1.1 (0.1, 2.5) | 0.2 (0.0, 0.5) |
| Term, small-for-gestational age | 16.5 (11.4, 21.7) | 3.6 (0.9, 7.0) | 0.7 (0.2, 1.3) |
| Low birth weight | 14.8 (12.3, 17.3) | 3.2 (0.8, 6.1) | 0.6 (0.2, 1.1) |
| **Child nutrition and infection** | **11.3 (4.1, 19.9)** | **2.5 (0.5, 5.5)** | **0.5 (0.1, 1.0)** |
| Childhood zinc deficiency | 2.9 (0.2, 11.6) | 0.7 (0.0, 2.8) | 0.1 (0.0, 0.5) |
| Childhood diarrhea | 10.9 (4.2, 18.6) | 2.4 (0.5, 5.2) | 0.4 (0.1, 1.0) |
| Non-exclusive breastfeeding | 1.3 (-1.0, 3.8) | 0.3 (-0.2, 0.9) | 0.1 (0.0, 0.2) |
| Discontinued breastfeeding | 1.0 (0.1, 2.6) | 0.2 (0.0, 0.6) | 0.0 (0.0, 0.1) |
| HIV infection without HAART before 2 years | No data | No data | No data |
| **Water, sanitation and biomass fuel use** | **7.2 (2.9, 11.0)** | **1.6 (0.3, 3.4)** | **0.3 (0.1, 0.6)** |
| Unimproved sanitation | 4.9 (0.8, 8.7) | 1.1 (0.1, 2.5) | 0.2 (0.0, 0.5) |
| Unimproved water | 0.4 (0.1, 0.7) | 0.1 (0.0, 0.2) | 0.0 (0.0, 0.0) |
| Use of biomass fuels | 2.0 (1.3, 2.9) | 0.4 (0.1, 0.9) | 0.1 (0.0, 0.2) |

**Fig 1: Stunting cases among 2-year olds attributable to individual risk factors**


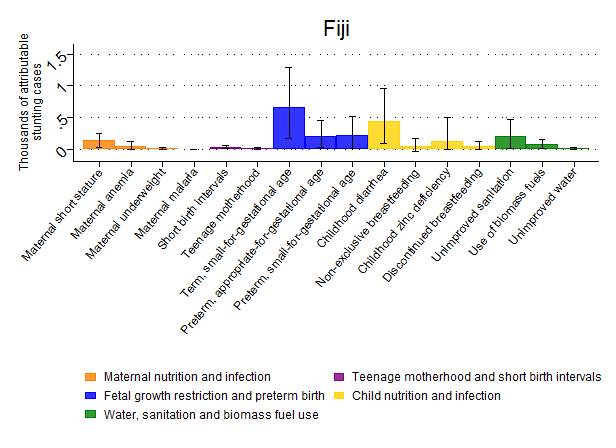


Gabon

**Region: Sub-Saharan Africa; Sub-region: Sub-Saharan Africa, Central**

**Stunting prevalence among children age 2: 24.1%**

**Stunting cases among children age 2: 11,602**

**Population of children age 2: 48,192**

This country profile provides results for 2011 from the analysis presented in *Risk factors for childhood stunting in 137 developing countries: a comparative risk assessment analysis at global, regional, and country levels.*

**Table 1: Population attributable fraction (PAF in %), attributable stunting prevalence (percentage points) and number of stunting cases among 2-year olds attributable to individual risks and risk factor clusters (95% confidence intervals presented in parentheses)**

| **Description** | **PAF** | **Attributable stunting prevalence** | **Attributable stunting cases (thousands)** |
| --- | --- | --- | --- |
| **Maternal nutrition and infection** | **10.1 (7.9, 12.4)** | **2.4 (1.4, 3.7)** | **1.2 (0.7, 1.8)** |
| Maternal short stature | 5.2 (5.0, 5.4) | 1.3 (0.7, 1.8) | 0.6 (0.3, 0.9) |
| Maternal underweight | 1.1 (0.4, 1.9) | 0.3 (0.1, 0.5) | 0.1 (0.0, 0.3) |
| Maternal malaria | 1.0 (0.4, 1.7) | 0.2 (0.1, 0.5) | 0.1 (0.0, 0.2) |
| Maternal anemia | 3.2 (1.0, 5.4) | 0.8 (0.2, 1.5) | 0.4 (0.1, 0.7) |
| **Teenage motherhood and short birth intervals** | **2.7 (2.5, 2.9)** | **0.7 (0.4, 0.9)** | **0.3 (0.2, 0.5)** |
| Teenage motherhood | 1.8 (1.7, 2.0) | 0.4 (0.3, 0.6) | 0.2 (0.1, 0.3) |
| Short birth intervals | 0.9 (0.8, 1.0) | 0.2 (0.1, 0.3) | 0.1 (0.1, 0.1) |
| **Fetal growth restriction and preterm birth** | **31.4 (26.7, 35.9)** | **7.6 (4.4, 10.9)** | **3.6 (2.1, 5.2)** |
| Preterm, small-for-gestational age | 5.9 (1.4, 10.6) | 1.4 (0.3, 3.0) | 0.7 (0.1, 1.5) |
| Preterm, appropriate-for-gestational age | 8.3 (3.0, 13.9) | 2.0 (0.8, 3.8) | 1.0 (0.4, 1.9) |
| Term, small-for-gestational age | 20.4 (16.2, 24.8) | 4.9 (2.7, 7.2) | 2.4 (1.3, 3.5) |
| Low birth weight | 21.6 (18.2, 24.9) | 5.2 (3.0, 7.7) | 2.5 (1.5, 3.7) |
| **Child nutrition and infection** | **12.4 (4.7, 22.2)** | **3.0 (0.9, 5.8)** | **1.4 (0.5, 2.8)** |
| Childhood zinc deficiency | 0.9 (0.3, 2.2) | 0.2 (0.1, 0.6) | 0.1 (0.0, 0.3) |
| Childhood diarrhea | 12.0 (4.4, 21.8) | 2.9 (0.9, 5.7) | 1.4 (0.4, 2.7) |
| Non-exclusive breastfeeding | 2.1 (0.3, 4.9) | 0.5 (0.1, 1.2) | 0.2 (0.0, 0.6) |
| Discontinued breastfeeding | 1.6 (0.2, 4.0) | 0.4 (0.0, 1.1) | 0.2 (0.0, 0.5) |
| HIV infection without HAART before 2 years | 1.2 (0.2, 6.0) | 0.3 (0.0, 1.5) | 0.1 (0.0, 0.7) |
| **Water, sanitation and biomass fuel use** | **19.7 (16.9, 22.4)** | **4.7 (2.7, 7.0)** | **2.3 (1.3, 3.4)** |
| Unimproved sanitation | 17.5 (14.8, 20.2) | 4.2 (2.5, 6.2) | 2.0 (1.2, 3.0) |
| Unimproved water | 1.0 (0.5, 1.8) | 0.3 (0.1, 0.5) | 0.1 (0.0, 0.2) |
| Use of biomass fuels | 1.7 (0.7, 2.7) | 0.4 (0.1, 0.7) | 0.2 (0.1, 0.4) |

**Fig 1: Stunting cases among 2-year olds attributable to individual risk factors**


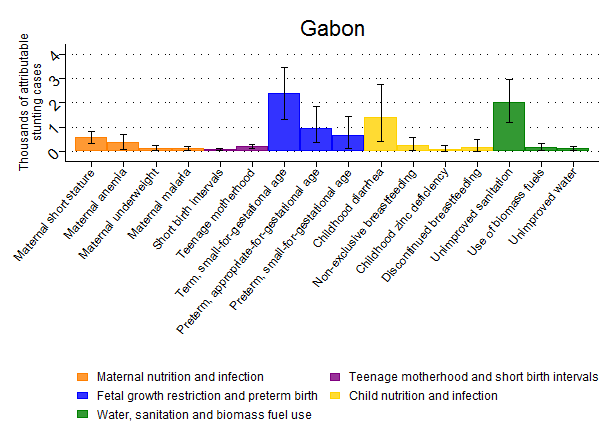


Gambia

**Region: Sub-Saharan Africa; Sub-region: Sub-Saharan Africa, West**

**Stunting prevalence among children age 2: 36.9%**

**Stunting cases among children age 2: 27,131**

**Population of children age 2: 73,615**

This country profile provides results for 2011 from the analysis presented in *Risk factors for childhood stunting in 137 developing countries: a comparative risk assessment analysis at global, regional, and country levels.*

**Table 1: Population attributable fraction (PAF in %), attributable stunting prevalence (percentage points) and number of stunting cases among 2-year olds attributable to individual risks and risk factor clusters (95% confidence intervals presented in parentheses)**

| **Description** | **PAF** | **Attributable stunting prevalence** | **Attributable stunting cases (thousands)** |
| --- | --- | --- | --- |
| **Maternal nutrition and infection** | **10.2 (7.4, 13.3)** | **3.8 (2.4, 5.3)** | **2.8 (1.8, 3.9)** |
| Maternal short stature | 4.2 (4.0, 4.4) | 1.6 (1.2, 2.0) | 1.1 (0.8, 1.4) |
| Maternal underweight | 2.0 (0.9, 3.4) | 0.7 (0.3, 1.3) | 0.5 (0.2, 0.9) |
| Maternal malaria | 0.4 (0.1, 0.7) | 0.2 (0.0, 0.3) | 0.1 (0.0, 0.2) |
| Maternal anemia | 3.9 (1.2, 7.0) | 1.4 (0.5, 2.7) | 1.1 (0.3, 2.0) |
| **Teenage motherhood and short birth intervals** | **1.9 (1.8, 2.0)** | **0.7 (0.5, 0.9)** | **0.5 (0.4, 0.6)** |
| Teenage motherhood | 1.1 (1.0, 1.1) | 0.4 (0.3, 0.5) | 0.3 (0.2, 0.4) |
| Short birth intervals | 0.8 (0.8, 0.9) | 0.3 (0.2, 0.4) | 0.2 (0.2, 0.3) |
| **Fetal growth restriction and preterm birth** | **35.1 (30.5, 39.0)** | **12.9 (9.4, 16.6)** | **9.5 (6.9, 12.2)** |
| Preterm, small-for-gestational age | 4.8 (0.7, 9.5) | 1.8 (0.2, 3.7) | 1.3 (0.2, 2.8) |
| Preterm, appropriate-for-gestational age | 6.6 (1.1, 12.1) | 2.5 (0.4, 4.6) | 1.8 (0.3, 3.4) |
| Term, small-for-gestational age | 26.9 (22.3, 32.0) | 9.9 (7.1, 13.1) | 7.3 (5.2, 9.6) |
| Low birth weight | 27.7 (23.7, 31.6) | 10.2 (7.3, 13.3) | 7.5 (5.4, 9.8) |
| **Child nutrition and infection** | **13.4 (5.2, 23.2)** | **4.9 (1.8, 8.7)** | **3.6 (1.3, 6.4)** |
| Childhood zinc deficiency | 1.5 (0.6, 2.8) | 0.6 (0.2, 1.2) | 0.4 (0.2, 0.9) |
| Childhood diarrhea | 12.7 (4.8, 22.7) | 4.7 (1.6, 8.5) | 3.4 (1.2, 6.2) |
| Non-exclusive breastfeeding | 1.1 (-2.0, 3.6) | 0.4 (-0.7, 1.4) | 0.3 (-0.5, 1.0) |
| HIV infection without HAART before 2 years | 0.2 (0.1, 0.5) | 0.1 (0.0, 0.2) | 0.0 (0.0, 0.1) |
| Discontinued breastfeeding | 0.3 (0.0, 0.8) | 0.1 (0.0, 0.3) | 0.1 (0.0, 0.2) |
| **Water, sanitation and biomass fuel use** | **19.3 (16.4, 22.4)** | **7.1 (5.0, 9.4)** | **5.2 (3.7, 6.9)** |
| Unimproved sanitation | 11.8 (9.1, 14.6) | 4.4 (2.9, 6.0) | 3.2 (2.2, 4.4) |
| Unimproved water | 1.0 (0.4, 1.6) | 0.4 (0.1, 0.6) | 0.3 (0.1, 0.5) |
| Use of biomass fuels | 7.6 (5.6, 9.8) | 2.8 (1.8, 4.0) | 2.1 (1.3, 2.9) |

**Fig 1: Stunting cases among 2-year olds attributable to individual risk factors**


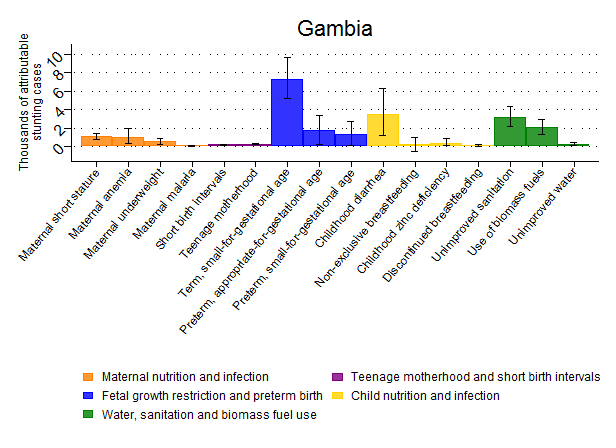


Georgia

**Region: Central Asia Eastern Europe/Central Asia; Sub-region: Asia, Central**

**Stunting prevalence among children age 2: 13.5%**

**Stunting cases among children age 2: 7,910**

**Population of children age 2: 58,540**

This country profile provides results for 2011 from the analysis presented in *Risk factors for childhood stunting in 137 developing countries: a comparative risk assessment analysis at global, regional, and country levels.*

**Table 1: Population attributable fraction (PAF in %), attributable stunting prevalence (percentage points) and number of stunting cases among 2-year olds attributable to individual risks and risk factor clusters (95% confidence intervals presented in parentheses)**

| **Description** | **PAF** | **Attributable stunting prevalence** | **Attributable stunting cases (thousands)** |
| --- | --- | --- | --- |
| **Maternal nutrition and infection** | **3.9 (3.3, 4.7)** | **0.5 (0.3, 0.7)** | **0.3 (0.2, 0.4)** |
| Maternal short stature | 2.9 (2.7, 3.1) | 0.4 (0.3, 0.5) | 0.2 (0.1, 0.3) |
| Maternal underweight | 0.3 (0.1, 0.6) | 0.0 (0.0, 0.1) | 0.0 (0.0, 0.1) |
| Maternal malaria | 0.0 (0.0, 0.0) | 0.0 (0.0, 0.0) | 0.0 (0.0, 0.0) |
| Maternal anemia | 0.8 (0.2, 1.6) | 0.1 (0.0, 0.2) | 0.1 (0.0, 0.1) |
| **Teenage motherhood and short birth intervals** | **1.6 (1.5, 1.7)** | **0.2 (0.1, 0.3)** | **0.1 (0.1, 0.2)** |
| Teenage motherhood | 0.5 (0.5, 0.6) | 0.1 (0.0, 0.1) | 0.0 (0.0, 0.1) |
| Short birth intervals | 1.0 (1.0, 1.1) | 0.1 (0.1, 0.2) | 0.1 (0.1, 0.1) |
| **Fetal growth restriction and preterm birth** | **22.9 (17.8, 27.9)** | **3.1 (1.9, 4.5)** | **1.8 (1.1, 2.6)** |
| Preterm, small-for-gestational age | 5.4 (2.7, 8.3) | 0.7 (0.3, 1.3) | 0.4 (0.2, 0.7) |
| Preterm, appropriate-for-gestational age | 4.8 (2.6, 7.4) | 0.7 (0.3, 1.1) | 0.4 (0.2, 0.6) |
| Term, small-for-gestational age | 14.4 (8.8, 19.9) | 1.9 (1.1, 3.0) | 1.1 (0.6, 1.7) |
| Low birth weight | 11.6 (9.6, 13.6) | 1.6 (1.0, 2.2) | 0.9 (0.6, 1.3) |
| **Child nutrition and infection** | **20.4 (8.8, 39.4)** | **2.8 (1.1, 5.6)** | **1.6 (0.7, 3.3)** |
| Childhood zinc deficiency | 6.9 (1.2, 37.6) | 0.9 (0.2, 5.2) | 0.5 (0.1, 3.1) |
| Childhood diarrhea | 17.7 (7.6, 28.4) | 2.4 (1.0, 4.2) | 1.4 (0.6, 2.4) |
| Non-exclusive breastfeeding | 3.6 (0.9, 7.3) | 0.5 (0.1, 1.0) | 0.3 (0.1, 0.6) |
| Discontinued breastfeeding | 2.8 (0.5, 6.3) | 0.4 (0.1, 0.9) | 0.2 (0.0, 0.5) |
| HIV infection without HAART before 2 years | No data | No data | No data |
| **Water, sanitation and biomass fuel use** | **4.3 (3.3, 5.3)** | **0.6 (0.4, 0.9)** | **0.3 (0.2, 0.5)** |
| Unimproved sanitation | 2.2 (1.4, 2.9) | 0.3 (0.2, 0.5) | 0.2 (0.1, 0.3) |
| Unimproved water | 0.4 (0.2, 0.8) | 0.1 (0.0, 0.1) | 0.0 (0.0, 0.1) |
| Use of biomass fuels | 1.8 (1.2, 2.6) | 0.2 (0.1, 0.4) | 0.1 (0.1, 0.2) |

**Fig 1: Stunting cases among 2-year olds attributable to individual risk factors**


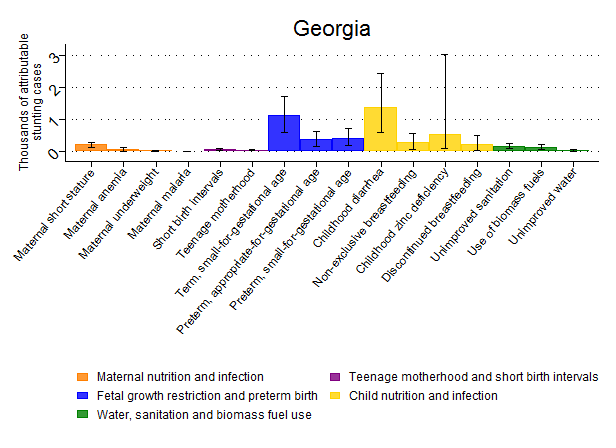


Ghana

**Region: Sub-Saharan Africa; Sub-region: Sub-Saharan Africa, West**

**Stunting prevalence among children age 2: 31.2%**

**Stunting cases among children age 2: 254,739**

**Population of children age 2: 816,046**

This country profile provides results for 2011 from the analysis presented in *Risk factors for childhood stunting in 137 developing countries: a comparative risk assessment analysis at global, regional, and country levels.*

**Table 1: Population attributable fraction (PAF in %), attributable stunting prevalence (percentage points) and number of stunting cases among 2-year olds attributable to individual risks and risk factor clusters (95% confidence intervals presented in parentheses)**

| **Description** | **PAF** | **Attributable stunting prevalence** | **Attributable stunting cases (thousands)** |
| --- | --- | --- | --- |
| **Maternal nutrition and infection** | **13.0 (10.2, 15.9)** | **4.1 (2.9, 5.5)** | **33.3 (23.9, 44.5)** |
| Maternal short stature | 5.9 (5.8, 6.1) | 1.8 (1.5, 2.2) | 15.1 (12.0, 18.2) |
| Maternal underweight | 1.0 (0.4, 1.7) | 0.3 (0.1, 0.5) | 2.6 (1.1, 4.4) |
| Maternal malaria | 3.7 (1.5, 5.8) | 1.1 (0.4, 1.9) | 9.3 (3.7, 15.5) |
| Maternal anemia | 3.1 (0.9, 5.2) | 1.0 (0.3, 1.6) | 7.9 (2.4, 13.5) |
| **Teenage motherhood and short birth intervals** | **1.3 (1.2, 1.4)** | **0.4 (0.3, 0.5)** | **3.4 (2.6, 4.1)** |
| Teenage motherhood | 0.7 (0.6, 0.8) | 0.2 (0.2, 0.3) | 1.8 (1.4, 2.3) |
| Short birth intervals | 0.6 (0.5, 0.7) | 0.2 (0.1, 0.2) | 1.6 (1.2, 2.0) |
| **Fetal growth restriction and preterm birth** | **30.7 (25.5, 35.1)** | **9.6 (7.3, 12.1)** | **78.2 (59.2, 98.8)** |
| Preterm, small-for-gestational age | 5.4 (0.9, 10.7) | 1.7 (0.3, 3.5) | 13.8 (2.2, 28.2) |
| Preterm, appropriate-for-gestational age | 7.5 (1.3, 13.5) | 2.3 (0.4, 4.4) | 19.1 (3.2, 35.8) |
| Term, small-for-gestational age | 20.7 (16.7, 25.3) | 6.5 (4.7, 8.4) | 52.8 (38.6, 68.7) |
| Low birth weight | 20.5 (17.3, 23.7) | 6.4 (4.9, 8.1) | 52.2 (39.8, 65.7) |
| **Child nutrition and infection** | **13.1 (4.7, 22.3)** | **4.1 (1.4, 7.4)** | **33.5 (11.6, 60.6)** |
| Childhood zinc deficiency | 1.0 (0.4, 1.9) | 0.3 (0.1, 0.6) | 2.5 (1.0, 4.7) |
| Childhood diarrhea | 12.7 (4.3, 21.9) | 4.0 (1.3, 7.3) | 32.4 (10.4, 59.2) |
| Non-exclusive breastfeeding | 1.0 (-1.1, 3.5) | 0.3 (-0.4, 1.1) | 2.6 (-2.9, 9.0) |
| HIV infection without HAART before 2 years | 0.2 (0.1, 0.4) | 0.0 (0.0, 0.1) | 0.4 (0.2, 0.9) |
| Discontinued breastfeeding | 0.5 (0.0, 1.5) | 0.2 (0.0, 0.5) | 1.3 (0.0, 3.9) |
| **Water, sanitation and biomass fuel use** | **28.7 (26.6, 30.8)** | **9.0 (7.0, 10.9)** | **73.2 (57.2, 89.3)** |
| Unimproved sanitation | 23.9 (22.0, 25.9) | 7.5 (5.9, 9.1) | 60.9 (47.8, 74.7) |
| Unimproved water | 1.3 (0.7, 1.9) | 0.4 (0.2, 0.6) | 3.3 (1.7, 5.2) |
| Use of biomass fuels | 5.1 (3.6, 6.7) | 1.6 (1.1, 2.2) | 13.1 (8.7, 18.0) |

**Fig 1: Stunting cases among 2-year olds attributable to individual risk factors**


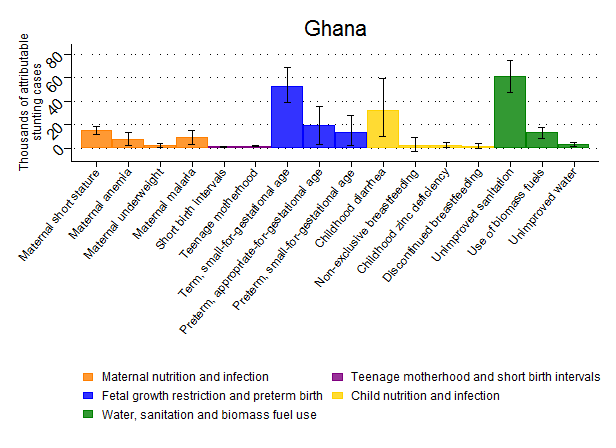


Grenada

**Region: Latin America/Caribbean; Sub-region: Caribbean**

**Stunting prevalence among children age 2: 19.4%**

**Stunting cases among children age 2: 393**

**Population of children age 2: 2,025**

This country profile provides results for 2011 from the analysis presented in *Risk factors for childhood stunting in 137 developing countries: a comparative risk assessment analysis at global, regional, and country levels.*

**Table 1: Population attributable fraction (PAF in %), attributable stunting prevalence (percentage points) and number of stunting cases among 2-year olds attributable to individual risks and risk factor clusters (95% confidence intervals presented in parentheses)**

| **Description** | **PAF** | **Attributable stunting prevalence** | **Attributable stunting cases (thousands)** |
| --- | --- | --- | --- |
| **Maternal nutrition and infection** | **3.5 (2.6, 4.6)** | **0.7 (0.2, 1.2)** | **0.0 (0.0, 0.0)** |
| Maternal short stature | 2.2 (2.0, 2.4) | 0.4 (0.2, 0.7) | 0.0 (0.0, 0.0) |
| Maternal underweight | 0.4 (0.0, 0.8) | 0.1 (0.0, 0.2) | 0.0 (0.0, 0.0) |
| Maternal malaria | 0.0 (0.0, 0.0) | 0.0 (0.0, 0.0) | 0.0 (0.0, 0.0) |
| Maternal anemia | 1.0 (0.2, 2.0) | 0.2 (0.0, 0.5) | 0.0 (0.0, 0.0) |
| **Teenage motherhood and short birth intervals** | **2.3 (2.2, 2.4)** | **0.4 (0.2, 0.7)** | **0.0 (0.0, 0.0)** |
| Teenage motherhood | 1.3 (1.3, 1.4) | 0.3 (0.1, 0.4) | 0.0 (0.0, 0.0) |
| Short birth intervals | 0.9 (0.9, 1.0) | 0.2 (0.1, 0.3) | 0.0 (0.0, 0.0) |
| **Fetal growth restriction and preterm birth** | **20.9 (15.9, 26.0)** | **4.1 (1.3, 6.9)** | **0.1 (0.0, 0.1)** |
| Preterm, small-for-gestational age | 5.8 (1.3, 10.9) | 1.1 (0.2, 2.5) | 0.0 (0.0, 0.1) |
| Preterm, appropriate-for-gestational age | 5.7 (1.4, 10.3) | 1.1 (0.2, 2.3) | 0.0 (0.0, 0.0) |
| Term, small-for-gestational age | 11.0 (7.1, 14.9) | 2.1 (0.7, 3.8) | 0.0 (0.0, 0.1) |
| Low birth weight | 12.6 (10.5, 14.8) | 2.4 (0.9, 4.1) | 0.0 (0.0, 0.1) |
| **Child nutrition and infection** | **17.3 (7.5, 27.9)** | **3.4 (0.9, 7.1)** | **0.1 (0.0, 0.1)** |
| Childhood zinc deficiency | 0.8 (0.2, 2.6) | 0.2 (0.0, 0.5) | 0.0 (0.0, 0.0) |
| Childhood diarrhea | 17.0 (7.1, 27.5) | 3.3 (0.8, 7.0) | 0.1 (0.0, 0.1) |
| Non-exclusive breastfeeding | 3.0 (0.2, 6.6) | 0.6 (0.0, 1.6) | 0.0 (0.0, 0.0) |
| HIV infection without HAART before 2 years | No data | No data | No data |
| Discontinued breastfeeding | 2.5 (0.4, 5.6) | 0.5 (0.0, 1.3) | 0.0 (0.0, 0.0) |
| **Water, sanitation and biomass fuel use** | **3.2 (2.3, 4.2)** | **0.6 (0.2, 1.1)** | **0.0 (0.0, 0.0)** |
| Unimproved sanitation | 2.9 (2.2, 3.7) | 0.6 (0.2, 1.0) | 0.0 (0.0, 0.0) |
| Unimproved water | 0.3 (0.1, 0.6) | 0.1 (0.0, 0.1) | 0.0 (0.0, 0.0) |
| Use of biomass fuels | 0.0 (-0.5, 0.4) | 0.0 (-0.1, 0.1) | 0.0 (0.0, 0.0) |

**Fig 1: Stunting cases among 2-year olds attributable to individual risk factors**


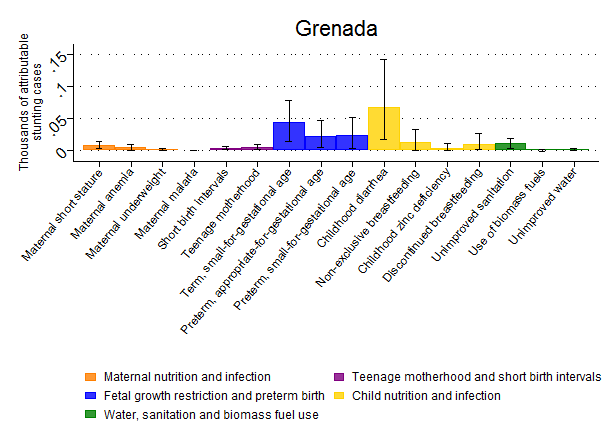


Guatemala

**Region: Latin America/Caribbean; Sub-region: Latin America, Central**

**Stunting prevalence among children age 2: 52.3%**

**Stunting cases among children age 2: 220,821**

**Population of children age 2: 422,440**

This country profile provides results for 2011 from the analysis presented in *Risk factors for childhood stunting in 137 developing countries: a comparative risk assessment analysis at global, regional, and country levels.*

**Table 1: Population attributable fraction (PAF in %), attributable stunting prevalence (percentage points) and number of stunting cases among 2-year olds attributable to individual risks and risk factor clusters (95% confidence intervals presented in parentheses)**

| **Description** | **PAF** | **Attributable stunting prevalence** | **Attributable stunting cases (thousands)** |
| --- | --- | --- | --- |
| **Maternal nutrition and infection** | **16.2 (15.4, 17.3)** | **8.5 (7.0, 10.0)** | **35.9 (29.7, 42.3)** |
| Maternal short stature | 15.0 (14.8, 15.3) | 7.9 (6.6, 9.1) | 33.2 (27.9, 38.6) |
| Maternal underweight | 0.3 (0.1, 0.6) | 0.1 (0.0, 0.3) | 0.6 (0.1, 1.2) |
| Maternal malaria | 0.0 (0.0, 0.0) | 0.0 (0.0, 0.0) | 0.0 (0.0, 0.0) |
| Maternal anemia | 1.1 (0.3, 2.3) | 0.6 (0.1, 1.2) | 2.5 (0.6, 5.1) |
| **Teenage motherhood and short birth intervals** | **2.5 (2.3, 2.6)** | **1.3 (1.1, 1.5)** | **5.4 (4.5, 6.4)** |
| Teenage motherhood | 1.2 (1.1, 1.3) | 0.6 (0.5, 0.7) | 2.6 (2.1, 3.1) |
| Short birth intervals | 1.3 (1.2, 1.4) | 0.7 (0.6, 0.8) | 2.9 (2.4, 3.4) |
| **Fetal growth restriction and preterm birth** | **20.7 (16.5, 24.8)** | **10.8 (8.1, 13.8)** | **45.7 (34.4, 58.2)** |
| Preterm, small-for-gestational age | 4.4 (2.0, 7.2) | 2.3 (1.0, 3.9) | 9.8 (4.3, 16.4) |
| Preterm, appropriate-for-gestational age | 4.3 (2.0, 6.8) | 2.3 (1.0, 3.6) | 9.6 (4.4, 15.4) |
| Term, small-for-gestational age | 13.2 (9.4, 17.4) | 6.9 (4.7, 9.6) | 29.3 (19.8, 40.5) |
| Low birth weight | 13.8 (11.5, 16.1) | 7.2 (5.6, 9.1) | 30.4 (23.8, 38.6) |
| **Child nutrition and infection** | **18.6 (8.0, 29.3)** | **9.7 (4.1, 15.5)** | **40.9 (17.4, 65.4)** |
| Childhood zinc deficiency | 0.9 (0.4, 1.6) | 0.5 (0.2, 0.8) | 2.1 (0.8, 3.5) |
| Childhood diarrhea | 18.2 (7.5, 28.8) | 9.5 (3.9, 15.3) | 40.1 (16.5, 64.5) |
| Non-exclusive breastfeeding | 1.6 (-2.1, 5.0) | 0.8 (-1.1, 2.7) | 3.5 (-4.6, 11.3) |
| Discontinued breastfeeding | 1.7 (0.2, 4.1) | 0.9 (0.1, 2.1) | 3.7 (0.4, 9.0) |
| HIV infection without HAART before 2 years | No data | No data | No data |
| **Water, sanitation and biomass fuel use** | **9.7 (7.7, 11.7)** | **5.1 (3.9, 6.5)** | **21.4 (16.4, 27.5)** |
| Unimproved sanitation | 6.5 (4.6, 8.4) | 3.4 (2.4, 4.6) | 14.4 (10.0, 19.5) |
| Unimproved water | 0.6 (0.3, 1.1) | 0.3 (0.1, 0.6) | 1.3 (0.6, 2.3) |
| Use of biomass fuels | 2.8 (1.9, 3.9) | 1.5 (0.9, 2.1) | 6.2 (4.0, 8.8) |

**Fig 1: Stunting cases among 2-year olds attributable to individual risk factors**


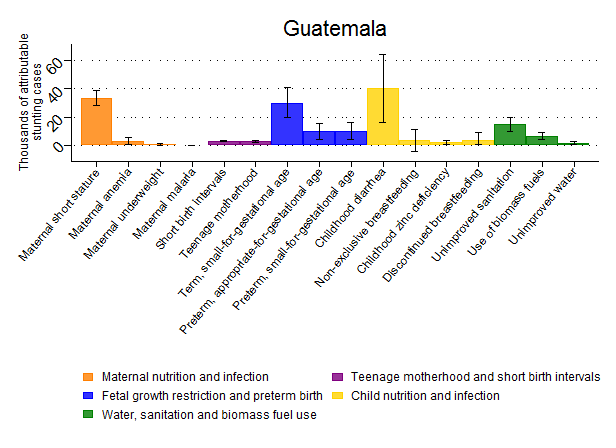


Guinea

**Region: Sub-Saharan Africa; Sub-region: Sub-Saharan Africa, West**

**Stunting prevalence among children age 2: 48.1%**

**Stunting cases among children age 2: 205,384**

**Population of children age 2: 427,348**

This country profile provides results for 2011 from the analysis presented in *Risk factors for childhood stunting in 137 developing countries: a comparative risk assessment analysis at global, regional, and country levels.*

**Table 1: Population attributable fraction (PAF in %), attributable stunting prevalence (percentage points) and number of stunting cases among 2-year olds attributable to individual risks and risk factor clusters (95% confidence intervals presented in parentheses)**

| **Description** | **PAF** | **Attributable stunting prevalence** | **Attributable stunting cases (thousands)** |
| --- | --- | --- | --- |
| **Maternal nutrition and infection** | **11.6 (9.3, 14.0)** | **5.6 (4.0, 7.3)** | **23.9 (17.2, 31.0)** |
| Maternal short stature | 5.5 (5.4, 5.7) | 2.7 (2.1, 3.2) | 11.4 (9.0, 13.8) |
| Maternal underweight | 1.3 (0.6, 2.2) | 0.6 (0.3, 1.1) | 2.8 (1.2, 4.7) |
| Maternal malaria | 2.4 (0.9, 3.9) | 1.2 (0.4, 2.0) | 4.9 (1.9, 8.4) |
| Maternal anemia | 2.8 (0.8, 4.8) | 1.4 (0.4, 2.4) | 5.8 (1.6, 10.3) |
| **Teenage motherhood and short birth intervals** | **1.8 (1.7, 1.9)** | **0.8 (0.7, 1.0)** | **3.6 (2.8, 4.4)** |
| Teenage motherhood | 1.2 (1.1, 1.3) | 0.6 (0.4, 0.7) | 2.4 (1.9, 2.9) |
| Short birth intervals | 0.6 (0.5, 0.7) | 0.3 (0.2, 0.4) | 1.2 (0.9, 1.5) |
| **Fetal growth restriction and preterm birth** | **30.7 (26.0, 35.2)** | **14.7 (11.1, 18.7)** | **62.9 (47.5, 80.1)** |
| Preterm, small-for-gestational age | 5.2 (0.6, 10.2) | 2.5 (0.3, 5.2) | 10.7 (1.3, 22.2) |
| Preterm, appropriate-for-gestational age | 7.1 (1.3, 13.0) | 3.4 (0.6, 6.6) | 14.5 (2.8, 28.3) |
| Term, small-for-gestational age | 21.2 (17.4, 25.2) | 10.2 (7.5, 13.2) | 43.6 (32.2, 56.5) |
| Low birth weight | 19.0 (16.0, 22.1) | 9.1 (6.8, 11.6) | 39.1 (29.0, 49.6) |
| **Child nutrition and infection** | **15.9 (5.6, 27.6)** | **7.6 (2.7, 13.5)** | **32.7 (11.4, 57.8)** |
| Childhood zinc deficiency | 0.7 (0.3, 1.2) | 0.3 (0.1, 0.6) | 1.3 (0.5, 2.4) |
| Childhood diarrhea | 15.7 (5.2, 27.4) | 7.5 (2.6, 13.4) | 32.1 (10.9, 57.1) |
| Non-exclusive breastfeeding | 1.5 (-1.2, 4.8) | 0.7 (-0.5, 2.4) | 3.2 (-2.3, 10.0) |
| HIV infection without HAART before 2 years | 0.1 (0.1, 0.3) | 0.1 (0.0, 0.1) | 0.3 (0.2, 0.6) |
| Discontinued breastfeeding | 0.6 (0.0, 1.7) | 0.3 (0.0, 0.8) | 1.2 (0.1, 3.4) |
| **Water, sanitation and biomass fuel use** | **28.5 (26.1, 30.8)** | **13.7 (10.5, 16.7)** | **58.4 (44.9, 71.4)** |
| Unimproved sanitation | 22.8 (20.6, 24.9) | 11.0 (8.5, 13.4) | 46.9 (36.3, 57.1) |
| Unimproved water | 2.1 (1.1, 3.4) | 1.0 (0.5, 1.7) | 4.4 (2.3, 7.1) |
| Use of biomass fuels | 5.3 (3.8, 6.8) | 2.5 (1.7, 3.6) | 10.8 (7.1, 15.2) |

**Fig 1: Stunting cases among 2-year olds attributable to individual risk factors**


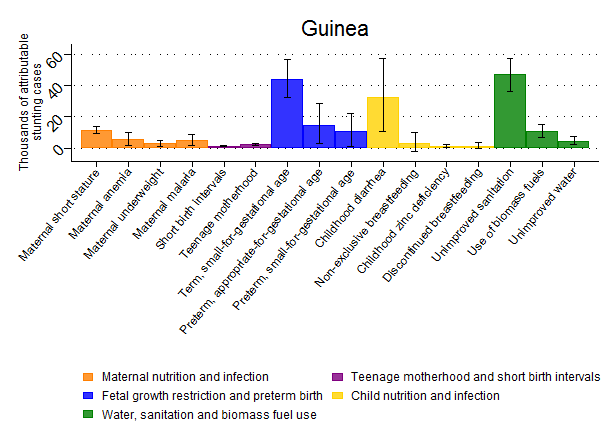


Guinea-Bissau

**Region: Sub-Saharan Africa; Sub-region: Sub-Saharan Africa, West**

**Stunting prevalence among children age 2: 44.2%**

**Stunting cases among children age 2: 27,849**

**Population of children age 2: 62,983**

This country profile provides results for 2011 from the analysis presented in *Risk factors for childhood stunting in 137 developing countries: a comparative risk assessment analysis at global, regional, and country levels.*

**Table 1: Population attributable fraction (PAF in %), attributable stunting prevalence (percentage points) and number of stunting cases among 2-year olds attributable to individual risks and risk factor clusters (95% confidence intervals presented in parentheses)**

| **Description** | **PAF** | **Attributable stunting prevalence** | **Attributable stunting cases (thousands)** |
| --- | --- | --- | --- |
| **Maternal nutrition and infection** | **12.4 (9.4, 15.8)** | **5.5 (3.6, 7.7)** | **3.5 (2.3, 4.9)** |
| Maternal short stature | 5.6 (5.3, 5.8) | 2.5 (1.8, 3.1) | 1.5 (1.1, 2.0) |
| Maternal underweight | 2.2 (0.9, 3.9) | 1.0 (0.4, 1.8) | 0.6 (0.2, 1.1) |
| Maternal malaria | 1.2 (0.5, 2.1) | 0.5 (0.2, 1.0) | 0.3 (0.1, 0.6) |
| Maternal anemia | 3.9 (1.2, 7.1) | 1.7 (0.5, 3.4) | 1.1 (0.3, 2.1) |
| **Teenage motherhood and short birth intervals** | **1.9 (1.8, 2.0)** | **0.8 (0.6, 1.1)** | **0.5 (0.4, 0.7)** |
| Teenage motherhood | 1.1 (1.0, 1.1) | 0.5 (0.3, 0.6) | 0.3 (0.2, 0.4) |
| Short birth intervals | 0.8 (0.8, 0.9) | 0.4 (0.3, 0.5) | 0.2 (0.2, 0.3) |
| **Fetal growth restriction and preterm birth** | **37.6 (33.2, 41.6)** | **16.6 (11.8, 21.6)** | **10.5 (7.5, 13.6)** |
| Preterm, small-for-gestational age | 3.9 (0.8, 7.3) | 1.7 (0.3, 3.4) | 1.1 (0.2, 2.1) |
| Preterm, appropriate-for-gestational age | 5.2 (1.1, 9.5) | 2.3 (0.5, 4.5) | 1.4 (0.3, 2.8) |
| Term, small-for-gestational age | 31.5 (26.5, 36.6) | 13.9 (9.7, 18.3) | 8.8 (6.1, 11.5) |
| Low birth weight | 31.5 (27.1, 35.7) | 13.9 (9.8, 18.2) | 8.8 (6.2, 11.5) |
| **Child nutrition and infection** | **17.8 (6.8, 31.0)** | **7.9 (2.9, 14.3)** | **5.0 (1.8, 9.0)** |
| Childhood zinc deficiency | 0.9 (0.4, 1.6) | 0.4 (0.2, 0.8) | 0.3 (0.1, 0.5) |
| Childhood diarrhea | 17.5 (6.3, 30.6) | 7.7 (2.7, 14.1) | 4.9 (1.7, 8.9) |
| Non-exclusive breastfeeding | 2.3 (-0.4, 6.0) | 1.0 (-0.2, 2.8) | 0.6 (-0.1, 1.8) |
| Discontinued breastfeeding | 0.5 (0.0, 1.4) | 0.2 (0.0, 0.6) | 0.1 (0.0, 0.4) |
| HIV infection without HAART before 2 years | 0.5 (0.3, 1.0) | 0.2 (0.1, 0.4) | 0.1 (0.1, 0.3) |
| **Water, sanitation and biomass fuel use** | **31.3 (28.6, 34.0)** | **13.8 (9.8, 17.8)** | **8.7 (6.2, 11.2)** |
| Unimproved sanitation | 22.6 (20.6, 24.6) | 10.0 (7.0, 13.0) | 6.3 (4.4, 8.2) |
| Unimproved water | 2.6 (1.5, 3.9) | 1.1 (0.6, 1.8) | 0.7 (0.4, 1.1) |
| Use of biomass fuels | 8.8 (6.4, 11.3) | 3.9 (2.5, 5.5) | 2.5 (1.6, 3.5) |

**Fig 1: Stunting cases among 2-year olds attributable to individual risk factors**


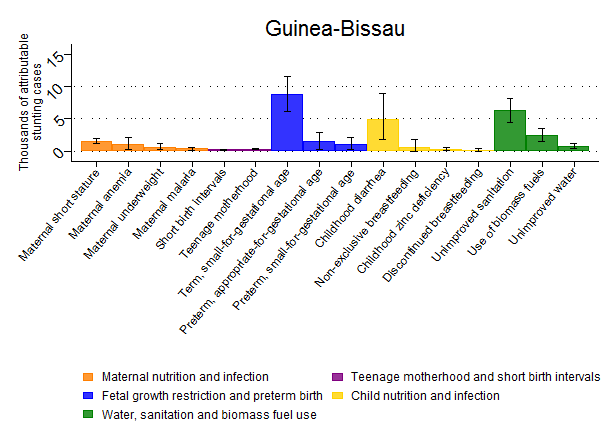


Guyana

**Region: Latin America/Caribbean; Sub-region: Caribbean**

**Stunting prevalence among children age 2: 18.2%**

**Stunting cases among children age 2: 2,642**

**Population of children age 2: 14,535**

This country profile provides results for 2011 from the analysis presented in *Risk factors for childhood stunting in 137 developing countries: a comparative risk assessment analysis at global, regional, and country levels.*

**Table 1: Population attributable fraction (PAF in %), attributable stunting prevalence (percentage points) and number of stunting cases among 2-year olds attributable to individual risks and risk factor clusters (95% confidence intervals presented in parentheses)**

| **Description** | **PAF** | **Attributable stunting prevalence** | **Attributable stunting cases (thousands)** |
| --- | --- | --- | --- |
| **Maternal nutrition and infection** | **8.8 (7.7, 10.1)** | **1.6 (1.2, 2.1)** | **0.2 (0.2, 0.3)** |
| Maternal short stature | 7.0 (6.7, 7.2) | 1.3 (1.0, 1.6) | 0.2 (0.1, 0.2) |
| Maternal underweight | 0.6 (0.1, 1.2) | 0.1 (0.0, 0.2) | 0.0 (0.0, 0.0) |
| Maternal malaria | 0.0 (0.0, 0.0) | 0.0 (0.0, 0.0) | 0.0 (0.0, 0.0) |
| Maternal anemia | 1.5 (0.4, 2.6) | 0.3 (0.1, 0.5) | 0.0 (0.0, 0.1) |
| **Teenage motherhood and short birth intervals** | **2.4 (2.2, 2.5)** | **0.4 (0.3, 0.5)** | **0.1 (0.0, 0.1)** |
| Teenage motherhood | 1.4 (1.3, 1.6) | 0.3 (0.2, 0.3) | 0.0 (0.0, 0.0) |
| Short birth intervals | 1.0 (0.9, 1.1) | 0.2 (0.1, 0.2) | 0.0 (0.0, 0.0) |
| **Fetal growth restriction and preterm birth** | **27.4 (21.3, 32.6)** | **5.0 (3.5, 6.6)** | **0.7 (0.5, 1.0)** |
| Preterm, small-for-gestational age | 7.0 (1.5, 12.9) | 1.3 (0.3, 2.5) | 0.2 (0.0, 0.4) |
| Preterm, appropriate-for-gestational age | 6.8 (0.9, 12.2) | 1.2 (0.2, 2.3) | 0.2 (0.0, 0.3) |
| Term, small-for-gestational age | 16.3 (12.0, 20.6) | 3.0 (2.0, 4.1) | 0.4 (0.3, 0.6) |
| Low birth weight | 17.7 (14.9, 20.6) | 3.2 (2.3, 4.2) | 0.5 (0.3, 0.6) |
| **Child nutrition and infection** | **19.1 (8.1, 30.0)** | **3.5 (1.5, 5.7)** | **0.5 (0.2, 0.8)** |
| Childhood zinc deficiency | 1.0 (0.3, 2.0) | 0.2 (0.1, 0.4) | 0.0 (0.0, 0.1) |
| Childhood diarrhea | 18.7 (7.7, 29.7) | 3.4 (1.4, 5.6) | 0.5 (0.2, 0.8) |
| Non-exclusive breastfeeding | 3.1 (0.1, 6.9) | 0.6 (0.0, 1.3) | 0.1 (0.0, 0.2) |
| Discontinued breastfeeding | 2.1 (0.3, 5.1) | 0.4 (0.0, 0.9) | 0.1 (0.0, 0.1) |
| HIV infection without HAART before 2 years | No data | No data | No data |
| **Water, sanitation and biomass fuel use** | **6.5 (5.0, 8.0)** | **1.2 (0.8, 1.6)** | **0.2 (0.1, 0.2)** |
| Unimproved sanitation | 5.6 (4.2, 7.0) | 1.0 (0.7, 1.4) | 0.1 (0.1, 0.2) |
| Unimproved water | 0.5 (0.3, 0.8) | 0.1 (0.0, 0.2) | 0.0 (0.0, 0.0) |
| Use of biomass fuels | 0.5 (0.0, 1.0) | 0.1 (0.0, 0.2) | 0.0 (0.0, 0.0) |

**Fig 1: Stunting cases among 2-year olds attributable to individual risk factors**


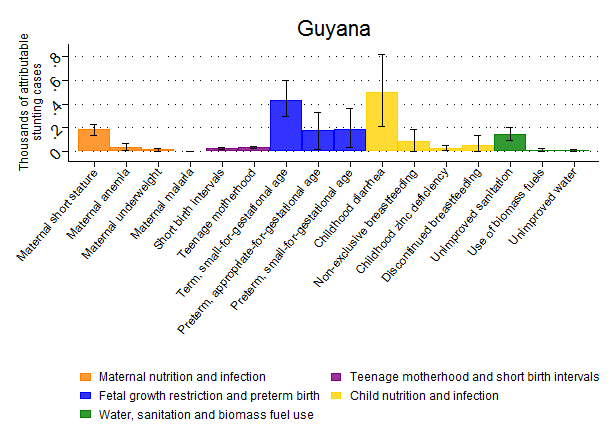


Haiti

**Region: Latin America/Caribbean; Sub-region: Caribbean**

**Stunting prevalence among children age 2: 30.1%**

**Stunting cases among children age 2: 79,998**

**Population of children age 2: 266,086**

This country profile provides results for 2011 from the analysis presented in *Risk factors for childhood stunting in 137 developing countries: a comparative risk assessment analysis at global, regional, and country levels.*

**Table 1: Population attributable fraction (PAF in %), attributable stunting prevalence (percentage points) and number of stunting cases among 2-year olds attributable to individual risks and risk factor clusters (95% confidence intervals presented in parentheses)**

| **Description** | **PAF** | **Attributable stunting prevalence** | **Attributable stunting cases (thousands)** |
| --- | --- | --- | --- |
| **Maternal nutrition and infection** | **11.0 (8.3, 13.8)** | **3.3 (2.2, 4.6)** | **8.8 (5.8, 12.2)** |
| Maternal short stature | 5.6 (5.4, 5.7) | 1.7 (1.2, 2.1) | 4.4 (3.2, 5.7) |
| Maternal underweight | 1.9 (0.7, 3.6) | 0.6 (0.2, 1.1) | 1.5 (0.6, 2.9) |
| Maternal malaria | 0.0 (0.0, 0.0) | 0.0 (0.0, 0.0) | 0.0 (0.0, 0.0) |
| Maternal anemia | 3.9 (1.1, 6.6) | 1.2 (0.3, 2.1) | 3.1 (0.9, 5.6) |
| **Teenage motherhood and short birth intervals** | **1.8 (1.7, 1.9)** | **0.5 (0.4, 0.7)** | **1.4 (1.0, 1.8)** |
| Teenage motherhood | 0.9 (0.8, 1.0) | 0.3 (0.2, 0.3) | 0.7 (0.5, 0.9) |
| Short birth intervals | 0.9 (0.8, 1.0) | 0.3 (0.2, 0.4) | 0.7 (0.5, 0.9) |
| **Fetal growth restriction and preterm birth** | **36.2 (31.2, 40.6)** | **10.9 (7.6, 14.4)** | **29.0 (20.3, 38.4)** |
| Preterm, small-for-gestational age | 6.3 (1.5, 12.0) | 1.9 (0.4, 3.8) | 5.0 (1.1, 10.2) |
| Preterm, appropriate-for-gestational age | 6.1 (1.4, 11.0) | 1.8 (0.4, 3.5) | 4.9 (1.1, 9.3) |
| Term, small-for-gestational age | 27.4 (21.6, 32.9) | 8.2 (5.6, 11.3) | 21.9 (14.8, 30.0) |
| Low birth weight | 32.1 (27.7, 36.3) | 9.7 (6.7, 12.8) | 25.7 (17.7, 34.1) |
| **Child nutrition and infection** | **24.6 (10.6, 39.9)** | **7.4 (2.9, 12.7)** | **19.7 (7.7, 33.9)** |
| Childhood zinc deficiency | 1.6 (0.6, 2.9) | 0.5 (0.2, 0.9) | 1.3 (0.5, 2.4) |
| Childhood diarrhea | 24.0 (10.1, 39.3) | 7.2 (2.7, 12.6) | 19.3 (7.1, 33.4) |
| Non-exclusive breastfeeding | 3.2 (-1.2, 8.5) | 1.0 (-0.3, 2.6) | 2.6 (-0.9, 6.9) |
| Discontinued breastfeeding | 2.1 (0.3, 5.2) | 0.6 (0.1, 1.7) | 1.7 (0.2, 4.5) |
| HIV infection without HAART before 2 years | No data | No data | No data |
| **Water, sanitation and biomass fuel use** | **28.2 (22.6, 33.2)** | **8.5 (5.7, 11.5)** | **22.5 (15.1, 30.5)** |
| Unimproved sanitation | 19.4 (13.8, 24.7) | 5.8 (3.6, 8.3) | 15.5 (9.7, 22.0) |
| Unimproved water | 2.4 (1.1, 3.9) | 0.7 (0.3, 1.2) | 1.9 (0.8, 3.2) |
| Use of biomass fuels | 8.7 (6.2, 11.3) | 2.6 (1.6, 3.8) | 7.0 (4.4, 10.0) |

**Fig 1: Stunting cases among 2-year olds attributable to individual risk factors**


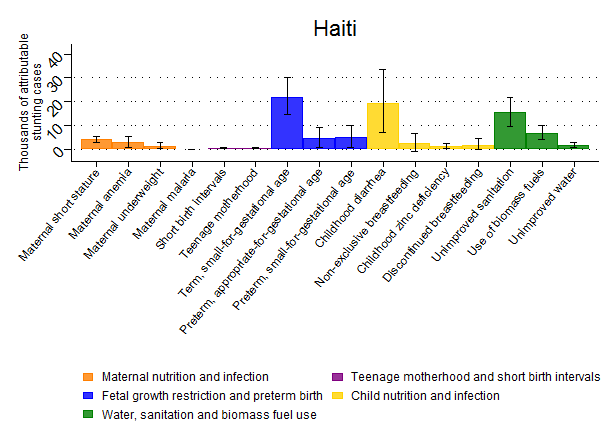


Honduras

**Region: Latin America/Caribbean; Sub-region: Latin America, Central**

**Stunting prevalence among children age 2: 31.3%**

**Stunting cases among children age 2: 54,880**

**Population of children age 2: 175,249**

This country profile provides results for 2011 from the analysis presented in *Risk factors for childhood stunting in 137 developing countries: a comparative risk assessment analysis at global, regional, and country levels.*

**Table 1: Population attributable fraction (PAF in %), attributable stunting prevalence (percentage points) and number of stunting cases among 2-year olds attributable to individual risks and risk factor clusters (95% confidence intervals presented in parentheses)**

| **Description** | **PAF** | **Attributable stunting prevalence** | **Attributable stunting cases (thousands)** |
| --- | --- | --- | --- |
| **Maternal nutrition and infection** | **12.1 (11.3, 13.2)** | **3.8 (2.7, 4.9)** | **6.6 (4.7, 8.7)** |
| Maternal short stature | 10.8 (10.6, 11.1) | 3.4 (2.4, 4.3) | 6.0 (4.3, 7.6) |
| Maternal underweight | 0.5 (0.1, 0.9) | 0.1 (0.0, 0.3) | 0.2 (0.1, 0.5) |
| Maternal malaria | 0.0 (0.0, 0.0) | 0.0 (0.0, 0.0) | 0.0 (0.0, 0.0) |
| Maternal anemia | 1.0 (0.2, 2.0) | 0.3 (0.1, 0.7) | 0.5 (0.1, 1.2) |
| **Teenage motherhood and short birth intervals** | **2.3 (2.2, 2.4)** | **0.7 (0.5, 0.9)** | **1.3 (0.9, 1.6)** |
| Teenage motherhood | 1.4 (1.3, 1.5) | 0.4 (0.3, 0.6) | 0.8 (0.6, 1.0) |
| Short birth intervals | 0.9 (0.8, 1.0) | 0.3 (0.2, 0.4) | 0.5 (0.3, 0.6) |
| **Fetal growth restriction and preterm birth** | **24.9 (18.5, 30.5)** | **7.8 (5.1, 10.7)** | **13.7 (8.9, 18.8)** |
| Preterm, small-for-gestational age | 6.4 (0.8, 12.4) | 2.0 (0.2, 4.0) | 3.5 (0.4, 7.0) |
| Preterm, appropriate-for-gestational age | 6.4 (1.0, 11.6) | 2.0 (0.3, 3.9) | 3.5 (0.5, 6.9) |
| Term, small-for-gestational age | 14.2 (10.4, 18.5) | 4.5 (2.8, 6.5) | 7.8 (5.0, 11.4) |
| Low birth weight | 16.4 (13.7, 19.1) | 5.1 (3.5, 6.9) | 9.0 (6.2, 12.1) |
| **Child nutrition and infection** | **18.0 (7.7, 28.4)** | **5.7 (2.3, 9.4)** | **9.9 (4.1, 16.5)** |
| Childhood zinc deficiency | 1.0 (0.4, 1.9) | 0.3 (0.1, 0.6) | 0.6 (0.2, 1.1) |
| Childhood diarrhea | 17.6 (7.2, 27.9) | 5.5 (2.2, 9.2) | 9.7 (3.8, 16.2) |
| Non-exclusive breastfeeding | 2.5 (-0.5, 6.2) | 0.8 (-0.1, 2.0) | 1.4 (-0.3, 3.5) |
| Discontinued breastfeeding | 1.7 (0.2, 4.0) | 0.5 (0.1, 1.3) | 0.9 (0.1, 2.4) |
| HIV infection without HAART before 2 years | No data | No data | No data |
| **Water, sanitation and biomass fuel use** | **10.3 (8.8, 11.9)** | **3.2 (2.3, 4.3)** | **5.7 (4.0, 7.5)** |
| Unimproved sanitation | 6.9 (5.6, 8.2) | 2.2 (1.5, 2.9) | 3.8 (2.6, 5.1) |
| Unimproved water | 0.9 (0.6, 1.4) | 0.3 (0.2, 0.5) | 0.5 (0.3, 0.8) |
| Use of biomass fuels | 2.8 (1.9, 3.8) | 0.9 (0.5, 1.3) | 1.5 (0.9, 2.3) |

**Fig 1: Stunting cases among 2-year olds attributable to individual risk factors**


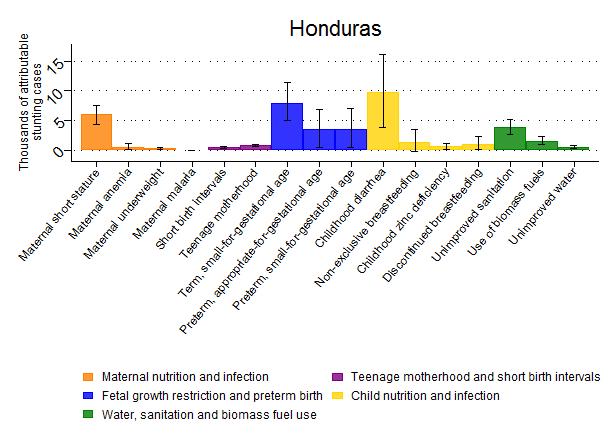


India

**Region: South Asia; Sub-region: Asia, South**

**Stunting prevalence among children age 2: 44.3%**

**Stunting cases among children age 2: 11,786,792**

**Population of children age 2: 26,594,676**

This country profile provides results for 2011 from the analysis presented in *Risk factors for childhood stunting in 137 developing countries: a comparative risk assessment analysis at global, regional, and country levels.*

**Table 1: Population attributable fraction (PAF in %), attributable stunting prevalence (percentage points) and number of stunting cases among 2-year olds attributable to individual risks and risk factor clusters (95% confidence intervals presented in parentheses)**

| **Description** | **PAF** | **Attributable stunting prevalence** | **Attributable stunting cases (thousands)** |
| --- | --- | --- | --- |
| **Maternal nutrition and infection** | **20.1 (16.7, 23.5)** | **8.9 (6.1, 11.9)** | **2367 (1634, 3175.1)** |
| Maternal short stature | 11.9 (11.7, 12.1) | 5.3 (3.8, 6.7) | 1399.6 (1000.6, 1791.9) |
| Maternal underweight | 4.9 (3.0, 7.1) | 2.2 (1.2, 3.4) | 581 (319.5, 908.4) |
| Maternal malaria | 0.0 (0.0, 0.0) | 0.0 (0.0, 0.0) | 0.0 (0.0, 0.0) |
| Maternal anemia | 4.6 (1.4, 8.0) | 2.0 (0.6, 3.8) | 543.8 (160.2, 1005.1) |
| **Teenage motherhood and short birth intervals** | **2.1 (2.0, 2.2)** | **0.9 (0.7, 1.2)** | **249.4 (176.2, 322.1)** |
| Teenage motherhood | 1.1 (1.0, 1.1) | 0.5 (0.3, 0.6) | 125.2 (88.4, 162.4) |
| Short birth intervals | 1.1 (1.0, 1.1) | 0.5 (0.3, 0.6) | 125.6 (88.5, 162.4) |
| **Fetal growth restriction and preterm birth** | **41.3 (37.6, 44.9)** | **18.3 (12.9, 24.0)** | **4864.1 (3439.2, 6375.1)** |
| Preterm, small-for-gestational age | 5.7 (3.6, 8.4) | 2.5 (1.4, 4.0) | 668.2 (372.3, 1058.7) |
| Preterm, appropriate-for-gestational age | 5.2 (3.5, 7.1) | 2.3 (1.3, 3.3) | 608.2 (348.5, 886.8) |
| Term, small-for-gestational age | 34.3 (29.8, 39.0) | 15.2 (10.6, 20.2) | 4046.6 (2813.2, 5363.8) |
| Low birth weight | 34.7 (30.1, 39.0) | 15.4 (10.6, 20.6) | 4085.7 (2812.6, 5475.4) |
| **Child nutrition and infection** | **11.7 (4.8, 19.7)** | **5.2 (2.1, 9.6)** | **1381.4 (545.3, 2540.2)** |
| Childhood zinc deficiency | 1.2 (0.5, 2.2) | 0.5 (0.2, 1.0) | 137.3 (48.2, 266.8) |
| Childhood diarrhea | 11.2 (4.2, 19.1) | 5.0 (1.8, 9.3) | 1320.3 (484, 2475.9) |
| Non-exclusive breastfeeding | 1.1 (-1.1, 3.4) | 0.5 (-0.5, 1.5) | 134.3 (-124.6, 410.3) |
| HIV infection without HAART before 2 years | 0.0 (0.0, 0.0) | 0.0 (0.0, 0.0) | 2.4 (1.1, 5.4) |
| Discontinued breastfeeding | 0.8 (0.1, 2.3) | 0.4 (0.0, 1.0) | 98.4 (11.1, 265.1) |
| **Water, sanitation and biomass fuel use** | **25.2 (22.6, 27.9)** | **11.2 (7.7, 14.5)** | **2972.2 (2050, 3846.8)** |
| Unimproved sanitation | 18.9 (17.0, 20.9) | 8.4 (5.8, 10.9) | 2227.1 (1543.2, 2900.9) |
| Unimproved water | 0.9 (0.5, 1.3) | 0.4 (0.2, 0.6) | 103 (54.8, 164.9) |
| Use of biomass fuels | 7.0 (4.8, 9.6) | 3.1 (1.9, 4.5) | 822.9 (502.6, 1204) |

**Fig 1: Stunting cases among 2-year olds attributable to individual risk factors**


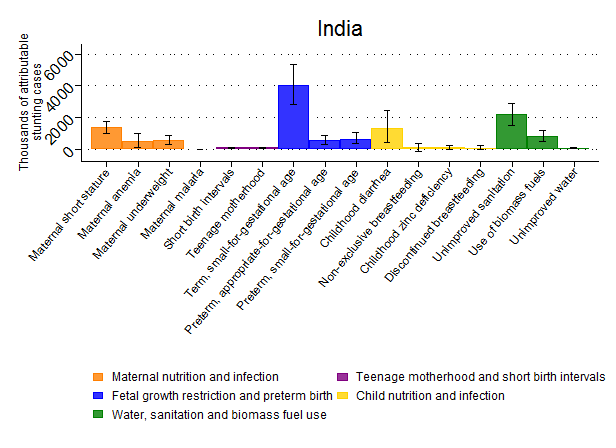


Indonesia

**Region: East Asia/Pacific; Sub-region: Asia, Southeast**

**Stunting prevalence among children age 2: 36.5%**

**Stunting cases among children age 2: 1,845,478**

**Population of children age 2: 5,051,107**

This country profile provides results for 2011 from the analysis presented in *Risk factors for childhood stunting in 137 developing countries: a comparative risk assessment analysis at global, regional, and country levels.*

**Table 1: Population attributable fraction (PAF in %), attributable stunting prevalence (percentage points) and number of stunting cases among 2-year olds attributable to individual risks and risk factor clusters (95% confidence intervals presented in parentheses)**

| **Description** | **PAF** | **Attributable stunting prevalence** | **Attributable stunting cases (thousands)** |
| --- | --- | --- | --- |
| **Maternal nutrition and infection** | **15.0 (13.8, 16.4)** | **5.5 (4.2, 6.8)** | **277.6 (209.8, 343.2)** |
| Maternal short stature | 12.6 (12.4, 12.8) | 4.6 (3.6, 5.6) | 232.3 (179.4, 283.2) |
| Maternal underweight | 1.4 (0.7, 2.3) | 0.5 (0.2, 0.9) | 26.5 (12.3, 44.7) |
| Maternal malaria | 0.0 (0.0, 0.0) | 0.0 (0.0, 0.0) | 0.0 (0.0, 0.0) |
| Maternal anemia | 1.4 (0.3, 2.8) | 0.5 (0.1, 1.0) | 25.7 (6.2, 52.0) |
| **Teenage motherhood and short birth intervals** | **1.2 (1.1, 1.2)** | **0.4 (0.3, 0.5)** | **21.3 (16.3, 26.3)** |
| Teenage motherhood | 0.6 (0.6, 0.6) | 0.2 (0.2, 0.3) | 11.1 (8.4, 13.7) |
| Short birth intervals | 0.6 (0.5, 0.6) | 0.2 (0.2, 0.3) | 10.3 (7.8, 12.9) |
| **Fetal growth restriction and preterm birth** | **31.0 (26.3, 35.4)** | **11.3 (8.3, 14.5)** | **572.4 (421.4, 733.1)** |
| Preterm, small-for-gestational age | 7.9 (4.3, 12.0) | 2.9 (1.4, 4.7) | 146.2 (72.9, 238.8) |
| Preterm, appropriate-for-gestational age | 7.3 (4.3, 10.8) | 2.7 (1.5, 4.2) | 135.6 (75.5, 212.6) |
| Term, small-for-gestational age | 19.1 (14.1, 24.0) | 7.0 (4.6, 9.5) | 352.6 (234.4, 480.2) |
| Low birth weight | 17.6 (14.8, 20.5) | 6.4 (4.8, 8.2) | 324.7 (240.3, 416.6) |
| **Child nutrition and infection** | **10.8 (4.2, 18.7)** | **4.0 (1.5, 7.2)** | **199.8 (75.5, 362.2)** |
| Childhood zinc deficiency | 1.5 (0.6, 2.9) | 0.6 (0.2, 1.1) | 27.9 (11.0, 55.7) |
| Childhood diarrhea | 10.2 (3.7, 18.0) | 3.7 (1.3, 6.9) | 187.3 (67.3, 348.6) |
| Non-exclusive breastfeeding | 1.4 (-0.4, 3.6) | 0.5 (-0.2, 1.3) | 25.5 (-7.9, 67.4) |
| Discontinued breastfeeding | 0.7 (0.0, 1.9) | 0.3 (0.0, 0.7) | 13.0 (0.9, 35.4) |
| HIV infection without HAART before 2 years | No data | No data | No data |
| **Water, sanitation and biomass fuel use** | **15.9 (13.9, 17.9)** | **5.8 (4.4, 7.4)** | **293.1 (221.9, 375.5)** |
| Unimproved sanitation | 12.3 (10.5, 14.3) | 4.5 (3.3, 5.8) | 227.6 (167.3, 291.1) |
| Unimproved water | 1.3 (0.7, 1.9) | 0.5 (0.3, 0.7) | 23.8 (13.2, 37.1) |
| Use of biomass fuels | 2.8 (1.6, 4.2) | 1.0 (0.6, 1.6) | 51.6 (28.5, 79.6) |

**Fig 1: Stunting cases among 2-year olds attributable to individual risk factors**


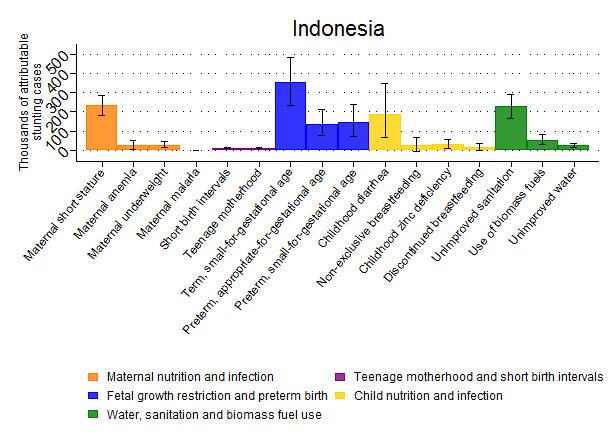


Iran (Islamic Republic of)

**Region: North Africa/Middle East; Sub-region: North Africa / Middle East**

**Stunting prevalence among children age 2: 18.8%**

**Stunting cases among children age 2: 253,601**

**Population of children age 2: 1,349,259**

This country profile provides results for 2011 from the analysis presented in *Risk factors for childhood stunting in 137 developing countries: a comparative risk assessment analysis at global, regional, and country levels.*

**Table 1: Population attributable fraction (PAF in %), attributable stunting prevalence (percentage points) and number of stunting cases among 2-year olds attributable to individual risks and risk factor clusters (95% confidence intervals presented in parentheses)**

| **Description** | **PAF** | **Attributable stunting prevalence** | **Attributable stunting cases (thousands)** |
| --- | --- | --- | --- |
| **Maternal nutrition and infection** | **7.2 (6.6, 8.1)** | **1.4 (0.7, 2.1)** | **18.4 (9.9, 28.0)** |
| Maternal short stature | 6.1 (5.9, 6.2) | 1.1 (0.6, 1.7) | 15.4 (8.5, 23.3) |
| Maternal underweight | 0.4 (0.2, 0.7) | 0.1 (0.0, 0.1) | 1.0 (0.4, 1.9) |
| Maternal malaria | 0.0 (0.0, 0.0) | 0.0 (0.0, 0.0) | 0.0 (0.0, 0.0) |
| Maternal anemia | 0.9 (0.2, 1.7) | 0.2 (0.0, 0.4) | 2.2 (0.4, 4.8) |
| **Teenage motherhood and short birth intervals** | **1.8 (1.7, 1.9)** | **0.3 (0.2, 0.5)** | **4.5 (2.4, 6.8)** |
| Teenage motherhood | 0.5 (0.5, 0.6) | 0.1 (0.1, 0.2) | 1.4 (0.7, 2.1) |
| Short birth intervals | 1.2 (1.1, 1.3) | 0.2 (0.1, 0.4) | 3.1 (1.7, 4.8) |
| **Fetal growth restriction and preterm birth** | **25.9 (21.1, 30.5)** | **4.9 (2.6, 7.6)** | **65.8 (35.1, 101.9)** |
| Preterm, small-for-gestational age | 7.4 (4.2, 11.5) | 1.4 (0.6, 2.4) | 18.7 (8.0, 32.9) |
| Preterm, appropriate-for-gestational age | 6.7 (4.2, 9.7) | 1.3 (0.6, 2.2) | 17.0 (7.5, 29.5) |
| Term, small-for-gestational age | 14.2 (9.1, 19.0) | 2.7 (1.2, 4.5) | 36.2 (16.5, 61.0) |
| Low birth weight | 12.2 (10.1, 14.3) | 2.3 (1.2, 3.5) | 30.9 (16.3, 46.7) |
| **Child nutrition and infection** | **13.0 (4.7, 23.8)** | **2.4 (0.7, 5.1)** | **33.0 (9.6, 68.4)** |
| Childhood zinc deficiency | 2.9 (0.7, 10.6) | 0.5 (0.1, 2.0) | 7.2 (1.5, 26.8) |
| Childhood diarrhea | 11.8 (3.9, 21.0) | 2.2 (0.6, 4.7) | 29.8 (8.5, 63.0) |
| Non-exclusive breastfeeding | 1.1 (-1.1, 3.7) | 0.2 (-0.2, 0.8) | 2.9 (-2.3, 10.3) |
| HIV infection without HAART before 2 years | No data | No data | No data |
| Discontinued breastfeeding | 0.7 (0.0, 2.0) | 0.1 (0.0, 0.4) | 1.7 (0.1, 5.4) |
| **Water, sanitation and biomass fuel use** | **2.5 (1.4, 3.6)** | **0.5 (0.2, 0.8)** | **6.3 (2.6, 11.1)** |
| Unimproved sanitation | 2.2 (1.1, 3.3) | 0.4 (0.1, 0.7) | 5.5 (2.0, 9.8) |
| Unimproved water | 0.3 (0.2, 0.6) | 0.1 (0.0, 0.1) | 0.9 (0.3, 1.7) |
| Use of biomass fuels | 0.0 (-0.2, 0.1) | 0.0 (0.0, 0.0) | 0.0 (-0.4, 0.4) |

**Fig 1: Stunting cases among 2-year olds attributable to individual risk factors**


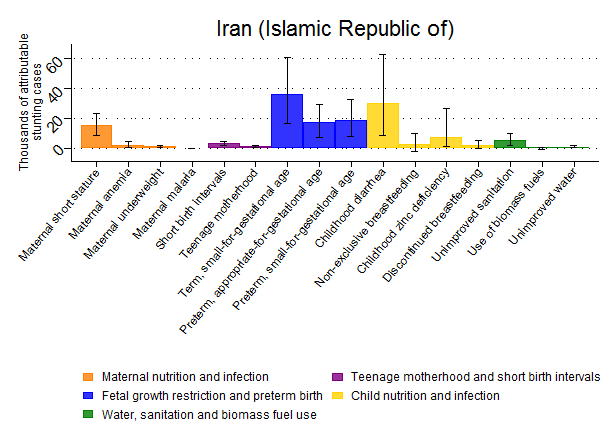


Iraq

**Region: North Africa/Middle East; Sub-region: North Africa / Middle East**

**Stunting prevalence among children age 2: 27.9%**

**Stunting cases among children age 2: 307,710**

**Population of children age 2: 1,104,318**

This country profile provides results for 2011 from the analysis presented in *Risk factors for childhood stunting in 137 developing countries: a comparative risk assessment analysis at global, regional, and country levels.*

**Table 1: Population attributable fraction (PAF in %), attributable stunting prevalence (percentage points) and number of stunting cases among 2-year olds attributable to individual risks and risk factor clusters (95% confidence intervals presented in parentheses)**

| **Description** | **PAF** | **Attributable stunting prevalence** | **Attributable stunting cases (thousands)** |
| --- | --- | --- | --- |
| **Maternal nutrition and infection** | **10.3 (9.2, 11.5)** | **2.9 (1.9, 3.9)** | **31.7 (21.2, 43.3)** |
| Maternal short stature | 9.3 (8.4, 10.1) | 2.6 (1.8, 3.5) | 28.7 (19.5, 39.1) |
| Maternal underweight | 0.3 (0.0, 0.6) | 0.1 (0.0, 0.2) | 0.9 (0.1, 2.0) |
| Maternal malaria | 0.0 (0.0, 0.0) | 0.0 (0.0, 0.0) | 0.0 (0.0, 0.0) |
| Maternal anemia | 0.8 (0.1, 1.7) | 0.2 (0.0, 0.5) | 2.4 (0.4, 5.4) |
| **Teenage motherhood and short birth intervals** | **1.8 (1.7, 1.9)** | **0.5 (0.3, 0.7)** | **5.4 (3.7, 7.3)** |
| Teenage motherhood | 0.5 (0.5, 0.6) | 0.2 (0.1, 0.2) | 1.7 (1.1, 2.2) |
| Short birth intervals | 1.2 (1.1, 1.3) | 0.3 (0.2, 0.5) | 3.8 (2.6, 5.1) |
| **Fetal growth restriction and preterm birth** | **20.2 (14.7, 25.5)** | **5.6 (3.4, 8.2)** | **62.1 (37.1, 90.7)** |
| Preterm, small-for-gestational age | 4.1 (0.6, 7.8) | 1.1 (0.1, 2.3) | 12.6 (1.6, 25.4) |
| Preterm, appropriate-for-gestational age | 3.8 (0.2, 7.2) | 1.1 (0.0, 2.2) | 11.6 (0.4, 23.9) |
| Term, small-for-gestational age | 13.5 (8.4, 19.1) | 3.8 (1.9, 5.9) | 41.5 (21.4, 65.3) |
| Low birth weight | 9.7 (8.0, 11.5) | 2.7 (1.7, 3.8) | 29.9 (18.9, 41.7) |
| **Child nutrition and infection** | **12.1 (4.2, 22.0)** | **3.4 (1.0, 6.8)** | **37.4 (11.5, 75.6)** |
| Childhood zinc deficiency | 0.9 (0.3, 2.4) | 0.2 (0.1, 0.7) | 2.7 (0.8, 7.4) |
| Childhood diarrhea | 11.7 (3.8, 21.7) | 3.3 (1.0, 6.7) | 36.3 (10.8, 73.8) |
| Non-exclusive breastfeeding | 1.7 (-0.3, 4.5) | 0.5 (-0.1, 1.2) | 5.1 (-1.1, 13.7) |
| Discontinued breastfeeding | 1.2 (0.1, 3.3) | 0.3 (0.0, 1.0) | 3.7 (0.4, 10.6) |
| HIV infection without HAART before 2 years | No data | No data | No data |
| **Water, sanitation and biomass fuel use** | **5.0 (2.7, 7.1)** | **1.4 (0.7, 2.3)** | **15.4 (7.5, 25.0)** |
| Unimproved sanitation | 3.9 (1.7, 6.1) | 1.1 (0.4, 1.9) | 12.0 (4.7, 21.0) |
| Unimproved water | 1.1 (0.4, 1.9) | 0.3 (0.1, 0.6) | 3.4 (1.2, 6.4) |
| Use of biomass fuels | 0.0 (-0.1, 0.2) | 0.0 (0.0, 0.1) | 0.1 (-0.5, 0.7) |

**Fig 1: Stunting cases among 2-year olds attributable to individual risk factors**


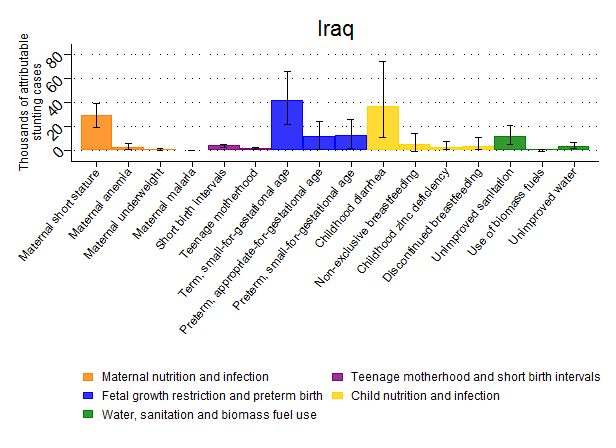


Jamaica

**Region: Latin America/Caribbean; Sub-region: Caribbean**

**Stunting prevalence among children age 2: 3.6%**

**Stunting cases among children age 2: 1,770**

**Population of children age 2: 49,635**

This country profile provides results for 2011 from the analysis presented in *Risk factors for childhood stunting in 137 developing countries: a comparative risk assessment analysis at global, regional, and country levels.*

**Table 1: Population attributable fraction (PAF in %), attributable stunting prevalence (percentage points) and number of stunting cases among 2-year olds attributable to individual risks and risk factor clusters (95% confidence intervals presented in parentheses)**

| **Description** | **PAF** | **Attributable stunting prevalence** | **Attributable stunting cases (thousands)** |
| --- | --- | --- | --- |
| **Maternal nutrition and infection** | **4.9 (3.7, 6.5)** | **0.2 (0.1, 0.3)** | **0.1 (0.0, 0.1)** |
| Maternal short stature | 2.9 (2.8, 3.0) | 0.1 (0.1, 0.2) | 0.1 (0.0, 0.1) |
| Maternal underweight | 0.5 (0.2, 1.0) | 0.0 (0.0, 0.0) | 0.0 (0.0, 0.0) |
| Maternal malaria | 0.0 (0.0, 0.0) | 0.0 (0.0, 0.0) | 0.0 (0.0, 0.0) |
| Maternal anemia | 1.5 (0.3, 3.1) | 0.1 (0.0, 0.1) | 0.0 (0.0, 0.1) |
| **Teenage motherhood and short birth intervals** | **2.3 (2.2, 2.4)** | **0.1 (0.0, 0.1)** | **0.0 (0.0, 0.1)** |
| Teenage motherhood | 1.3 (1.3, 1.4) | 0.0 (0.0, 0.1) | 0.0 (0.0, 0.0) |
| Short birth intervals | 0.9 (0.9, 1.0) | 0.0 (0.0, 0.0) | 0.0 (0.0, 0.0) |
| **Fetal growth restriction and preterm birth** | **25.5 (20.0, 30.3)** | **0.9 (0.5, 1.4)** | **0.5 (0.2, 0.7)** |
| Preterm, small-for-gestational age | 5.5 (1.0, 10.3) | 0.2 (0.0, 0.4) | 0.1 (0.0, 0.2) |
| Preterm, appropriate-for-gestational age | 5.4 (0.9, 10.0) | 0.2 (0.0, 0.4) | 0.1 (0.0, 0.2) |
| Term, small-for-gestational age | 16.6 (12.1, 21.0) | 0.6 (0.3, 0.9) | 0.3 (0.2, 0.5) |
| Low birth weight | 18.9 (15.9, 21.9) | 0.7 (0.4, 1.0) | 0.3 (0.2, 0.5) |
| **Child nutrition and infection** | **19.8 (7.9, 39.0)** | **0.7 (0.2, 1.4)** | **0.4 (0.1, 0.7)** |
| Childhood zinc deficiency | 7.3 (0.9, 43.7) | 0.3 (0.0, 1.6) | 0.1 (0.0, 0.8) |
| Childhood diarrhea | 17.3 (7.1, 27.4) | 0.6 (0.2, 1.1) | 0.3 (0.1, 0.6) |
| Non-exclusive breastfeeding | 2.9 (0.2, 6.3) | 0.1 (0.0, 0.2) | 0.1 (0.0, 0.1) |
| HIV infection without HAART before 2 years | No data | No data | No data |
| Discontinued breastfeeding | 2.3 (0.3, 5.5) | 0.1 (0.0, 0.2) | 0.0 (0.0, 0.1) |
| **Water, sanitation and biomass fuel use** | **7.0 (5.1, 8.9)** | **0.2 (0.1, 0.4)** | **0.1 (0.1, 0.2)** |
| Unimproved sanitation | 5.6 (4.5, 6.8) | 0.2 (0.1, 0.3) | 0.1 (0.1, 0.2) |
| Unimproved water | 0.5 (0.3, 0.8) | 0.0 (0.0, 0.0) | 0.0 (0.0, 0.0) |
| Use of biomass fuels | 0.9 (-0.7, 2.5) | 0.0 (0.0, 0.1) | 0.0 (0.0, 0.0) |

**Fig 1: Stunting cases among 2-year olds attributable to individual risk factors**


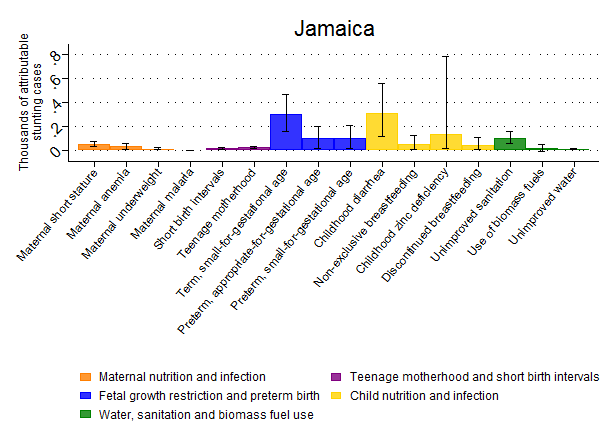


Jordan

**Region: North Africa/Middle East; Sub-region: North Africa / Middle East**

**Stunting prevalence among children age 2: 11.2%**

**Stunting cases among children age 2: 20,522**

**Population of children age 2: 183,374**

This country profile provides results for 2011 from the analysis presented in *Risk factors for childhood stunting in 137 developing countries: a comparative risk assessment analysis at global, regional, and country levels.*

**Table 1: Population attributable fraction (PAF in %), attributable stunting prevalence (percentage points) and number of stunting cases among 2-year olds attributable to individual risks and risk factor clusters (95% confidence intervals presented in parentheses)**

| **Description** | **PAF** | **Attributable stunting prevalence** | **Attributable stunting cases (thousands)** |
| --- | --- | --- | --- |
| **Maternal nutrition and infection** | **6.9 (6.2, 7.7)** | **0.8 (0.5, 1.0)** | **1.4 (1.0, 1.9)** |
| Maternal short stature | 5.8 (5.6, 5.9) | 0.6 (0.5, 0.8) | 1.2 (0.8, 1.5) |
| Maternal underweight | 0.2 (0.0, 0.3) | 0.0 (0.0, 0.0) | 0.0 (0.0, 0.1) |
| Maternal malaria | 0.0 (0.0, 0.0) | 0.0 (0.0, 0.0) | 0.0 (0.0, 0.0) |
| Maternal anemia | 1.0 (0.3, 1.9) | 0.1 (0.0, 0.2) | 0.2 (0.1, 0.4) |
| **Teenage motherhood and short birth intervals** | **1.7 (1.6, 1.8)** | **0.2 (0.1, 0.2)** | **0.3 (0.2, 0.4)** |
| Teenage motherhood | 0.3 (0.2, 0.3) | 0.0 (0.0, 0.0) | 0.1 (0.0, 0.1) |
| Short birth intervals | 1.4 (1.3, 1.5) | 0.2 (0.1, 0.2) | 0.3 (0.2, 0.4) |
| **Fetal growth restriction and preterm birth** | **27.9 (22.5, 33.0)** | **3.1 (2.1, 4.2)** | **5.7 (3.9, 7.7)** |
| Preterm, small-for-gestational age | 7.8 (3.1, 13.4) | 0.9 (0.3, 1.6) | 1.6 (0.6, 2.9) |
| Preterm, appropriate-for-gestational age | 7.3 (3.1, 11.9) | 0.8 (0.3, 1.4) | 1.5 (0.6, 2.6) |
| Term, small-for-gestational age | 15.5 (10.6, 20.6) | 1.7 (1.0, 2.5) | 3.2 (1.9, 4.6) |
| Low birth weight | 13.9 (11.6, 16.3) | 1.6 (1.1, 2.1) | 2.9 (1.9, 3.9) |
| **Child nutrition and infection** | **12.1 (4.1, 22.3)** | **1.4 (0.4, 2.7)** | **2.5 (0.8, 4.9)** |
| Childhood zinc deficiency | 2.9 (0.6, 13.4) | 0.3 (0.1, 1.4) | 0.6 (0.1, 2.5) |
| Childhood diarrhea | 10.8 (3.6, 20.0) | 1.2 (0.4, 2.4) | 2.2 (0.7, 4.4) |
| Non-exclusive breastfeeding | 1.7 (-0.1, 4.2) | 0.2 (0.0, 0.5) | 0.3 (0.0, 0.9) |
| HIV infection without HAART before 2 years | No data | No data | No data |
| Discontinued breastfeeding | 1.4 (0.2, 3.6) | 0.2 (0.0, 0.4) | 0.3 (0.0, 0.8) |
| **Water, sanitation and biomass fuel use** | **1.0 (0.6, 1.4)** | **0.1 (0.1, 0.2)** | **0.2 (0.1, 0.3)** |
| Unimproved sanitation | 0.7 (0.4, 1.1) | 0.1 (0.0, 0.1) | 0.2 (0.1, 0.3) |
| Unimproved water | 0.3 (0.1, 0.4) | 0.0 (0.0, 0.1) | 0.1 (0.0, 0.1) |
| Use of biomass fuels | 0.0 (-0.1, 0.1) | 0.0 (0.0, 0.0) | 0.0 (0.0, 0.0) |

**Fig 1: Stunting cases among 2-year olds attributable to individual risk factors**


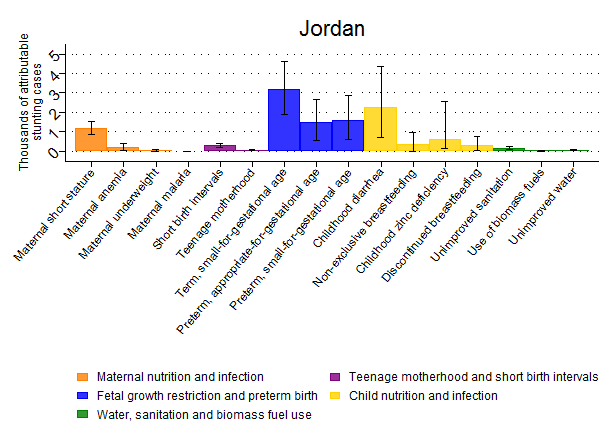


Kazakhstan

**Region: Central Asia Eastern Europe/Central Asia; Sub-region: Asia, Central**

**Stunting prevalence among children age 2: 15.2%**

**Stunting cases among children age 2: 55,230**

**Population of children age 2: 363,231**

This country profile provides results for 2011 from the analysis presented in *Risk factors for childhood stunting in 137 developing countries: a comparative risk assessment analysis at global, regional, and country levels.*

**Table 1: Population attributable fraction (PAF in %), attributable stunting prevalence (percentage points) and number of stunting cases among 2-year olds attributable to individual risks and risk factor clusters (95% confidence intervals presented in parentheses)**

| **Description** | **PAF** | **Attributable stunting prevalence** | **Attributable stunting cases (thousands)** |
| --- | --- | --- | --- |
| **Maternal nutrition and infection** | **5.9 (5.2, 6.9)** | **0.9 (0.5, 1.3)** | **3.3 (1.9, 4.8)** |
| Maternal short stature | 4.7 (4.5, 5.0) | 0.7 (0.4, 1.0) | 2.6 (1.5, 3.7) |
| Maternal underweight | 0.3 (0.0, 0.7) | 0.0 (0.0, 0.1) | 0.2 (0.0, 0.4) |
| Maternal malaria | 0.0 (0.0, 0.0) | 0.0 (0.0, 0.0) | 0.0 (0.0, 0.0) |
| Maternal anemia | 0.9 (0.2, 1.9) | 0.1 (0.0, 0.3) | 0.5 (0.1, 1.2) |
| **Teenage motherhood and short birth intervals** | **1.6 (1.4, 1.8)** | **0.2 (0.1, 0.3)** | **0.9 (0.5, 1.3)** |
| Teenage motherhood | 0.5 (0.4, 0.6) | 0.1 (0.0, 0.1) | 0.3 (0.2, 0.4) |
| Short birth intervals | 1.1 (0.9, 1.2) | 0.2 (0.1, 0.2) | 0.6 (0.3, 0.8) |
| **Fetal growth restriction and preterm birth** | **23.6 (19.2, 28.1)** | **3.6 (2.1, 5.4)** | **13.1 (7.5, 19.5)** |
| Preterm, small-for-gestational age | 5.3 (3.0, 8.1) | 0.8 (0.4, 1.4) | 2.9 (1.5, 5.1) |
| Preterm, appropriate-for-gestational age | 4.8 (2.9, 7.0) | 0.7 (0.4, 1.2) | 2.6 (1.3, 4.5) |
| Term, small-for-gestational age | 15.3 (10.4, 20.9) | 2.3 (1.2, 3.7) | 8.5 (4.3, 13.5) |
| Low birth weight | 12.6 (10.5, 14.8) | 1.9 (1.1, 2.9) | 7.0 (4.1, 10.4) |
| **Child nutrition and infection** | **18.4 (7.8, 29.4)** | **2.8 (1.0, 5.3)** | **10.1 (3.8, 19.1)** |
| Childhood zinc deficiency | 0.5 (0.1, 1.5) | 0.1 (0.0, 0.2) | 0.3 (0.1, 0.9) |
| Childhood diarrhea | 18.2 (7.6, 29.1) | 2.8 (1.0, 5.2) | 10.0 (3.7, 19.0) |
| Non-exclusive breastfeeding | 2.6 (-0.1, 6.0) | 0.4 (0.0, 0.9) | 1.4 (0.0, 3.4) |
| HIV infection without HAART before 2 years | No data | No data | No data |
| Discontinued breastfeeding | 2.1 (0.3, 5.1) | 0.3 (0.0, 0.8) | 1.2 (0.2, 2.9) |
| **Water, sanitation and biomass fuel use** | **1.8 (1.2, 2.5)** | **0.3 (0.1, 0.4)** | **1.0 (0.5, 1.6)** |
| Unimproved sanitation | 1.1 (0.7, 1.5) | 0.2 (0.1, 0.3) | 0.6 (0.3, 0.9) |
| Unimproved water | 0.3 (0.0, 0.6) | 0.0 (0.0, 0.1) | 0.1 (0.0, 0.3) |
| Use of biomass fuels | 0.4 (0.0, 1.0) | 0.1 (0.0, 0.2) | 0.2 (0.0, 0.6) |

**Fig 1: Stunting cases among 2-year olds attributable to individual risk factors**


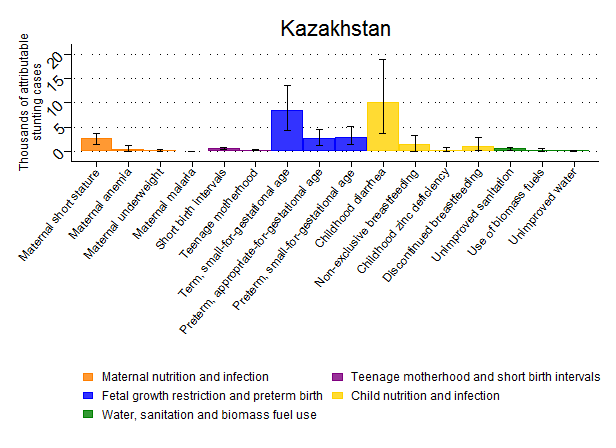


Kenya

**Region: Sub-Saharan Africa; Sub-region: Sub-Saharan Africa, East**

**Stunting prevalence among children age 2: 40.2%**

**Stunting cases among children age 2: 595,579**

**Population of children age 2: 1,481,661**

This country profile provides results for 2011 from the analysis presented in *Risk factors for childhood stunting in 137 developing countries: a comparative risk assessment analysis at global, regional, and country levels.*

**Table 1: Population attributable fraction (PAF in %), attributable stunting prevalence (percentage points) and number of stunting cases among 2-year olds attributable to individual risks and risk factor clusters (95% confidence intervals presented in parentheses)**

| **Description** | **PAF** | **Attributable stunting prevalence** | **Attributable stunting cases (thousands)** |
| --- | --- | --- | --- |
| **Maternal nutrition and infection** | **7.5 (6.5, 8.8)** | **3.0 (2.3, 3.8)** | **44.8 (34.2, 57.0)** |
| Maternal short stature | 5.4 (5.3, 5.6) | 2.2 (1.8, 2.6) | 32.4 (26.1, 38.9) |
| Maternal underweight | 0.9 (0.4, 1.5) | 0.3 (0.2, 0.6) | 5.1 (2.3, 9.2) |
| Maternal malaria | 0.1 (0.1, 0.3) | 0.1 (0.0, 0.1) | 0.9 (0.3, 1.5) |
| Maternal anemia | 1.2 (0.2, 2.5) | 0.5 (0.1, 1.0) | 7.3 (1.4, 15.1) |
| **Teenage motherhood and short birth intervals** | **2.1 (2.0, 2.2)** | **0.8 (0.7, 1.0)** | **12.4 (9.9, 15.0)** |
| Teenage motherhood | 1.1 (1.0, 1.2) | 0.4 (0.3, 0.5) | 6.4 (5.1, 7.7) |
| Short birth intervals | 1.0 (0.9, 1.1) | 0.4 (0.3, 0.5) | 6.1 (4.8, 7.4) |
| **Fetal growth restriction and preterm birth** | **24.4 (20.7, 28.1)** | **9.8 (7.5, 12.2)** | **145.5 (110.4, 180.7)** |
| Preterm, small-for-gestational age | 5.3 (2.8, 8.2) | 2.1 (1.1, 3.4) | 31.4 (15.8, 50.9) |
| Preterm, appropriate-for-gestational age | 7.0 (4.3, 10.1) | 2.8 (1.6, 4.3) | 41.9 (23.6, 64.0) |
| Term, small-for-gestational age | 14.2 (10.7, 17.7) | 5.7 (3.9, 7.5) | 84.5 (57.7, 111.5) |
| Low birth weight | 12.9 (10.7, 15.2) | 5.2 (3.8, 6.6) | 76.9 (56.9, 98.1) |
| **Child nutrition and infection** | **13.2 (5.2, 22.8)** | **5.3 (2.0, 9.4)** | **78.8 (30.0, 138.8)** |
| Childhood zinc deficiency | 1.0 (0.4, 1.9) | 0.4 (0.2, 0.8) | 6.1 (2.3, 11.1) |
| Childhood diarrhea | 12.8 (4.8, 22.5) | 5.1 (1.9, 9.2) | 76.1 (27.4, 136) |
| Non-exclusive breastfeeding | 1.8 (-0.3, 4.7) | 0.7 (-0.1, 1.9) | 10.7 (-1.6, 28.7) |
| HIV infection without HAART before 2 years | 0.5 (0.2, 1.0) | 0.2 (0.1, 0.4) | 2.8 (1.4, 5.9) |
| Discontinued breastfeeding | 0.8 (0.1, 2.3) | 0.3 (0.0, 0.9) | 5.0 (0.5, 13.9) |
| **Water, sanitation and biomass fuel use** | **25.1 (22.8, 27.3)** | **10.1 (7.9, 12.3)** | **149.6 (117.6, 182.3)** |
| Unimproved sanitation | 20.1 (18.0, 22.3) | 8.1 (6.3, 9.9) | 119.7 (93.3, 146.9) |
| Unimproved water | 3.1 (2.0, 4.5) | 1.3 (0.7, 1.9) | 18.7 (11.0, 28.5) |
| Use of biomass fuels | 3.2 (2.3, 4.4) | 1.3 (0.9, 1.8) | 19.3 (12.6, 27.3) |

**Fig 1: Stunting cases among 2-year olds attributable to individual risk factors**


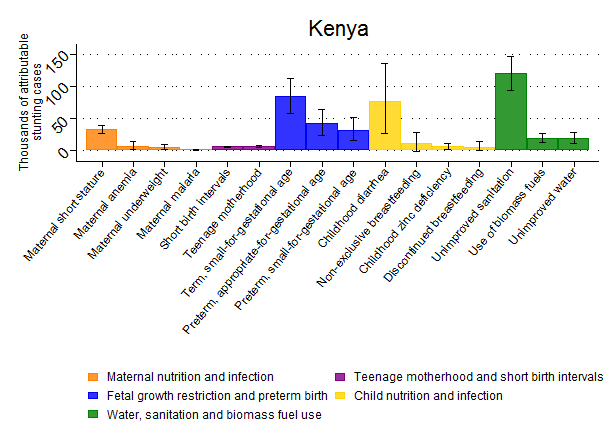


Kiribati

**Region: East Asia/Pacific; Sub-region: Oceania**

**Stunting prevalence among children age 2: 27.8%**

**Stunting cases among children age 2: 837**

**Population of children age 2: 3,010**

This country profile provides results for 2011 from the analysis presented in *Risk factors for childhood stunting in 137 developing countries: a comparative risk assessment analysis at global, regional, and country levels.*

**Table 1: Population attributable fraction (PAF in %), attributable stunting prevalence (percentage points) and number of stunting cases among 2-year olds attributable to individual risks and risk factor clusters (95% confidence intervals presented in parentheses)**

| **Description** | **PAF** | **Attributable stunting prevalence** | **Attributable stunting cases (thousands)** |
| --- | --- | --- | --- |
| **Maternal nutrition and infection** | **7.2 (6.2, 8.4)** | **2.0 (0.9, 3.3)** | **0.1 (0.0, 0.1)** |
| Maternal short stature | 6.0 (5.7, 6.2) | 1.7 (0.7, 2.7) | 0.1 (0.0, 0.1) |
| Maternal underweight | 0.2 (0.0, 0.5) | 0.1 (0.0, 0.1) | 0.0 (0.0, 0.0) |
| Maternal malaria | 0.0 (0.0, 0.0) | 0.0 (0.0, 0.0) | 0.0 (0.0, 0.0) |
| Maternal anemia | 1.1 (0.2, 2.3) | 0.3 (0.0, 0.7) | 0.0 (0.0, 0.0) |
| **Teenage motherhood and short birth intervals** | **1.3 (1.2, 1.4)** | **0.4 (0.2, 0.6)** | **0.0 (0.0, 0.0)** |
| Teenage motherhood | 0.5 (0.4, 0.5) | 0.1 (0.1, 0.2) | 0.0 (0.0, 0.0) |
| Short birth intervals | 0.8 (0.8, 0.9) | 0.2 (0.1, 0.4) | 0.0 (0.0, 0.0) |
| **Fetal growth restriction and preterm birth** | **25.6 (19.7, 30.9)** | **7.1 (3.0, 12.2)** | **0.2 (0.1, 0.4)** |
| Preterm, small-for-gestational age | 5.6 (1.0, 10.6) | 1.5 (0.3, 3.5) | 0.0 (0.0, 0.1) |
| Preterm, appropriate-for-gestational age | 5.2 (1.4, 9.4) | 1.5 (0.3, 3.2) | 0.0 (0.0, 0.1) |
| Term, small-for-gestational age | 16.8 (11.3, 22.1) | 4.7 (1.8, 8.2) | 0.1 (0.1, 0.2) |
| Low birth weight | 13.6 (11.3, 16.0) | 3.8 (1.6, 6.3) | 0.1 (0.0, 0.2) |
| **Child nutrition and infection** | **12.1 (5.2, 19.3)** | **3.4 (1.1, 6.8)** | **0.1 (0.0, 0.2)** |
| Childhood zinc deficiency | 2.0 (0.6, 6.8) | 0.6 (0.1, 1.9) | 0.0 (0.0, 0.1) |
| Childhood diarrhea | 11.2 (4.7, 17.7) | 3.1 (1.0, 6.3) | 0.1 (0.0, 0.2) |
| Non-exclusive breastfeeding | 1.3 (-1.3, 3.8) | 0.4 (-0.3, 1.2) | 0.0 (0.0, 0.0) |
| HIV infection without HAART before 2 years | No data | No data | No data |
| Discontinued breastfeeding | 0.8 (0.1, 2.0) | 0.2 (0.0, 0.6) | 0.0 (0.0, 0.0) |
| **Water, sanitation and biomass fuel use** | **20.8 (17.0, 24.4)** | **5.8 (2.5, 9.6)** | **0.2 (0.1, 0.3)** |
| Unimproved sanitation | 17.4 (13.6, 21.0) | 4.8 (2.1, 8.2) | 0.1 (0.1, 0.2) |
| Unimproved water | 2.1 (0.9, 3.5) | 0.6 (0.2, 1.2) | 0.0 (0.0, 0.0) |
| Use of biomass fuels | 2.1 (1.4, 2.9) | 0.6 (0.2, 1.0) | 0.0 (0.0, 0.0) |

**Fig 1: Stunting cases among 2-year olds attributable to individual risk factors**


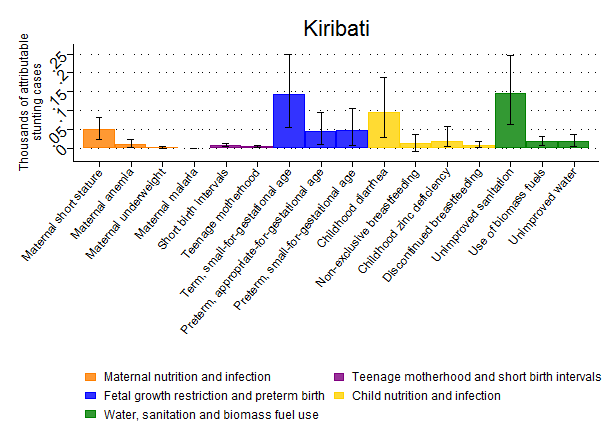


Kuwait

**Region: North Africa/Middle East; Sub-region: North Africa / Middle East**

**Stunting prevalence among children age 2: 2.0%**

**Stunting cases among children age 2: 1,314**

**Population of children age 2: 66,820**

This country profile provides results for 2011 from the analysis presented in *Risk factors for childhood stunting in 137 developing countries: a comparative risk assessment analysis at global, regional, and country levels.*

**Table 1: Population attributable fraction (PAF in %), attributable stunting prevalence (percentage points) and number of stunting cases among 2-year olds attributable to individual risks and risk factor clusters (95% confidence intervals presented in parentheses)**

| **Description** | **PAF** | **Attributable stunting prevalence** | **Attributable stunting cases (thousands)** |
| --- | --- | --- | --- |
| **Maternal nutrition and infection** | **6.2 (5.5, 7.2)** | **0.1 (0.1, 0.2)** | **0.1 (0.1, 0.1)** |
| Maternal short stature | 5.2 (5.0, 5.4) | 0.1 (0.1, 0.1) | 0.1 (0.1, 0.1) |
| Maternal underweight | 0.2 (0.0, 0.3) | 0.0 (0.0, 0.0) | 0.0 (0.0, 0.0) |
| Maternal malaria | 0.0 (0.0, 0.0) | 0.0 (0.0, 0.0) | 0.0 (0.0, 0.0) |
| Maternal anemia | 0.8 (0.2, 1.8) | 0.0 (0.0, 0.0) | 0.0 (0.0, 0.0) |
| **Teenage motherhood and short birth intervals** | **1.8 (1.7, 1.9)** | **0.0 (0.0, 0.0)** | **0.0 (0.0, 0.0)** |
| Teenage motherhood | 0.5 (0.5, 0.6) | 0.0 (0.0, 0.0) | 0.0 (0.0, 0.0) |
| Short birth intervals | 1.2 (1.1, 1.3) | 0.0 (0.0, 0.0) | 0.0 (0.0, 0.0) |
| **Fetal growth restriction and preterm birth** | **24.0 (18.0, 29.7)** | **0.5 (0.3, 0.7)** | **0.3 (0.2, 0.4)** |
| Preterm, small-for-gestational age | 6.4 (1.5, 11.9) | 0.1 (0.0, 0.2) | 0.1 (0.0, 0.2) |
| Preterm, appropriate-for-gestational age | 5.6 (1.2, 10.3) | 0.1 (0.0, 0.2) | 0.1 (0.0, 0.1) |
| Term, small-for-gestational age | 14.0 (9.1, 19.4) | 0.3 (0.2, 0.4) | 0.2 (0.1, 0.3) |
| Low birth weight | 12.8 (10.6, 15.0) | 0.3 (0.2, 0.3) | 0.2 (0.1, 0.2) |
| **Child nutrition and infection** | **8.4 (3.0, 16.3)** | **0.2 (0.1, 0.3)** | **0.1 (0.0, 0.2)** |
| Childhood zinc deficiency | 2.8 (0.3, 18.4) | 0.1 (0.0, 0.3) | 0.0 (0.0, 0.2) |
| Childhood diarrhea | 7.2 (2.7, 11.9) | 0.1 (0.1, 0.3) | 0.1 (0.0, 0.2) |
| Non-exclusive breastfeeding | 1.7 (0.5, 3.5) | 0.0 (0.0, 0.1) | 0.0 (0.0, 0.0) |
| Discontinued breastfeeding | 1.3 (0.2, 2.9) | 0.0 (0.0, 0.1) | 0.0 (0.0, 0.0) |
| HIV infection without HAART before 2 years | No data | No data | No data |
| **Water, sanitation and biomass fuel use** | **0.1 (0.0, 0.2)** | **0.0 (0.0, 0.0)** | **0.0 (0.0, 0.0)** |
| Unimproved sanitation | 0.0 (0.0, 0.0) | 0.0 (0.0, 0.0) | 0.0 (0.0, 0.0) |
| Unimproved water | 0.1 (0.0, 0.2) | 0.0 (0.0, 0.0) | 0.0 (0.0, 0.0) |
| Use of biomass fuels | 0.0 (0.0, 0.0) | 0.0 (0.0, 0.0) | 0.0 (0.0, 0.0) |

**Fig 1: Stunting cases among 2-year olds attributable to individual risk factors**


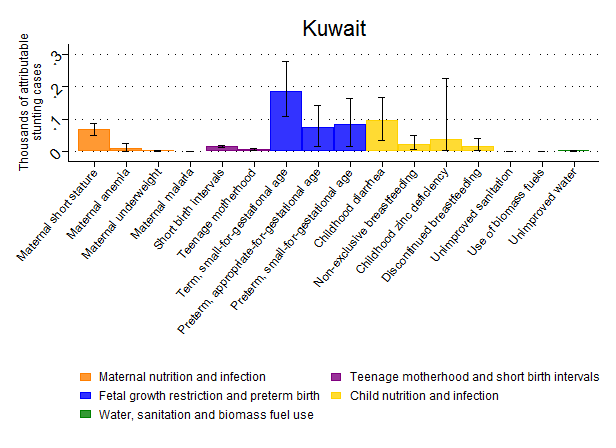


Kyrgyzstan

**Region: Central Asia Eastern Europe/Central Asia; Sub-region: Asia, Central**

**Stunting prevalence among children age 2: 26.6%**

**Stunting cases among children age 2: 37,385**

**Population of children age 2: 140,683**

This country profile provides results for 2011 from the analysis presented in *Risk factors for childhood stunting in 137 developing countries: a comparative risk assessment analysis at global, regional, and country levels.*

**Table 1: Population attributable fraction (PAF in %), attributable stunting prevalence (percentage points) and number of stunting cases among 2-year olds attributable to individual risks and risk factor clusters (95% confidence intervals presented in parentheses)**

| **Description** | **PAF** | **Attributable stunting prevalence** | **Attributable stunting cases (thousands)** |
| --- | --- | --- | --- |
| **Maternal nutrition and infection** | **6.3 (5.5, 7.1)** | **1.7 (1.0, 2.4)** | **2.3 (1.4, 3.3)** |
| Maternal short stature | 5.1 (4.9, 5.3) | 1.4 (0.8, 1.9) | 1.9 (1.1, 2.7) |
| Maternal underweight | 0.3 (0.1, 0.7) | 0.1 (0.0, 0.2) | 0.1 (0.0, 0.3) |
| Maternal malaria | 0.0 (0.0, 0.0) | 0.0 (0.0, 0.0) | 0.0 (0.0, 0.0) |
| Maternal anemia | 0.9 (0.2, 1.6) | 0.2 (0.1, 0.5) | 0.3 (0.1, 0.7) |
| **Teenage motherhood and short birth intervals** | **1.7 (1.5, 1.8)** | **0.4 (0.3, 0.6)** | **0.6 (0.4, 0.9)** |
| Teenage motherhood | 0.5 (0.5, 0.6) | 0.1 (0.1, 0.2) | 0.2 (0.1, 0.3) |
| Short birth intervals | 1.1 (1.0, 1.3) | 0.3 (0.2, 0.4) | 0.4 (0.3, 0.6) |
| **Fetal growth restriction and preterm birth** | **23.2 (17.2, 28.5)** | **6.2 (3.6, 9.4)** | **8.7 (5.0, 13.2)** |
| Preterm, small-for-gestational age | 6.2 (1.3, 11.6) | 1.6 (0.3, 3.4) | 2.3 (0.4, 4.8) |
| Preterm, appropriate-for-gestational age | 5.6 (0.8, 9.8) | 1.5 (0.2, 3.0) | 2.1 (0.3, 4.2) |
| Term, small-for-gestational age | 13.3 (7.7, 18.4) | 3.5 (1.7, 5.6) | 5.0 (2.4, 7.9) |
| Low birth weight | 10.7 (8.8, 12.6) | 2.8 (1.6, 4.1) | 4.0 (2.3, 5.8) |
| **Child nutrition and infection** | **19.1 (8.3, 29.9)** | **5.1 (1.9, 9.3)** | **7.2 (2.6, 13.1)** |
| Childhood zinc deficiency | 0.5 (0.1, 1.3) | 0.1 (0.0, 0.4) | 0.2 (0.0, 0.5) |
| Childhood diarrhea | 18.9 (8.2, 29.6) | 5.1 (1.8, 9.3) | 7.1 (2.6, 13.0) |
| Non-exclusive breastfeeding | 2.0 (-1.3, 5.5) | 0.5 (-0.3, 1.6) | 0.7 (-0.4, 2.3) |
| Discontinued breastfeeding | 1.7 (0.2, 4.2) | 0.5 (0.0, 1.2) | 0.6 (0.1, 1.7) |
| HIV infection without HAART before 2 years | No data | No data | No data |
| **Water, sanitation and biomass fuel use** | **4.3 (3.4, 5.3)** | **1.2 (0.7, 1.7)** | **1.6 (0.9, 2.4)** |
| Unimproved sanitation | 2.2 (1.4, 2.9) | 0.6 (0.3, 0.9) | 0.8 (0.4, 1.3) |
| Unimproved water | 0.9 (0.5, 1.5) | 0.3 (0.1, 0.5) | 0.4 (0.1, 0.6) |
| Use of biomass fuels | 1.3 (0.8, 1.8) | 0.3 (0.2, 0.6) | 0.5 (0.2, 0.8) |

**Fig 1: Stunting cases among 2-year olds attributable to individual risk factors**


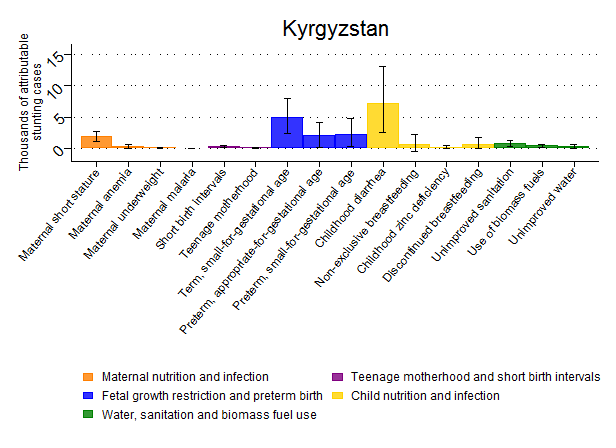


Lao People’s Democratic Republic

**Region: East Asia/Pacific; Sub-region: Asia, Southeast**

**Stunting prevalence among children age 2: 46.7%**

**Stunting cases among children age 2: 81,959**

**Population of children age 2: 175,673**

This country profile provides results for 2011 from the analysis presented in *Risk factors for childhood stunting in 137 developing countries: a comparative risk assessment analysis at global, regional, and country levels.*

**Table 1: Population attributable fraction (PAF in %), attributable stunting prevalence (percentage points) and number of stunting cases among 2-year olds attributable to individual risks and risk factor clusters (95% confidence intervals presented in parentheses)**

| **Description** | **PAF** | **Attributable stunting prevalence** | **Attributable stunting cases (thousands)** |
| --- | --- | --- | --- |
| **Maternal nutrition and infection** | **15.4 (14.1, 16.9)** | **7.2 (5.3, 9.1)** | **12.6 (9.4, 15.9)** |
| Maternal short stature | 12.6 (12.4, 12.9) | 5.9 (4.4, 7.4) | 10.4 (7.7, 13.0) |
| Maternal underweight | 1.5 (0.7, 2.4) | 0.7 (0.3, 1.2) | 1.2 (0.6, 2.1) |
| Maternal malaria | 0.0 (0.0, 0.0) | 0.0 (0.0, 0.0) | 0.0 (0.0, 0.0) |
| Maternal anemia | 1.7 (0.4, 3.1) | 0.8 (0.2, 1.5) | 1.4 (0.4, 2.7) |
| **Teenage motherhood and short birth intervals** | **1.3 (1.2, 1.4)** | **0.6 (0.4, 0.8)** | **1.1 (0.8, 1.3)** |
| Teenage motherhood | 0.5 (0.4, 0.5) | 0.2 (0.2, 0.3) | 0.4 (0.3, 0.5) |
| Short birth intervals | 0.8 (0.8, 0.9) | 0.4 (0.3, 0.5) | 0.7 (0.5, 0.9) |
| **Fetal growth restriction and preterm birth** | **28.6 (22.6, 33.7)** | **13.3 (9.1, 17.6)** | **23.4 (16.0, 30.9)** |
| Preterm, small-for-gestational age | 5.7 (0.6, 11.3) | 2.7 (0.3, 5.5) | 4.7 (0.4, 9.7) |
| Preterm, appropriate-for-gestational age | 5.3 (0.8, 10.2) | 2.5 (0.3, 4.9) | 4.3 (0.6, 8.7) |
| Term, small-for-gestational age | 20.0 (15.0, 25.2) | 9.3 (6.3, 12.8) | 16.4 (11.0, 22.5) |
| Low birth weight | 17.2 (14.4, 20.0) | 8.0 (5.7, 10.7) | 14.1 (10.1, 18.7) |
| **Child nutrition and infection** | **13.1 (5.4, 22.2)** | **6.1 (2.4, 10.6)** | **10.7 (4.2, 18.7)** |
| Childhood zinc deficiency | 0.5 (0.2, 1.0) | 0.2 (0.1, 0.5) | 0.4 (0.2, 0.8) |
| Childhood diarrhea | 12.9 (5.1, 22.0) | 6.0 (2.3, 10.5) | 10.6 (4.1, 18.4) |
| Non-exclusive breastfeeding | 1.9 (-0.3, 4.8) | 0.9 (-0.1, 2.3) | 1.6 (-0.2, 4.1) |
| Discontinued breastfeeding | 0.8 (0.1, 2.1) | 0.4 (0.0, 1.0) | 0.6 (0.0, 1.8) |
| HIV infection without HAART before 2 years | No data | No data | No data |
| **Water, sanitation and biomass fuel use** | **19.0 (16.0, 21.9)** | **8.9 (6.4, 11.6)** | **15.6 (11.3, 20.4)** |
| Unimproved sanitation | 12.6 (9.6, 15.4) | 5.9 (4.1, 8.0) | 10.3 (7.2, 14.0) |
| Unimproved water | 2.6 (1.5, 3.9) | 1.2 (0.7, 1.9) | 2.1 (1.1, 3.4) |
| Use of biomass fuels | 4.8 (3.5, 6.2) | 2.3 (1.5, 3.2) | 4.0 (2.6, 5.7) |

**Fig 1: Stunting cases among 2-year olds attributable to individual risk factors**


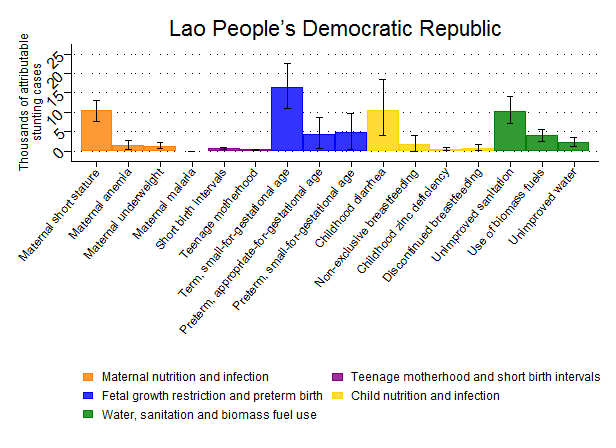


Lebanon

**Region: North Africa/Middle East; Sub-region: North Africa / Middle East**

**Stunting prevalence among children age 2: 12.6%**

**Stunting cases among children age 2: 8,143**

**Population of children age 2: 64,603**

This country profile provides results for 2011 from the analysis presented in *Risk factors for childhood stunting in 137 developing countries: a comparative risk assessment analysis at global, regional, and country levels.*

**Table 1: Population attributable fraction (PAF in %), attributable stunting prevalence (percentage points) and number of stunting cases among 2-year olds attributable to individual risks and risk factor clusters (95% confidence intervals presented in parentheses)**

| **Description** | **PAF** | **Attributable stunting prevalence** | **Attributable stunting cases (thousands)** |
| --- | --- | --- | --- |
| **Maternal nutrition and infection** | **4.7 (4.0, 5.5)** | **0.6 (0.2, 1.0)** | **0.4 (0.1, 0.6)** |
| Maternal short stature | 3.8 (3.5, 4.0) | 0.5 (0.2, 0.8) | 0.3 (0.1, 0.5) |
| Maternal underweight | 0.2 (0.0, 0.4) | 0.0 (0.0, 0.1) | 0.0 (0.0, 0.0) |
| Maternal malaria | 0.0 (0.0, 0.0) | 0.0 (0.0, 0.0) | 0.0 (0.0, 0.0) |
| Maternal anemia | 0.7 (0.2, 1.5) | 0.1 (0.0, 0.2) | 0.1 (0.0, 0.1) |
| **Teenage motherhood and short birth intervals** | **1.8 (1.7, 1.9)** | **0.2 (0.1, 0.4)** | **0.1 (0.1, 0.2)** |
| Teenage motherhood | 0.5 (0.5, 0.6) | 0.1 (0.0, 0.1) | 0.0 (0.0, 0.1) |
| Short birth intervals | 1.2 (1.1, 1.3) | 0.2 (0.1, 0.3) | 0.1 (0.0, 0.2) |
| **Fetal growth restriction and preterm birth** | **20.1 (15.5, 24.6)** | **2.5 (0.9, 4.3)** | **1.6 (0.6, 2.8)** |
| Preterm, small-for-gestational age | 4.8 (2.6, 7.4) | 0.6 (0.2, 1.2) | 0.4 (0.1, 0.7) |
| Preterm, appropriate-for-gestational age | 4.5 (2.8, 6.4) | 0.6 (0.2, 1.1) | 0.4 (0.1, 0.7) |
| Term, small-for-gestational age | 12.2 (7.0, 17.4) | 1.5 (0.5, 2.8) | 1.0 (0.3, 1.8) |
| Low birth weight | 10.4 (8.6, 12.2) | 1.3 (0.5, 2.2) | 0.8 (0.3, 1.4) |
| **Child nutrition and infection** | **13.2 (4.3, 25.8)** | **1.7 (0.4, 4.0)** | **1.1 (0.2, 2.6)** |
| Childhood zinc deficiency | 5.5 (0.7, 29.1) | 0.7 (0.1, 3.8) | 0.4 (0.0, 2.5) |
| Childhood diarrhea | 10.8 (3.8, 20.6) | 1.4 (0.3, 3.1) | 0.9 (0.2, 2.0) |
| Non-exclusive breastfeeding | 2.1 (0.5, 5.0) | 0.3 (0.0, 0.7) | 0.2 (0.0, 0.5) |
| Discontinued breastfeeding | 1.8 (0.2, 4.3) | 0.2 (0.0, 0.6) | 0.1 (0.0, 0.4) |
| HIV infection without HAART before 2 years | No data | No data | No data |
| **Water, sanitation and biomass fuel use** | **1.8 (1.2, 2.4)** | **0.2 (0.1, 0.4)** | **0.1 (0.1, 0.3)** |
| Unimproved sanitation | 1.8 (1.3, 2.4) | 0.2 (0.1, 0.4) | 0.1 (0.1, 0.3) |
| Unimproved water | 0.0 (0.0, 0.0) | 0.0 (0.0, 0.0) | 0.0 (0.0, 0.0) |
| Use of biomass fuels | 0.0 (-0.1, 0.1) | 0.0 (0.0, 0.0) | 0.0 (0.0, 0.0) |

**Fig 1: Stunting cases among 2-year olds attributable to individual risk factors**


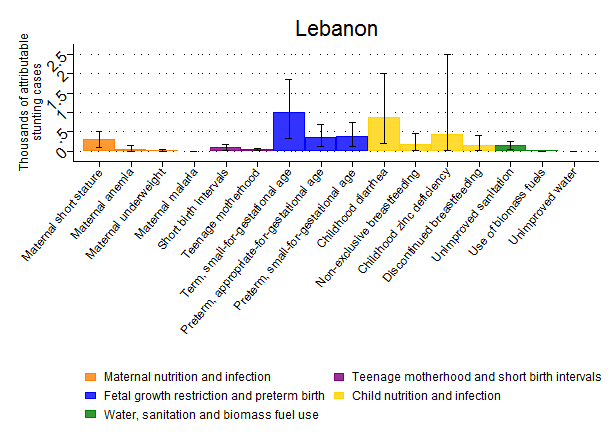


Lesotho

**Region: Sub-Saharan Africa; Sub-region: Sub-Saharan Africa, Southern**

**Stunting prevalence among children age 2: 42.5%**

**Stunting cases among children age 2: 24,621**

**Population of children age 2: 57,884**

This country profile provides results for 2011 from the analysis presented in *Risk factors for childhood stunting in 137 developing countries: a comparative risk assessment analysis at global, regional, and country levels.*

**Table 1: Population attributable fraction (PAF in %), attributable stunting prevalence (percentage points) and number of stunting cases among 2-year olds attributable to individual risks and risk factor clusters (95% confidence intervals presented in parentheses)**

| **Description** | **PAF** | **Attributable stunting prevalence** | **Attributable stunting cases (thousands)** |
| --- | --- | --- | --- |
| **Maternal nutrition and infection** | **9.4 (8.3, 10.7)** | **4.0 (3.2, 4.9)** | **2.3 (1.8, 2.8)** |
| Maternal short stature | 7.2 (7.0, 7.4) | 3.1 (2.5, 3.6) | 1.8 (1.5, 2.1) |
| Maternal underweight | 0.8 (0.3, 1.4) | 0.3 (0.1, 0.6) | 0.2 (0.1, 0.4) |
| Maternal malaria | 0.2 (0.1, 0.3) | 0.1 (0.0, 0.1) | 0.0 (0.0, 0.1) |
| Maternal anemia | 1.4 (0.4, 2.5) | 0.6 (0.2, 1.1) | 0.3 (0.1, 0.7) |
| **Teenage motherhood and short birth intervals** | **1.7 (1.5, 1.8)** | **0.7 (0.6, 0.9)** | **0.4 (0.3, 0.5)** |
| Teenage motherhood | 1.2 (1.1, 1.4) | 0.5 (0.4, 0.6) | 0.3 (0.2, 0.4) |
| Short birth intervals | 0.4 (0.4, 0.5) | 0.2 (0.1, 0.2) | 0.1 (0.1, 0.1) |
| **Fetal growth restriction and preterm birth** | **28.1 (23.6, 32.4)** | **12.0 (9.4, 14.9)** | **6.9 (5.4, 8.6)** |
| Preterm, small-for-gestational age | 4.5 (0.4, 9.0) | 1.9 (0.2, 3.9) | 1.1 (0.1, 2.2) |
| Preterm, appropriate-for-gestational age | 6.5 (1.1, 11.8) | 2.8 (0.5, 5.2) | 1.6 (0.3, 3.0) |
| Term, small-for-gestational age | 19.5 (15.9, 23.2) | 8.3 (6.3, 10.5) | 4.8 (3.7, 6.1) |
| Low birth weight | 16.8 (14.1, 19.6) | 7.2 (5.5, 8.9) | 4.1 (3.2, 5.2) |
| **Child nutrition and infection** | **14.1 (5.7, 24.2)** | **6.0 (2.4, 10.6)** | **3.5 (1.4, 6.1)** |
| Childhood zinc deficiency | 1.4 (0.6, 2.5) | 0.6 (0.2, 1.1) | 0.4 (0.1, 0.6) |
| Childhood diarrhea | 13.5 (5.2, 23.7) | 5.8 (2.2, 10.3) | 3.3 (1.3, 6.0) |
| Non-exclusive breastfeeding | 1.7 (-0.7, 4.5) | 0.7 (-0.3, 1.9) | 0.4 (-0.2, 1.1) |
| Discontinued breastfeeding | 1.0 (0.1, 2.6) | 0.4 (0.0, 1.2) | 0.2 (0.0, 0.7) |
| HIV infection without HAART before 2 years | 1.9 (1.1, 3.6) | 0.8 (0.4, 1.6) | 0.5 (0.3, 0.9) |
| **Water, sanitation and biomass fuel use** | **23.5 (20.9, 26.1)** | **10.0 (7.8, 12.1)** | **5.8 (4.5, 7.0)** |
| Unimproved sanitation | 19.6 (17.0, 22.0) | 8.4 (6.5, 10.2) | 4.8 (3.8, 5.9) |
| Unimproved water | 1.5 (0.8, 2.3) | 0.6 (0.3, 1.0) | 0.4 (0.2, 0.6) |
| Use of biomass fuels | 3.3 (2.3, 4.5) | 1.4 (0.9, 2.0) | 0.8 (0.5, 1.2) |

**Fig 1: Stunting cases among 2-year olds attributable to individual risk factors**


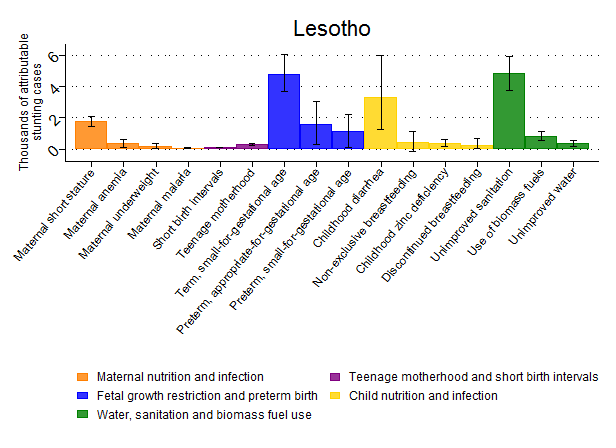


Liberia

**Region: Sub-Saharan Africa; Sub-region: Sub-Saharan Africa, West**

**Stunting prevalence among children age 2: 42.3%**

**Stunting cases among children age 2: 61,545**

**Population of children age 2: 145,428**

This country profile provides results for 2011 from the analysis presented in *Risk factors for childhood stunting in 137 developing countries: a comparative risk assessment analysis at global, regional, and country levels.*

**Table 1: Population attributable fraction (PAF in %), attributable stunting prevalence (percentage points) and number of stunting cases among 2-year olds attributable to individual risks and risk factor clusters (95% confidence intervals presented in parentheses)**

| **Description** | **PAF** | **Attributable stunting prevalence** | **Attributable stunting cases (thousands)** |
| --- | --- | --- | --- |
| **Maternal nutrition and infection** | **13.8 (11.4, 16.3)** | **5.8 (4.1, 7.7)** | **8.5 (6.0, 11.2)** |
| Maternal short stature | 8.0 (7.8, 8.2) | 3.4 (2.5, 4.2) | 4.9 (3.7, 6.1) |
| Maternal underweight | 1.3 (0.5, 2.2) | 0.5 (0.2, 1.0) | 0.8 (0.3, 1.4) |
| Maternal malaria | 2.4 (0.9, 3.9) | 1.0 (0.4, 1.7) | 1.5 (0.5, 2.5) |
| Maternal anemia | 2.8 (0.9, 4.9) | 1.2 (0.4, 2.2) | 1.7 (0.5, 3.1) |
| **Teenage motherhood and short birth intervals** | **2.0 (1.8, 2.1)** | **0.8 (0.6, 1.0)** | **1.2 (0.9, 1.5)** |
| Teenage motherhood | 1.1 (1.0, 1.2) | 0.5 (0.4, 0.6) | 0.7 (0.5, 0.9) |
| Short birth intervals | 0.8 (0.8, 0.9) | 0.4 (0.3, 0.5) | 0.5 (0.4, 0.7) |
| **Fetal growth restriction and preterm birth** | **30.3 (25.1, 34.8)** | **12.8 (9.2, 17.0)** | **18.6 (13.4, 24.7)** |
| Preterm, small-for-gestational age | 5.1 (0.4, 10.1) | 2.2 (0.2, 4.5) | 3.1 (0.2, 6.5) |
| Preterm, appropriate-for-gestational age | 7.0 (1.0, 12.8) | 3.0 (0.4, 5.7) | 4.3 (0.6, 8.3) |
| Term, small-for-gestational age | 20.9 (16.8, 25.3) | 8.9 (6.2, 11.7) | 12.9 (9.1, 17.1) |
| Low birth weight | 20.9 (17.6, 24.1) | 8.8 (6.4, 11.5) | 12.8 (9.4, 16.8) |
| **Child nutrition and infection** | **14.2 (5.2, 24.8)** | **6.0 (2.2, 11.2)** | **8.8 (3.1, 16.3)** |
| Childhood zinc deficiency | 1.2 (0.5, 2.2) | 0.5 (0.2, 1.0) | 0.7 (0.3, 1.4) |
| Childhood diarrhea | 13.7 (4.7, 24.5) | 5.8 (2.0, 11.1) | 8.4 (2.9, 16.1) |
| Non-exclusive breastfeeding | 1.7 (-0.9, 4.9) | 0.7 (-0.4, 2.1) | 1.0 (-0.5, 3.0) |
| HIV infection without HAART before 2 years | 0.1 (0.0, 0.1) | 0.0 (0.0, 0.1) | 0.0 (0.0, 0.1) |
| Discontinued breastfeeding | 0.9 (0.1, 2.4) | 0.4 (0.0, 1.0) | 0.6 (0.1, 1.5) |
| **Water, sanitation and biomass fuel use** | **29.6 (27.1, 32.0)** | **12.5 (9.5, 15.8)** | **18.2 (13.8, 22.9)** |
| Unimproved sanitation | 23.5 (21.4, 25.7) | 9.9 (7.4, 12.6) | 14.5 (10.7, 18.3) |
| Unimproved water | 2.2 (1.3, 3.4) | 0.9 (0.5, 1.5) | 1.4 (0.7, 2.2) |
| Use of biomass fuels | 5.9 (4.2, 7.5) | 2.5 (1.6, 3.5) | 3.6 (2.4, 5.1) |

**Fig 1: Stunting cases among 2-year olds attributable to individual risk factors**


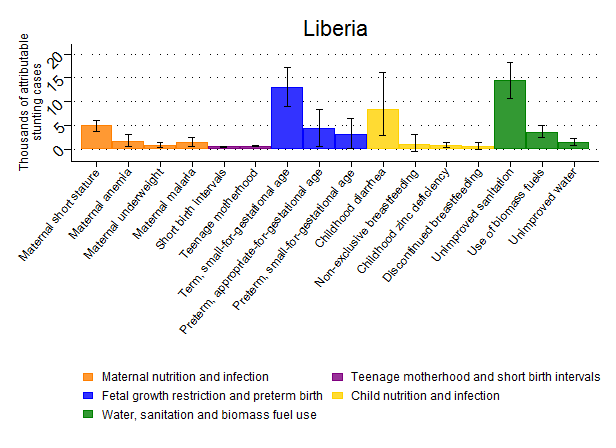


Libyan Arab Jamahiriya

**Region: North Africa/Middle East; Sub-region: North Africa / Middle East**

**Stunting prevalence among children age 2: 17.9%**

**Stunting cases among children age 2: 24,492**

**Population of children age 2: 137,071**

This country profile provides results for 2011 from the analysis presented in *Risk factors for childhood stunting in 137 developing countries: a comparative risk assessment analysis at global, regional, and country levels.*

**Table 1: Population attributable fraction (PAF in %), attributable stunting prevalence (percentage points) and number of stunting cases among 2-year olds attributable to individual risks and risk factor clusters (95% confidence intervals presented in parentheses)**

| **Description** | **PAF** | **Attributable stunting prevalence** | **Attributable stunting cases (thousands)** |
| --- | --- | --- | --- |
| **Maternal nutrition and infection** | **5.4 (4.8, 6.1)** | **1.0 (0.6, 1.4)** | **1.3 (0.8, 1.9)** |
| Maternal short stature | 4.5 (4.2, 4.7) | 0.8 (0.5, 1.1) | 1.1 (0.7, 1.6) |
| Maternal underweight | 0.1 (0.0, 0.3) | 0.0 (0.0, 0.1) | 0.0 (0.0, 0.1) |
| Maternal malaria | 0.3 (0.1, 0.5) | 0.1 (0.0, 0.1) | 0.1 (0.0, 0.1) |
| Maternal anemia | 0.5 (0.1, 1.1) | 0.1 (0.0, 0.2) | 0.1 (0.0, 0.3) |
| **Teenage motherhood and short birth intervals** | **1.8 (1.7, 1.9)** | **0.3 (0.2, 0.4)** | **0.4 (0.3, 0.6)** |
| Teenage motherhood | 0.5 (0.5, 0.6) | 0.1 (0.1, 0.1) | 0.1 (0.1, 0.2) |
| Short birth intervals | 1.2 (1.1, 1.3) | 0.2 (0.1, 0.3) | 0.3 (0.2, 0.4) |
| **Fetal growth restriction and preterm birth** | **15.6 (10.2, 20.0)** | **2.8 (1.4, 4.4)** | **3.8 (1.9, 6.0)** |
| Preterm, small-for-gestational age | 3.8 (0.3, 7.7) | 0.7 (0.1, 1.4) | 0.9 (0.1, 2.0) |
| Preterm, appropriate-for-gestational age | 5.3 (0.9, 9.6) | 1.0 (0.1, 1.9) | 1.3 (0.2, 2.6) |
| Term, small-for-gestational age | 7.4 (4.0, 10.5) | 1.3 (0.6, 2.2) | 1.8 (0.9, 3.1) |
| Low birth weight | 7.2 (5.9, 8.5) | 1.3 (0.7, 1.9) | 1.8 (1.0, 2.6) |
| **Child nutrition and infection** | **12.0 (4.4, 21.7)** | **2.1 (0.7, 4.2)** | **2.9 (0.9, 5.7)** |
| Childhood zinc deficiency | 2.5 (0.6, 8.3) | 0.4 (0.1, 1.4) | 0.6 (0.1, 1.9) |
| Childhood diarrhea | 10.9 (3.8, 19.9) | 1.9 (0.6, 3.8) | 2.7 (0.8, 5.2) |
| Non-exclusive breastfeeding | 1.7 (0.0, 4.2) | 0.3 (0.0, 0.8) | 0.4 (0.0, 1.1) |
| Discontinued breastfeeding | 1.3 (0.1, 3.4) | 0.2 (0.0, 0.6) | 0.3 (0.0, 0.8) |
| HIV infection without HAART before 2 years | No data | No data | No data |
| **Water, sanitation and biomass fuel use** | **3.2 (2.4, 4.1)** | **0.6 (0.3, 0.9)** | **0.8 (0.4, 1.2)** |
| Unimproved sanitation | 1.8 (1.4, 2.2) | 0.3 (0.2, 0.5) | 0.4 (0.3, 0.6) |
| Unimproved water | 1.4 (0.6, 2.3) | 0.2 (0.1, 0.5) | 0.3 (0.1, 0.7) |
| Use of biomass fuels | 0.0 (-0.1, 0.1) | 0.0 (0.0, 0.0) | 0.0 (0.0, 0.0) |

**Fig 1: Stunting cases among 2-year olds attributable to individual risk factors**


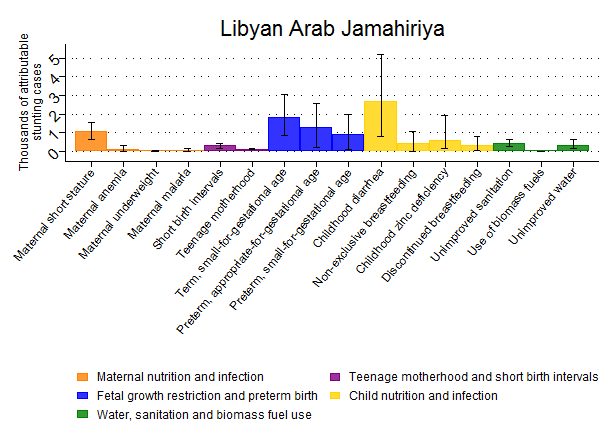


Madagascar

**Region: Sub-Saharan Africa; Sub-region: Sub-Saharan Africa, East**

**Stunting prevalence among children age 2: 52.0%**

**Stunting cases among children age 2: 391,109**

**Population of children age 2: 752,196**

This country profile provides results for 2011 from the analysis presented in *Risk factors for childhood stunting in 137 developing countries: a comparative risk assessment analysis at global, regional, and country levels.*

**Table 1: Population attributable fraction (PAF in %), attributable stunting prevalence (percentage points) and number of stunting cases among 2-year olds attributable to individual risks and risk factor clusters (95% confidence intervals presented in parentheses)**

| **Description** | **PAF** | **Attributable stunting prevalence** | **Attributable stunting cases (thousands)** |
| --- | --- | --- | --- |
| **Maternal nutrition and infection** | **15.1 (13.4, 17.2)** | **7.9 (6.4, 9.7)** | **59.2 (47.9, 72.7)** |
| Maternal short stature | 11.0 (10.7, 11.2) | 5.7 (4.7, 6.8) | 42.9 (35.4, 51.3) |
| Maternal underweight | 2.2 (1.2, 3.6) | 1.2 (0.6, 1.9) | 8.7 (4.5, 14.5) |
| Maternal malaria | 0.4 (0.2, 0.7) | 0.2 (0.1, 0.4) | 1.7 (0.6, 2.9) |
| Maternal anemia | 2.1 (0.6, 3.9) | 1.1 (0.3, 2.0) | 8.2 (2.2, 15.2) |
| **Teenage motherhood and short birth intervals** | **2.5 (2.4, 2.6)** | **1.3 (1.1, 1.5)** | **9.7 (7.9, 11.5)** |
| Teenage motherhood | 1.5 (1.4, 1.6) | 0.8 (0.6, 0.9) | 5.7 (4.7, 6.9) |
| Short birth intervals | 1.0 (0.9, 1.1) | 0.5 (0.4, 0.6) | 4.0 (3.2, 4.8) |
| **Fetal growth restriction and preterm birth** | **31.2 (26.5, 35.8)** | **16.2 (12.5, 20.1)** | **122 (94.2, 150.8)** |
| Preterm, small-for-gestational age | 5.4 (1.2, 9.9) | 2.8 (0.6, 5.3) | 21.0 (4.5, 40.1) |
| Preterm, appropriate-for-gestational age | 7.2 (1.6, 12.6) | 3.7 (0.8, 6.6) | 28.0 (6.0, 50.0) |
| Term, small-for-gestational age | 21.6 (17.3, 26.2) | 11.2 (8.6, 14.6) | 84.6 (64.3, 109.7) |
| Low birth weight | 23.1 (19.6, 26.6) | 12.0 (9.5, 15.0) | 90.3 (71.1, 113) |
| **Child nutrition and infection** | **12.5 (4.6, 22.1)** | **6.5 (2.3, 12.0)** | **48.8 (17.5, 90.5)** |
| Childhood zinc deficiency | 0.5 (0.2, 1.0) | 0.3 (0.1, 0.5) | 2.1 (0.9, 3.8) |
| Childhood diarrhea | 12.2 (4.4, 21.9) | 6.4 (2.2, 12.0) | 47.8 (16.7, 90.1) |
| Non-exclusive breastfeeding | 1.0 (-1.0, 3.3) | 0.5 (-0.5, 1.7) | 4.0 (-4.0, 13.0) |
| HIV infection without HAART before 2 years | 0.0 (0.0, 0.1) | 0.0 (0.0, 0.0) | 0.2 (0.1, 0.3) |
| Discontinued breastfeeding | 0.6 (0.0, 1.5) | 0.3 (0.0, 0.8) | 2.3 (0.2, 6.2) |
| **Water, sanitation and biomass fuel use** | **31.6 (29.2, 34.1)** | **16.4 (13.4, 19.9)** | **123.7 (100.8, 150)** |
| Unimproved sanitation | 23.7 (21.8, 25.7) | 12.3 (10.1, 14.9) | 92.7 (76.0, 112.2) |
| Unimproved water | 4.2 (2.7, 5.8) | 2.2 (1.4, 3.2) | 16.3 (10.2, 23.9) |
| Use of biomass fuels | 6.5 (4.7, 8.3) | 3.4 (2.3, 4.6) | 25.4 (17.6, 34.7) |

**Fig 1: Stunting cases among 2-year olds attributable to individual risk factors**


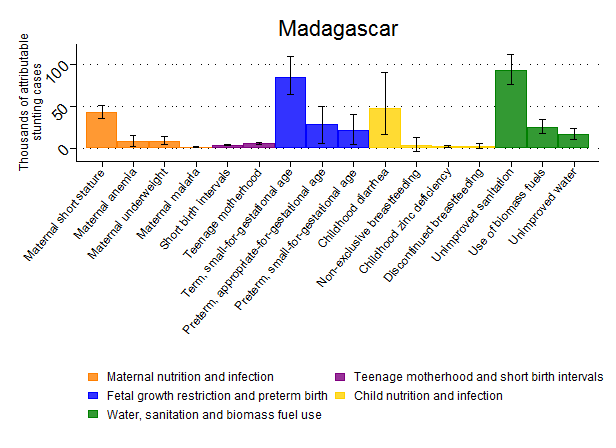


Malawi

**Region: Sub-Saharan Africa; Sub-region: Sub-Saharan Africa, East**

**Stunting prevalence among children age 2: 53.4%**

**Stunting cases among children age 2: 324,733**

**Population of children age 2: 608,676**

This country profile provides results for 2011 from the analysis presented in *Risk factors for childhood stunting in 137 developing countries: a comparative risk assessment analysis at global, regional, and country levels.*

**Table 1: Population attributable fraction (PAF in %), attributable stunting prevalence (percentage points) and number of stunting cases among 2-year olds attributable to individual risks and risk factor clusters (95% confidence intervals presented in parentheses)**

| **Description** | **PAF** | **Attributable stunting prevalence** | **Attributable stunting cases (thousands)** |
| --- | --- | --- | --- |
| **Maternal nutrition and infection** | **13.5 (11.5, 15.6)** | **7.2 (5.9, 8.6)** | **43.8 (36.0, 52.4)** |
| Maternal short stature | 8.6 (8.4, 8.8) | 4.6 (4.0, 5.1) | 27.9 (24.6, 31.1) |
| Maternal underweight | 1.3 (0.6, 2.2) | 0.7 (0.3, 1.2) | 4.2 (1.8, 7.0) |
| Maternal malaria | 2.2 (0.9, 3.6) | 1.2 (0.5, 2.0) | 7.2 (2.8, 12.0) |
| Maternal anemia | 1.9 (0.5, 3.5) | 1.0 (0.3, 1.9) | 6.3 (1.6, 11.3) |
| **Teenage motherhood and short birth intervals** | **1.8 (1.7, 1.9)** | **0.9 (0.8, 1.1)** | **5.8 (5.0, 6.5)** |
| Teenage motherhood | 1.1 (1.0, 1.2) | 0.6 (0.5, 0.7) | 3.6 (3.1, 4.1) |
| Short birth intervals | 0.7 (0.6, 0.7) | 0.4 (0.3, 0.4) | 2.2 (1.9, 2.5) |
| **Fetal growth restriction and preterm birth** | **31.3 (27.5, 35.0)** | **16.7 (14.1, 19.6)** | **101.6 (85.9, 119)** |
| Preterm, small-for-gestational age | 6.7 (3.7, 10.6) | 3.6 (1.9, 5.8) | 21.9 (11.6, 35.3) |
| Preterm, appropriate-for-gestational age | 9.2 (5.8, 13.0) | 4.9 (2.9, 7.1) | 29.8 (17.7, 43.1) |
| Term, small-for-gestational age | 18.8 (15.0, 22.7) | 10.1 (7.7, 12.6) | 61.2 (47.0, 76.4) |
| Low birth weight | 19.4 (16.3, 22.5) | 10.3 (8.4, 12.6) | 63.0 (51.4, 76.8) |
| **Child nutrition and infection** | **14.1 (5.4, 24.4)** | **7.5 (2.8, 13.1)** | **45.7 (17.3, 79.9)** |
| Childhood zinc deficiency | 1.3 (0.5, 2.2) | 0.7 (0.3, 1.2) | 4.3 (1.7, 7.3) |
| Childhood diarrhea | 13.5 (4.8, 23.8) | 7.2 (2.5, 12.9) | 43.8 (15.1, 78.3) |
| Non-exclusive breastfeeding | 1.3 (-1.4, 4.2) | 0.7 (-0.8, 2.3) | 4.3 (-4.6, 14.0) |
| Discontinued breastfeeding | 0.5 (0.0, 1.5) | 0.3 (0.0, 0.8) | 1.6 (0.0, 4.8) |
| HIV infection without HAART before 2 years | 0.7 (0.4, 1.2) | 0.4 (0.2, 0.6) | 2.2 (1.3, 3.8) |
| **Water, sanitation and biomass fuel use** | **21.5 (19.2, 23.6)** | **11.5 (9.7, 13.2)** | **69.7 (59.1, 80.5)** |
| Unimproved sanitation | 15.7 (13.8, 17.7) | 8.4 (7.0, 9.8) | 50.9 (42.3, 59.9) |
| Unimproved water | 1.6 (0.9, 2.3) | 0.8 (0.5, 1.2) | 5.0 (3.0, 7.5) |
| Use of biomass fuels | 5.4 (3.9, 6.9) | 2.9 (2.0, 3.8) | 17.6 (12.4, 23.3) |

**Fig 1: Stunting cases among 2-year olds attributable to individual risk factors**


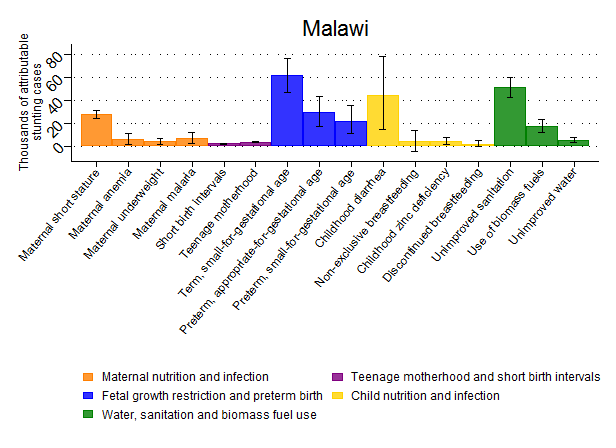


Malaysia

**Region: East Asia/Pacific; Sub-region: Asia, Southeast**

**Stunting prevalence among children age 2: 17.6%**

**Stunting cases among children age 2: 83,837**

**Population of children age 2: 476,462**

This country profile provides results for 2011 from the analysis presented in *Risk factors for childhood stunting in 137 developing countries: a comparative risk assessment analysis at global, regional, and country levels.*

**Table 1: Population attributable fraction (PAF in %), attributable stunting prevalence (percentage points) and number of stunting cases among 2-year olds attributable to individual risks and risk factor clusters (95% confidence intervals presented in parentheses)**

| **Description** | **PAF** | **Attributable stunting prevalence** | **Attributable stunting cases (thousands)** |
| --- | --- | --- | --- |
| **Maternal nutrition and infection** | **10.9 (9.8, 12.3)** | **1.9 (1.2, 2.7)** | **9.2 (5.7, 12.9)** |
| Maternal short stature | 8.8 (8.6, 9.0) | 1.6 (1.0, 2.2) | 7.4 (4.6, 10.4) |
| Maternal underweight | 1.1 (0.5, 1.8) | 0.2 (0.1, 0.4) | 0.9 (0.4, 1.7) |
| Maternal malaria | 0.0 (0.0, 0.0) | 0.0 (0.0, 0.0) | 0.0 (0.0, 0.0) |
| Maternal anemia | 1.2 (0.2, 2.6) | 0.2 (0.0, 0.5) | 1.0 (0.2, 2.3) |
| **Teenage motherhood and short birth intervals** | **1.3 (1.2, 1.4)** | **0.2 (0.1, 0.3)** | **1.1 (0.7, 1.5)** |
| Teenage motherhood | 0.5 (0.4, 0.5) | 0.1 (0.0, 0.1) | 0.4 (0.2, 0.5) |
| Short birth intervals | 0.8 (0.8, 0.9) | 0.1 (0.1, 0.2) | 0.7 (0.4, 1.0) |
| **Fetal growth restriction and preterm birth** | **28.1 (21.6, 33.8)** | **4.9 (2.9, 7.2)** | **23.6 (13.9, 34.3)** |
| Preterm, small-for-gestational age | 6.6 (0.7, 12.6) | 1.2 (0.1, 2.5) | 5.6 (0.6, 11.7) |
| Preterm, appropriate-for-gestational age | 6.2 (1.5, 11.1) | 1.1 (0.3, 2.2) | 5.2 (1.2, 10.4) |
| Term, small-for-gestational age | 17.9 (12.3, 23.4) | 3.1 (1.7, 4.7) | 15.0 (8.3, 22.2) |
| Low birth weight | 17.6 (14.8, 20.5) | 3.1 (1.9, 4.4) | 14.8 (9.1, 21.0) |
| **Child nutrition and infection** | **10.8 (4.3, 18.4)** | **1.9 (0.7, 3.6)** | **9.0 (3.1, 17.2)** |
| Childhood zinc deficiency | 1.9 (0.4, 7.5) | 0.3 (0.1, 1.2) | 1.6 (0.3, 5.9) |
| Childhood diarrhea | 9.9 (3.7, 16.7) | 1.7 (0.6, 3.3) | 8.3 (2.7, 15.7) |
| Non-exclusive breastfeeding | 1.5 (-0.4, 3.7) | 0.3 (-0.1, 0.7) | 1.2 (-0.3, 3.4) |
| Discontinued breastfeeding | 1.3 (0.2, 2.9) | 0.2 (0.0, 0.6) | 1.1 (0.1, 2.7) |
| HIV infection without HAART before 2 years | No data | No data | No data |
| **Water, sanitation and biomass fuel use** | **2.3 (0.5, 4.1)** | **0.4 (0.1, 0.8)** | **1.9 (0.4, 3.9)** |
| Unimproved sanitation | 2.2 (0.3, 4.0) | 0.4 (0.1, 0.8) | 1.8 (0.3, 3.6) |
| Unimproved water | 0.2 (-0.2, 0.5) | 0.0 (0.0, 0.1) | 0.1 (-0.1, 0.5) |
| Use of biomass fuels | 0.0 (-0.3, 0.3) | 0.0 (0.0, 0.0) | 0.0 (-0.2, 0.2) |

**Fig 1: Stunting cases among 2-year olds attributable to individual risk factors**


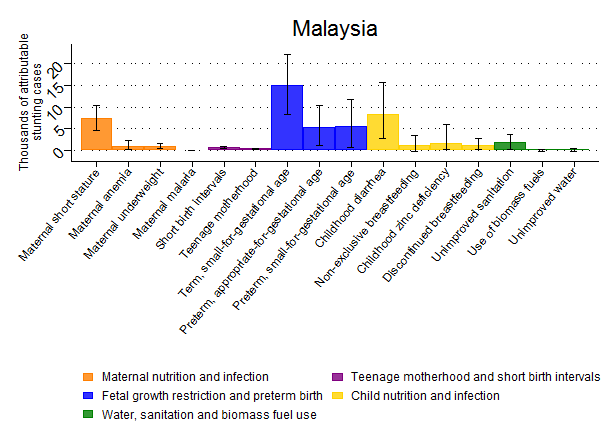


Maldives

**Region: East Asia/Pacific; Sub-region: Asia, Southeast**

**Stunting prevalence among children age 2: 21.0%**

**Stunting cases among children age 2: 1,492**

**Population of children age 2: 7,105**

This country profile provides results for 2011 from the analysis presented in *Risk factors for childhood stunting in 137 developing countries: a comparative risk assessment analysis at global, regional, and country levels.*

**Table 1: Population attributable fraction (PAF in %), attributable stunting prevalence (percentage points) and number of stunting cases among 2-year olds attributable to individual risks and risk factor clusters (95% confidence intervals presented in parentheses)**

| **Description** | **PAF** | **Attributable stunting prevalence** | **Attributable stunting cases (thousands)** |
| --- | --- | --- | --- |
| **Maternal nutrition and infection** | **13.7 (12.3, 15.2)** | **2.9 (1.8, 4.0)** | **0.2 (0.1, 0.3)** |
| Maternal short stature | 11.2 (11.0, 11.5) | 2.4 (1.5, 3.2) | 0.2 (0.1, 0.2) |
| Maternal underweight | 1.1 (0.5, 1.8) | 0.2 (0.1, 0.4) | 0.0 (0.0, 0.0) |
| Maternal malaria | 0.0 (0.0, 0.0) | 0.0 (0.0, 0.0) | 0.0 (0.0, 0.0) |
| Maternal anemia | 1.7 (0.5, 3.2) | 0.4 (0.1, 0.7) | 0.0 (0.0, 0.1) |
| **Teenage motherhood and short birth intervals** | **0.6 (0.6, 0.7)** | **0.1 (0.1, 0.2)** | **0.0 (0.0, 0.0)** |
| Teenage motherhood | 0.2 (0.1, 0.2) | 0.0 (0.0, 0.1) | 0.0 (0.0, 0.0) |
| Short birth intervals | 0.5 (0.4, 0.5) | 0.1 (0.1, 0.1) | 0.0 (0.0, 0.0) |
| **Fetal growth restriction and preterm birth** | **24.8 (20.1, 29.8)** | **5.2 (3.1, 7.5)** | **0.4 (0.2, 0.5)** |
| Preterm, small-for-gestational age | 4.4 (2.3, 6.9) | 0.9 (0.4, 1.6) | 0.1 (0.0, 0.1) |
| Preterm, appropriate-for-gestational age | 4.1 (2.2, 6.0) | 0.9 (0.4, 1.4) | 0.1 (0.0, 0.1) |
| Term, small-for-gestational age | 18.0 (13.0, 23.5) | 3.8 (2.2, 5.7) | 0.3 (0.2, 0.4) |
| Low birth weight | 16.0 (13.4, 18.7) | 3.4 (2.1, 4.9) | 0.2 (0.1, 0.3) |
| **Child nutrition and infection** | **9.5 (3.5, 17.0)** | **2.0 (0.7, 4.0)** | **0.1 (0.0, 0.3)** |
| Childhood zinc deficiency | 0.9 (0.3, 1.9) | 0.2 (0.1, 0.4) | 0.0 (0.0, 0.0) |
| Childhood diarrhea | 9.1 (3.2, 16.6) | 1.9 (0.6, 3.9) | 0.1 (0.0, 0.3) |
| Non-exclusive breastfeeding | 1.0 (-0.8, 3.1) | 0.2 (-0.2, 0.7) | 0.0 (0.0, 0.0) |
| HIV infection without HAART before 2 years | No data | No data | No data |
| Discontinued breastfeeding | 0.7 (0.1, 1.8) | 0.1 (0.0, 0.4) | 0.0 (0.0, 0.0) |
| **Water, sanitation and biomass fuel use** | **3.0 (0.1, 5.8)** | **0.6 (0.0, 1.3)** | **0.0 (0.0, 0.1)** |
| Unimproved sanitation | 2.5 (0.1, 5.0) | 0.5 (0.0, 1.1) | 0.0 (0.0, 0.1) |
| Unimproved water | 0.1 (-0.3, 0.4) | 0.0 (-0.1, 0.1) | 0.0 (0.0, 0.0) |
| Use of biomass fuels | 0.4 (-0.9, 1.6) | 0.1 (-0.2, 0.4) | 0.0 (0.0, 0.0) |

**Fig 1: Stunting cases among 2-year olds attributable to individual risk factors**


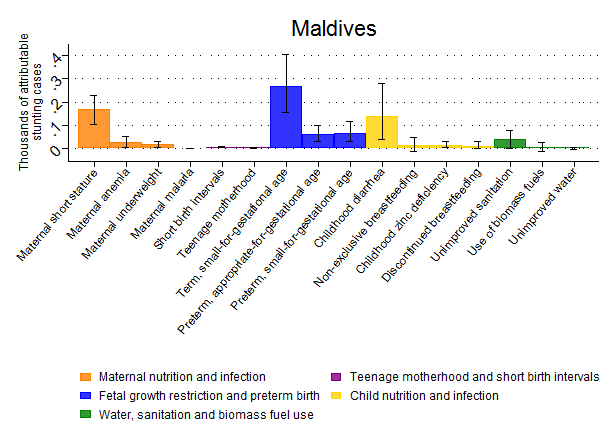


Mali

**Region: Sub-Saharan Africa; Sub-region: Sub-Saharan Africa, West**

**Stunting prevalence among children age 2: 44.7%**

**Stunting cases among children age 2: 310,557**

**Population of children age 2: 694,168**

This country profile provides results for 2011 from the analysis presented in *Risk factors for childhood stunting in 137 developing countries: a comparative risk assessment analysis at global, regional, and country levels.*

**Table 1: Population attributable fraction (PAF in %), attributable stunting prevalence (percentage points) and number of stunting cases among 2-year olds attributable to individual risks and risk factor clusters (95% confidence intervals presented in parentheses)**

| **Description** | **PAF** | **Attributable stunting prevalence** | **Attributable stunting cases (thousands)** |
| --- | --- | --- | --- |
| **Maternal nutrition and infection** | **11.3 (8.4, 14.3)** | **5.0 (3.3, 6.9)** | **35.0 (22.7, 48.2)** |
| Maternal short stature | 3.9 (3.8, 4.1) | 1.8 (1.4, 2.2) | 12.2 (9.4, 15.3) |
| Maternal underweight | 1.9 (1.0, 3.1) | 0.9 (0.4, 1.5) | 6.1 (2.8, 10.3) |
| Maternal malaria | 1.9 (0.7, 3.2) | 0.9 (0.3, 1.5) | 6.0 (2.1, 10.5) |
| Maternal anemia | 4.0 (1.2, 6.6) | 1.8 (0.5, 3.2) | 12.3 (3.4, 22.1) |
| **Teenage motherhood and short birth intervals** | **2.4 (2.3, 2.5)** | **1.1 (0.8, 1.4)** | **7.5 (5.7, 9.5)** |
| Teenage motherhood | 1.3 (1.3, 1.4) | 0.6 (0.5, 0.8) | 4.2 (3.2, 5.2) |
| Short birth intervals | 1.1 (1.0, 1.2) | 0.5 (0.4, 0.6) | 3.4 (2.6, 4.3) |
| **Fetal growth restriction and preterm birth** | **35.7 (31.4, 39.9)** | **16.0 (11.7, 20.5)** | **110.9 (81.3, 142.2)** |
| Preterm, small-for-gestational age | 4.1 (0.6, 8.2) | 1.9 (0.3, 3.8) | 12.9 (1.9, 26.2) |
| Preterm, appropriate-for-gestational age | 5.4 (0.6, 10.6) | 2.4 (0.3, 4.9) | 16.9 (1.8, 33.7) |
| Term, small-for-gestational age | 29.0 (24.0, 34.2) | 13.0 (9.3, 17.1) | 90.2 (64.8, 119) |
| Low birth weight | 26.4 (22.6, 30.3) | 11.8 (8.6, 15.4) | 82.2 (59.7, 106.8) |
| **Child nutrition and infection** | **17.7 (6.9, 31.7)** | **7.9 (2.9, 14.5)** | **55.0 (20.0, 100.4)** |
| Childhood zinc deficiency | 0.9 (0.3, 1.6) | 0.4 (0.1, 0.7) | 2.7 (1.0, 5.1) |
| Childhood diarrhea | 17.3 (6.5, 31.3) | 7.8 (2.8, 14.4) | 53.9 (19.3, 99.8) |
| Non-exclusive breastfeeding | 1.5 (-1.7, 5.0) | 0.7 (-0.8, 2.3) | 4.8 (-5.4, 15.9) |
| HIV infection without HAART before 2 years | 0.1 (0.0, 0.2) | 0.0 (0.0, 0.1) | 0.3 (0.1, 0.6) |
| Discontinued breastfeeding | 0.9 (0.0, 2.3) | 0.4 (0.0, 1.0) | 2.6 (0.2, 7.1) |
| **Water, sanitation and biomass fuel use** | **29.1 (26.4, 31.8)** | **13.0 (9.9, 16.5)** | **90.3 (68.5, 114.4)** |
| Unimproved sanitation | 21.0 (18.7, 23.4) | 9.4 (7.1, 12.1) | 65.3 (49.0, 84.2) |
| Unimproved water | 3.0 (1.7, 4.4) | 1.3 (0.7, 2.1) | 9.2 (5.0, 14.7) |
| Use of biomass fuels | 7.4 (5.3, 9.5) | 3.3 (2.1, 4.6) | 23.1 (14.8, 32.3) |

**Fig 1: Stunting cases among 2-year olds attributable to individual risk factors**


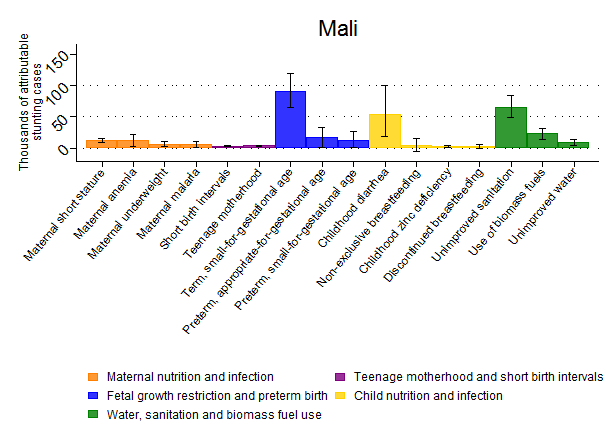


Marshall Islands

**Region: East Asia/Pacific; Sub-region: Oceania**

**Stunting prevalence among children age 2: 20.1%**

**Stunting cases among children age 2: 205**

**Population of children age 2: 1,020**

This country profile provides results for 2011 from the analysis presented in *Risk factors for childhood stunting in 137 developing countries: a comparative risk assessment analysis at global, regional, and country levels.*

**Table 1: Population attributable fraction (PAF in %), attributable stunting prevalence (percentage points) and number of stunting cases among 2-year olds attributable to individual risks and risk factor clusters (95% confidence intervals presented in parentheses)**

| **Description** | **PAF** | **Attributable stunting prevalence** | **Attributable stunting cases (thousands)** |
| --- | --- | --- | --- |
| **Maternal nutrition and infection** | **7.6 (6.1, 9.5)** | **1.5 (0.5, 2.6)** | **0.0 (0.0, 0.0)** |
| Maternal short stature | 5.6 (5.5, 5.8) | 1.1 (0.4, 1.9) | 0.0 (0.0, 0.0) |
| Maternal underweight | 0.4 (0.2, 0.6) | 0.1 (0.0, 0.2) | 0.0 (0.0, 0.0) |
| Maternal malaria | 0.0 (0.0, 0.0) | 0.0 (0.0, 0.0) | 0.0 (0.0, 0.0) |
| Maternal anemia | 1.7 (0.2, 3.7) | 0.3 (0.0, 0.9) | 0.0 (0.0, 0.0) |
| **Teenage motherhood and short birth intervals** | **1.3 (1.2, 1.4)** | **0.3 (0.1, 0.4)** | **0.0 (0.0, 0.0)** |
| Teenage motherhood | 0.5 (0.4, 0.5) | 0.1 (0.0, 0.2) | 0.0 (0.0, 0.0) |
| Short birth intervals | 0.8 (0.8, 0.9) | 0.2 (0.1, 0.3) | 0.0 (0.0, 0.0) |
| **Fetal growth restriction and preterm birth** | **30.7 (25.4, 35.8)** | **6.2 (2.0, 10.4)** | **0.1 (0.0, 0.1)** |
| Preterm, small-for-gestational age | 4.7 (-0.3, 10.1) | 0.9 (0.0, 2.4) | 0.0 (0.0, 0.0) |
| Preterm, appropriate-for-gestational age | 4.4 (0.0, 9.3) | 0.9 (0.0, 2.2) | 0.0 (0.0, 0.0) |
| Term, small-for-gestational age | 24.0 (18.6, 29.6) | 4.8 (1.6, 8.3) | 0.0 (0.0, 0.1) |
| Low birth weight | 22.9 (19.4, 26.3) | 4.6 (1.5, 7.7) | 0.0 (0.0, 0.1) |
| **Child nutrition and infection** | **11.8 (5.1, 19.1)** | **2.4 (0.6, 4.6)** | **0.0 (0.0, 0.0)** |
| Childhood zinc deficiency | 1.5 (0.3, 6.5) | 0.3 (0.0, 1.1) | 0.0 (0.0, 0.0) |
| Childhood diarrhea | 11.2 (4.7, 17.7) | 2.2 (0.6, 4.4) | 0.0 (0.0, 0.0) |
| Non-exclusive breastfeeding | 1.7 (-0.6, 4.1) | 0.3 (-0.1, 0.9) | 0.0 (0.0, 0.0) |
| HIV infection without HAART before 2 years | No data | No data | No data |
| Discontinued breastfeeding | 0.8 (0.1, 1.9) | 0.2 (0.0, 0.4) | 0.0 (0.0, 0.0) |
| **Water, sanitation and biomass fuel use** | **10.5 (8.1, 13.0)** | **2.1 (0.7, 3.7)** | **0.0 (0.0, 0.0)** |
| Unimproved sanitation | 7.9 (6.2, 9.7) | 1.6 (0.5, 2.7) | 0.0 (0.0, 0.0) |
| Unimproved water | 0.3 (-0.4, 1.1) | 0.1 (-0.1, 0.3) | 0.0 (0.0, 0.0) |
| Use of biomass fuels | 2.6 (1.0, 4.2) | 0.5 (0.1, 1.1) | 0.0 (0.0, 0.0) |

**Fig 1: Stunting cases among 2-year olds attributable to individual risk factors**


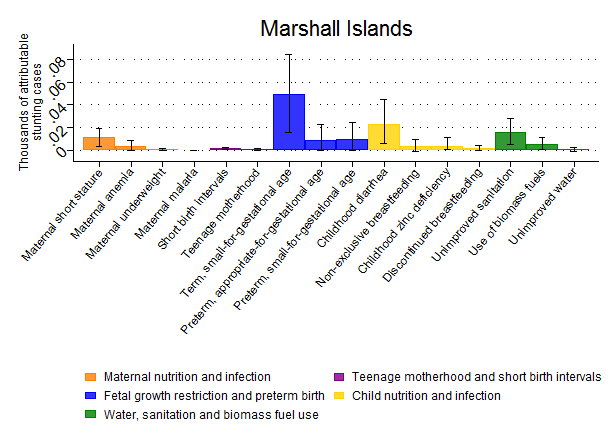


Mauritania

**Region: Sub-Saharan Africa; Sub-region: Sub-Saharan Africa, West**

**Stunting prevalence among children age 2: 32.5%**

**Stunting cases among children age 2: 40,850**

**Population of children age 2: 125,758**

This country profile provides results for 2011 from the analysis presented in *Risk factors for childhood stunting in 137 developing countries: a comparative risk assessment analysis at global, regional, and country levels.*

**Table 1: Population attributable fraction (PAF in %), attributable stunting prevalence (percentage points) and number of stunting cases among 2-year olds attributable to individual risks and risk factor clusters (95% confidence intervals presented in parentheses)**

| **Description** | **PAF** | **Attributable stunting prevalence** | **Attributable stunting cases (thousands)** |
| --- | --- | --- | --- |
| **Maternal nutrition and infection** | **13.4 (9.7, 17.4)** | **4.3 (2.9, 6.0)** | **5.5 (3.6, 7.6)** |
| Maternal short stature | 5.9 (5.5, 6.3) | 1.9 (1.4, 2.4) | 2.4 (1.8, 3.0) |
| Maternal underweight | 2.5 (1.0, 4.4) | 0.8 (0.3, 1.5) | 1.0 (0.4, 1.9) |
| Maternal malaria | 1.1 (0.4, 1.9) | 0.4 (0.1, 0.6) | 0.5 (0.2, 0.8) |
| Maternal anemia | 4.5 (1.2, 8.4) | 1.4 (0.4, 2.8) | 1.8 (0.5, 3.5) |
| **Teenage motherhood and short birth intervals** | **1.9 (1.8, 2.0)** | **0.6 (0.4, 0.8)** | **0.8 (0.6, 1.0)** |
| Teenage motherhood | 1.1 (1.0, 1.1) | 0.4 (0.3, 0.4) | 0.4 (0.3, 0.6) |
| Short birth intervals | 0.8 (0.8, 0.9) | 0.3 (0.2, 0.3) | 0.3 (0.2, 0.4) |
| **Fetal growth restriction and preterm birth** | **41.6 (37.1, 45.9)** | **13.5 (9.5, 17.4)** | **17.0 (12.0, 21.9)** |
| Preterm, small-for-gestational age | 4.8 (1.2, 9.1) | 1.6 (0.4, 3.2) | 2.0 (0.5, 4.0) |
| Preterm, appropriate-for-gestational age | 6.5 (1.4, 11.6) | 2.1 (0.4, 3.9) | 2.6 (0.5, 4.9) |
| Term, small-for-gestational age | 34.4 (28.7, 40.4) | 11.2 (7.8, 14.8) | 14.0 (9.8, 18.6) |
| Low birth weight | 39.3 (34.4, 43.9) | 12.8 (9.0, 16.5) | 16.1 (11.3, 20.8) |
| **Child nutrition and infection** | **13.2 (5.3, 22.6)** | **4.3 (1.6, 7.7)** | **5.4 (2.1, 9.7)** |
| Childhood zinc deficiency | 0.5 (0.2, 1.0) | 0.2 (0.1, 0.3) | 0.2 (0.1, 0.4) |
| Childhood diarrhea | 13.0 (5.1, 22.4) | 4.2 (1.6, 7.6) | 5.3 (2.0, 9.5) |
| Non-exclusive breastfeeding | 2.0 (0.0, 4.9) | 0.6 (0.0, 1.6) | 0.8 (0.0, 2.0) |
| Discontinued breastfeeding | 0.6 (0.0, 1.9) | 0.2 (0.0, 0.6) | 0.3 (0.0, 0.8) |
| HIV infection without HAART before 2 years | 0.1 (0.0, 0.4) | 0.0 (0.0, 0.1) | 0.1 (0.0, 0.2) |
| **Water, sanitation and biomass fuel use** | **29.9 (27.1, 32.8)** | **9.7 (7.0, 12.3)** | **12.2 (8.9, 15.5)** |
| Unimproved sanitation | 21.5 (19.3, 23.9) | 7.0 (5.0, 8.9) | 8.8 (6.3, 11.2) |
| Unimproved water | 3.8 (2.3, 5.5) | 1.2 (0.7, 1.9) | 1.5 (0.9, 2.4) |
| Use of biomass fuels | 7.2 (5.0, 9.6) | 2.3 (1.4, 3.3) | 2.9 (1.8, 4.2) |

**Fig 1: Stunting cases among 2-year olds attributable to individual risk factors**


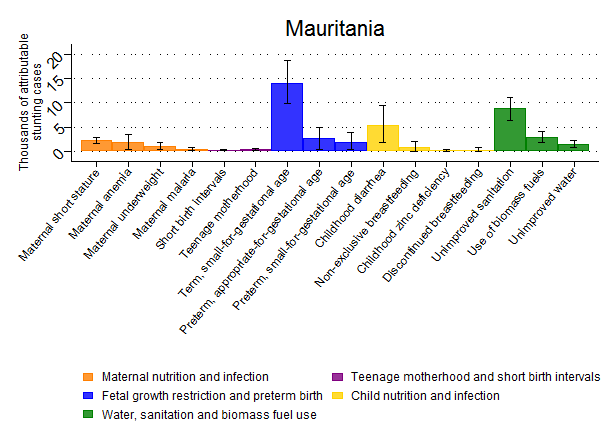


Mauritius

**Region: East Asia/Pacific; Sub-region: Asia, Southeast**

**Stunting prevalence among children age 2: 17.1%**

**Stunting cases among children age 2: 2,619**

**Population of children age 2: 15,347**

This country profile provides results for 2011 from the analysis presented in *Risk factors for childhood stunting in 137 developing countries: a comparative risk assessment analysis at global, regional, and country levels.*

**Table 1: Population attributable fraction (PAF in %), attributable stunting prevalence (percentage points) and number of stunting cases among 2-year olds attributable to individual risks and risk factor clusters (95% confidence intervals presented in parentheses)**

| **Description** | **PAF** | **Attributable stunting prevalence** | **Attributable stunting cases (thousands)** |
| --- | --- | --- | --- |
| **Maternal nutrition and infection** | **10.7 (9.2, 12.4)** | **1.8 (0.8, 2.9)** | **0.3 (0.1, 0.4)** |
| Maternal short stature | 8.3 (8.0, 8.5) | 1.4 (0.6, 2.2) | 0.2 (0.1, 0.3) |
| Maternal underweight | 0.9 (0.3, 1.6) | 0.2 (0.0, 0.3) | 0.0 (0.0, 0.0) |
| Maternal malaria | 0.0 (0.0, 0.0) | 0.0 (0.0, 0.0) | 0.0 (0.0, 0.0) |
| Maternal anemia | 1.7 (0.4, 3.6) | 0.3 (0.1, 0.7) | 0.0 (0.0, 0.1) |
| **Teenage motherhood and short birth intervals** | **1.3 (1.2, 1.4)** | **0.2 (0.1, 0.3)** | **0.0 (0.0, 0.1)** |
| Teenage motherhood | 0.5 (0.4, 0.5) | 0.1 (0.0, 0.1) | 0.0 (0.0, 0.0) |
| Short birth intervals | 0.8 (0.8, 0.9) | 0.1 (0.1, 0.2) | 0.0 (0.0, 0.0) |
| **Fetal growth restriction and preterm birth** | **27.6 (21.7, 32.8)** | **4.7 (2.0, 7.6)** | **0.7 (0.3, 1.2)** |
| Preterm, small-for-gestational age | 4.8 (0.8, 9.5) | 0.8 (0.1, 1.9) | 0.1 (0.0, 0.3) |
| Preterm, appropriate-for-gestational age | 6.9 (1.8, 12.7) | 1.2 (0.3, 2.4) | 0.2 (0.0, 0.4) |
| Term, small-for-gestational age | 18.2 (13.1, 23.4) | 3.1 (1.2, 5.0) | 0.5 (0.2, 0.8) |
| Low birth weight | 21.1 (17.8, 24.4) | 3.6 (1.5, 5.7) | 0.6 (0.2, 0.9) |
| **Child nutrition and infection** | **10.9 (3.3, 22.0)** | **1.9 (0.4, 4.4)** | **0.3 (0.1, 0.7)** |
| Childhood zinc deficiency | 2.7 (0.5, 13.9) | 0.5 (0.1, 2.3) | 0.1 (0.0, 0.4) |
| Childhood diarrhea | 9.6 (2.7, 18.4) | 1.6 (0.4, 3.8) | 0.3 (0.1, 0.6) |
| Non-exclusive breastfeeding | 1.5 (-0.2, 4.0) | 0.2 (-0.1, 0.8) | 0.0 (0.0, 0.1) |
| Discontinued breastfeeding | 1.3 (0.1, 3.3) | 0.2 (0.0, 0.7) | 0.0 (0.0, 0.1) |
| HIV infection without HAART before 2 years | 0.1 (0.0, 0.3) | 0.0 (0.0, 0.0) | 0.0 (0.0, 0.0) |
| **Water, sanitation and biomass fuel use** | **2.9 (1.9, 3.9)** | **0.5 (0.2, 0.9)** | **0.1 (0.0, 0.1)** |
| Unimproved sanitation | 2.9 (2.0, 3.8) | 0.5 (0.2, 0.8) | 0.1 (0.0, 0.1) |
| Unimproved water | 0.0 (0.0, 0.0) | 0.0 (0.0, 0.0) | 0.0 (0.0, 0.0) |
| Use of biomass fuels | 0.0 (-0.3, 0.4) | 0.0 (-0.1, 0.1) | 0.0 (0.0, 0.0) |

**Fig 1: Stunting cases among 2-year olds attributable to individual risk factors**


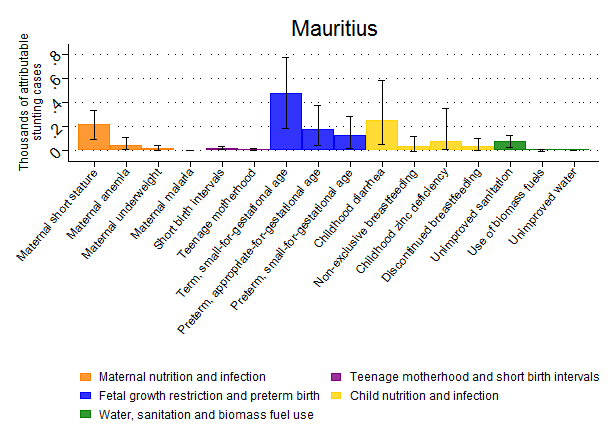


Mexico

**Region: Latin America/Caribbean; Sub-region: Latin America, Central**

**Stunting prevalence among children age 2: 16.5%**

**Stunting cases among children age 2: 392,420**

**Population of children age 2: 2,375,702**

This country profile provides results for 2011 from the analysis presented in *Risk factors for childhood stunting in 137 developing countries: a comparative risk assessment analysis at global, regional, and country levels.*

**Table 1: Population attributable fraction (PAF in %), attributable stunting prevalence (percentage points) and number of stunting cases among 2-year olds attributable to individual risks and risk factor clusters (95% confidence intervals presented in parentheses)**

| **Description** | **PAF** | **Attributable stunting prevalence** | **Attributable stunting cases (thousands)** |
| --- | --- | --- | --- |
| **Maternal nutrition and infection** | **10.2 (9.7, 10.8)** | **1.7 (1.2, 2.2)** | **40.1 (28.4, 52.2)** |
| Maternal short stature | 9.5 (9.3, 9.7) | 1.6 (1.1, 2.0) | 37.3 (26.4, 48.6) |
| Maternal underweight | 0.2 (0.1, 0.3) | 0.0 (0.0, 0.1) | 0.7 (0.2, 1.3) |
| Maternal malaria | 0.0 (0.0, 0.0) | 0.0 (0.0, 0.0) | 0.0 (0.0, 0.0) |
| Maternal anemia | 0.6 (0.2, 1.2) | 0.1 (0.0, 0.2) | 2.5 (0.6, 4.7) |
| **Teenage motherhood and short birth intervals** | **2.5 (2.4, 2.6)** | **0.4 (0.3, 0.5)** | **9.7 (6.9, 12.6)** |
| Teenage motherhood | 1.5 (1.4, 1.6) | 0.2 (0.2, 0.3) | 5.9 (4.1, 7.7) |
| Short birth intervals | 1.0 (0.9, 1.1) | 0.2 (0.1, 0.2) | 3.9 (2.8, 5.1) |
| **Fetal growth restriction and preterm birth** | **17.4 (14.0, 20.9)** | **2.9 (2.0, 3.9)** | **68.2 (46.5, 93.7)** |
| Preterm, small-for-gestational age | 4.4 (3.0, 6.2) | 0.7 (0.4, 1.1) | 17.1 (10.2, 26.0) |
| Preterm, appropriate-for-gestational age | 4.4 (3.3, 5.6) | 0.7 (0.5, 1.0) | 17.3 (11.0, 24.5) |
| Term, small-for-gestational age | 9.6 (5.9, 13.2) | 1.6 (0.9, 2.4) | 37.7 (21.3, 57.3) |
| Low birth weight | 10.5 (8.7, 12.4) | 1.7 (1.2, 2.4) | 41.2 (27.9, 56.2) |
| **Child nutrition and infection** | **17.9 (7.6, 28.3)** | **3.0 (1.1, 5.1)** | **70.1 (27.1, 120)** |
| Childhood zinc deficiency | 1.5 (0.5, 3.6) | 0.3 (0.1, 0.6) | 6.0 (1.9, 14.1) |
| Childhood diarrhea | 17.2 (6.9, 27.5) | 2.8 (1.0, 4.9) | 67.6 (24.9, 117.1) |
| Non-exclusive breastfeeding | 3.9 (1.1, 7.5) | 0.6 (0.2, 1.4) | 15.4 (4.3, 32.2) |
| HIV infection without HAART before 2 years | No data | No data | No data |
| Discontinued breastfeeding | 2.8 (0.5, 6.0) | 0.5 (0.1, 1.1) | 10.9 (1.8, 25.1) |
| **Water, sanitation and biomass fuel use** | **6.2 (4.7, 7.7)** | **1.0 (0.7, 1.5)** | **24.3 (15.9, 34.5)** |
| Unimproved sanitation | 5.2 (3.8, 6.7) | 0.9 (0.6, 1.2) | 20.6 (13.2, 29.5) |
| Unimproved water | 0.4 (0.2, 0.7) | 0.1 (0.0, 0.1) | 1.7 (0.8, 3.0) |
| Use of biomass fuels | 0.6 (0.3, 0.9) | 0.1 (0.0, 0.2) | 2.2 (0.9, 3.8) |

**Fig 1: Stunting cases among 2-year olds attributable to individual risk factors**


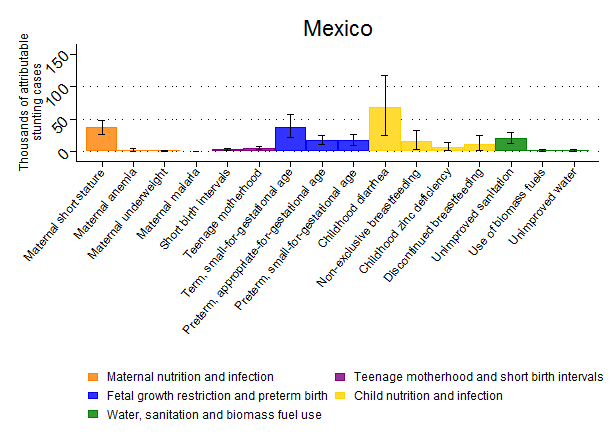


Micronesia (Federated States of)

**Region: East Asia/Pacific; Sub-region: Oceania**

**Stunting prevalence among children age 2: 25.9%**

**Stunting cases among children age 2: 651**

**Population of children age 2: 2,512**

This country profile provides results for 2011 from the analysis presented in *Risk factors for childhood stunting in 137 developing countries: a comparative risk assessment analysis at global, regional, and country levels.*

**Table 1: Population attributable fraction (PAF in %), attributable stunting prevalence (percentage points) and number of stunting cases among 2-year olds attributable to individual risks and risk factor clusters (95% confidence intervals presented in parentheses)**

| **Description** | **PAF** | **Attributable stunting prevalence** | **Attributable stunting cases (thousands)** |
| --- | --- | --- | --- |
| **Maternal nutrition and infection** | **9.0 (7.8, 10.5)** | **2.3 (1.2, 3.6)** | **0.1 (0.0, 0.1)** |
| Maternal short stature | 7.5 (7.2, 7.8) | 1.9 (1.0, 2.9) | 0.0 (0.0, 0.1) |
| Maternal underweight | 0.3 (0.0, 0.6) | 0.1 (0.0, 0.2) | 0.0 (0.0, 0.0) |
| Maternal malaria | 0.0 (0.0, 0.0) | 0.0 (0.0, 0.0) | 0.0 (0.0, 0.0) |
| Maternal anemia | 1.3 (0.1, 2.9) | 0.3 (0.0, 0.8) | 0.0 (0.0, 0.0) |
| **Teenage motherhood and short birth intervals** | **1.3 (1.2, 1.4)** | **0.3 (0.2, 0.5)** | **0.0 (0.0, 0.0)** |
| Teenage motherhood | 0.5 (0.4, 0.5) | 0.1 (0.1, 0.2) | 0.0 (0.0, 0.0) |
| Short birth intervals | 0.8 (0.8, 0.9) | 0.2 (0.1, 0.3) | 0.0 (0.0, 0.0) |
| **Fetal growth restriction and preterm birth** | **29.0 (23.5, 34.1)** | **7.5 (3.6, 11.4)** | **0.2 (0.1, 0.3)** |
| Preterm, small-for-gestational age | 6.1 (1.1, 11.8) | 1.6 (0.2, 3.4) | 0.0 (0.0, 0.1) |
| Preterm, appropriate-for-gestational age | 5.4 (1.1, 10.2) | 1.4 (0.3, 2.9) | 0.0 (0.0, 0.1) |
| Term, small-for-gestational age | 20.0 (15.2, 25.3) | 5.2 (2.6, 8.3) | 0.1 (0.1, 0.2) |
| Low birth weight | 17.6 (14.8, 20.5) | 4.6 (2.3, 6.9) | 0.1 (0.1, 0.2) |
| **Child nutrition and infection** | **11.3 (4.7, 18.8)** | **2.9 (1.0, 5.5)** | **0.1 (0.0, 0.1)** |
| Childhood zinc deficiency | 1.2 (0.3, 3.4) | 0.3 (0.1, 0.9) | 0.0 (0.0, 0.0) |
| Childhood diarrhea | 10.8 (4.4, 18.0) | 2.8 (0.9, 5.4) | 0.1 (0.0, 0.1) |
| Non-exclusive breastfeeding | 1.3 (-0.8, 3.6) | 0.3 (-0.2, 1.0) | 0.0 (0.0, 0.0) |
| HIV infection without HAART before 2 years | No data | No data | No data |
| Discontinued breastfeeding | 0.8 (0.1, 2.1) | 0.2 (0.0, 0.6) | 0.0 (0.0, 0.0) |
| **Water, sanitation and biomass fuel use** | **18.7 (14.3, 23.0)** | **4.9 (2.5, 7.5)** | **0.1 (0.1, 0.2)** |
| Unimproved sanitation | 16.1 (11.7, 20.5) | 4.2 (2.1, 6.6) | 0.1 (0.1, 0.2) |
| Unimproved water | 0.6 (0.1, 1.1) | 0.2 (0.0, 0.3) | 0.0 (0.0, 0.0) |
| Use of biomass fuels | 2.5 (1.5, 3.7) | 0.7 (0.3, 1.2) | 0.0 (0.0, 0.0) |

**Fig 1: Stunting cases among 2-year olds attributable to individual risk factors**


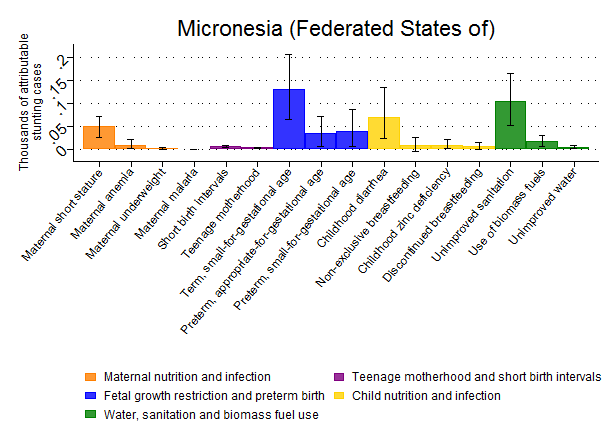


Mongolia

**Region: Central Asia Eastern Europe/Central Asia; Sub-region: Asia, Central**

**Stunting prevalence among children age 2: 25.0%**

**Stunting cases among children age 2: 16,021**

**Population of children age 2: 63,998**

This country profile provides results for 2011 from the analysis presented in *Risk factors for childhood stunting in 137 developing countries: a comparative risk assessment analysis at global, regional, and country levels.*

**Table 1: Population attributable fraction (PAF in %), attributable stunting prevalence (percentage points) and number of stunting cases among 2-year olds attributable to individual risks and risk factor clusters (95% confidence intervals presented in parentheses)**

| **Description** | **PAF** | **Attributable stunting prevalence** | **Attributable stunting cases (thousands)** |
| --- | --- | --- | --- |
| **Maternal nutrition and infection** | **7.0 (6.6, 7.5)** | **1.8 (1.3, 2.2)** | **1.1 (0.8, 1.4)** |
| Maternal short stature | 6.4 (6.2, 6.6) | 1.6 (1.2, 2.0) | 1.0 (0.8, 1.3) |
| Maternal underweight | 0.2 (0.1, 0.4) | 0.1 (0.0, 0.1) | 0.0 (0.0, 0.1) |
| Maternal malaria | 0.0 (0.0, 0.0) | 0.0 (0.0, 0.0) | 0.0 (0.0, 0.0) |
| Maternal anemia | 0.4 (0.1, 1.0) | 0.1 (0.0, 0.3) | 0.1 (0.0, 0.2) |
| **Teenage motherhood and short birth intervals** | **1.6 (1.5, 1.7)** | **0.4 (0.3, 0.5)** | **0.3 (0.2, 0.3)** |
| Teenage motherhood | 0.5 (0.5, 0.6) | 0.1 (0.1, 0.2) | 0.1 (0.1, 0.1) |
| Short birth intervals | 1.0 (1.0, 1.1) | 0.3 (0.2, 0.3) | 0.2 (0.1, 0.2) |
| **Fetal growth restriction and preterm birth** | **22.0 (16.4, 27.2)** | **5.5 (3.7, 7.6)** | **3.5 (2.4, 4.8)** |
| Preterm, small-for-gestational age | 8.1 (3.5, 13.4) | 2.0 (0.8, 3.6) | 1.3 (0.5, 2.3) |
| Preterm, appropriate-for-gestational age | 7.4 (3.4, 11.7) | 1.8 (0.8, 3.1) | 1.2 (0.5, 2.0) |
| Term, small-for-gestational age | 8.3 (3.7, 12.9) | 2.1 (0.9, 3.4) | 1.3 (0.6, 2.2) |
| Low birth weight | 6.8 (5.6, 8.1) | 1.7 (1.2, 2.2) | 1.1 (0.8, 1.4) |
| **Child nutrition and infection** | **11.3 (4.6, 18.8)** | **2.8 (1.1, 4.9)** | **1.8 (0.7, 3.1)** |
| Childhood zinc deficiency | 0.2 (0.1, 0.5) | 0.1 (0.0, 0.1) | 0.0 (0.0, 0.1) |
| Childhood diarrhea | 11.2 (4.4, 18.7) | 2.8 (1.1, 4.9) | 1.8 (0.7, 3.1) |
| Non-exclusive breastfeeding | 0.7 (-1.5, 2.8) | 0.2 (-0.4, 0.7) | 0.1 (-0.2, 0.5) |
| Discontinued breastfeeding | 0.6 (0.1, 1.7) | 0.2 (0.0, 0.4) | 0.1 (0.0, 0.3) |
| HIV infection without HAART before 2 years | No data | No data | No data |
| **Water, sanitation and biomass fuel use** | **11.7 (9.1, 14.4)** | **2.9 (2.0, 4.0)** | **1.9 (1.3, 2.5)** |
| Unimproved sanitation | 9.0 (6.5, 11.7) | 2.3 (1.5, 3.2) | 1.4 (1.0, 2.0) |
| Unimproved water | 1.5 (0.8, 2.3) | 0.4 (0.2, 0.6) | 0.2 (0.1, 0.4) |
| Use of biomass fuels | 1.5 (1.0, 2.0) | 0.4 (0.2, 0.5) | 0.2 (0.1, 0.3) |

**Fig 1: Stunting cases among 2-year olds attributable to individual risk factors**


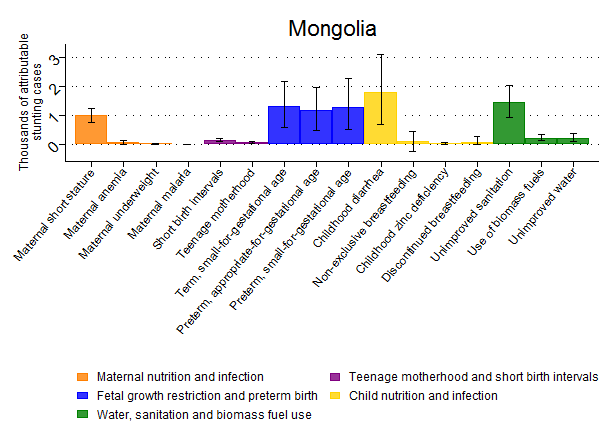


Morocco

**Region: North Africa/Middle East; Sub-region: North Africa / Middle East**

**Stunting prevalence among children age 2: 30.4%**

**Stunting cases among children age 2: 206,355**

**Population of children age 2: 678,324**

This country profile provides results for 2011 from the analysis presented in *Risk factors for childhood stunting in 137 developing countries: a comparative risk assessment analysis at global, regional, and country levels.*

**Table 1: Population attributable fraction (PAF in %), attributable stunting prevalence (percentage points) and number of stunting cases among 2-year olds attributable to individual risks and risk factor clusters (95% confidence intervals presented in parentheses)**

| **Description** | **PAF** | **Attributable stunting prevalence** | **Attributable stunting cases (thousands)** |
| --- | --- | --- | --- |
| **Maternal nutrition and infection** | **7.1 (6.6, 7.7)** | **2.2 (1.2, 3.1)** | **14.7 (8.3, 20.7)** |
| Maternal short stature | 6.2 (6.0, 6.5) | 1.9 (1.1, 2.6) | 12.9 (7.5, 17.8) |
| Maternal underweight | 0.2 (0.1, 0.4) | 0.1 (0.0, 0.1) | 0.4 (0.1, 0.9) |
| Maternal malaria | 0.2 (0.1, 0.3) | 0.0 (0.0, 0.1) | 0.3 (0.1, 0.6) |
| Maternal anemia | 0.6 (0.1, 1.2) | 0.2 (0.0, 0.4) | 1.2 (0.3, 2.7) |
| **Teenage motherhood and short birth intervals** | **1.3 (1.2, 1.3)** | **0.4 (0.2, 0.5)** | **2.6 (1.5, 3.6)** |
| Teenage motherhood | 0.5 (0.5, 0.6) | 0.2 (0.1, 0.2) | 1.1 (0.6, 1.6) |
| Short birth intervals | 0.7 (0.6, 0.8) | 0.2 (0.1, 0.3) | 1.5 (0.8, 2.1) |
| **Fetal growth restriction and preterm birth** | **14.8 (10.1, 19.5)** | **4.5 (2.4, 7.1)** | **30.5 (16.1, 47.9)** |
| Preterm, small-for-gestational age | 3.4 (0.5, 6.9) | 1.0 (0.1, 2.3) | 6.9 (0.9, 15.3) |
| Preterm, appropriate-for-gestational age | 4.4 (1.0, 8.3) | 1.3 (0.2, 2.9) | 9.1 (1.6, 19.4) |
| Term, small-for-gestational age | 7.8 (4.5, 11.2) | 2.4 (1.1, 4.0) | 16.0 (7.2, 27.0) |
| Low birth weight | 6.8 (5.6, 8.1) | 2.1 (1.2, 3.1) | 14.1 (8.2, 20.8) |
| **Child nutrition and infection** | **12.6 (4.5, 23.4)** | **3.8 (1.2, 7.7)** | **25.9 (8.2, 52.3)** |
| Childhood zinc deficiency | 1.3 (0.4, 3.7) | 0.4 (0.1, 1.3) | 2.6 (0.7, 8.5) |
| Childhood diarrhea | 12.0 (4.1, 22.8) | 3.6 (1.1, 7.3) | 24.8 (7.5, 49.8) |
| Non-exclusive breastfeeding | 1.8 (-0.3, 4.8) | 0.6 (-0.1, 1.5) | 3.7 (-0.6, 10.3) |
| Discontinued breastfeeding | 1.5 (0.2, 3.7) | 0.4 (0.1, 1.2) | 3.0 (0.3, 8.2) |
| HIV infection without HAART before 2 years | No data | No data | No data |
| **Water, sanitation and biomass fuel use** | **7.5 (5.3, 9.6)** | **2.3 (1.2, 3.5)** | **15.5 (8.3, 23.8)** |
| Unimproved sanitation | 6.2 (4.0, 8.3) | 1.9 (0.9, 3.0) | 12.9 (6.4, 20.0) |
| Unimproved water | 1.3 (0.7, 2.0) | 0.4 (0.2, 0.7) | 2.7 (1.2, 4.8) |
| Use of biomass fuels | 0.1 (-0.1, 0.2) | 0.0 (0.0, 0.1) | 0.2 (-0.2, 0.5) |

**Fig 1: Stunting cases among 2-year olds attributable to individual risk factors**


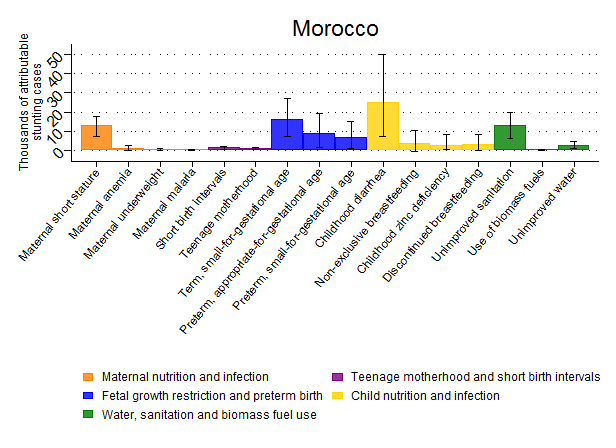


Mozambique

**Region: Sub-Saharan Africa; Sub-region: Sub-Saharan Africa, East**

**Stunting prevalence among children age 2: 47.1%**

**Stunting cases among children age 2: 470,875**

**Population of children age 2: 1,000,108**

This country profile provides results for 2011 from the analysis presented in *Risk factors for childhood stunting in 137 developing countries: a comparative risk assessment analysis at global, regional, and country levels.*

**Table 1: Population attributable fraction (PAF in %), attributable stunting prevalence (percentage points) and number of stunting cases among 2-year olds attributable to individual risks and risk factor clusters (95% confidence intervals presented in parentheses)**

| **Description** | **PAF** | **Attributable stunting prevalence** | **Attributable stunting cases (thousands)** |
| --- | --- | --- | --- |
| **Maternal nutrition and infection** | **14.5 (12.4, 16.8)** | **6.8 (5.2, 8.6)** | **68.4 (52.2, 85.8)** |
| Maternal short stature | 8.9 (8.7, 9.1) | 4.2 (3.4, 4.9) | 41.8 (34.1, 49.1) |
| Maternal underweight | 1.6 (0.8, 2.7) | 0.8 (0.4, 1.3) | 7.7 (3.5, 13.1) |
| Maternal malaria | 1.9 (0.7, 3.2) | 0.9 (0.3, 1.5) | 9.0 (3.4, 14.9) |
| Maternal anemia | 2.8 (0.8, 4.8) | 1.3 (0.4, 2.3) | 13.1 (3.9, 23.4) |
| **Teenage motherhood and short birth intervals** | **2.0 (1.9, 2.1)** | **0.9 (0.7, 1.1)** | **9.2 (7.4, 10.9)** |
| Teenage motherhood | 1.4 (1.3, 1.4) | 0.6 (0.5, 0.8) | 6.4 (5.1, 7.6) |
| Short birth intervals | 0.6 (0.6, 0.7) | 0.3 (0.2, 0.3) | 2.9 (2.3, 3.5) |
| **Fetal growth restriction and preterm birth** | **33.1 (29.1, 36.8)** | **15.6 (12.2, 18.9)** | **155.9 (121.9, 188.6)** |
| Preterm, small-for-gestational age | 5.8 (2.4, 9.9) | 2.7 (1.1, 4.8) | 27.1 (10.7, 48.5) |
| Preterm, appropriate-for-gestational age | 8.1 (4.1, 12.4) | 3.8 (1.8, 6.1) | 38.3 (18.2, 60.5) |
| Term, small-for-gestational age | 22.7 (18.9, 27.1) | 10.7 (8.1, 13.3) | 106.9 (81.3, 132.9) |
| Low birth weight | 22.6 (19.2, 26.1) | 10.6 (8.2, 13.0) | 106.5 (82.2, 130.4) |
| **Child nutrition and infection** | **16.0 (6.4, 27.5)** | **7.5 (3.0, 12.9)** | **75.2 (30.3, 129.2)** |
| Childhood zinc deficiency | 1.1 (0.4, 1.9) | 0.5 (0.2, 0.9) | 5.2 (2.0, 9.3) |
| Childhood diarrhea | 15.5 (5.9, 26.8) | 7.3 (2.8, 12.8) | 73.0 (28.0, 127.8) |
| Non-exclusive breastfeeding | 1.6 (-1.2, 4.9) | 0.8 (-0.6, 2.3) | 7.6 (-5.9, 23.3) |
| HIV infection without HAART before 2 years | 0.9 (0.5, 1.6) | 0.4 (0.2, 0.8) | 4.1 (2.2, 7.7) |
| Discontinued breastfeeding | 1.1 (0.1, 3.2) | 0.5 (0.0, 1.5) | 5.3 (0.4, 15.0) |
| **Water, sanitation and biomass fuel use** | **30.8 (28.2, 33.4)** | **14.5 (11.7, 17.4)** | **145.2 (116.9, 174.3)** |
| Unimproved sanitation | 23.1 (20.9, 25.3) | 10.9 (8.7, 13.0) | 108.7 (87.1, 130.1) |
| Unimproved water | 4.1 (2.6, 5.8) | 1.9 (1.2, 2.8) | 19.2 (12.0, 27.9) |
| Use of biomass fuels | 6.3 (4.5, 8.0) | 2.9 (2.0, 4.0) | 29.5 (20.2, 40.1) |

**Fig 1: Stunting cases among 2-year olds attributable to individual risk factors**


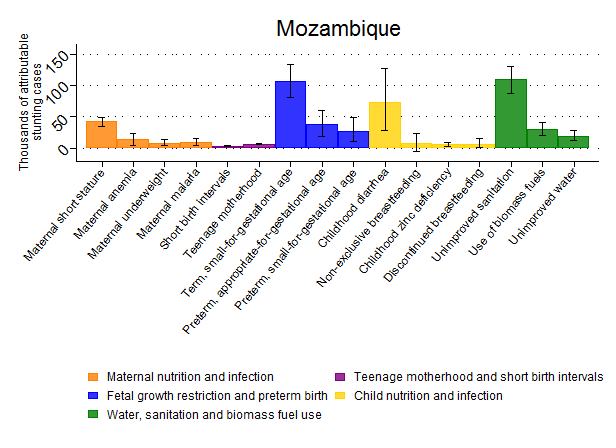


Myanmar

**Region: East Asia/Pacific; Sub-region: Asia, Southeast**

**Stunting prevalence among children age 2: 44.1%**

**Stunting cases among children age 2: 450,211**

**Population of children age 2: 1,020,722**

This country profile provides results for 2011 from the analysis presented in *Risk factors for childhood stunting in 137 developing countries: a comparative risk assessment analysis at global, regional, and country levels.*

**Table 1: Population attributable fraction (PAF in %), attributable stunting prevalence (percentage points) and number of stunting cases among 2-year olds attributable to individual risks and risk factor clusters (95% confidence intervals presented in parentheses)**

| **Description** | **PAF** | **Attributable stunting prevalence** | **Attributable stunting cases (thousands)** |
| --- | --- | --- | --- |
| **Maternal nutrition and infection** | **13.7 (11.9, 15.7)** | **6.1 (4.3, 8.1)** | **61.8 (44.0, 82.6)** |
| Maternal short stature | 10.2 (9.9, 10.4) | 4.5 (3.3, 5.8) | 45.7 (33.2, 59.5) |
| Maternal underweight | 2.0 (1.0, 3.2) | 0.9 (0.4, 1.5) | 9.1 (4.2, 15.6) |
| Maternal malaria | 0.0 (0.0, 0.0) | 0.0 (0.0, 0.0) | 0.0 (0.0, 0.0) |
| Maternal anemia | 2.0 (0.5, 3.8) | 0.9 (0.2, 1.7) | 9.0 (2.2, 17.8) |
| **Teenage motherhood and short birth intervals** | **1.3 (1.2, 1.4)** | **0.6 (0.4, 0.7)** | **5.8 (4.2, 7.5)** |
| Teenage motherhood | 0.5 (0.4, 0.5) | 0.2 (0.1, 0.3) | 2.0 (1.5, 2.7) |
| Short birth intervals | 0.8 (0.8, 0.9) | 0.4 (0.3, 0.5) | 3.8 (2.7, 5.0) |
| **Fetal growth restriction and preterm birth** | **34.4 (29.5, 38.7)** | **15.2 (10.7, 20.3)** | **155 (109.2, 207.3)** |
| Preterm, small-for-gestational age | 6.2 (1.2, 11.4) | 2.7 (0.6, 5.3) | 27.7 (5.7, 54.2) |
| Preterm, appropriate-for-gestational age | 5.5 (0.8, 9.9) | 2.4 (0.3, 4.7) | 24.8 (3.3, 48.0) |
| Term, small-for-gestational age | 26.0 (21.1, 30.8) | 11.5 (7.6, 15.7) | 117.1 (77.6, 159.8) |
| Low birth weight | 22.4 (19.0, 25.8) | 9.9 (7.0, 13.6) | 100.9 (71.1, 138.4) |
| **Child nutrition and infection** | **11.3 (4.6, 19.6)** | **5.0 (1.9, 9.3)** | **51.0 (19.4, 94.8)** |
| Childhood zinc deficiency | 0.8 (0.3, 1.5) | 0.3 (0.1, 0.7) | 3.5 (1.3, 6.7) |
| Childhood diarrhea | 11.0 (4.2, 19.2) | 4.8 (1.8, 9.2) | 49.4 (18.0, 93.7) |
| Non-exclusive breastfeeding | 1.5 (-0.2, 3.7) | 0.6 (-0.1, 1.7) | 6.6 (-1.1, 17.4) |
| HIV infection without HAART before 2 years | No data | No data | No data |
| Discontinued breastfeeding | 0.5 (0.0, 1.4) | 0.2 (0.0, 0.6) | 2.4 (0.2, 6.3) |
| **Water, sanitation and biomass fuel use** | **14.1 (11.1, 17.1)** | **6.2 (4.3, 8.6)** | **63.6 (44.0, 88.1)** |
| Unimproved sanitation | 7.1 (4.7, 9.6) | 3.2 (1.9, 4.7) | 32.2 (18.9, 47.6) |
| Unimproved water | 1.6 (0.8, 2.4) | 0.7 (0.3, 1.1) | 7.0 (3.4, 11.7) |
| Use of biomass fuels | 6.0 (4.3, 7.8) | 2.7 (1.7, 3.9) | 27.3 (17.1, 40.3) |

**Fig 1: Stunting cases among 2-year olds attributable to individual risk factors**


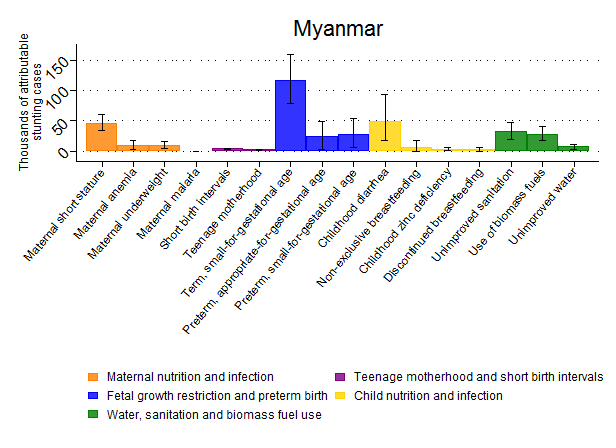


Namibia

**Region: Sub-Saharan Africa; Sub-region: Sub-Saharan Africa, Southern**

**Stunting prevalence among children age 2: 36.0%**

**Stunting cases among children age 2: 23,803**

**Population of children age 2: 66,201**

This country profile provides results for 2011 from the analysis presented in *Risk factors for childhood stunting in 137 developing countries: a comparative risk assessment analysis at global, regional, and country levels.*

**Table 1: Population attributable fraction (PAF in %), attributable stunting prevalence (percentage points) and number of stunting cases among 2-year olds attributable to individual risks and risk factor clusters (95% confidence intervals presented in parentheses)**

| **Description** | **PAF** | **Attributable stunting prevalence** | **Attributable stunting cases (thousands)** |
| --- | --- | --- | --- |
| **Maternal nutrition and infection** | **8.3 (6.6, 10.7)** | **3.0 (2.0, 4.2)** | **2.0 (1.3, 2.8)** |
| Maternal short stature | 4.8 (4.6, 4.9) | 1.7 (1.2, 2.2) | 1.1 (0.8, 1.5) |
| Maternal underweight | 1.5 (0.7, 2.5) | 0.5 (0.2, 0.9) | 0.3 (0.1, 0.6) |
| Maternal malaria | 0.3 (0.1, 0.4) | 0.1 (0.0, 0.2) | 0.1 (0.0, 0.1) |
| Maternal anemia | 2.0 (0.5, 4.2) | 0.7 (0.2, 1.6) | 0.5 (0.1, 1.0) |
| **Teenage motherhood and short birth intervals** | **1.5 (1.4, 1.7)** | **0.6 (0.4, 0.7)** | **0.4 (0.3, 0.5)** |
| Teenage motherhood | 1.0 (0.9, 1.1) | 0.4 (0.3, 0.5) | 0.2 (0.2, 0.3) |
| Short birth intervals | 0.5 (0.5, 0.6) | 0.2 (0.1, 0.2) | 0.1 (0.1, 0.2) |
| **Fetal growth restriction and preterm birth** | **30.7 (25.3, 35.5)** | **11.0 (7.5, 14.6)** | **7.3 (5.0, 9.6)** |
| Preterm, small-for-gestational age | 5.4 (1.1, 10.2) | 1.9 (0.4, 3.9) | 1.3 (0.3, 2.6) |
| Preterm, appropriate-for-gestational age | 7.5 (1.6, 13.3) | 2.7 (0.6, 5.0) | 1.8 (0.4, 3.3) |
| Term, small-for-gestational age | 20.8 (16.4, 26.0) | 7.5 (4.9, 10.4) | 4.9 (3.3, 6.9) |
| Low birth weight | 23.2 (19.7, 26.7) | 8.3 (5.9, 11.0) | 5.5 (3.9, 7.3) |
| **Child nutrition and infection** | **11.8 (4.3, 21.1)** | **4.2 (1.5, 7.9)** | **2.8 (1.0, 5.3)** |
| Childhood zinc deficiency | 0.9 (0.3, 1.6) | 0.3 (0.1, 0.6) | 0.2 (0.1, 0.4) |
| Childhood diarrhea | 11.4 (3.9, 20.7) | 4.1 (1.4, 7.8) | 2.7 (0.9, 5.2) |
| Non-exclusive breastfeeding | 1.8 (-0.1, 4.7) | 0.7 (0.0, 1.7) | 0.4 (0.0, 1.2) |
| HIV infection without HAART before 2 years | 0.5 (0.2, 1.1) | 0.2 (0.1, 0.4) | 0.1 (0.0, 0.3) |
| Discontinued breastfeeding | 1.2 (0.2, 3.0) | 0.4 (0.0, 1.1) | 0.3 (0.0, 0.8) |
| **Water, sanitation and biomass fuel use** | **22.6 (19.9, 25.3)** | **8.1 (5.7, 10.6)** | **5.4 (3.8, 7.0)** |
| Unimproved sanitation | 18.6 (16.0, 21.2) | 6.7 (4.7, 8.8) | 4.4 (3.1, 5.8) |
| Unimproved water | 0.8 (0.3, 1.4) | 0.3 (0.1, 0.5) | 0.2 (0.1, 0.3) |
| Use of biomass fuels | 4.2 (2.8, 5.6) | 1.5 (0.9, 2.2) | 1.0 (0.6, 1.5) |

**Fig 1: Stunting cases among 2-year olds attributable to individual risk factors**


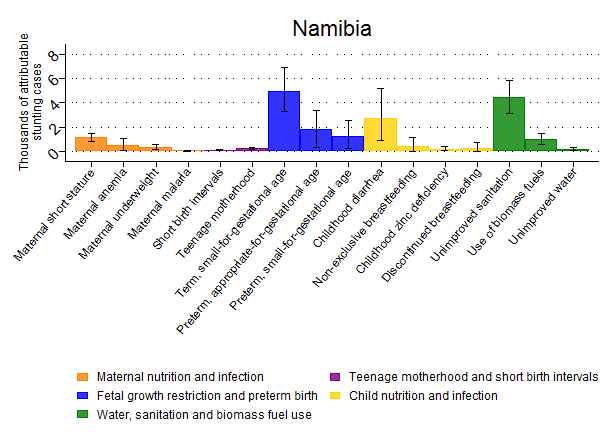


Nepal

**Region: South Asia; Sub-region: Asia, South**

**Stunting prevalence among children age 2: 54.4%**

**Stunting cases among children age 2: 338,334**

**Population of children age 2: 621,915**

This country profile provides results for 2011 from the analysis presented in *Risk factors for childhood stunting in 137 developing countries: a comparative risk assessment analysis at global, regional, and country levels.*

**Table 1: Population attributable fraction (PAF in %), attributable stunting prevalence (percentage points) and number of stunting cases among 2-year olds attributable to individual risks and risk factor clusters (95% confidence intervals presented in parentheses)**

| **Description** | **PAF** | **Attributable stunting prevalence** | **Attributable stunting cases (thousands)** |
| --- | --- | --- | --- |
| **Maternal nutrition and infection** | **18.6 (16.2, 21.4)** | **10.1 (8.3, 12.3)** | **63.1 (51.9, 76.5)** |
| Maternal short stature | 13.1 (12.9, 13.3) | 7.1 (6.0, 8.1) | 44.2 (37.6, 50.6) |
| Maternal underweight | 3.2 (1.7, 5.0) | 1.7 (0.9, 2.8) | 10.8 (5.8, 17.5) |
| Maternal malaria | 0.0 (0.0, 0.0) | 0.0 (0.0, 0.0) | 0.0 (0.0, 0.0) |
| Maternal anemia | 3.3 (0.9, 5.8) | 1.8 (0.5, 3.3) | 11.3 (3.2, 20.2) |
| **Teenage motherhood and short birth intervals** | **2.0 (1.9, 2.1)** | **1.1 (0.9, 1.2)** | **6.7 (5.7, 7.7)** |
| Teenage motherhood | 1.2 (1.1, 1.3) | 0.6 (0.5, 0.8) | 4.0 (3.4, 4.7) |
| Short birth intervals | 0.8 (0.7, 0.9) | 0.4 (0.4, 0.5) | 2.7 (2.2, 3.1) |
| **Fetal growth restriction and preterm birth** | **38.2 (34.7, 41.8)** | **20.8 (17.4, 24.2)** | **129.2 (108.3, 150.7)** |
| Preterm, small-for-gestational age | 6.4 (3.4, 9.8) | 3.5 (1.8, 5.6) | 21.5 (11.4, 35.0) |
| Preterm, appropriate-for-gestational age | 5.9 (3.6, 8.6) | 3.2 (1.9, 4.7) | 19.9 (11.6, 29.5) |
| Term, small-for-gestational age | 29.8 (25.7, 34.4) | 16.2 (13.0, 19.7) | 101 (80.9, 122.5) |
| Low birth weight | 29.0 (24.8, 33.0) | 15.8 (12.7, 19.4) | 98.0 (79.1, 120.4) |
| **Child nutrition and infection** | **10.9 (4.4, 19.1)** | **5.9 (2.4, 10.5)** | **36.7 (14.7, 65.3)** |
| Childhood zinc deficiency | 0.7 (0.3, 1.2) | 0.4 (0.2, 0.7) | 2.4 (1.0, 4.1) |
| Childhood diarrhea | 10.5 (4.1, 18.8) | 5.7 (2.2, 10.3) | 35.6 (13.6, 64.2) |
| Non-exclusive breastfeeding | 0.8 (-1.1, 2.9) | 0.5 (-0.6, 1.6) | 2.8 (-3.8, 9.8) |
| HIV infection without HAART before 2 years | No data | No data | No data |
| Discontinued breastfeeding | 0.4 (0.0, 1.1) | 0.2 (0.0, 0.6) | 1.3 (0.0, 3.8) |
| **Water, sanitation and biomass fuel use** | **24.9 (22.3, 27.6)** | **13.6 (11.1, 16.1)** | **84.3 (69.3, 99.9)** |
| Unimproved sanitation | 18.4 (16.1, 20.5) | 10.0 (8.1, 11.9) | 62.3 (50.7, 74.2) |
| Unimproved water | 1.0 (0.5, 1.7) | 0.6 (0.3, 0.9) | 3.5 (1.8, 5.7) |
| Use of biomass fuels | 7.0 (4.9, 9.3) | 3.8 (2.6, 5.2) | 23.7 (16.0, 32.4) |

**Fig 1: Stunting cases among 2-year olds attributable to individual risk factors**


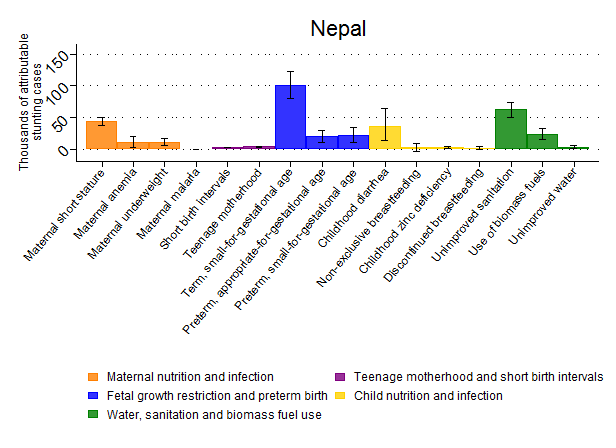


Nicaragua

**Region: Latin America/Caribbean; Sub-region: Latin America, Central**

**Stunting prevalence among children age 2: 25.4%**

**Stunting cases among children age 2: 32,211**

**Population of children age 2: 126,878**

This country profile provides results for 2011 from the analysis presented in *Risk factors for childhood stunting in 137 developing countries: a comparative risk assessment analysis at global, regional, and country levels.*

**Table 1: Population attributable fraction (PAF in %), attributable stunting prevalence (percentage points) and number of stunting cases among 2-year olds attributable to individual risks and risk factor clusters (95% confidence intervals presented in parentheses)**

| **Description** | **PAF** | **Attributable stunting prevalence** | **Attributable stunting cases (thousands)** |
| --- | --- | --- | --- |
| **Maternal nutrition and infection** | **10.9 (10.1, 11.9)** | **2.8 (2.1, 3.5)** | **3.5 (2.7, 4.4)** |
| Maternal short stature | 9.8 (9.6, 10.1) | 2.5 (2.0, 3.1) | 3.2 (2.5, 3.9) |
| Maternal underweight | 0.4 (0.1, 0.7) | 0.1 (0.0, 0.2) | 0.1 (0.0, 0.2) |
| Maternal malaria | 0.0 (0.0, 0.0) | 0.0 (0.0, 0.0) | 0.0 (0.0, 0.0) |
| Maternal anemia | 0.8 (0.1, 1.8) | 0.2 (0.0, 0.5) | 0.3 (0.0, 0.6) |
| **Teenage motherhood and short birth intervals** | **2.8 (2.7, 3.0)** | **0.7 (0.6, 0.9)** | **0.9 (0.7, 1.1)** |
| Teenage motherhood | 1.8 (1.6, 1.9) | 0.4 (0.3, 0.5) | 0.6 (0.4, 0.7) |
| Short birth intervals | 1.1 (1.0, 1.2) | 0.3 (0.2, 0.4) | 0.4 (0.3, 0.4) |
| **Fetal growth restriction and preterm birth** | **22.2 (16.4, 27.8)** | **5.6 (3.8, 7.8)** | **7.2 (4.8, 9.8)** |
| Preterm, small-for-gestational age | 5.1 (0.1, 10.7) | 1.3 (0.0, 2.8) | 1.6 (0.1, 3.5) |
| Preterm, appropriate-for-gestational age | 5.2 (0.1, 10.1) | 1.3 (0.0, 2.7) | 1.7 (0.0, 3.4) |
| Term, small-for-gestational age | 13.6 (9.8, 17.6) | 3.4 (2.2, 4.9) | 4.4 (2.8, 6.2) |
| Low birth weight | 14.8 (12.3, 17.3) | 3.8 (2.8, 4.8) | 4.8 (3.5, 6.1) |
| **Child nutrition and infection** | **18.5 (8.0, 30.2)** | **4.7 (2.0, 8.0)** | **6.0 (2.5, 10.2)** |
| Childhood zinc deficiency | 1.5 (0.6, 2.7) | 0.4 (0.1, 0.7) | 0.5 (0.2, 0.9) |
| Childhood diarrhea | 17.9 (7.6, 29.7) | 4.5 (1.9, 7.8) | 5.8 (2.4, 9.9) |
| Non-exclusive breastfeeding | 2.6 (-0.6, 6.3) | 0.7 (-0.2, 1.7) | 0.8 (-0.2, 2.2) |
| Discontinued breastfeeding | 2.3 (0.3, 5.4) | 0.6 (0.1, 1.4) | 0.7 (0.1, 1.8) |
| HIV infection without HAART before 2 years | No data | No data | No data |
| **Water, sanitation and biomass fuel use** | **16.3 (13.2, 19.6)** | **4.1 (3.0, 5.5)** | **5.3 (3.8, 7.0)** |
| Unimproved sanitation | 13.1 (9.9, 16.5) | 3.3 (2.3, 4.6) | 4.2 (2.9, 5.9) |
| Unimproved water | 1.1 (0.5, 1.8) | 0.3 (0.1, 0.5) | 0.4 (0.2, 0.6) |
| Use of biomass fuels | 2.6 (1.8, 3.6) | 0.7 (0.4, 1.0) | 0.8 (0.5, 1.2) |

**Fig 1: Stunting cases among 2-year olds attributable to individual risk factors**


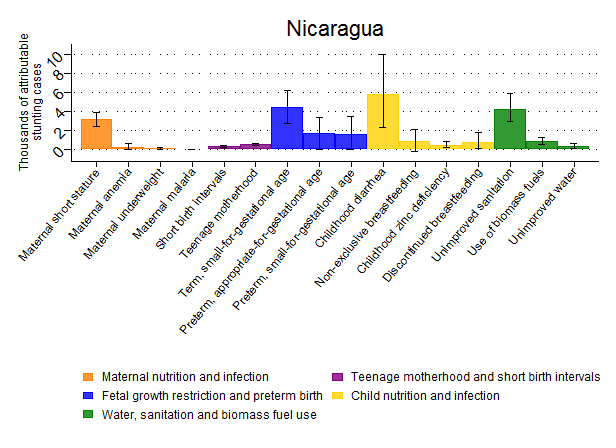


Niger

**Region: Sub-Saharan Africa; Sub-region: Sub-Saharan Africa, West**

**Stunting prevalence among children age 2: 66.0%**

**Stunting cases among children age 2: 547,064**

**Population of children age 2: 828,756**

This country profile provides results for 2011 from the analysis presented in *Risk factors for childhood stunting in 137 developing countries: a comparative risk assessment analysis at global, regional, and country levels.*

**Table 1: Population attributable fraction (PAF in %), attributable stunting prevalence (percentage points) and number of stunting cases among 2-year olds attributable to individual risks and risk factor clusters (95% confidence intervals presented in parentheses)**

| **Description** | **PAF** | **Attributable stunting prevalence** | **Attributable stunting cases (thousands)** |
| --- | --- | --- | --- |
| **Maternal nutrition and infection** | **13.6 (10.1, 17.0)** | **9.0 (6.2, 12.0)** | **74.4 (51.7, 99.5)** |
| Maternal short stature | 4.7 (4.5, 4.9) | 3.1 (2.5, 3.8) | 25.7 (20.5, 31.2) |
| Maternal underweight | 2.9 (1.5, 4.7) | 1.9 (1.0, 3.1) | 15.9 (8.1, 25.7) |
| Maternal malaria | 1.9 (0.7, 3.2) | 1.3 (0.5, 2.2) | 10.5 (3.8, 18.3) |
| Maternal anemia | 4.8 (1.4, 8.0) | 3.2 (0.9, 5.4) | 26.3 (7.7, 45.0) |
| **Teenage motherhood and short birth intervals** | **2.2 (2.1, 2.3)** | **1.5 (1.2, 1.8)** | **12.1 (9.5, 14.7)** |
| Teenage motherhood | 1.1 (1.1, 1.2) | 0.8 (0.6, 0.9) | 6.2 (4.9, 7.6) |
| Short birth intervals | 1.1 (1.0, 1.2) | 0.7 (0.6, 0.9) | 5.9 (4.6, 7.2) |
| **Fetal growth restriction and preterm birth** | **36.8 (32.0, 41.6)** | **24.3 (18.7, 30.1)** | **201.5 (154.8, 249.6)** |
| Preterm, small-for-gestational age | 3.2 (-0.3, 7.2) | 2.1 (-0.2, 4.9) | 17.7 (-1.7, 40.8) |
| Preterm, appropriate-for-gestational age | 4.5 (0.0, 9.0) | 3.0 (0.0, 6.1) | 24.6 (0.2, 50.8) |
| Term, small-for-gestational age | 31.6 (26.4, 37.4) | 20.9 (15.9, 26.9) | 172.9 (131.8, 223.1) |
| Low birth weight | 33.9 (29.4, 38.3) | 22.4 (17.2, 28.1) | 185.4 (142.7, 233) |
| **Child nutrition and infection** | **17.5 (6.1, 30.2)** | **11.6 (4.1, 20.8)** | **95.9 (34.0, 172.7)** |
| Childhood zinc deficiency | 0.4 (0.2, 0.8) | 0.3 (0.1, 0.5) | 2.5 (0.9, 4.3) |
| Childhood diarrhea | 17.3 (5.9, 30.1) | 11.4 (4.0, 20.7) | 94.9 (32.8, 171.7) |
| Non-exclusive breastfeeding | 2.1 (-0.4, 5.5) | 1.4 (-0.2, 3.7) | 11.7 (-2.0, 30.8) |
| Discontinued breastfeeding | 0.6 (0.0, 1.8) | 0.4 (0.0, 1.2) | 3.5 (0.1, 9.9) |
| HIV infection without HAART before 2 years | 0.0 (0.0, 0.0) | 0.0 (0.0, 0.0) | 0.1 (0.1, 0.2) |
| **Water, sanitation and biomass fuel use** | **34.2 (31.5, 36.8)** | **22.6 (17.6, 27.6)** | **187 (146.2, 228.4)** |
| Unimproved sanitation | 24.6 (22.6, 26.6) | 16.2 (12.6, 19.9) | 134.4 (104.1, 164.9) |
| Unimproved water | 3.9 (2.4, 5.6) | 2.5 (1.5, 3.8) | 21.1 (12.3, 31.3) |
| Use of biomass fuels | 9.3 (6.6, 11.9) | 6.1 (4.1, 8.4) | 50.6 (33.7, 69.3) |

**Fig 1: Stunting cases among 2-year olds attributable to individual risk factors**


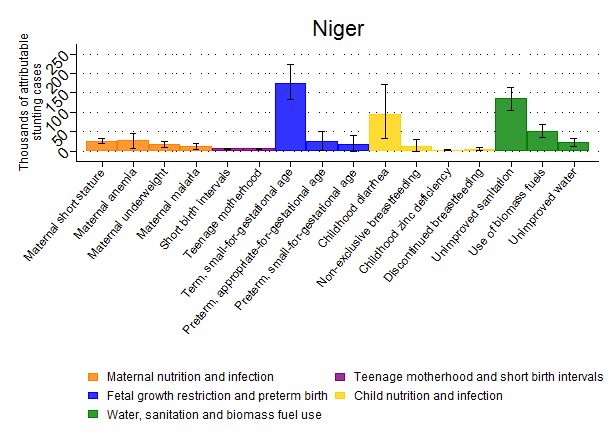


Nigeria

**Region: Sub-Saharan Africa; Sub-region: Sub-Saharan Africa, West**

**Stunting prevalence among children age 2: 42.3%**

**Stunting cases among children age 2: 2,777,701**

**Population of children age 2: 6,562,042**

This country profile provides results for 2011 from the analysis presented in *Risk factors for childhood stunting in 137 developing countries: a comparative risk assessment analysis at global, regional, and country levels.*

**Table 1: Population attributable fraction (PAF in %), attributable stunting prevalence (percentage points) and number of stunting cases among 2-year olds attributable to individual risks and risk factor clusters (95% confidence intervals presented in parentheses)**

| **Description** | **PAF** | **Attributable stunting prevalence** | **Attributable stunting cases (thousands)** |
| --- | --- | --- | --- |
| **Maternal nutrition and infection** | **12.5 (10.3, 14.8)** | **5.3 (3.9, 6.8)** | **347.3 (253, 444.9)** |
| Maternal short stature | 6.8 (6.7, 7.0) | 2.9 (2.3, 3.5) | 189.8 (151, 227.7) |
| Maternal underweight | 1.2 (0.6, 1.9) | 0.5 (0.2, 0.9) | 33.4 (15.8, 57.0) |
| Maternal malaria | 2.4 (0.9, 3.8) | 1.0 (0.4, 1.7) | 65.9 (26.2, 110.7) |
| Maternal anemia | 2.6 (0.8, 4.6) | 1.1 (0.3, 2.0) | 73.2 (21.4, 130.6) |
| **Teenage motherhood and short birth intervals** | **2.1 (2.0, 2.2)** | **0.9 (0.7, 1.1)** | **58.7 (46.8, 71.2)** |
| Teenage motherhood | 1.0 (1.0, 1.1) | 0.4 (0.3, 0.5) | 28.4 (22.7, 34.6) |
| Short birth intervals | 1.1 (1.0, 1.2) | 0.5 (0.4, 0.6) | 30.6 (24.2, 37.3) |
| **Fetal growth restriction and preterm birth** | **29.8 (26.5, 33.1)** | **12.6 (9.6, 15.5)** | **826.6 (631.5, 1014.3)** |
| Preterm, small-for-gestational age | 4.9 (2.8, 7.6) | 2.1 (1.1, 3.3) | 136.1 (74.8, 219.4) |
| Preterm, appropriate-for-gestational age | 6.5 (4.1, 9.1) | 2.7 (1.7, 4.0) | 179.9 (108.7, 264) |
| Term, small-for-gestational age | 21.0 (17.4, 24.9) | 8.9 (6.7, 11.3) | 583.6 (437.4, 741.6) |
| Low birth weight | 18.4 (15.4, 21.4) | 7.8 (5.9, 10.0) | 510.5 (386.9, 655.7) |
| **Child nutrition and infection** | **17.5 (6.6, 31.1)** | **7.4 (2.7, 13.6)** | **486.4 (178.4, 892.6)** |
| Childhood zinc deficiency | 0.8 (0.3, 1.6) | 0.4 (0.1, 0.7) | 23.3 (8.6, 45.4) |
| Childhood diarrhea | 17.2 (6.3, 30.8) | 7.3 (2.5, 13.5) | 476.8 (165.9, 885.7) |
| Non-exclusive breastfeeding | 2.3 (-0.3, 6.0) | 1.0 (-0.2, 2.5) | 65.1 (-10.4, 162.6) |
| Discontinued breastfeeding | 1.0 (0.1, 2.5) | 0.4 (0.0, 1.1) | 27.3 (2.6, 71.7) |
| HIV infection without HAART before 2 years | 0.4 (0.2, 0.9) | 0.2 (0.1, 0.4) | 10.7 (4.4, 24.7) |
| **Water, sanitation and biomass fuel use** | **25.7 (23.4, 28.0)** | **10.9 (8.5, 13.4)** | **713.1 (554.7, 876.2)** |
| Unimproved sanitation | 19.9 (17.9, 21.9) | 8.4 (6.5, 10.5) | 552.4 (423.4, 686.2) |
| Unimproved water | 3.1 (2.0, 4.4) | 1.3 (0.8, 1.9) | 86.7 (53.9, 124.2) |
| Use of biomass fuels | 4.2 (2.9, 5.6) | 1.8 (1.2, 2.5) | 117.5 (76.4, 166.3) |

**Fig 1: Stunting cases among 2-year olds attributable to individual risk factors**


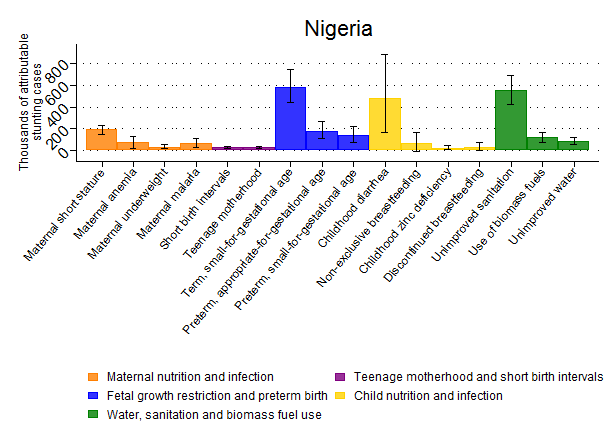


Oman

**Region: North Africa/Middle East; Sub-region: North Africa / Middle East**

**Stunting prevalence among children age 2: 12.8%**

**Stunting cases among children age 2: 8,710**

**Population of children age 2: 67,828**

This country profile provides results for 2011 from the analysis presented in *Risk factors for childhood stunting in 137 developing countries: a comparative risk assessment analysis at global, regional, and country levels.*

**Table 1: Population attributable fraction (PAF in %), attributable stunting prevalence (percentage points) and number of stunting cases among 2-year olds attributable to individual risks and risk factor clusters (95% confidence intervals presented in parentheses)**

| **Description** | **PAF** | **Attributable stunting prevalence** | **Attributable stunting cases (thousands)** |
| --- | --- | --- | --- |
| **Maternal nutrition and infection** | **11.7 (10.4, 13.1)** | **1.5 (1.1, 2.0)** | **1.0 (0.7, 1.3)** |
| Maternal short stature | 9.8 (9.1, 10.4) | 1.3 (0.9, 1.6) | 0.9 (0.6, 1.1) |
| Maternal underweight | 0.6 (0.2, 1.1) | 0.1 (0.0, 0.1) | 0.1 (0.0, 0.1) |
| Maternal malaria | 0.0 (0.0, 0.0) | 0.0 (0.0, 0.0) | 0.0 (0.0, 0.0) |
| Maternal anemia | 1.5 (0.3, 2.8) | 0.2 (0.0, 0.4) | 0.1 (0.0, 0.3) |
| **Teenage motherhood and short birth intervals** | **1.8 (1.7, 1.9)** | **0.2 (0.2, 0.3)** | **0.2 (0.1, 0.2)** |
| Teenage motherhood | 0.5 (0.5, 0.6) | 0.1 (0.0, 0.1) | 0.0 (0.0, 0.1) |
| Short birth intervals | 1.2 (1.1, 1.3) | 0.2 (0.1, 0.2) | 0.1 (0.1, 0.1) |
| **Fetal growth restriction and preterm birth** | **28.4 (23.4, 33.0)** | **3.6 (2.5, 4.8)** | **2.5 (1.7, 3.3)** |
| Preterm, small-for-gestational age | 7.8 (3.8, 12.4) | 1.0 (0.5, 1.7) | 0.7 (0.3, 1.1) |
| Preterm, appropriate-for-gestational age | 7.2 (3.6, 11.1) | 0.9 (0.4, 1.6) | 0.6 (0.3, 1.1) |
| Term, small-for-gestational age | 16.4 (11.4, 21.4) | 2.1 (1.3, 3.0) | 1.4 (0.9, 2.0) |
| Low birth weight | 16.0 (13.4, 18.7) | 2.1 (1.4, 2.8) | 1.4 (1.0, 1.9) |
| **Child nutrition and infection** | **8.3 (3.5, 14.1)** | **1.1 (0.4, 2.0)** | **0.7 (0.3, 1.3)** |
| Childhood zinc deficiency | 2.3 (0.7, 6.2) | 0.3 (0.1, 0.8) | 0.2 (0.1, 0.5) |
| Childhood diarrhea | 7.2 (2.9, 12.2) | 0.9 (0.3, 1.7) | 0.6 (0.2, 1.1) |
| Non-exclusive breastfeeding | 0.7 (-0.7, 2.1) | 0.1 (-0.1, 0.3) | 0.1 (-0.1, 0.2) |
| HIV infection without HAART before 2 years | No data | No data | No data |
| Discontinued breastfeeding | 0.5 (0.0, 1.4) | 0.1 (0.0, 0.2) | 0.0 (0.0, 0.1) |
| **Water, sanitation and biomass fuel use** | **2.1 (0.7, 3.5)** | **0.3 (0.1, 0.5)** | **0.2 (0.1, 0.3)** |
| Unimproved sanitation | 1.4 (0.1, 2.8) | 0.2 (0.0, 0.4) | 0.1 (0.0, 0.3) |
| Unimproved water | 0.7 (0.3, 1.2) | 0.1 (0.0, 0.2) | 0.1 (0.0, 0.1) |
| Use of biomass fuels | 0.0 (0.0, 0.0) | 0.0 (0.0, 0.0) | 0.0 (0.0, 0.0) |

**Fig 1: Stunting cases among 2-year olds attributable to individual risk factors**


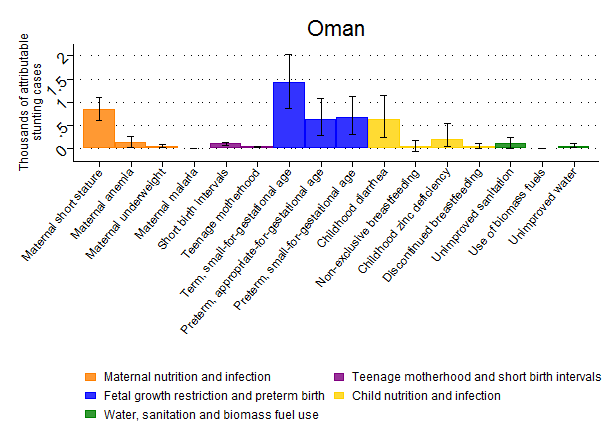


Pakistan

**Region: South Asia; Sub-region: Asia, South**

**Stunting prevalence among children age 2: 48.2%**

**Stunting cases among children age 2: 2,469,091**

**Population of children age 2: 5,118,224**

This country profile provides results for 2011 from the analysis presented in *Risk factors for childhood stunting in 137 developing countries: a comparative risk assessment analysis at global, regional, and country levels.*

**Table 1: Population attributable fraction (PAF in %), attributable stunting prevalence (percentage points) and number of stunting cases among 2-year olds attributable to individual risks and risk factor clusters (95% confidence intervals presented in parentheses)**

| **Description** | **PAF** | **Attributable stunting prevalence** | **Attributable stunting cases (thousands)** |
| --- | --- | --- | --- |
| **Maternal nutrition and infection** | **16.2 (13.4, 19.0)** | **7.8 (5.8, 10.0)** | **399.4 (296.9, 514.4)** |
| Maternal short stature | 9.5 (9.3, 9.7) | 4.6 (3.7, 5.4) | 234.5 (191.6, 277.9) |
| Maternal underweight | 3.3 (1.8, 5.3) | 1.6 (0.9, 2.6) | 82.7 (44.2, 134.4) |
| Maternal malaria | 0.0 (0.0, 0.0) | 0.0 (0.0, 0.0) | 0.0 (0.0, 0.0) |
| Maternal anemia | 4.2 (1.2, 7.0) | 2.0 (0.6, 3.6) | 103 (29.5, 182.6) |
| **Teenage motherhood and short birth intervals** | **2.2 (2.1, 2.3)** | **1.1 (0.9, 1.3)** | **54.1 (43.6, 64.6)** |
| Teenage motherhood | 0.6 (0.6, 0.7) | 0.3 (0.2, 0.4) | 15.8 (12.6, 19.2) |
| Short birth intervals | 1.6 (1.4, 1.7) | 0.8 (0.6, 0.9) | 38.5 (30.7, 46.3) |
| **Fetal growth restriction and preterm birth** | **41.6 (38.0, 45.2)** | **20.1 (15.9, 24.1)** | **1027.6 (814, 1234.1)** |
| Preterm, small-for-gestational age | 6.6 (3.6, 10.5) | 3.2 (1.7, 5.1) | 163.8 (85.4, 261.5) |
| Preterm, appropriate-for-gestational age | 6.2 (3.7, 9.1) | 3.0 (1.7, 4.6) | 154.1 (88.2, 237.2) |
| Term, small-for-gestational age | 33.3 (28.8, 38.3) | 16.1 (12.4, 19.9) | 821.7 (633.1, 1020.8) |
| Low birth weight | 33.3 (28.8, 37.6) | 16.1 (12.5, 19.7) | 822.5 (640, 1005.8) |
| **Child nutrition and infection** | **14.7 (5.5, 26.0)** | **7.1 (2.7, 13.3)** | **363.7 (136, 681.6)** |
| Childhood zinc deficiency | 0.7 (0.3, 1.2) | 0.3 (0.1, 0.6) | 16.8 (6.5, 30.2) |
| Childhood diarrhea | 14.4 (5.2, 25.7) | 7.0 (2.5, 13.2) | 356.5 (129.7, 673.4) |
| Non-exclusive breastfeeding | 2.2 (0.0, 5.3) | 1.1 (0.0, 2.6) | 55.5 (0.2, 133.4) |
| HIV infection without HAART before 2 years | No data | No data | No data |
| Discontinued breastfeeding | 1.3 (0.1, 3.3) | 0.6 (0.1, 1.7) | 31.5 (3.5, 87.1) |
| **Water, sanitation and biomass fuel use** | **22.2 (19.5, 24.9)** | **10.7 (8.3, 13.1)** | **547.6 (424, 671.3)** |
| Unimproved sanitation | 16.1 (13.9, 18.5) | 7.8 (6.0, 9.7) | 397.7 (306.3, 494.8) |
| Unimproved water | 0.7 (0.3, 1.1) | 0.3 (0.2, 0.6) | 17.1 (8.3, 28.7) |
| Use of biomass fuels | 6.6 (4.6, 8.8) | 3.2 (2.1, 4.5) | 162.7 (106.6, 227.9) |

**Fig 1: Stunting cases among 2-year olds attributable to individual risk factors**


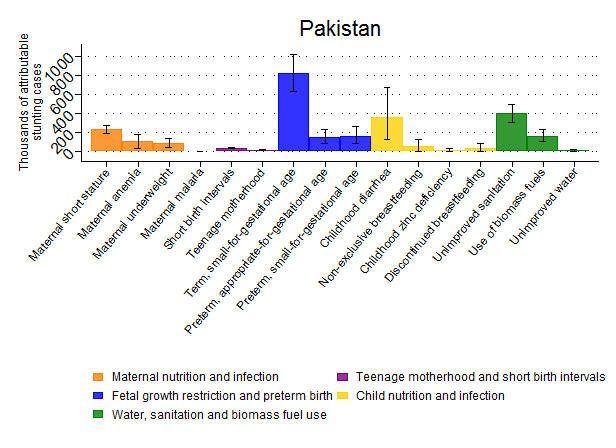


Panama

**Region: Latin America/Caribbean; Sub-region: Latin America, Central**

**Stunting prevalence among children age 2: 13.7%**

**Stunting cases among children age 2: 10,106**

**Population of children age 2: 73,797**

This country profile provides results for 2011 from the analysis presented in *Risk factors for childhood stunting in 137 developing countries: a comparative risk assessment analysis at global, regional, and country levels.*

**Table 1: Population attributable fraction (PAF in %), attributable stunting prevalence (percentage points) and number of stunting cases among 2-year olds attributable to individual risks and risk factor clusters (95% confidence intervals presented in parentheses)**

| **Description** | **PAF** | **Attributable stunting prevalence** | **Attributable stunting cases (thousands)** |
| --- | --- | --- | --- |
| **Maternal nutrition and infection** | **10.3 (9.4, 11.4)** | **1.4 (0.6, 2.2)** | **1.0 (0.5, 1.6)** |
| Maternal short stature | 9.1 (8.7, 9.5) | 1.2 (0.5, 1.9) | 0.9 (0.4, 1.4) |
| Maternal underweight | 0.3 (0.1, 0.6) | 0.0 (0.0, 0.1) | 0.0 (0.0, 0.1) |
| Maternal malaria | 0.0 (0.0, 0.0) | 0.0 (0.0, 0.0) | 0.0 (0.0, 0.0) |
| Maternal anemia | 1.1 (0.3, 2.1) | 0.1 (0.0, 0.3) | 0.1 (0.0, 0.2) |
| **Teenage motherhood and short birth intervals** | **2.5 (2.4, 2.6)** | **0.3 (0.1, 0.5)** | **0.2 (0.1, 0.4)** |
| Teenage motherhood | 1.5 (1.4, 1.6) | 0.2 (0.1, 0.3) | 0.2 (0.1, 0.2) |
| Short birth intervals | 1.0 (0.9, 1.1) | 0.1 (0.1, 0.2) | 0.1 (0.0, 0.2) |
| **Fetal growth restriction and preterm birth** | **20.7 (16.3, 24.8)** | **2.8 (1.2, 4.5)** | **2.1 (0.9, 3.3)** |
| Preterm, small-for-gestational age | 4.6 (2.0, 7.9) | 0.6 (0.2, 1.2) | 0.5 (0.1, 0.9) |
| Preterm, appropriate-for-gestational age | 4.6 (2.3, 7.1) | 0.6 (0.2, 1.2) | 0.5 (0.1, 0.9) |
| Term, small-for-gestational age | 12.7 (8.8, 16.9) | 1.7 (0.7, 2.9) | 1.3 (0.5, 2.1) |
| Low birth weight | 14.1 (11.7, 16.5) | 1.9 (0.9, 3.1) | 1.4 (0.6, 2.3) |
| **Child nutrition and infection** | **17.9 (7.7, 28.6)** | **2.5 (0.8, 4.8)** | **1.8 (0.6, 3.5)** |
| Childhood zinc deficiency | 1.7 (0.5, 5.5) | 0.2 (0.0, 0.8) | 0.2 (0.0, 0.6) |
| Childhood diarrhea | 17.2 (7.2, 27.5) | 2.4 (0.7, 4.6) | 1.7 (0.5, 3.4) |
| Non-exclusive breastfeeding | 2.7 (-0.4, 6.4) | 0.4 (-0.1, 1.0) | 0.3 (0.0, 0.8) |
| HIV infection without HAART before 2 years | No data | No data | No data |
| Discontinued breastfeeding | 2.3 (0.4, 5.6) | 0.3 (0.0, 0.9) | 0.2 (0.0, 0.6) |
| **Water, sanitation and biomass fuel use** | **9.5 (7.0, 11.9)** | **1.3 (0.6, 2.2)** | **1.0 (0.4, 1.6)** |
| Unimproved sanitation | 8.1 (5.7, 10.5) | 1.1 (0.4, 1.9) | 0.8 (0.3, 1.4) |
| Unimproved water | 0.5 (0.2, 0.9) | 0.1 (0.0, 0.1) | 0.1 (0.0, 0.1) |
| Use of biomass fuels | 0.9 (0.4, 1.5) | 0.1 (0.0, 0.3) | 0.1 (0.0, 0.2) |

**Fig 1: Stunting cases among 2-year olds attributable to individual risk factors**


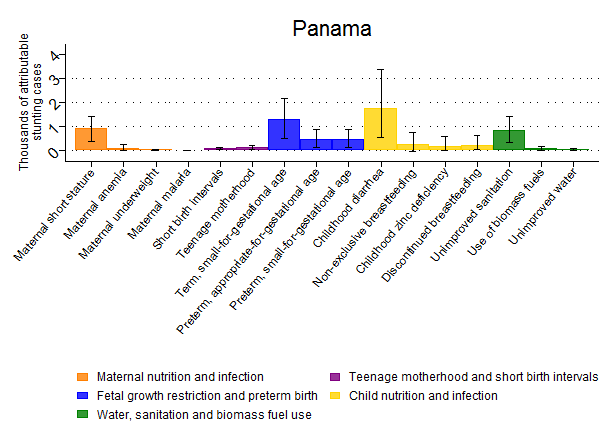


Papua New Guinea

**Region: East Asia/Pacific; Sub-region: Oceania**

**Stunting prevalence among children age 2: 39.5%**

**Stunting cases among children age 2: 81,672**

**Population of children age 2: 206,879**

This country profile provides results for 2011 from the analysis presented in *Risk factors for childhood stunting in 137 developing countries: a comparative risk assessment analysis at global, regional, and country levels.*

**Table 1: Population attributable fraction (PAF in %), attributable stunting prevalence (percentage points) and number of stunting cases among 2-year olds attributable to individual risks and risk factor clusters (95% confidence intervals presented in parentheses)**

| **Description** | **PAF** | **Attributable stunting prevalence** | **Attributable stunting cases (thousands)** |
| --- | --- | --- | --- |
| **Maternal nutrition and infection** | **10.5 (9.3, 12.0)** | **4.1 (2.6, 5.8)** | **8.5 (5.3, 12.0)** |
| Maternal short stature | 8.6 (8.3, 8.9) | 3.4 (2.2, 4.6) | 7.0 (4.6, 9.6) |
| Maternal underweight | 0.5 (0.1, 1.0) | 0.2 (0.0, 0.4) | 0.4 (0.1, 0.8) |
| Maternal malaria | 0.0 (0.0, 0.0) | 0.0 (0.0, 0.0) | 0.0 (0.0, 0.0) |
| Maternal anemia | 1.6 (0.4, 3.3) | 0.6 (0.1, 1.4) | 1.3 (0.3, 2.8) |
| **Teenage motherhood and short birth intervals** | **1.3 (1.2, 1.4)** | **0.5 (0.3, 0.7)** | **1.1 (0.7, 1.4)** |
| Teenage motherhood | 0.5 (0.4, 0.5) | 0.2 (0.1, 0.2) | 0.4 (0.2, 0.5) |
| Short birth intervals | 0.8 (0.8, 0.9) | 0.3 (0.2, 0.5) | 0.7 (0.4, 1.0) |
| **Fetal growth restriction and preterm birth** | **26.3 (20.4, 31.4)** | **10.4 (6.4, 14.8)** | **21.5 (13.2, 30.6)** |
| Preterm, small-for-gestational age | 3.7 (-0.2, 7.9) | 1.5 (-0.1, 3.4) | 3.0 (-0.1, 7.1) |
| Preterm, appropriate-for-gestational age | 3.4 (0.2, 7.0) | 1.4 (0.1, 3.0) | 2.8 (0.1, 6.2) |
| Term, small-for-gestational age | 20.8 (15.1, 26.0) | 8.2 (4.9, 12.2) | 17.0 (10.2, 25.2) |
| Low birth weight | 16.1 (13.5, 18.8) | 6.4 (3.9, 8.9) | 13.2 (8.0, 18.3) |
| **Child nutrition and infection** | **13.8 (5.5, 23.1)** | **5.5 (2.0, 10.0)** | **11.3 (4.2, 20.8)** |
| Childhood zinc deficiency | 0.4 (0.1, 0.7) | 0.1 (0.0, 0.3) | 0.3 (0.1, 0.6) |
| Childhood diarrhea | 13.7 (5.3, 23.0) | 5.4 (2.0, 10.0) | 11.2 (4.1, 20.6) |
| Non-exclusive breastfeeding | 0.9 (-2.0, 3.6) | 0.4 (-0.8, 1.5) | 0.8 (-1.7, 3.1) |
| HIV infection without HAART before 2 years | No data | No data | No data |
| Discontinued breastfeeding | 0.8 (0.0, 2.2) | 0.3 (0.0, 0.9) | 0.6 (0.0, 1.9) |
| **Water, sanitation and biomass fuel use** | **27.7 (23.8, 31.5)** | **10.9 (7.1, 15.3)** | **22.6 (14.6, 31.6)** |
| Unimproved sanitation | 22.0 (18.0, 25.6) | 8.7 (5.6, 12.3) | 17.9 (11.5, 25.5) |
| Unimproved water | 4.0 (2.0, 6.4) | 1.6 (0.7, 2.8) | 3.3 (1.5, 5.9) |
| Use of biomass fuels | 3.5 (2.5, 4.7) | 1.4 (0.8, 2.1) | 2.9 (1.7, 4.4) |

**Fig 1: Stunting cases among 2-year olds attributable to individual risk factors**


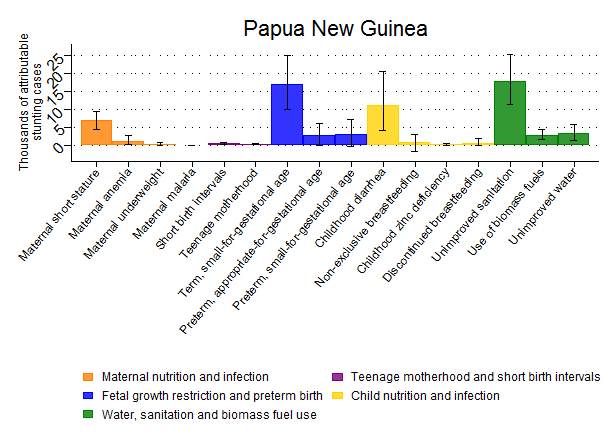


Paraguay

**Region: Latin America/Caribbean; Sub-region: Latin America, Tropical**

**Stunting prevalence among children age 2: 15.2%**

**Stunting cases among children age 2: 21,237**

**Population of children age 2: 139,554**

This country profile provides results for 2011 from the analysis presented in *Risk factors for childhood stunting in 137 developing countries: a comparative risk assessment analysis at global, regional, and country levels.*

**Table 1: Population attributable fraction (PAF in %), attributable stunting prevalence (percentage points) and number of stunting cases among 2-year olds attributable to individual risks and risk factor clusters (95% confidence intervals presented in parentheses)**

| **Description** | **PAF** | **Attributable stunting prevalence** | **Attributable stunting cases (thousands)** |
| --- | --- | --- | --- |
| **Maternal nutrition and infection** | **10.3 (9.1, 11.6)** | **1.6 (0.8, 2.3)** | **2.2 (1.1, 3.2)** |
| Maternal short stature | 9.3 (8.3, 10.0) | 1.4 (0.8, 2.1) | 2.0 (1.1, 2.9) |
| Maternal underweight | 0.3 (0.0, 0.5) | 0.0 (0.0, 0.1) | 0.1 (0.0, 0.1) |
| Maternal malaria | 0.0 (0.0, 0.0) | 0.0 (0.0, 0.0) | 0.0 (0.0, 0.0) |
| Maternal anemia | 0.9 (0.1, 1.9) | 0.1 (0.0, 0.3) | 0.2 (0.0, 0.5) |
| **Teenage motherhood and short birth intervals** | **2.5 (2.3, 2.6)** | **0.4 (0.2, 0.5)** | **0.5 (0.3, 0.8)** |
| Teenage motherhood | 1.3 (1.2, 1.5) | 0.2 (0.1, 0.3) | 0.3 (0.2, 0.4) |
| Short birth intervals | 1.1 (1.0, 1.2) | 0.2 (0.1, 0.3) | 0.2 (0.1, 0.4) |
| **Fetal growth restriction and preterm birth** | **18.4 (12.2, 23.9)** | **2.8 (1.3, 4.5)** | **3.9 (1.8, 6.3)** |
| Preterm, small-for-gestational age | 4.5 (-0.4, 9.6) | 0.7 (-0.1, 1.6) | 1.0 (-0.1, 2.2) |
| Preterm, appropriate-for-gestational age | 4.4 (-0.1, 9.3) | 0.7 (0.0, 1.6) | 0.9 (0.0, 2.2) |
| Term, small-for-gestational age | 10.5 (6.9, 14.3) | 1.6 (0.8, 2.6) | 2.2 (1.0, 3.6) |
| Low birth weight | 10.8 (8.9, 12.8) | 1.6 (0.8, 2.5) | 2.3 (1.2, 3.5) |
| **Child nutrition and infection** | **17.8 (7.6, 28.2)** | **2.7 (0.9, 5.0)** | **3.8 (1.3, 7.0)** |
| Childhood zinc deficiency | 0.7 (0.2, 1.7) | 0.1 (0.0, 0.3) | 0.1 (0.0, 0.4) |
| Childhood diarrhea | 17.5 (7.5, 27.9) | 2.7 (0.9, 5.0) | 3.7 (1.2, 6.9) |
| Non-exclusive breastfeeding | 2.8 (0.0, 6.2) | 0.4 (0.0, 1.0) | 0.6 (0.0, 1.4) |
| HIV infection without HAART before 2 years | No data | No data | No data |
| Discontinued breastfeeding | 2.5 (0.4, 5.6) | 0.4 (0.1, 1.0) | 0.5 (0.1, 1.3) |
| **Water, sanitation and biomass fuel use** | **9.9 (7.3, 12.2)** | **1.5 (0.7, 2.3)** | **2.1 (1.0, 3.3)** |
| Unimproved sanitation | 7.4 (5.0, 9.9) | 1.1 (0.5, 1.8) | 1.6 (0.7, 2.5) |
| Unimproved water | 1.0 (0.5, 1.6) | 0.2 (0.1, 0.3) | 0.2 (0.1, 0.4) |
| Use of biomass fuels | 1.7 (1.1, 2.3) | 0.3 (0.1, 0.4) | 0.4 (0.2, 0.6) |

**Fig 1: Stunting cases among 2-year olds attributable to individual risk factors**


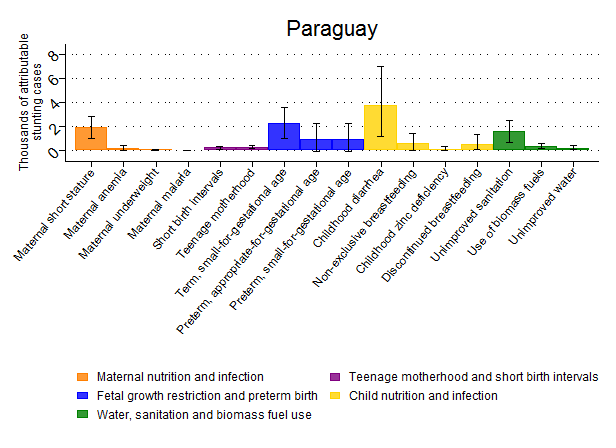


Peru

**Region: Latin America/Caribbean; Sub-region: Latin America, Andean**

**Stunting prevalence among children age 2: 24.2%**

**Stunting cases among children age 2: 148,809**

**Population of children age 2: 614,060**

This country profile provides results for 2011 from the analysis presented in *Risk factors for childhood stunting in 137 developing countries: a comparative risk assessment analysis at global, regional, and country levels.*

**Table 1: Population attributable fraction (PAF in %), attributable stunting prevalence (percentage points) and number of stunting cases among 2-year olds attributable to individual risks and risk factor clusters (95% confidence intervals presented in parentheses)**

| **Description** | **PAF** | **Attributable stunting prevalence** | **Attributable stunting cases (thousands)** |
| --- | --- | --- | --- |
| **Maternal nutrition and infection** | **12.8 (12.3, 13.4)** | **3.1 (2.7, 3.6)** | **19.1 (16.6, 21.9)** |
| Maternal short stature | 12.0 (11.8, 12.2) | 2.9 (2.5, 3.3) | 17.9 (15.5, 20.3) |
| Maternal underweight | 0.1 (0.0, 0.2) | 0.0 (0.0, 0.1) | 0.2 (0.1, 0.4) |
| Maternal malaria | 0.0 (0.0, 0.0) | 0.0 (0.0, 0.0) | 0.0 (0.0, 0.0) |
| Maternal anemia | 0.8 (0.2, 1.4) | 0.2 (0.1, 0.3) | 1.2 (0.3, 2.1) |
| **Teenage motherhood and short birth intervals** | **1.9 (1.8, 2.0)** | **0.5 (0.4, 0.5)** | **2.8 (2.4, 3.2)** |
| Teenage motherhood | 1.0 (0.9, 1.1) | 0.2 (0.2, 0.3) | 1.5 (1.3, 1.7) |
| Short birth intervals | 0.9 (0.8, 0.9) | 0.2 (0.2, 0.2) | 1.3 (1.1, 1.5) |
| **Fetal growth restriction and preterm birth** | **18.6 (14.8, 22.6)** | **4.5 (3.4, 5.6)** | **27.7 (20.9, 34.4)** |
| Preterm, small-for-gestational age | 4.3 (2.0, 7.2) | 1.0 (0.5, 1.8) | 6.4 (3.1, 10.8) |
| Preterm, appropriate-for-gestational age | 4.3 (2.0, 6.8) | 1.1 (0.5, 1.7) | 6.5 (3.0, 10.3) |
| Term, small-for-gestational age | 11.1 (7.5, 14.8) | 2.7 (1.7, 3.6) | 16.5 (10.5, 22.4) |
| Low birth weight | 12.0 (10.0, 14.1) | 2.9 (2.3, 3.6) | 17.9 (14.4, 21.9) |
| **Child nutrition and infection** | **18.0 (7.6, 28.7)** | **4.4 (1.8, 7.1)** | **26.7 (11.1, 43.4)** |
| Childhood zinc deficiency | 0.9 (0.3, 1.6) | 0.2 (0.1, 0.4) | 1.3 (0.5, 2.5) |
| Childhood diarrhea | 17.6 (7.2, 28.3) | 4.3 (1.7, 7.0) | 26.2 (10.5, 42.9) |
| Non-exclusive breastfeeding | 1.4 (-2.2, 4.6) | 0.3 (-0.5, 1.2) | 2.1 (-3.2, 7.1) |
| HIV infection without HAART before 2 years | No data | No data | No data |
| Discontinued breastfeeding | 1.4 (0.2, 3.4) | 0.3 (0.0, 0.8) | 2.0 (0.3, 5.0) |
| **Water, sanitation and biomass fuel use** | **11.6 (9.5, 13.6)** | **2.8 (2.2, 3.4)** | **17.2 (13.6, 21.1)** |
| Unimproved sanitation | 9.1 (7.2, 11.0) | 2.2 (1.7, 2.8) | 13.6 (10.3, 17.1) |
| Unimproved water | 1.2 (0.6, 2.0) | 0.3 (0.1, 0.5) | 1.8 (0.9, 2.9) |
| Use of biomass fuels | 1.5 (1.0, 2.1) | 0.4 (0.2, 0.5) | 2.3 (1.5, 3.2) |

**Fig 1: Stunting cases among 2-year olds attributable to individual risk factors**


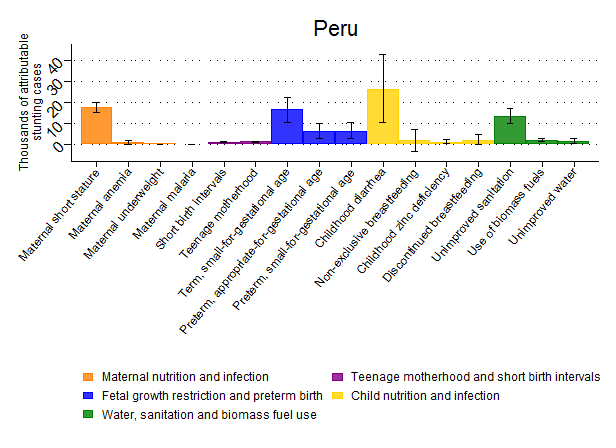


Philippines

**Region: East Asia/Pacific; Sub-region: Asia, Southeast**

**Stunting prevalence among children age 2: 35.6%**

**Stunting cases among children age 2: 820,400**

**Population of children age 2: 2,307,415**

This country profile provides results for 2011 from the analysis presented in *Risk factors for childhood stunting in 137 developing countries: a comparative risk assessment analysis at global, regional, and country levels.*

**Table 1: Population attributable fraction (PAF in %), attributable stunting prevalence (percentage points) and number of stunting cases among 2-year olds attributable to individual risks and risk factor clusters (95% confidence intervals presented in parentheses)**

| **Description** | **PAF** | **Attributable stunting prevalence** | **Attributable stunting cases (thousands)** |
| --- | --- | --- | --- |
| **Maternal nutrition and infection** | **18.9 (17.0, 21.2)** | **6.7 (5.4, 8.0)** | **155 (125.3, 185.2)** |
| Maternal short stature | 14.8 (14.5, 15.1) | 5.3 (4.4, 6.1) | 121.4 (101.3, 141.2) |
| Maternal underweight | 2.4 (1.3, 3.8) | 0.9 (0.5, 1.4) | 19.9 (10.4, 32.8) |
| Maternal malaria | 0.0 (0.0, 0.0) | 0.0 (0.0, 0.0) | 0.0 (0.0, 0.0) |
| Maternal anemia | 2.4 (0.6, 4.7) | 0.9 (0.2, 1.7) | 20.0 (5.5, 39.3) |
| **Teenage motherhood and short birth intervals** | **1.7 (1.6, 1.9)** | **0.6 (0.5, 0.7)** | **14.3 (11.7, 16.8)** |
| Teenage motherhood | 0.6 (0.5, 0.6) | 0.2 (0.2, 0.2) | 4.6 (3.7, 5.5) |
| Short birth intervals | 1.2 (1.1, 1.3) | 0.4 (0.3, 0.5) | 9.7 (8.0, 11.5) |
| **Fetal growth restriction and preterm birth** | **35.7 (30.8, 40.1)** | **12.7 (10.2, 15.3)** | **292.9 (236.3, 353.8)** |
| Preterm, small-for-gestational age | 7.0 (1.3, 13.5) | 2.5 (0.5, 4.8) | 57.4 (11.0, 111.5) |
| Preterm, appropriate-for-gestational age | 6.5 (1.4, 11.7) | 2.3 (0.5, 4.3) | 53.6 (11.5, 99.3) |
| Term, small-for-gestational age | 26.0 (20.9, 31.5) | 9.2 (7.0, 11.8) | 213.1 (161.1, 273.2) |
| Low birth weight | 27.4 (23.4, 31.3) | 9.7 (7.7, 11.9) | 224.5 (178.5, 275.2) |
| **Child nutrition and infection** | **11.1 (4.4, 17.9)** | **4.0 (1.5, 6.7)** | **91.2 (34.3, 155.5)** |
| Childhood zinc deficiency | 0.4 (0.2, 0.7) | 0.1 (0.1, 0.2) | 3.3 (1.3, 5.8) |
| Childhood diarrhea | 10.9 (4.2, 17.8) | 3.9 (1.4, 6.7) | 89.8 (33.0, 154.7) |
| Non-exclusive breastfeeding | 1.7 (-0.1, 3.8) | 0.6 (0.0, 1.4) | 13.6 (-1.0, 31.5) |
| HIV infection without HAART before 2 years | No data | No data | No data |
| Discontinued breastfeeding | 1.4 (0.2, 3.3) | 0.5 (0.1, 1.2) | 11.3 (1.5, 27.6) |
| **Water, sanitation and biomass fuel use** | **12.5 (10.0, 15.3)** | **4.4 (3.3, 5.7)** | **102.6 (76.8, 131.3)** |
| Unimproved sanitation | 7.8 (5.9, 9.8) | 2.8 (1.9, 3.7) | 64.4 (44.8, 84.8) |
| Unimproved water | 0.6 (0.3, 1.0) | 0.2 (0.1, 0.4) | 5.0 (2.6, 8.2) |
| Use of biomass fuels | 4.5 (2.5, 6.8) | 1.6 (0.9, 2.4) | 36.7 (20.0, 55.6) |

**Fig 1: Stunting cases among 2-year olds attributable to individual risk factors**


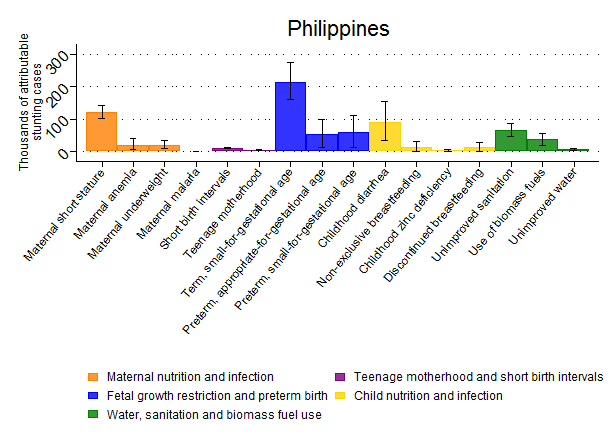


Qatar

**Region: North Africa/Middle East; Sub-region: North Africa / Middle East**

**Stunting prevalence among children age 2: 8.9%**

**Stunting cases among children age 2: 1,834**

**Population of children age 2: 20,589**

This country profile provides results for 2011 from the analysis presented in *Risk factors for childhood stunting in 137 developing countries: a comparative risk assessment analysis at global, regional, and country levels.*

**Table 1: Population attributable fraction (PAF in %), attributable stunting prevalence (percentage points) and number of stunting cases among 2-year olds attributable to individual risks and risk factor clusters (95% confidence intervals presented in parentheses)**

| **Description** | **PAF** | **Attributable stunting prevalence** | **Attributable stunting cases (thousands)** |
| --- | --- | --- | --- |
| **Maternal nutrition and infection** | **6.4 (5.6, 7.4)** | **0.6 (0.1, 1.1)** | **0.1 (0.0, 0.2)** |
| Maternal short stature | 5.4 (5.1, 5.6) | 0.5 (0.1, 0.9) | 0.1 (0.0, 0.2) |
| Maternal underweight | 0.2 (0.0, 0.4) | 0.0 (0.0, 0.0) | 0.0 (0.0, 0.0) |
| Maternal malaria | 0.0 (0.0, 0.0) | 0.0 (0.0, 0.0) | 0.0 (0.0, 0.0) |
| Maternal anemia | 0.9 (0.2, 1.9) | 0.1 (0.0, 0.2) | 0.0 (0.0, 0.0) |
| **Teenage motherhood and short birth intervals** | **1.8 (1.7, 1.9)** | **0.2 (0.0, 0.3)** | **0.0 (0.0, 0.1)** |
| Teenage motherhood | 0.5 (0.5, 0.6) | 0.0 (0.0, 0.1) | 0.0 (0.0, 0.0) |
| Short birth intervals | 1.2 (1.1, 1.3) | 0.1 (0.0, 0.2) | 0.0 (0.0, 0.0) |
| **Fetal growth restriction and preterm birth** | **23.4 (17.1, 29.1)** | **2.1 (0.3, 3.9)** | **0.4 (0.1, 0.8)** |
| Preterm, small-for-gestational age | 6.3 (0.9, 11.5) | 0.6 (0.0, 1.4) | 0.1 (0.0, 0.3) |
| Preterm, appropriate-for-gestational age | 5.7 (1.0, 10.8) | 0.5 (0.0, 1.3) | 0.1 (0.0, 0.3) |
| Term, small-for-gestational age | 13.2 (8.2, 18.5) | 1.2 (0.2, 2.4) | 0.2 (0.0, 0.5) |
| Low birth weight | 12.0 (10.0, 14.1) | 1.1 (0.2, 2.0) | 0.2 (0.0, 0.4) |
| **Child nutrition and infection** | **11.3 (3.7, 32.9)** | **1.0 (0.1, 3.1)** | **0.2 (0.0, 0.6)** |
| Childhood zinc deficiency | 9.2 (1.6, 43.0) | 0.8 (0.1, 4.0) | 0.2 (0.0, 0.8) |
| Childhood diarrhea | 7.2 (2.9, 11.9) | 0.6 (0.1, 1.5) | 0.1 (0.0, 0.3) |
| Non-exclusive breastfeeding | 2.2 (0.7, 4.3) | 0.2 (0.0, 0.5) | 0.0 (0.0, 0.1) |
| HIV infection without HAART before 2 years | No data | No data | No data |
| Discontinued breastfeeding | 1.5 (0.3, 3.2) | 0.1 (0.0, 0.4) | 0.0 (0.0, 0.1) |
| **Water, sanitation and biomass fuel use** | **0.0 (-0.2, 0.1)** | **0.0 (0.0, 0.0)** | **0.0 (0.0, 0.0)** |
| Unimproved sanitation | 0.0 (0.0, 0.0) | 0.0 (0.0, 0.0) | 0.0 (0.0, 0.0) |
| Unimproved water | 0.0 (0.0, 0.0) | 0.0 (0.0, 0.0) | 0.0 (0.0, 0.0) |
| Use of biomass fuels | 0.0 (-0.2, 0.1) | 0.0 (0.0, 0.0) | 0.0 (0.0, 0.0) |

**Fig 1: Stunting cases among 2-year olds attributable to individual risk factors**


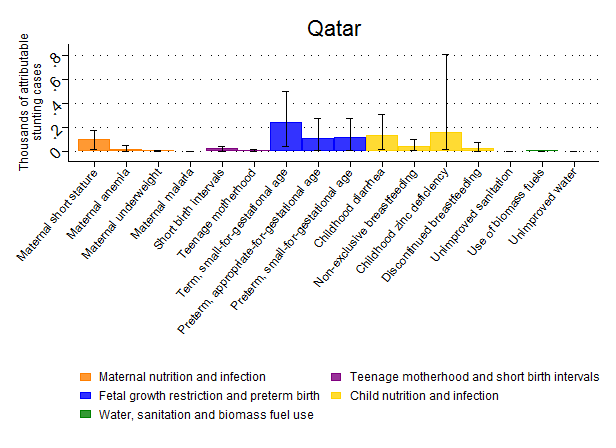


Rwanda

**Region: Sub-Saharan Africa; Sub-region: Sub-Saharan Africa, East**

**Stunting prevalence among children age 2: 49.8%**

**Stunting cases among children age 2: 180,207**

**Population of children age 2: 361,746**

This country profile provides results for 2011 from the analysis presented in *Risk factors for childhood stunting in 137 developing countries: a comparative risk assessment analysis at global, regional, and country levels.*

**Table 1: Population attributable fraction (PAF in %), attributable stunting prevalence (percentage points) and number of stunting cases among 2-year olds attributable to individual risks and risk factor clusters (95% confidence intervals presented in parentheses)**

| **Description** | **PAF** | **Attributable stunting prevalence** | **Attributable stunting cases (thousands)** |
| --- | --- | --- | --- |
| **Maternal nutrition and infection** | **9.0 (8.4, 9.7)** | **4.5 (3.8, 5.2)** | **16.1 (13.6, 18.8)** |
| Maternal short stature | 7.7 (7.5, 7.9) | 3.8 (3.3, 4.4) | 13.9 (11.9, 15.9) |
| Maternal underweight | 0.6 (0.3, 1.1) | 0.3 (0.1, 0.6) | 1.1 (0.5, 2.0) |
| Maternal malaria | 0.1 (0.0, 0.2) | 0.1 (0.0, 0.1) | 0.2 (0.1, 0.4) |
| Maternal anemia | 0.6 (0.2, 1.1) | 0.3 (0.1, 0.5) | 1.1 (0.3, 1.9) |
| **Teenage motherhood and short birth intervals** | **1.2 (1.1, 1.2)** | **0.6 (0.5, 0.7)** | **2.1 (1.8, 2.4)** |
| Teenage motherhood | 0.3 (0.3, 0.3) | 0.2 (0.1, 0.2) | 0.6 (0.5, 0.7) |
| Short birth intervals | 0.9 (0.8, 0.9) | 0.4 (0.4, 0.5) | 1.5 (1.3, 1.8) |
| **Fetal growth restriction and preterm birth** | **20.5 (14.8, 25.5)** | **10.2 (7.2, 13.3)** | **37.0 (26.0, 47.9)** |
| Preterm, small-for-gestational age | 4.2 (0.1, 8.7) | 2.1 (0.0, 4.3) | 7.5 (0.1, 15.7) |
| Preterm, appropriate-for-gestational age | 5.7 (0.2, 11.1) | 2.8 (0.1, 5.5) | 10.2 (0.3, 20.0) |
| Term, small-for-gestational age | 12.1 (8.8, 15.7) | 6.0 (4.1, 8.1) | 21.8 (14.9, 29.4) |
| Low birth weight | 10.8 (8.9, 12.8) | 5.4 (4.2, 6.6) | 19.5 (15.1, 24.0) |
| **Child nutrition and infection** | **14.4 (5.7, 24.1)** | **7.1 (2.9, 12.0)** | **25.8 (10.3, 43.5)** |
| Childhood zinc deficiency | 1.1 (0.5, 1.9) | 0.6 (0.2, 1.0) | 2.1 (0.8, 3.5) |
| Childhood diarrhea | 13.9 (5.3, 23.5) | 6.9 (2.6, 11.7) | 24.9 (9.5, 42.5) |
| Non-exclusive breastfeeding | 0.7 (-1.2, 2.7) | 0.4 (-0.6, 1.4) | 1.3 (-2.2, 4.9) |
| HIV infection without HAART before 2 years | 0.1 (0.1, 0.2) | 0.1 (0.0, 0.1) | 0.2 (0.1, 0.4) |
| Discontinued breastfeeding | 0.5 (0.0, 1.5) | 0.3 (0.0, 0.7) | 1.0 (0.0, 2.7) |
| **Water, sanitation and biomass fuel use** | **16.5 (13.2, 19.7)** | **8.2 (6.3, 10.2)** | **29.7 (22.7, 37.0)** |
| Unimproved sanitation | 11.7 (8.5, 15.0) | 5.8 (4.1, 7.6) | 21.2 (14.9, 27.7) |
| Unimproved water | 2.4 (1.3, 3.6) | 1.2 (0.7, 1.8) | 4.3 (2.4, 6.6) |
| Use of biomass fuels | 3.0 (2.2, 3.9) | 1.5 (1.0, 2.0) | 5.5 (3.8, 7.4) |

**Fig 1: Stunting cases among 2-year olds attributable to individual risk factors**


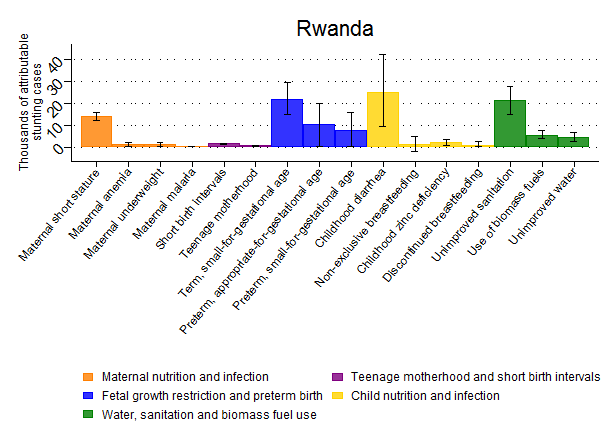


Samoa

**Region: East Asia/Pacific; Sub-region: Oceania**

**Stunting prevalence among children age 2: 14.8%**

**Stunting cases among children age 2: 764**

**Population of children age 2: 5,176**

This country profile provides results for 2011 from the analysis presented in *Risk factors for childhood stunting in 137 developing countries: a comparative risk assessment analysis at global, regional, and country levels.*

**Table 1: Population attributable fraction (PAF in %), attributable stunting prevalence (percentage points) and number of stunting cases among 2-year olds attributable to individual risks and risk factor clusters (95% confidence intervals presented in parentheses)**

| **Description** | **PAF** | **Attributable stunting prevalence** | **Attributable stunting cases (thousands)** |
| --- | --- | --- | --- |
| **Maternal nutrition and infection** | **3.2 (3.0, 3.4)** | **0.5 (0.2, 0.8)** | **0.0 (0.0, 0.0)** |
| Maternal short stature | 3.0 (2.9, 3.2) | 0.4 (0.2, 0.8) | 0.0 (0.0, 0.0) |
| Maternal underweight | 0.0 (0.0, 0.0) | 0.0 (0.0, 0.0) | 0.0 (0.0, 0.0) |
| Maternal malaria | 0.0 (0.0, 0.0) | 0.0 (0.0, 0.0) | 0.0 (0.0, 0.0) |
| Maternal anemia | 0.2 (0.0, 0.4) | 0.0 (0.0, 0.1) | 0.0 (0.0, 0.0) |
| **Teenage motherhood and short birth intervals** | **1.3 (1.2, 1.4)** | **0.2 (0.1, 0.3)** | **0.0 (0.0, 0.0)** |
| Teenage motherhood | 0.5 (0.4, 0.5) | 0.1 (0.0, 0.1) | 0.0 (0.0, 0.0) |
| Short birth intervals | 0.8 (0.8, 0.9) | 0.1 (0.0, 0.2) | 0.0 (0.0, 0.0) |
| **Fetal growth restriction and preterm birth** | **9.1 (4.2, 14.0)** | **1.3 (0.4, 2.7)** | **0.1 (0.0, 0.1)** |
| Preterm, small-for-gestational age | 3.2 (-0.2, 7.1) | 0.5 (0.0, 1.3) | 0.0 (0.0, 0.1) |
| Preterm, appropriate-for-gestational age | 2.9 (-0.5, 6.2) | 0.4 (-0.1, 1.1) | 0.0 (0.0, 0.1) |
| Term, small-for-gestational age | 3.2 (0.2, 6.0) | 0.5 (0.0, 1.2) | 0.0 (0.0, 0.1) |
| Low birth weight | 2.3 (1.8, 2.7) | 0.3 (0.1, 0.6) | 0.0 (0.0, 0.0) |
| **Child nutrition and infection** | **11.4 (4.4, 20.0)** | **1.7 (0.4, 3.6)** | **0.1 (0.0, 0.2)** |
| Childhood zinc deficiency | 1.9 (0.3, 12.3) | 0.3 (0.0, 2.0) | 0.0 (0.0, 0.1) |
| Childhood diarrhea | 10.6 (4.1, 18.1) | 1.6 (0.4, 3.3) | 0.1 (0.0, 0.2) |
| Non-exclusive breastfeeding | 1.3 (-0.6, 3.8) | 0.2 (-0.1, 0.6) | 0.0 (0.0, 0.0) |
| HIV infection without HAART before 2 years | No data | No data | No data |
| Discontinued breastfeeding | 1.0 (0.1, 2.5) | 0.1 (0.0, 0.4) | 0.0 (0.0, 0.0) |
| **Water, sanitation and biomass fuel use** | **3.8 (2.9, 4.7)** | **0.6 (0.2, 1.0)** | **0.0 (0.0, 0.1)** |
| Unimproved sanitation | 3.2 (2.4, 4.1) | 0.5 (0.2, 0.9) | 0.0 (0.0, 0.0) |
| Unimproved water | 0.2 (0.0, 0.3) | 0.0 (0.0, 0.1) | 0.0 (0.0, 0.0) |
| Use of biomass fuels | 0.4 (0.3, 0.6) | 0.1 (0.0, 0.1) | 0.0 (0.0, 0.0) |

**Fig 1: Stunting cases among 2-year olds attributable to individual risk factors**


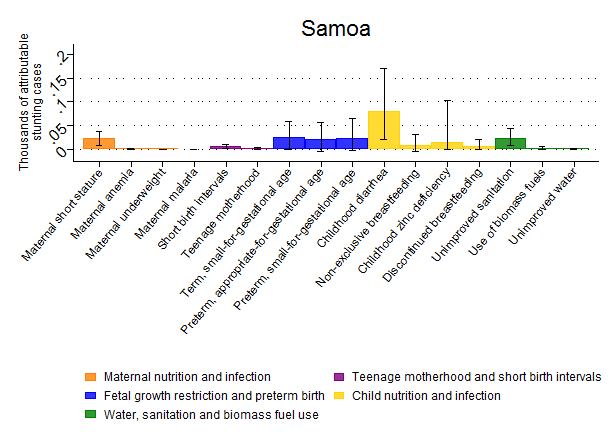


Sao Tome and Principe

**Region: Sub-Saharan Africa; Sub-region: Sub-Saharan Africa, West**

**Stunting prevalence among children age 2: 24.3%**

**Stunting cases among children age 2: 1,483**

**Population of children age 2: 6,104**

This country profile provides results for 2011 from the analysis presented in *Risk factors for childhood stunting in 137 developing countries: a comparative risk assessment analysis at global, regional, and country levels.*

**Table 1: Population attributable fraction (PAF in %), attributable stunting prevalence (percentage points) and number of stunting cases among 2-year olds attributable to individual risks and risk factor clusters (95% confidence intervals presented in parentheses)**

| **Description** | **PAF** | **Attributable stunting prevalence** | **Attributable stunting cases (thousands)** |
| --- | --- | --- | --- |
| **Maternal nutrition and infection** | **8.1 (6.9, 9.5)** | **2.0 (1.5, 2.5)** | **0.1 (0.1, 0.2)** |
| Maternal short stature | 6.0 (5.8, 6.2) | 1.4 (1.2, 1.7) | 0.1 (0.1, 0.1) |
| Maternal underweight | 0.7 (0.3, 1.4) | 0.2 (0.1, 0.4) | 0.0 (0.0, 0.0) |
| Maternal malaria | 0.0 (0.0, 0.0) | 0.0 (0.0, 0.0) | 0.0 (0.0, 0.0) |
| Maternal anemia | 1.6 (0.4, 2.7) | 0.4 (0.1, 0.7) | 0.0 (0.0, 0.0) |
| **Teenage motherhood and short birth intervals** | **1.5 (1.4, 1.7)** | **0.4 (0.3, 0.5)** | **0.0 (0.0, 0.0)** |
| Teenage motherhood | 1.1 (1.0, 1.2) | 0.3 (0.2, 0.3) | 0.0 (0.0, 0.0) |
| Short birth intervals | 0.5 (0.4, 0.5) | 0.1 (0.1, 0.1) | 0.0 (0.0, 0.0) |
| **Fetal growth restriction and preterm birth** | **23.0 (18.0, 27.9)** | **5.6 (4.0, 7.4)** | **0.3 (0.2, 0.5)** |
| Preterm, small-for-gestational age | 3.9 (0.0, 7.9) | 0.9 (0.0, 1.9) | 0.1 (0.0, 0.1) |
| Preterm, appropriate-for-gestational age | 5.7 (0.7, 11.0) | 1.4 (0.2, 2.8) | 0.1 (0.0, 0.2) |
| Term, small-for-gestational age | 15.1 (11.6, 19.1) | 3.7 (2.6, 4.9) | 0.2 (0.2, 0.3) |
| Low birth weight | 13.1 (10.8, 15.3) | 3.2 (2.4, 4.0) | 0.2 (0.1, 0.2) |
| **Child nutrition and infection** | **12.5 (4.5, 22.1)** | **3.0 (1.1, 5.5)** | **0.2 (0.1, 0.3)** |
| Childhood zinc deficiency | 1.4 (0.5, 2.5) | 0.3 (0.1, 0.6) | 0.0 (0.0, 0.0) |
| Childhood diarrhea | 11.9 (4.0, 21.6) | 2.9 (1.0, 5.3) | 0.2 (0.1, 0.3) |
| Non-exclusive breastfeeding | 1.2 (-1.0, 3.8) | 0.3 (-0.2, 0.9) | 0.0 (0.0, 0.1) |
| HIV infection without HAART before 2 years | 0.2 (0.1, 0.6) | 0.1 (0.0, 0.1) | 0.0 (0.0, 0.0) |
| Discontinued breastfeeding | 0.6 (0.0, 1.6) | 0.1 (0.0, 0.4) | 0.0 (0.0, 0.0) |
| **Water, sanitation and biomass fuel use** | **23.4 (20.9, 26.0)** | **5.7 (4.4, 7.1)** | **0.3 (0.3, 0.4)** |
| Unimproved sanitation | 20.5 (18.0, 23.1) | 5.0 (3.9, 6.3) | 0.3 (0.2, 0.4) |
| Unimproved water | 0.8 (0.1, 1.6) | 0.2 (0.0, 0.4) | 0.0 (0.0, 0.0) |
| Use of biomass fuels | 2.9 (2.0, 3.8) | 0.7 (0.5, 1.0) | 0.0 (0.0, 0.1) |

**Fig 1: Stunting cases among 2-year olds attributable to individual risk factors**

Saudi Arabia

**Region: North Africa/Middle East; Sub-region: North Africa / Middle East**

**Stunting prevalence among children age 2: 13.1%**

**Stunting cases among children age 2: 79,764**

**Population of children age 2: 609,352**

This country profile provides results for 2011 from the analysis presented in *Risk factors for childhood stunting in 137 developing countries: a comparative risk assessment analysis at global, regional, and country levels.*

**Table 1: Population attributable fraction (PAF in %), attributable stunting prevalence (percentage points) and number of stunting cases among 2-year olds attributable to individual risks and risk factor clusters (95% confidence intervals presented in parentheses)**

| **Description** | **PAF** | **Attributable stunting prevalence** | **Attributable stunting cases (thousands)** |
| --- | --- | --- | --- |
| **Maternal nutrition and infection** | **10.6 (9.7, 11.8)** | **1.4 (0.8, 2.0)** | **8.5 (5.0, 12.3)** |
| Maternal short stature | 9.2 (9.0, 9.4) | 1.2 (0.7, 1.7) | 7.3 (4.3, 10.4) |
| Maternal underweight | 0.2 (0.1, 0.4) | 0.0 (0.0, 0.1) | 0.2 (0.1, 0.4) |
| Maternal malaria | 0.0 (0.0, 0.0) | 0.0 (0.0, 0.0) | 0.0 (0.0, 0.0) |
| Maternal anemia | 1.3 (0.3, 2.6) | 0.2 (0.0, 0.4) | 1.1 (0.3, 2.3) |
| **Teenage motherhood and short birth intervals** | **1.8 (1.7, 1.9)** | **0.2 (0.1, 0.3)** | **1.4 (0.8, 2.0)** |
| Teenage motherhood | 0.5 (0.5, 0.6) | 0.1 (0.0, 0.1) | 0.4 (0.2, 0.6) |
| Short birth intervals | 1.2 (1.1, 1.3) | 0.2 (0.1, 0.2) | 1.0 (0.6, 1.4) |
| **Fetal growth restriction and preterm birth** | **20.6 (15.5, 25.5)** | **2.7 (1.5, 4.0)** | **16.4 (9.0, 24.5)** |
| Preterm, small-for-gestational age | 3.6 (2.0, 5.6) | 0.5 (0.2, 0.8) | 2.9 (1.4, 5.1) |
| Preterm, appropriate-for-gestational age | 3.4 (2.0, 4.9) | 0.4 (0.2, 0.8) | 2.7 (1.3, 4.6) |
| Term, small-for-gestational age | 14.7 (9.3, 20.4) | 1.9 (1.0, 3.1) | 11.7 (5.9, 19.1) |
| Low birth weight | 12.6 (10.5, 14.8) | 1.7 (0.9, 2.5) | 10.1 (5.8, 14.9) |
| **Child nutrition and infection** | **8.8 (3.4, 16.8)** | **1.2 (0.4, 2.4)** | **7.0 (2.4, 14.7)** |
| Childhood zinc deficiency | 3.5 (0.6, 16.1) | 0.5 (0.1, 2.0) | 2.8 (0.5, 11.9) |
| Childhood diarrhea | 7.2 (2.8, 12.1) | 0.9 (0.3, 1.8) | 5.7 (2.1, 10.9) |
| Non-exclusive breastfeeding | 1.1 (-0.1, 2.7) | 0.1 (0.0, 0.4) | 0.9 (-0.1, 2.4) |
| HIV infection without HAART before 2 years | No data | No data | No data |
| Discontinued breastfeeding | 1.0 (0.2, 2.4) | 0.1 (0.0, 0.3) | 0.8 (0.1, 2.0) |
| **Water, sanitation and biomass fuel use** | **0.6 (-0.4, 1.5)** | **0.1 (-0.1, 0.2)** | **0.5 (-0.3, 1.4)** |
| Unimproved sanitation | 0.3 (-0.6, 1.3) | 0.0 (-0.1, 0.2) | 0.3 (-0.5, 1.1) |
| Unimproved water | 0.3 (0.1, 0.5) | 0.0 (0.0, 0.1) | 0.2 (0.0, 0.5) |
| Use of biomass fuels | 0.0 (-0.2, 0.2) | 0.0 (0.0, 0.0) | 0.0 (-0.2, 0.2) |

**Fig 1: Stunting cases among 2-year olds attributable to individual risk factors**

Senegal

**Region: Sub-Saharan Africa; Sub-region: Sub-Saharan Africa, West**

**Stunting prevalence among children age 2: 30.1%**

**Stunting cases among children age 2: 153,332**

**Population of children age 2: 508,917**

This country profile provides results for 2011 from the analysis presented in *Risk factors for childhood stunting in 137 developing countries: a comparative risk assessment analysis at global, regional, and country levels.*

**Table 1: Population attributable fraction (PAF in %), attributable stunting prevalence (percentage points) and number of stunting cases among 2-year olds attributable to individual risks and risk factor clusters (95% confidence intervals presented in parentheses)**

| **Description** | **PAF** | **Attributable stunting prevalence** | **Attributable stunting cases (thousands)** |
| --- | --- | --- | --- |
| **Maternal nutrition and infection** | **9.8 (6.8, 12.8)** | **2.9 (2.0, 4.1)** | **15.0 (9.9, 20.9)** |
| Maternal short stature | 3.0 (2.9, 3.1) | 0.9 (0.7, 1.1) | 4.6 (3.7, 5.4) |
| Maternal underweight | 2.1 (1.0, 3.4) | 0.6 (0.3, 1.1) | 3.2 (1.5, 5.4) |
| Maternal malaria | 1.0 (0.4, 1.6) | 0.3 (0.1, 0.5) | 1.5 (0.5, 2.6) |
| Maternal anemia | 4.1 (1.2, 6.7) | 1.2 (0.3, 2.1) | 6.3 (1.8, 10.9) |
| **Teenage motherhood and short birth intervals** | **1.9 (1.8, 2.0)** | **0.6 (0.5, 0.7)** | **3.0 (2.4, 3.6)** |
| Teenage motherhood | 1.1 (1.0, 1.2) | 0.3 (0.3, 0.4) | 1.7 (1.3, 2.0) |
| Short birth intervals | 0.9 (0.8, 0.9) | 0.3 (0.2, 0.3) | 1.3 (1.1, 1.6) |
| **Fetal growth restriction and preterm birth** | **32.0 (26.9, 36.6)** | **9.6 (7.4, 12.0)** | **49.0 (37.5, 61.2)** |
| Preterm, small-for-gestational age | 3.7 (-0.1, 7.9) | 1.1 (0.0, 2.4) | 5.7 (-0.2, 12.5) |
| Preterm, appropriate-for-gestational age | 5.1 (0.1, 10.3) | 1.5 (0.0, 3.1) | 7.8 (0.1, 15.8) |
| Term, small-for-gestational age | 25.6 (20.6, 30.3) | 7.7 (5.8, 9.8) | 39.2 (29.6, 49.8) |
| Low birth weight | 26.6 (22.7, 30.4) | 8.0 (6.1, 9.9) | 40.7 (30.9, 50.6) |
| **Child nutrition and infection** | **13.4 (5.5, 23.6)** | **4.1 (1.6, 7.3)** | **20.6 (8.1, 37.2)** |
| Childhood zinc deficiency | 1.3 (0.5, 2.3) | 0.4 (0.1, 0.7) | 1.9 (0.7, 3.6) |
| Childhood diarrhea | 12.9 (5.0, 23.0) | 3.9 (1.4, 7.1) | 19.8 (7.3, 36.3) |
| Non-exclusive breastfeeding | 1.4 (-0.9, 3.8) | 0.4 (-0.3, 1.2) | 2.1 (-1.3, 6.0) |
| Discontinued breastfeeding | 0.6 (0.0, 1.6) | 0.2 (0.0, 0.5) | 0.9 (0.0, 2.6) |
| HIV infection without HAART before 2 years | 0.1 (0.0, 0.3) | 0.0 (0.0, 0.1) | 0.1 (0.1, 0.4) |
| **Water, sanitation and biomass fuel use** | **21.0 (18.4, 23.6)** | **6.3 (4.9, 7.9)** | **32.2 (25.0, 40.0)** |
| Unimproved sanitation | 15.1 (13.0, 17.3) | 4.5 (3.5, 5.7) | 23.2 (17.8, 29.0) |
| Unimproved water | 2.2 (1.3, 3.3) | 0.7 (0.3, 1.0) | 3.4 (1.8, 5.2) |
| Use of biomass fuels | 4.8 (3.2, 6.7) | 1.5 (0.9, 2.1) | 7.4 (4.6, 10.9) |

**Fig 1: Stunting cases among 2-year olds attributable to individual risk factors**

Seychelles

**Region: East Asia/Pacific; Sub-region: Asia, Southeast**

**Stunting prevalence among children age 2: 12.9%**

**Stunting cases among children age 2: 223**

**Population of children age 2: 1,733**

This country profile provides results for 2011 from the analysis presented in *Risk factors for childhood stunting in 137 developing countries: a comparative risk assessment analysis at global, regional, and country levels.*

**Table 1: Population attributable fraction (PAF in %), attributable stunting prevalence (percentage points) and number of stunting cases among 2-year olds attributable to individual risks and risk factor clusters (95% confidence intervals presented in parentheses)**

| **Description** | **PAF** | **Attributable stunting prevalence** | **Attributable stunting cases (thousands)** |
| --- | --- | --- | --- |
| **Maternal nutrition and infection** | **5.1 (4.0, 6.4)** | **0.7 (0.2, 1.2)** | **0.0 (0.0, 0.0)** |
| Maternal short stature | 3.5 (3.4, 3.7) | 0.5 (0.1, 0.8) | 0.0 (0.0, 0.0) |
| Maternal underweight | 0.5 (0.1, 0.9) | 0.1 (0.0, 0.1) | 0.0 (0.0, 0.0) |
| Maternal malaria | 0.0 (0.0, 0.0) | 0.0 (0.0, 0.0) | 0.0 (0.0, 0.0) |
| Maternal anemia | 1.2 (0.2, 2.4) | 0.1 (0.0, 0.4) | 0.0 (0.0, 0.0) |
| **Teenage motherhood and short birth intervals** | **1.3 (1.2, 1.4)** | **0.2 (0.0, 0.3)** | **0.0 (0.0, 0.0)** |
| Teenage motherhood | 0.5 (0.4, 0.5) | 0.1 (0.0, 0.1) | 0.0 (0.0, 0.0) |
| Short birth intervals | 0.8 (0.8, 0.9) | 0.1 (0.0, 0.2) | 0.0 (0.0, 0.0) |
| **Fetal growth restriction and preterm birth** | **22.7 (16.7, 28.7)** | **2.9 (0.8, 5.5)** | **0.1 (0.0, 0.1)** |
| Preterm, small-for-gestational age | 4.0 (-0.5, 8.9) | 0.5 (-0.1, 1.4) | 0.0 (0.0, 0.0) |
| Preterm, appropriate-for-gestational age | 5.3 (-0.2, 11.3) | 0.7 (0.0, 1.7) | 0.0 (0.0, 0.0) |
| Term, small-for-gestational age | 15.0 (10.3, 19.6) | 1.9 (0.6, 3.7) | 0.0 (0.0, 0.1) |
| Low birth weight | 16.1 (13.5, 18.8) | 2.1 (0.5, 3.7) | 0.0 (0.0, 0.1) |
| **Child nutrition and infection** | **11.9 (4.8, 20.9)** | **1.5 (0.3, 3.4)** | **0.0 (0.0, 0.1)** |
| Childhood zinc deficiency | 3.2 (0.4, 18.6) | 0.4 (0.0, 1.9) | 0.0 (0.0, 0.0) |
| Childhood diarrhea | 10.5 (4.1, 16.8) | 1.4 (0.3, 2.9) | 0.0 (0.0, 0.0) |
| Non-exclusive breastfeeding | 1.7 (-0.2, 4.1) | 0.2 (0.0, 0.6) | 0.0 (0.0, 0.0) |
| Discontinued breastfeeding | 1.5 (0.2, 3.4) | 0.2 (0.0, 0.6) | 0.0 (0.0, 0.0) |
| HIV infection without HAART before 2 years | No data | No data | No data |
| **Water, sanitation and biomass fuel use** | **3.9 (2.9, 4.9)** | **0.5 (0.1, 0.9)** | **0.0 (0.0, 0.0)** |
| Unimproved sanitation | 3.6 (2.6, 4.5) | 0.5 (0.1, 0.9) | 0.0 (0.0, 0.0) |
| Unimproved water | 0.3 (0.1, 0.6) | 0.0 (0.0, 0.1) | 0.0 (0.0, 0.0) |
| Use of biomass fuels | 0.0 (-0.3, 0.2) | 0.0 (0.0, 0.0) | 0.0 (0.0, 0.0) |

**Fig 1: Stunting cases among 2-year olds attributable to individual risk factors**

Sierra Leone

**Region: Sub-Saharan Africa; Sub-region: Sub-Saharan Africa, West**

**Stunting prevalence among children age 2: 44.0%**

**Stunting cases among children age 2: 98,944**

**Population of children age 2: 224,862**

This country profile provides results for 2011 from the analysis presented in *Risk factors for childhood stunting in 137 developing countries: a comparative risk assessment analysis at global, regional, and country levels.*

**Table 1: Population attributable fraction (PAF in %), attributable stunting prevalence (percentage points) and number of stunting cases among 2-year olds attributable to individual risks and risk factor clusters (95% confidence intervals presented in parentheses)**

| **Description** | **PAF** | **Attributable stunting prevalence** | **Attributable stunting cases (thousands)** |
| --- | --- | --- | --- |
| **Maternal nutrition and infection** | **13.5 (11.3, 15.8)** | **5.9 (4.3, 7.7)** | **13.3 (9.8, 17.4)** |
| Maternal short stature | 7.6 (7.4, 7.8) | 3.4 (2.6, 4.1) | 7.6 (5.8, 9.3) |
| Maternal underweight | 1.5 (0.7, 2.4) | 0.6 (0.3, 1.1) | 1.4 (0.7, 2.5) |
| Maternal malaria | 2.6 (1.0, 4.2) | 1.1 (0.4, 1.9) | 2.5 (0.9, 4.2) |
| Maternal anemia | 2.4 (0.7, 4.3) | 1.1 (0.3, 2.0) | 2.4 (0.7, 4.5) |
| **Teenage motherhood and short birth intervals** | **1.9 (1.8, 2.1)** | **0.9 (0.7, 1.1)** | **1.9 (1.5, 2.4)** |
| Teenage motherhood | 1.1 (1.0, 1.2) | 0.5 (0.4, 0.6) | 1.1 (0.8, 1.4) |
| Short birth intervals | 0.8 (0.8, 0.9) | 0.4 (0.3, 0.5) | 0.8 (0.6, 1.0) |
| **Fetal growth restriction and preterm birth** | **31.6 (27.1, 35.5)** | **13.9 (10.3, 17.8)** | **31.3 (23.1, 39.9)** |
| Preterm, small-for-gestational age | 3.7 (0.6, 7.3) | 1.6 (0.2, 3.3) | 3.7 (0.5, 7.4) |
| Preterm, appropriate-for-gestational age | 5.1 (0.9, 9.5) | 2.2 (0.4, 4.2) | 5.0 (0.9, 9.4) |
| Term, small-for-gestational age | 25.1 (20.8, 29.4) | 11.0 (8.0, 14.4) | 24.8 (18.1, 32.3) |
| Low birth weight | 20.7 (17.5, 24.0) | 9.1 (6.7, 11.6) | 20.5 (15.0, 26.0) |
| **Child nutrition and infection** | **16.4 (6.4, 28.3)** | **7.2 (2.6, 13.2)** | **16.3 (5.9, 29.7)** |
| Childhood zinc deficiency | 1.2 (0.5, 2.1) | 0.5 (0.2, 1.0) | 1.2 (0.4, 2.2) |
| Childhood diarrhea | 15.9 (6.0, 27.9) | 7.0 (2.5, 13.0) | 15.8 (5.6, 29.3) |
| Non-exclusive breastfeeding | 2.3 (0.0, 5.8) | 1.0 (0.0, 2.5) | 2.3 (0.0, 5.6) |
| HIV infection without HAART before 2 years | 0.2 (0.1, 0.5) | 0.1 (0.0, 0.2) | 0.2 (0.1, 0.5) |
| Discontinued breastfeeding | 0.9 (0.1, 2.5) | 0.4 (0.0, 1.1) | 0.9 (0.1, 2.5) |
| **Water, sanitation and biomass fuel use** | **30.9 (28.7, 33.1)** | **13.6 (10.4, 16.7)** | **30.6 (23.4, 37.6)** |
| Unimproved sanitation | 24.1 (22.2, 26.1) | 10.6 (8.1, 13.2) | 23.9 (18.1, 29.6) |
| Unimproved water | 3.4 (2.1, 4.8) | 1.5 (0.9, 2.2) | 3.3 (1.9, 5.1) |
| Use of biomass fuels | 5.8 (4.2, 7.5) | 2.6 (1.7, 3.5) | 5.8 (3.8, 7.9) |

**Fig 1: Stunting cases among 2-year olds attributable to individual risk factors**

Solomon Islands

**Region: East Asia/Pacific; Sub-region: Oceania**

**Stunting prevalence among children age 2: 34.0%**

**Stunting cases among children age 2: 5,795**

**Population of children age 2: 17,019**

This country profile provides results for 2011 from the analysis presented in *Risk factors for childhood stunting in 137 developing countries: a comparative risk assessment analysis at global, regional, and country levels.*

**Table 1: Population attributable fraction (PAF in %), attributable stunting prevalence (percentage points) and number of stunting cases among 2-year olds attributable to individual risks and risk factor clusters (95% confidence intervals presented in parentheses)**

| **Description** | **PAF** | **Attributable stunting prevalence** | **Attributable stunting cases (thousands)** |
| --- | --- | --- | --- |
| **Maternal nutrition and infection** | **10.5 (9.0, 12.2)** | **3.6 (2.7, 4.6)** | **0.6 (0.5, 0.8)** |
| Maternal short stature | 8.5 (8.2, 8.9) | 2.9 (2.3, 3.6) | 0.5 (0.4, 0.6) |
| Maternal underweight | 0.5 (0.1, 0.9) | 0.2 (0.0, 0.3) | 0.0 (0.0, 0.1) |
| Maternal malaria | 0.0 (0.0, 0.0) | 0.0 (0.0, 0.0) | 0.0 (0.0, 0.0) |
| Maternal anemia | 1.7 (0.3, 3.6) | 0.6 (0.1, 1.3) | 0.1 (0.0, 0.2) |
| **Teenage motherhood and short birth intervals** | **1.3 (1.2, 1.4)** | **0.4 (0.3, 0.5)** | **0.1 (0.1, 0.1)** |
| Teenage motherhood | 0.5 (0.4, 0.5) | 0.2 (0.1, 0.2) | 0.0 (0.0, 0.0) |
| Short birth intervals | 0.8 (0.8, 0.9) | 0.3 (0.2, 0.4) | 0.0 (0.0, 0.1) |
| **Fetal growth restriction and preterm birth** | **30.0 (24.2, 35.3)** | **10.2 (7.4, 13.2)** | **1.7 (1.3, 2.3)** |
| Preterm, small-for-gestational age | 6.4 (0.6, 12.6) | 2.2 (0.2, 4.3) | 0.4 (0.0, 0.7) |
| Preterm, appropriate-for-gestational age | 6.1 (0.9, 11.5) | 2.1 (0.3, 4.1) | 0.4 (0.0, 0.7) |
| Term, small-for-gestational age | 20.3 (15.5, 25.9) | 6.9 (4.7, 9.5) | 1.2 (0.8, 1.6) |
| Low birth weight | 19.4 (16.3, 22.5) | 6.6 (4.8, 8.4) | 1.1 (0.8, 1.4) |
| **Child nutrition and infection** | **11.7 (4.9, 19.2)** | **4.0 (1.6, 6.8)** | **0.7 (0.3, 1.2)** |
| Childhood zinc deficiency | 1.4 (0.5, 2.6) | 0.5 (0.2, 0.9) | 0.1 (0.0, 0.2) |
| Childhood diarrhea | 11.0 (4.4, 18.5) | 3.8 (1.5, 6.6) | 0.6 (0.2, 1.1) |
| Non-exclusive breastfeeding | 0.7 (-1.7, 2.7) | 0.2 (-0.6, 0.9) | 0.0 (-0.1, 0.2) |
| Discontinued breastfeeding | 0.7 (0.1, 1.9) | 0.2 (0.0, 0.7) | 0.0 (0.0, 0.1) |
| HIV infection without HAART before 2 years | No data | No data | No data |
| **Water, sanitation and biomass fuel use** | **25.8 (23.3, 28.3)** | **8.8 (6.6, 11.0)** | **1.5 (1.1, 1.9)** |
| Unimproved sanitation | 20.6 (18.5, 22.8) | 7.0 (5.3, 8.8) | 1.2 (0.9, 1.5) |
| Unimproved water | 1.4 (0.6, 2.3) | 0.5 (0.2, 0.8) | 0.1 (0.0, 0.1) |
| Use of biomass fuels | 5.2 (3.6, 6.9) | 1.8 (1.2, 2.4) | 0.3 (0.2, 0.4) |

**Fig 1: Stunting cases among 2-year olds attributable to individual risk factors**

Somalia

**Region: Sub-Saharan Africa; Sub-region: Sub-Saharan Africa, East**

**Stunting prevalence among children age 2: 49.5%**

**Stunting cases among children age 2: 213,618**

**Population of children age 2: 431,966**

This country profile provides results for 2011 from the analysis presented in *Risk factors for childhood stunting in 137 developing countries: a comparative risk assessment analysis at global, regional, and country levels.*

**Table 1: Population attributable fraction (PAF in %), attributable stunting prevalence (percentage points) and number of stunting cases among 2-year olds attributable to individual risks and risk factor clusters (95% confidence intervals presented in parentheses)**

| **Description** | **PAF** | **Attributable stunting prevalence** | **Attributable stunting cases (thousands)** |
| --- | --- | --- | --- |
| **Maternal nutrition and infection** | **13.2 (11.5, 15.2)** | **6.6 (4.5, 8.6)** | **28.3 (19.6, 37.3)** |
| Maternal short stature | 10.0 (9.1, 10.7) | 4.9 (3.4, 6.4) | 21.3 (14.8, 27.7) |
| Maternal underweight | 1.5 (0.5, 2.6) | 0.7 (0.3, 1.4) | 3.2 (1.2, 6.0) |
| Maternal malaria | 0.1 (0.0, 0.2) | 0.1 (0.0, 0.1) | 0.3 (0.1, 0.5) |
| Maternal anemia | 2.1 (0.6, 3.7) | 1.0 (0.3, 1.8) | 4.4 (1.2, 8.0) |
| **Teenage motherhood and short birth intervals** | **1.9 (1.8, 2.0)** | **0.9 (0.7, 1.2)** | **4.0 (2.8, 5.2)** |
| Teenage motherhood | 1.0 (0.9, 1.0) | 0.5 (0.3, 0.6) | 2.0 (1.4, 2.6) |
| Short birth intervals | 1.0 (0.9, 1.0) | 0.5 (0.3, 0.6) | 2.0 (1.4, 2.7) |
| **Fetal growth restriction and preterm birth** | **30.3 (25.8, 34.7)** | **15.0 (10.2, 20.2)** | **64.7 (44.2, 87.3)** |
| Preterm, small-for-gestational age | 4.5 (1.1, 8.4) | 2.2 (0.5, 4.4) | 9.5 (2.2, 18.8) |
| Preterm, appropriate-for-gestational age | 6.1 (1.4, 11.0) | 3.0 (0.7, 5.8) | 13.1 (3.0, 24.9) |
| Term, small-for-gestational age | 22.2 (18.1, 26.7) | 11.0 (7.3, 15.5) | 47.4 (31.7, 66.7) |
| Low birth weight | 17.7 (14.9, 20.6) | 8.8 (5.9, 11.7) | 37.9 (25.5, 50.5) |
| **Child nutrition and infection** | **16.7 (5.8, 29.8)** | **8.2 (2.7, 15.4)** | **35.6 (11.9, 66.3)** |
| Childhood zinc deficiency | 0.8 (0.3, 1.7) | 0.4 (0.1, 0.9) | 1.8 (0.6, 3.8) |
| Childhood diarrhea | 16.3 (5.4, 29.3) | 8.1 (2.5, 15.1) | 34.8 (10.9, 65.4) |
| Non-exclusive breastfeeding | 2.9 (0.4, 7.2) | 1.4 (0.2, 3.7) | 6.1 (0.8, 16.1) |
| HIV infection without HAART before 2 years | No data | No data | No data |
| Discontinued breastfeeding | 1.9 (0.2, 4.7) | 0.9 (0.1, 2.4) | 4.0 (0.5, 10.6) |
| **Water, sanitation and biomass fuel use** | **28.3 (25.4, 31.2)** | **14.0 (9.6, 18.3)** | **60.6 (41.6, 79.2)** |
| Unimproved sanitation | 20.5 (17.8, 23.1) | 10.1 (6.9, 13.4) | 43.8 (29.8, 58.1) |
| Unimproved water | 5.2 (3.4, 7.4) | 2.6 (1.5, 3.9) | 11.2 (6.3, 16.9) |
| Use of biomass fuels | 4.9 (3.5, 6.3) | 2.4 (1.5, 3.5) | 10.5 (6.3, 15.3) |

**Fig 1: Stunting cases among 2-year olds attributable to individual risk factors**

South Africa

**Region: Sub-Saharan Africa; Sub-region: Sub-Saharan Africa, Southern**

**Stunting prevalence among children age 2: 25.5%**

**Stunting cases among children age 2: 283,282**

**Population of children age 2: 1,110,597**

This country profile provides results for 2011 from the analysis presented in *Risk factors for childhood stunting in 137 developing countries: a comparative risk assessment analysis at global, regional, and country levels.*

**Table 1: Population attributable fraction (PAF in %), attributable stunting prevalence (percentage points) and number of stunting cases among 2-year olds attributable to individual risks and risk factor clusters (95% confidence intervals presented in parentheses)**

| **Description** | **PAF** | **Attributable stunting prevalence** | **Attributable stunting cases (thousands)** |
| --- | --- | --- | --- |
| **Maternal nutrition and infection** | **8.7 (7.3, 10.6)** | **2.2 (1.6, 2.9)** | **24.8 (17.9, 32.0)** |
| Maternal short stature | 6.1 (5.9, 6.2) | 1.5 (1.2, 1.9) | 17.2 (13.3, 21.0) |
| Maternal underweight | 0.6 (0.3, 1.1) | 0.2 (0.1, 0.3) | 1.8 (0.7, 3.4) |
| Maternal malaria | 0.4 (0.1, 0.7) | 0.1 (0.0, 0.2) | 1.2 (0.4, 2.1) |
| Maternal anemia | 1.8 (0.4, 3.7) | 0.5 (0.1, 1.0) | 5.1 (1.1, 11.0) |
| **Teenage motherhood and short birth intervals** | **1.7 (1.5, 1.8)** | **0.4 (0.3, 0.5)** | **4.7 (3.6, 5.8)** |
| Teenage motherhood | 1.1 (1.0, 1.2) | 0.3 (0.2, 0.4) | 3.2 (2.4, 4.0) |
| Short birth intervals | 0.5 (0.5, 0.6) | 0.1 (0.1, 0.2) | 1.5 (1.1, 1.9) |
| **Fetal growth restriction and preterm birth** | **27.8 (23.4, 32.3)** | **7.1 (5.2, 9.1)** | **78.8 (57.3, 101.4)** |
| Preterm, small-for-gestational age | 3.3 (0.7, 6.4) | 0.8 (0.2, 1.8) | 9.4 (1.9, 19.7) |
| Preterm, appropriate-for-gestational age | 4.4 (0.7, 8.1) | 1.1 (0.2, 2.1) | 12.6 (1.7, 23.7) |
| Term, small-for-gestational age | 21.9 (17.4, 26.7) | 5.6 (3.9, 7.4) | 62.0 (43.1, 81.9) |
| Low birth weight | 22.5 (19.1, 25.9) | 5.7 (4.2, 7.3) | 63.7 (47.0, 81.3) |
| **Child nutrition and infection** | **11.8 (4.2, 21.1)** | **3.0 (1.0, 5.4)** | **33.3 (11.6, 60.0)** |
| Childhood zinc deficiency | 1.2 (0.4, 2.3) | 0.3 (0.1, 0.6) | 3.3 (1.2, 6.7) |
| Childhood diarrhea | 11.3 (3.9, 20.5) | 2.9 (1.0, 5.3) | 31.9 (10.6, 58.4) |
| Non-exclusive breastfeeding | 2.2 (0.5, 5.3) | 0.6 (0.1, 1.4) | 6.2 (1.2, 15.2) |
| HIV infection without HAART before 2 years | 1.3 (0.4, 4.4) | 0.3 (0.1, 1.2) | 3.6 (1.0, 13.3) |
| Discontinued breastfeeding | 1.3 (0.1, 3.5) | 0.3 (0.0, 0.9) | 3.6 (0.4, 10.0) |
| **Water, sanitation and biomass fuel use** | **9.2 (7.2, 11.1)** | **2.3 (1.6, 3.1)** | **26.0 (18.0, 34.6)** |
| Unimproved sanitation | 7.5 (5.6, 9.4) | 1.9 (1.3, 2.6) | 21.3 (14.4, 28.8) |
| Unimproved water | 0.7 (0.4, 1.1) | 0.2 (0.1, 0.3) | 2.0 (1.0, 3.3) |
| Use of biomass fuels | 1.1 (0.5, 1.8) | 0.3 (0.1, 0.5) | 3.1 (1.4, 5.3) |

**Fig 1: Stunting cases among 2-year olds attributable to individual risk factors**

Sri Lanka

**Region: East Asia/Pacific; Sub-region: Asia, Southeast**

**Stunting prevalence among children age 2: 19.7%**

**Stunting cases among children age 2: 69,186**

**Population of children age 2: 350,679**

This country profile provides results for 2011 from the analysis presented in *Risk factors for childhood stunting in 137 developing countries: a comparative risk assessment analysis at global, regional, and country levels.*

**Table 1: Population attributable fraction (PAF in %), attributable stunting prevalence (percentage points) and number of stunting cases among 2-year olds attributable to individual risks and risk factor clusters (95% confidence intervals presented in parentheses)**

| **Description** | **PAF** | **Attributable stunting prevalence** | **Attributable stunting cases (thousands)** |
| --- | --- | --- | --- |
| **Maternal nutrition and infection** | **13.6 (12.2, 15.1)** | **2.7 (2.2, 3.2)** | **9.4 (7.7, 11.4)** |
| Maternal short stature | 10.9 (10.6, 11.2) | 2.2 (1.8, 2.5) | 7.5 (6.2, 8.9) |
| Maternal underweight | 1.6 (0.8, 2.6) | 0.3 (0.2, 0.5) | 1.1 (0.6, 1.8) |
| Maternal malaria | 0.0 (0.0, 0.0) | 0.0 (0.0, 0.0) | 0.0 (0.0, 0.0) |
| Maternal anemia | 1.4 (0.4, 2.8) | 0.3 (0.1, 0.5) | 1.0 (0.2, 1.9) |
| **Teenage motherhood and short birth intervals** | **1.3 (1.2, 1.4)** | **0.3 (0.2, 0.3)** | **0.9 (0.7, 1.0)** |
| Teenage motherhood | 0.5 (0.4, 0.5) | 0.1 (0.1, 0.1) | 0.3 (0.3, 0.4) |
| Short birth intervals | 0.8 (0.8, 0.9) | 0.2 (0.1, 0.2) | 0.6 (0.5, 0.7) |
| **Fetal growth restriction and preterm birth** | **29.6 (24.6, 34.1)** | **5.8 (4.5, 7.2)** | **20.5 (15.9, 25.3)** |
| Preterm, small-for-gestational age | 5.8 (2.3, 10.0) | 1.1 (0.4, 2.0) | 4.0 (1.5, 7.2) |
| Preterm, appropriate-for-gestational age | 5.2 (2.3, 8.5) | 1.0 (0.4, 1.7) | 3.6 (1.5, 5.9) |
| Term, small-for-gestational age | 21.1 (16.2, 26.0) | 4.2 (3.0, 5.4) | 14.6 (10.6, 18.9) |
| Low birth weight | 20.5 (17.3, 23.7) | 4.0 (3.2, 5.0) | 14.2 (11.2, 17.5) |
| **Child nutrition and infection** | **10.2 (3.8, 18.0)** | **2.0 (0.8, 3.6)** | **7.1 (2.7, 12.6)** |
| Childhood zinc deficiency | 2.6 (1.0, 5.1) | 0.5 (0.2, 1.0) | 1.8 (0.7, 3.5) |
| Childhood diarrhea | 9.0 (3.0, 16.7) | 1.8 (0.6, 3.3) | 6.2 (2.0, 11.7) |
| Non-exclusive breastfeeding | 0.7 (-1.1, 2.5) | 0.1 (-0.2, 0.5) | 0.5 (-0.8, 1.7) |
| Discontinued breastfeeding | 0.5 (0.0, 1.3) | 0.1 (0.0, 0.3) | 0.3 (0.0, 0.9) |
| HIV infection without HAART before 2 years | No data | No data | No data |
| **Water, sanitation and biomass fuel use** | **9.2 (6.7, 11.6)** | **1.8 (1.3, 2.4)** | **6.4 (4.5, 8.4)** |
| Unimproved sanitation | 3.9 (1.8, 5.9) | 0.8 (0.4, 1.2) | 2.7 (1.2, 4.2) |
| Unimproved water | 0.9 (0.5, 1.4) | 0.2 (0.1, 0.3) | 0.6 (0.3, 1.0) |
| Use of biomass fuels | 4.7 (3.2, 6.3) | 0.9 (0.6, 1.3) | 3.2 (2.1, 4.6) |

**Fig 1: Stunting cases among 2-year olds attributable to individual risk factors**

St. Lucia

**Region: Latin America/Caribbean; Sub-region: Caribbean**

**Stunting prevalence among children age 2: 21.0%**

**Stunting cases among children age 2: 604**

**Population of children age 2: 2,875**

This country profile provides results for 2011 from the analysis presented in *Risk factors for childhood stunting in 137 developing countries: a comparative risk assessment analysis at global, regional, and country levels.*

**Table 1: Population attributable fraction (PAF in %), attributable stunting prevalence (percentage points) and number of stunting cases among 2-year olds attributable to individual risks and risk factor clusters (95% confidence intervals presented in parentheses)**

| **Description** | **PAF** | **Attributable stunting prevalence** | **Attributable stunting cases (thousands)** |
| --- | --- | --- | --- |
| **Maternal nutrition and infection** | **5.0 (3.8, 6.4)** | **1.0 (0.4, 1.8)** | **0.0 (0.0, 0.1)** |
| Maternal short stature | 3.3 (3.0, 3.5) | 0.7 (0.3, 1.1) | 0.0 (0.0, 0.0) |
| Maternal underweight | 0.4 (0.1, 0.8) | 0.1 (0.0, 0.2) | 0.0 (0.0, 0.0) |
| Maternal malaria | 0.0 (0.0, 0.0) | 0.0 (0.0, 0.0) | 0.0 (0.0, 0.0) |
| Maternal anemia | 1.3 (0.3, 2.7) | 0.3 (0.1, 0.7) | 0.0 (0.0, 0.0) |
| **Teenage motherhood and short birth intervals** | **2.3 (2.2, 2.4)** | **0.5 (0.2, 0.8)** | **0.0 (0.0, 0.0)** |
| Teenage motherhood | 1.3 (1.3, 1.4) | 0.3 (0.1, 0.4) | 0.0 (0.0, 0.0) |
| Short birth intervals | 0.9 (0.9, 1.0) | 0.2 (0.1, 0.3) | 0.0 (0.0, 0.0) |
| **Fetal growth restriction and preterm birth** | **23.2 (17.8, 28.1)** | **4.9 (1.7, 8.2)** | **0.1 (0.1, 0.2)** |
| Preterm, small-for-gestational age | 5.3 (1.0, 10.4) | 1.1 (0.2, 2.6) | 0.0 (0.0, 0.1) |
| Preterm, appropriate-for-gestational age | 5.4 (0.8, 9.9) | 1.1 (0.1, 2.6) | 0.0 (0.0, 0.1) |
| Term, small-for-gestational age | 14.3 (10.3, 18.7) | 3.0 (1.1, 5.2) | 0.1 (0.0, 0.1) |
| Low birth weight | 16.3 (13.6, 19.0) | 3.4 (1.3, 5.6) | 0.1 (0.0, 0.2) |
| **Child nutrition and infection** | **16.9 (7.4, 26.9)** | **3.5 (1.0, 6.9)** | **0.1 (0.0, 0.2)** |
| Childhood zinc deficiency | 0.4 (0.1, 1.0) | 0.1 (0.0, 0.2) | 0.0 (0.0, 0.0) |
| Childhood diarrhea | 16.7 (7.2, 26.7) | 3.5 (1.0, 6.9) | 0.1 (0.0, 0.2) |
| Non-exclusive breastfeeding | 3.0 (0.4, 6.4) | 0.6 (0.1, 1.5) | 0.0 (0.0, 0.0) |
| Discontinued breastfeeding | 2.5 (0.4, 5.5) | 0.5 (0.1, 1.3) | 0.0 (0.0, 0.0) |
| HIV infection without HAART before 2 years | No data | No data | No data |
| **Water, sanitation and biomass fuel use** | **10.1 (6.7, 13.4)** | **2.1 (0.8, 3.9)** | **0.1 (0.0, 0.1)** |
| Unimproved sanitation | 9.7 (6.4, 12.9) | 2.0 (0.7, 3.7) | 0.1 (0.0, 0.1) |
| Unimproved water | 0.4 (0.1, 0.8) | 0.1 (0.0, 0.2) | 0.0 (0.0, 0.0) |
| Use of biomass fuels | 0.1 (-0.5, 0.6) | 0.0 (-0.1, 0.1) | 0.0 (0.0, 0.0) |

**Fig 1: Stunting cases among 2-year olds attributable to individual risk factors**

St. Vincent

**Region: Latin America/Caribbean; Sub-region: Caribbean**

**Stunting prevalence among children age 2: 18.7%**

**Stunting cases among children age 2: 348**

**Population of children age 2: 1,864**

This country profile provides results for 2011 from the analysis presented in *Risk factors for childhood stunting in 137 developing countries: a comparative risk assessment analysis at global, regional, and country levels.*

**Table 1: Population attributable fraction (PAF in %), attributable stunting prevalence (percentage points) and number of stunting cases among 2-year olds attributable to individual risks and risk factor clusters (95% confidence intervals presented in parentheses)**

| **Description** | **PAF** | **Attributable stunting prevalence** | **Attributable stunting cases (thousands)** |
| --- | --- | --- | --- |
| **Maternal nutrition and infection** | **9.8 (8.6, 11.3)** | **1.8 (0.6, 3.1)** | **0.0 (0.0, 0.1)** |
| Maternal short stature | 8.5 (7.5, 9.3) | 1.6 (0.6, 2.6) | 0.0 (0.0, 0.0) |
| Maternal underweight | 0.4 (0.1, 0.8) | 0.1 (0.0, 0.2) | 0.0 (0.0, 0.0) |
| Maternal malaria | 0.0 (0.0, 0.0) | 0.0 (0.0, 0.0) | 0.0 (0.0, 0.0) |
| Maternal anemia | 1.1 (0.2, 2.2) | 0.2 (0.0, 0.5) | 0.0 (0.0, 0.0) |
| **Teenage motherhood and short birth intervals** | **2.3 (2.2, 2.4)** | **0.4 (0.1, 0.7)** | **0.0 (0.0, 0.0)** |
| Teenage motherhood | 1.3 (1.3, 1.4) | 0.3 (0.1, 0.4) | 0.0 (0.0, 0.0) |
| Short birth intervals | 0.9 (0.9, 1.0) | 0.2 (0.1, 0.3) | 0.0 (0.0, 0.0) |
| **Fetal growth restriction and preterm birth** | **22.2 (16.0, 27.7)** | **4.1 (1.4, 7.0)** | **0.1 (0.0, 0.1)** |
| Preterm, small-for-gestational age | 6.0 (0.9, 11.3) | 1.1 (0.1, 2.6) | 0.0 (0.0, 0.0) |
| Preterm, appropriate-for-gestational age | 5.9 (0.9, 11.1) | 1.1 (0.1, 2.5) | 0.0 (0.0, 0.0) |
| Term, small-for-gestational age | 12.1 (8.2, 16.1) | 2.2 (0.8, 4.0) | 0.0 (0.0, 0.1) |
| Low birth weight | 13.2 (11.0, 15.5) | 2.5 (0.8, 4.2) | 0.0 (0.0, 0.1) |
| **Child nutrition and infection** | **18.0 (7.9, 28.0)** | **3.4 (0.9, 6.9)** | **0.1 (0.0, 0.1)** |
| Childhood zinc deficiency | 0.9 (0.3, 2.8) | 0.2 (0.0, 0.6) | 0.0 (0.0, 0.0) |
| Childhood diarrhea | 17.6 (7.3, 27.5) | 3.3 (0.9, 6.7) | 0.1 (0.0, 0.1) |
| Non-exclusive breastfeeding | 3.0 (0.1, 7.0) | 0.6 (0.0, 1.6) | 0.0 (0.0, 0.0) |
| HIV infection without HAART before 2 years | No data | No data | No data |
| Discontinued breastfeeding | 2.6 (0.4, 6.1) | 0.5 (0.0, 1.3) | 0.0 (0.0, 0.0) |
| **Water, sanitation and biomass fuel use** | **8.1 (5.6, 10.7)** | **1.5 (0.5, 2.7)** | **0.0 (0.0, 0.1)** |
| Unimproved sanitation | 7.8 (5.5, 10.2) | 1.5 (0.5, 2.5) | 0.0 (0.0, 0.0) |
| Unimproved water | 0.3 (0.1, 0.6) | 0.1 (0.0, 0.1) | 0.0 (0.0, 0.0) |
| Use of biomass fuels | 0.0 (-1.1, 1.0) | 0.0 (-0.2, 0.2) | 0.0 (0.0, 0.0) |

**Fig 1: Stunting cases among 2-year olds attributable to individual risk factors**

State of Palestine

**Region: North Africa/Middle East; Sub-region: North Africa / Middle East**

**Stunting prevalence among children age 2: 14.2%**

**Stunting cases among children age 2: 19,476**

**Population of children age 2: 137,319**

This country profile provides results for 2011 from the analysis presented in *Risk factors for childhood stunting in 137 developing countries: a comparative risk assessment analysis at global, regional, and country levels.*

**Table 1: Population attributable fraction (PAF in %), attributable stunting prevalence (percentage points) and number of stunting cases among 2-year olds attributable to individual risks and risk factor clusters (95% confidence intervals presented in parentheses)**

| **Description** | **PAF** | **Attributable stunting prevalence** | **Attributable stunting cases (thousands)** |
| --- | --- | --- | --- |
| **Maternal nutrition and infection** | **6.7 (5.9, 7.8)** | **0.9 (0.6, 1.3)** | **1.3 (0.8, 1.8)** |
| Maternal short stature | 5.6 (5.3, 5.8) | 0.8 (0.5, 1.1) | 1.1 (0.7, 1.5) |
| Maternal underweight | 0.2 (0.0, 0.5) | 0.0 (0.0, 0.1) | 0.0 (0.0, 0.1) |
| Maternal malaria | 0.0 (0.0, 0.0) | 0.0 (0.0, 0.0) | 0.0 (0.0, 0.0) |
| Maternal anemia | 1.0 (0.2, 2.0) | 0.1 (0.0, 0.3) | 0.2 (0.0, 0.4) |
| **Teenage motherhood and short birth intervals** | **1.8 (1.7, 1.9)** | **0.2 (0.2, 0.3)** | **0.3 (0.2, 0.5)** |
| Teenage motherhood | 0.5 (0.5, 0.6) | 0.1 (0.0, 0.1) | 0.1 (0.1, 0.1) |
| Short birth intervals | 1.2 (1.1, 1.3) | 0.2 (0.1, 0.2) | 0.2 (0.2, 0.3) |
| **Fetal growth restriction and preterm birth** | **23.7 (21.6, 25.9)** | **3.4 (2.1, 4.7)** | **4.6 (2.9, 6.5)** |
| Preterm, small-for-gestational age | 5.5 (3.7, 7.8) | 0.8 (0.4, 1.3) | 1.1 (0.6, 1.7) |
| Preterm, appropriate-for-gestational age | 5.5 (4.0, 7.2) | 0.8 (0.4, 1.2) | 1.1 (0.6, 1.6) |
| Term, small-for-gestational age | 14.5 (12.4, 16.8) | 2.1 (1.3, 2.9) | 2.8 (1.7, 4.0) |
| Low birth weight | 13.1 (10.9, 15.4) | 1.9 (1.1, 2.7) | 2.6 (1.5, 3.7) |
| **Child nutrition and infection** | **19.1 (8.3, 30.6)** | **2.7 (1.0, 4.9)** | **3.7 (1.4, 6.7)** |
| Childhood zinc deficiency | 3.4 (0.6, 14.1) | 0.5 (0.1, 2.0) | 0.7 (0.1, 2.7) |
| Childhood diarrhea | 17.6 (7.3, 27.9) | 2.5 (0.9, 4.4) | 3.4 (1.3, 6.1) |
| Non-exclusive breastfeeding | 2.5 (-0.5, 6.1) | 0.4 (-0.1, 0.9) | 0.5 (-0.1, 1.3) |
| Discontinued breastfeeding | 1.6 (0.2, 3.9) | 0.2 (0.0, 0.6) | 0.3 (0.0, 0.8) |
| HIV infection without HAART before 2 years | No data | No data | No data |
| **Water, sanitation and biomass fuel use** | **3.3 (2.8, 3.7)** | **0.5 (0.3, 0.7)** | **0.6 (0.4, 0.9)** |
| Unimproved sanitation | 2.6 (2.3, 3.0) | 0.4 (0.2, 0.5) | 0.5 (0.3, 0.7) |
| Unimproved water | 0.6 (0.4, 0.8) | 0.1 (0.0, 0.1) | 0.1 (0.1, 0.2) |
| Use of biomass fuels | 0.1 (0.1, 0.2) | 0.0 (0.0, 0.0) | 0.0 (0.0, 0.0) |

**Fig 1: Stunting cases among 2-year olds attributable to individual risk factors**

Sudan

**Region: Sub-Saharan Africa; Sub-region: Sub-Saharan Africa, East**

**Stunting prevalence among children age 2: 40.5%**

**Stunting cases among children age 2: 509,533**

**Population of children age 2: 1,257,928**

This country profile provides results for 2011 from the analysis presented in *Risk factors for childhood stunting in 137 developing countries: a comparative risk assessment analysis at global, regional, and country levels.*

**Table 1: Population attributable fraction (PAF in %), attributable stunting prevalence (percentage points) and number of stunting cases among 2-year olds attributable to individual risks and risk factor clusters (95% confidence intervals presented in parentheses)**

| **Description** | **PAF** | **Attributable stunting prevalence** | **Attributable stunting cases (thousands)** |
| --- | --- | --- | --- |
| **Maternal nutrition and infection** | **15.6 (12.7, 18.9)** | **6.3 (4.8, 8.1)** | **79.6 (60.1, 102.3)** |
| Maternal short stature | 9.9 (9.1, 10.6) | 4.0 (3.2, 4.9) | 50.5 (40.0, 61.2) |
| Maternal underweight | 2.3 (0.9, 4.0) | 0.9 (0.3, 1.7) | 11.7 (4.4, 21.2) |
| Maternal malaria | 0.8 (0.3, 1.4) | 0.3 (0.1, 0.6) | 4.2 (1.5, 7.3) |
| Maternal anemia | 3.3 (0.9, 6.7) | 1.4 (0.4, 2.7) | 17.0 (4.4, 34.1) |
| **Teenage motherhood and short birth intervals** | **1.9 (1.8, 2.0)** | **0.8 (0.6, 0.9)** | **9.7 (7.7, 11.6)** |
| Teenage motherhood | 1.0 (0.9, 1.0) | 0.4 (0.3, 0.5) | 4.8 (3.8, 5.8) |
| Short birth intervals | 1.0 (0.9, 1.0) | 0.4 (0.3, 0.5) | 4.9 (3.8, 5.8) |
| **Fetal growth restriction and preterm birth** | **39.0 (34.6, 43.1)** | **15.8 (12.6, 19.3)** | **198.7 (158.5, 243)** |
| Preterm, small-for-gestational age | 4.4 (2.0, 7.2) | 1.8 (0.8, 3.0) | 22.2 (10.3, 37.3) |
| Preterm, appropriate-for-gestational age | 6.0 (3.1, 9.3) | 2.4 (1.2, 3.9) | 30.3 (15.7, 49.4) |
| Term, small-for-gestational age | 32.2 (26.8, 37.1) | 13.0 (10.2, 16.5) | 163.8 (127.8, 207.1) |
| Low birth weight | 37.1 (32.4, 41.6) | 15.0 (11.7, 18.1) | 189 (147.1, 228) |
| **Child nutrition and infection** | **15.1 (5.8, 27.1)** | **6.1 (2.3, 10.9)** | **76.8 (28.6, 137.4)** |
| Childhood zinc deficiency | 0.3 (0.1, 0.6) | 0.1 (0.1, 0.3) | 1.8 (0.7, 3.4) |
| Childhood diarrhea | 14.9 (5.7, 26.9) | 6.0 (2.2, 10.8) | 76.1 (27.7, 136.4) |
| Non-exclusive breastfeeding | 2.2 (-0.2, 5.6) | 0.9 (-0.1, 2.3) | 11.1 (-1.1, 29.0) |
| HIV infection without HAART before 2 years | No data | No data | No data |
| Discontinued breastfeeding | 1.4 (0.2, 3.6) | 0.6 (0.1, 1.5) | 7.0 (0.8, 18.8) |
| **Water, sanitation and biomass fuel use** | **30.1 (26.7, 33.7)** | **12.2 (9.6, 15.0)** | **153.5 (120.5, 188.4)** |
| Unimproved sanitation | 21.5 (18.7, 24.3) | 8.7 (6.8, 10.7) | 109.4 (85.2, 134.8) |
| Unimproved water | 3.0 (1.4, 5.0) | 1.2 (0.6, 2.1) | 15.1 (7.0, 26.1) |
| Use of biomass fuels | 8.3 (5.7, 11.0) | 3.4 (2.2, 4.8) | 42.3 (28.0, 59.9) |

**Fig 1: Stunting cases among 2-year olds attributable to individual risk factors**

Suriname

**Region: Latin America/Caribbean; Sub-region: Caribbean**

**Stunting prevalence among children age 2: 12.1%**

**Stunting cases among children age 2: 1,209**

**Population of children age 2: 9,967**

This country profile provides results for 2011 from the analysis presented in *Risk factors for childhood stunting in 137 developing countries: a comparative risk assessment analysis at global, regional, and country levels.*

**Table 1: Population attributable fraction (PAF in %), attributable stunting prevalence (percentage points) and number of stunting cases among 2-year olds attributable to individual risks and risk factor clusters (95% confidence intervals presented in parentheses)**

| **Description** | **PAF** | **Attributable stunting prevalence** | **Attributable stunting cases (thousands)** |
| --- | --- | --- | --- |
| **Maternal nutrition and infection** | **10.4 (8.8, 12.1)** | **1.3 (0.8, 1.7)** | **0.1 (0.1, 0.2)** |
| Maternal short stature | 8.6 (7.7, 9.4) | 1.0 (0.7, 1.4) | 0.1 (0.1, 0.1) |
| Maternal underweight | 0.5 (0.0, 1.1) | 0.1 (0.0, 0.1) | 0.0 (0.0, 0.0) |
| Maternal malaria | 0.0 (0.0, 0.0) | 0.0 (0.0, 0.0) | 0.0 (0.0, 0.0) |
| Maternal anemia | 1.4 (0.3, 2.9) | 0.2 (0.0, 0.4) | 0.0 (0.0, 0.0) |
| **Teenage motherhood and short birth intervals** | **2.3 (2.2, 2.4)** | **0.3 (0.2, 0.4)** | **0.0 (0.0, 0.0)** |
| Teenage motherhood | 1.3 (1.3, 1.4) | 0.2 (0.1, 0.2) | 0.0 (0.0, 0.0) |
| Short birth intervals | 0.9 (0.9, 1.0) | 0.1 (0.1, 0.2) | 0.0 (0.0, 0.0) |
| **Fetal growth restriction and preterm birth** | **24.3 (20.0, 28.3)** | **2.9 (1.9, 4.2)** | **0.3 (0.2, 0.4)** |
| Preterm, small-for-gestational age | 5.0 (2.1, 8.4) | 0.6 (0.2, 1.1) | 0.1 (0.0, 0.1) |
| Preterm, appropriate-for-gestational age | 5.0 (2.2, 8.0) | 0.6 (0.2, 1.0) | 0.1 (0.0, 0.1) |
| Term, small-for-gestational age | 16.0 (12.0, 20.5) | 1.9 (1.2, 2.9) | 0.2 (0.1, 0.3) |
| Low birth weight | 17.3 (14.5, 20.2) | 2.1 (1.3, 3.0) | 0.2 (0.1, 0.3) |
| **Child nutrition and infection** | **19.2 (8.4, 30.3)** | **2.3 (0.9, 4.1)** | **0.2 (0.1, 0.4)** |
| Childhood zinc deficiency | 1.6 (0.5, 4.1) | 0.2 (0.1, 0.5) | 0.0 (0.0, 0.1) |
| Childhood diarrhea | 18.6 (7.8, 29.2) | 2.3 (0.9, 4.0) | 0.2 (0.1, 0.4) |
| Non-exclusive breastfeeding | 4.2 (1.3, 8.2) | 0.5 (0.1, 1.1) | 0.1 (0.0, 0.1) |
| Discontinued breastfeeding | 2.8 (0.5, 6.3) | 0.3 (0.1, 0.8) | 0.0 (0.0, 0.1) |
| HIV infection without HAART before 2 years | No data | No data | No data |
| **Water, sanitation and biomass fuel use** | **6.4 (4.9, 8.0)** | **0.8 (0.5, 1.1)** | **0.1 (0.0, 0.1)** |
| Unimproved sanitation | 5.2 (3.8, 6.7) | 0.6 (0.4, 0.9) | 0.1 (0.0, 0.1) |
| Unimproved water | 0.5 (0.2, 0.9) | 0.1 (0.0, 0.1) | 0.0 (0.0, 0.0) |
| Use of biomass fuels | 0.7 (0.3, 1.3) | 0.1 (0.0, 0.2) | 0.0 (0.0, 0.0) |

**Fig 1: Stunting cases among 2-year olds attributable to individual risk factors**

Swaziland

**Region: Sub-Saharan Africa; Sub-region: Sub-Saharan Africa, Southern**

**Stunting prevalence among children age 2: 38.9%**

**Stunting cases among children age 2: 14,330**

**Population of children age 2: 36,792**

This country profile provides results for 2011 from the analysis presented in *Risk factors for childhood stunting in 137 developing countries: a comparative risk assessment analysis at global, regional, and country levels.*

**Table 1: Population attributable fraction (PAF in %), attributable stunting prevalence (percentage points) and number of stunting cases among 2-year olds attributable to individual risks and risk factor clusters (95% confidence intervals presented in parentheses)**

| **Description** | **PAF** | **Attributable stunting prevalence** | **Attributable stunting cases (thousands)** |
| --- | --- | --- | --- |
| **Maternal nutrition and infection** | **7.1 (6.1, 8.3)** | **2.8 (2.2, 3.4)** | **1.0 (0.8, 1.3)** |
| Maternal short stature | 5.3 (5.1, 5.5) | 2.1 (1.7, 2.4) | 0.8 (0.6, 0.9) |
| Maternal underweight | 0.6 (0.2, 1.2) | 0.2 (0.1, 0.5) | 0.1 (0.0, 0.2) |
| Maternal malaria | 0.1 (0.0, 0.1) | 0.0 (0.0, 0.0) | 0.0 (0.0, 0.0) |
| Maternal anemia | 1.2 (0.3, 2.4) | 0.5 (0.1, 0.9) | 0.2 (0.0, 0.3) |
| **Teenage motherhood and short birth intervals** | **2.1 (2.0, 2.3)** | **0.8 (0.7, 1.0)** | **0.3 (0.2, 0.4)** |
| Teenage motherhood | 1.5 (1.4, 1.7) | 0.6 (0.5, 0.7) | 0.2 (0.2, 0.3) |
| Short birth intervals | 0.6 (0.5, 0.7) | 0.2 (0.2, 0.3) | 0.1 (0.1, 0.1) |
| **Fetal growth restriction and preterm birth** | **27.4 (21.7, 32.2)** | **10.7 (8.0, 13.4)** | **3.9 (2.9, 4.9)** |
| Preterm, small-for-gestational age | 5.4 (0.7, 10.9) | 2.1 (0.3, 4.3) | 0.8 (0.1, 1.6) |
| Preterm, appropriate-for-gestational age | 7.3 (1.2, 13.5) | 2.9 (0.5, 5.4) | 1.1 (0.2, 2.0) |
| Term, small-for-gestational age | 17.1 (13.6, 20.7) | 6.6 (5.1, 8.5) | 2.4 (1.9, 3.1) |
| Low birth weight | 15.0 (12.5, 17.6) | 5.9 (4.6, 7.3) | 2.2 (1.7, 2.7) |
| **Child nutrition and infection** | **14.2 (5.2, 25.1)** | **5.5 (2.0, 9.8)** | **2.0 (0.7, 3.6)** |
| Childhood zinc deficiency | 0.7 (0.3, 1.3) | 0.3 (0.1, 0.5) | 0.1 (0.0, 0.2) |
| Childhood diarrhea | 13.9 (4.8, 24.9) | 5.4 (1.9, 9.6) | 2.0 (0.7, 3.5) |
| Non-exclusive breastfeeding | 2.0 (-0.3, 5.4) | 0.8 (-0.1, 2.1) | 0.3 (0.0, 0.8) |
| HIV infection without HAART before 2 years | 1.9 (1.0, 3.6) | 0.7 (0.4, 1.4) | 0.3 (0.1, 0.5) |
| Discontinued breastfeeding | 1.1 (0.1, 2.9) | 0.4 (0.0, 1.1) | 0.2 (0.0, 0.4) |
| **Water, sanitation and biomass fuel use** | **17.5 (15.1, 19.9)** | **6.8 (5.6, 8.4)** | **2.5 (2.1, 3.1)** |
| Unimproved sanitation | 12.9 (10.7, 15.2) | 5.0 (3.9, 6.3) | 1.8 (1.4, 2.3) |
| Unimproved water | 2.5 (1.5, 3.6) | 1.0 (0.6, 1.4) | 0.4 (0.2, 0.5) |
| Use of biomass fuels | 3.0 (2.0, 4.1) | 1.2 (0.8, 1.7) | 0.4 (0.3, 0.6) |

**Fig 1: Stunting cases among 2-year olds attributable to individual risk factors**

Syrian Arab Republic

**Region: North Africa/Middle East; Sub-region: North Africa / Middle East**

**Stunting prevalence among children age 2: 30.9%**

**Stunting cases among children age 2: 152,027**

**Population of children age 2: 492,092**

This country profile provides results for 2011 from the analysis presented in *Risk factors for childhood stunting in 137 developing countries: a comparative risk assessment analysis at global, regional, and country levels.*

**Table 1: Population attributable fraction (PAF in %), attributable stunting prevalence (percentage points) and number of stunting cases among 2-year olds attributable to individual risks and risk factor clusters (95% confidence intervals presented in parentheses)**

| **Description** | **PAF** | **Attributable stunting prevalence** | **Attributable stunting cases (thousands)** |
| --- | --- | --- | --- |
| **Maternal nutrition and infection** | **10.6 (9.3, 12.1)** | **3.3 (2.4, 4.2)** | **16.2 (11.9, 20.9)** |
| Maternal short stature | 9.2 (8.3, 9.9) | 2.8 (2.1, 3.6) | 14.0 (10.5, 17.6) |
| Maternal underweight | 0.4 (0.1, 0.8) | 0.1 (0.0, 0.3) | 0.6 (0.1, 1.3) |
| Maternal malaria | 0.0 (0.0, 0.0) | 0.0 (0.0, 0.0) | 0.0 (0.0, 0.0) |
| Maternal anemia | 1.2 (0.2, 2.5) | 0.4 (0.1, 0.8) | 1.8 (0.3, 3.9) |
| **Teenage motherhood and short birth intervals** | **1.8 (1.7, 1.9)** | **0.5 (0.4, 0.7)** | **2.7 (2.0, 3.4)** |
| Teenage motherhood | 0.5 (0.5, 0.6) | 0.2 (0.1, 0.2) | 0.8 (0.6, 1.0) |
| Short birth intervals | 1.2 (1.1, 1.3) | 0.4 (0.3, 0.5) | 1.9 (1.4, 2.3) |
| **Fetal growth restriction and preterm birth** | **26.3 (20.7, 31.7)** | **8.1 (5.8, 11.1)** | **40.1 (28.3, 54.4)** |
| Preterm, small-for-gestational age | 5.9 (0.5, 11.8) | 1.8 (0.1, 3.8) | 9.0 (0.7, 18.8) |
| Preterm, appropriate-for-gestational age | 5.8 (1.3, 10.4) | 1.8 (0.4, 3.4) | 8.8 (1.9, 17.0) |
| Term, small-for-gestational age | 16.9 (11.7, 22.2) | 5.2 (3.2, 7.5) | 25.7 (16.0, 36.7) |
| Low birth weight | 15.3 (12.8, 17.9) | 4.7 (3.4, 6.2) | 23.3 (16.6, 30.5) |
| **Child nutrition and infection** | **11.5 (3.7, 21.3)** | **3.6 (1.1, 6.9)** | **17.5 (5.4, 34.0)** |
| Childhood zinc deficiency | 1.1 (0.3, 2.8) | 0.4 (0.1, 1.0) | 1.7 (0.5, 4.8) |
| Childhood diarrhea | 11.0 (3.3, 20.7) | 3.4 (1.0, 6.7) | 16.7 (4.8, 32.9) |
| Non-exclusive breastfeeding | 1.5 (-0.4, 4.1) | 0.5 (-0.1, 1.3) | 2.3 (-0.7, 6.6) |
| HIV infection without HAART before 2 years | No data | No data | No data |
| Discontinued breastfeeding | 1.1 (0.1, 2.9) | 0.3 (0.0, 0.9) | 1.7 (0.2, 4.6) |
| **Water, sanitation and biomass fuel use** | **2.9 (1.9, 3.9)** | **0.9 (0.5, 1.3)** | **4.3 (2.7, 6.4)** |
| Unimproved sanitation | 2.2 (1.3, 3.1) | 0.7 (0.4, 1.0) | 3.3 (1.9, 5.2) |
| Unimproved water | 0.7 (0.3, 1.1) | 0.2 (0.1, 0.4) | 1.1 (0.5, 1.8) |
| Use of biomass fuels | 0.0 (-0.2, 0.2) | 0.0 (0.0, 0.0) | 0.0 (-0.2, 0.2) |

**Fig 1: Stunting cases among 2-year olds attributable to individual risk factors**

Taiwan

**Region: East Asia/Pacific; Sub-region: Asia, East**

**Stunting prevalence among children age 2: 17.2%**

**Stunting cases among children age 2: 41,624**

**Population of children age 2: 241,657**

This country profile provides results for 2011 from the analysis presented in *Risk factors for childhood stunting in 137 developing countries: a comparative risk assessment analysis at global, regional, and country levels.*

**Table 1: Population attributable fraction (PAF in %), attributable stunting prevalence (percentage points) and number of stunting cases among 2-year olds attributable to individual risks and risk factor clusters (95% confidence intervals presented in parentheses)**

| **Description** | **PAF** | **Attributable stunting prevalence** | **Attributable stunting cases (thousands)** |
| --- | --- | --- | --- |
| **Maternal nutrition and infection** | **8.9 (8.2, 9.5)** | **1.5 (0.5, 2.6)** | **3.7 (1.2, 6.3)** |
| Maternal short stature | 7.9 (7.5, 8.3) | 1.4 (0.5, 2.3) | 3.3 (1.1, 5.6) |
| Maternal underweight | 0.4 (0.2, 0.7) | 0.1 (0.0, 0.2) | 0.2 (0.0, 0.4) |
| Maternal malaria | 0.0 (0.0, 0.0) | 0.0 (0.0, 0.0) | 0.0 (0.0, 0.0) |
| Maternal anemia | 0.6 (0.1, 1.2) | 0.1 (0.0, 0.3) | 0.2 (0.0, 0.6) |
| **Teenage motherhood and short birth intervals** | **1.3 (1.2, 1.4)** | **0.2 (0.1, 0.4)** | **0.5 (0.2, 0.9)** |
| Teenage motherhood | 0.5 (0.4, 0.5) | 0.1 (0.0, 0.1) | 0.2 (0.1, 0.3) |
| Short birth intervals | 0.8 (0.8, 0.9) | 0.1 (0.0, 0.3) | 0.4 (0.1, 0.6) |
| **Fetal growth restriction and preterm birth** | **19.7 (15.6, 23.7)** | **3.4 (1.1, 6.0)** | **8.2 (2.8, 14.5)** |
| Preterm, small-for-gestational age | 5.7 (2.7, 9.4) | 1.0 (0.3, 2.0) | 2.3 (0.7, 4.9) |
| Preterm, appropriate-for-gestational age | 5.1 (2.5, 8.1) | 0.9 (0.2, 1.7) | 2.1 (0.6, 4.2) |
| Term, small-for-gestational age | 10.4 (7.1, 13.8) | 1.8 (0.6, 3.3) | 4.3 (1.4, 7.9) |
| Low birth weight | 8.1 (6.6, 9.5) | 1.4 (0.5, 2.4) | 3.4 (1.1, 5.8) |
| **Child nutrition and infection** | **11.3 (4.7, 19.5)** | **2.0 (0.5, 4.2)** | **4.7 (1.2, 10.1)** |
| Childhood zinc deficiency | 2.6 (0.5, 11.4) | 0.4 (0.1, 2.1) | 1.1 (0.1, 5.1) |
| Childhood diarrhea | 10.2 (4.2, 17.3) | 1.8 (0.4, 3.7) | 4.2 (1.1, 8.8) |
| Non-exclusive breastfeeding | 1.8 (0.2, 4.3) | 0.3 (0.0, 0.9) | 0.8 (0.1, 2.1) |
| Discontinued breastfeeding | 1.1 (0.2, 2.5) | 0.2 (0.0, 0.5) | 0.5 (0.0, 1.3) |
| HIV infection without HAART before 2 years | No data | No data | No data |
| **Water, sanitation and biomass fuel use** | **11.8 (8.6, 14.9)** | **2.0 (0.6, 3.6)** | **4.9 (1.5, 8.7)** |
| Unimproved sanitation | 9.9 (6.7, 13.2) | 1.7 (0.5, 3.1) | 4.1 (1.3, 7.5) |
| Unimproved water | 0.3 (0.1, 0.6) | 0.1 (0.0, 0.1) | 0.1 (0.0, 0.3) |
| Use of biomass fuels | 1.7 (1.2, 2.3) | 0.3 (0.1, 0.6) | 0.7 (0.2, 1.3) |

**Fig 1: Stunting cases among 2-year olds attributable to individual risk factors**

Tajikistan

**Region: Central Asia Eastern Europe/Central Asia; Sub-region: Asia, Central**

**Stunting prevalence among children age 2: 43.7%**

**Stunting cases among children age 2: 100,766**

**Population of children age 2: 230,711**

This country profile provides results for 2011 from the analysis presented in *Risk factors for childhood stunting in 137 developing countries: a comparative risk assessment analysis at global, regional, and country levels.*

**Table 1: Population attributable fraction (PAF in %), attributable stunting prevalence (percentage points) and number of stunting cases among 2-year olds attributable to individual risks and risk factor clusters (95% confidence intervals presented in parentheses)**

| **Description** | **PAF** | **Attributable stunting prevalence** | **Attributable stunting cases (thousands)** |
| --- | --- | --- | --- |
| **Maternal nutrition and infection** | **7.3 (6.5, 8.2)** | **3.2 (2.3, 4.1)** | **7.3 (5.4, 9.4)** |
| Maternal short stature | 6.0 (5.8, 6.3) | 2.6 (2.0, 3.3) | 6.1 (4.6, 7.6) |
| Maternal underweight | 0.5 (0.1, 0.8) | 0.2 (0.1, 0.4) | 0.5 (0.1, 0.9) |
| Maternal malaria | 0.0 (0.0, 0.0) | 0.0 (0.0, 0.0) | 0.0 (0.0, 0.0) |
| Maternal anemia | 0.9 (0.2, 1.7) | 0.4 (0.1, 0.8) | 0.9 (0.2, 1.8) |
| **Teenage motherhood and short birth intervals** | **1.6 (1.5, 1.7)** | **0.7 (0.5, 0.9)** | **1.6 (1.2, 2.0)** |
| Teenage motherhood | 0.5 (0.5, 0.6) | 0.2 (0.2, 0.3) | 0.5 (0.4, 0.7) |
| Short birth intervals | 1.0 (1.0, 1.1) | 0.5 (0.3, 0.6) | 1.1 (0.8, 1.3) |
| **Fetal growth restriction and preterm birth** | **25.0 (18.5, 30.8)** | **10.9 (7.2, 14.9)** | **25.2 (16.7, 34.4)** |
| Preterm, small-for-gestational age | 6.3 (1.9, 11.9) | 2.7 (0.7, 5.4) | 6.3 (1.7, 12.4) |
| Preterm, appropriate-for-gestational age | 5.7 (0.8, 10.7) | 2.5 (0.4, 4.9) | 5.7 (0.8, 11.3) |
| Term, small-for-gestational age | 15.1 (9.7, 20.7) | 6.6 (4.0, 9.6) | 15.2 (9.3, 22.3) |
| Low birth weight | 11.3 (9.3, 13.3) | 4.9 (3.6, 6.5) | 11.4 (8.2, 15.0) |
| **Child nutrition and infection** | **19.8 (9.0, 31.1)** | **8.7 (3.6, 14.5)** | **20.0 (8.3, 33.5)** |
| Childhood zinc deficiency | 0.7 (0.3, 1.3) | 0.3 (0.1, 0.6) | 0.7 (0.3, 1.4) |
| Childhood diarrhea | 19.6 (8.6, 30.7) | 8.5 (3.5, 14.4) | 19.7 (8.0, 33.3) |
| Non-exclusive breastfeeding | 2.0 (-1.6, 6.0) | 0.9 (-0.7, 2.7) | 2.0 (-1.7, 6.2) |
| Discontinued breastfeeding | 1.5 (0.2, 3.9) | 0.6 (0.1, 1.7) | 1.5 (0.2, 4.0) |
| HIV infection without HAART before 2 years | No data | No data | No data |
| **Water, sanitation and biomass fuel use** | **5.5 (3.6, 7.5)** | **2.4 (1.5, 3.5)** | **5.6 (3.4, 8.2)** |
| Unimproved sanitation | 1.8 (0.6, 3.0) | 0.8 (0.2, 1.4) | 1.8 (0.5, 3.3) |
| Unimproved water | 2.4 (1.3, 3.6) | 1.0 (0.5, 1.6) | 2.4 (1.2, 3.8) |
| Use of biomass fuels | 1.4 (0.3, 2.6) | 0.6 (0.1, 1.2) | 1.5 (0.3, 2.8) |

**Fig 1: Stunting cases among 2-year olds attributable to individual risk factors**

Thailand

**Region: East Asia/Pacific; Sub-region: Asia, Southeast**

**Stunting prevalence among children age 2: 14.6%**

**Stunting cases among children age 2: 114,113**

**Population of children age 2: 782,732**

This country profile provides results for 2011 from the analysis presented in *Risk factors for childhood stunting in 137 developing countries: a comparative risk assessment analysis at global, regional, and country levels.*

**Table 1: Population attributable fraction (PAF in %), attributable stunting prevalence (percentage points) and number of stunting cases among 2-year olds attributable to individual risks and risk factor clusters (95% confidence intervals presented in parentheses)**

| **Description** | **PAF** | **Attributable stunting prevalence** | **Attributable stunting cases (thousands)** |
| --- | --- | --- | --- |
| **Maternal nutrition and infection** | **10.7 (9.5, 12.0)** | **1.6 (1.0, 2.1)** | **12.2 (8.2, 16.4)** |
| Maternal short stature | 8.8 (8.6, 9.0) | 1.3 (0.9, 1.7) | 10.0 (6.8, 13.2) |
| Maternal underweight | 0.9 (0.4, 1.4) | 0.1 (0.1, 0.2) | 1.0 (0.4, 1.7) |
| Maternal malaria | 0.0 (0.0, 0.0) | 0.0 (0.0, 0.0) | 0.0 (0.0, 0.0) |
| Maternal anemia | 1.2 (0.1, 2.6) | 0.2 (0.0, 0.4) | 1.4 (0.1, 3.1) |
| **Teenage motherhood and short birth intervals** | **1.3 (1.2, 1.4)** | **0.2 (0.1, 0.2)** | **1.5 (1.0, 1.9)** |
| Teenage motherhood | 0.5 (0.4, 0.5) | 0.1 (0.0, 0.1) | 0.5 (0.3, 0.7) |
| Short birth intervals | 0.8 (0.8, 0.9) | 0.1 (0.1, 0.2) | 1.0 (0.7, 1.3) |
| **Fetal growth restriction and preterm birth** | **26.8 (22.1, 31.3)** | **3.9 (2.6, 5.4)** | **30.5 (20.0, 42.0)** |
| Preterm, small-for-gestational age | 6.8 (4.1, 10.3) | 1.0 (0.5, 1.7) | 7.7 (4.0, 12.9) |
| Preterm, appropriate-for-gestational age | 6.2 (3.8, 8.9) | 0.9 (0.5, 1.4) | 7.0 (3.8, 11.2) |
| Term, small-for-gestational age | 16.3 (10.9, 21.3) | 2.4 (1.4, 3.5) | 18.5 (11.1, 27.7) |
| Low birth weight | 15.0 (12.5, 17.6) | 2.2 (1.5, 3.0) | 17.2 (11.5, 23.4) |
| **Child nutrition and infection** | **10.1 (3.5, 18.1)** | **1.5 (0.5, 2.8)** | **11.6 (3.7, 22.0)** |
| Childhood zinc deficiency | 2.5 (0.7, 7.0) | 0.4 (0.1, 1.1) | 2.8 (0.7, 8.6) |
| Childhood diarrhea | 9.0 (2.9, 16.4) | 1.3 (0.4, 2.5) | 10.3 (3.1, 19.9) |
| Non-exclusive breastfeeding | 2.0 (0.5, 4.1) | 0.3 (0.1, 0.7) | 2.3 (0.5, 5.1) |
| HIV infection without HAART before 2 years | No data | No data | No data |
| Discontinued breastfeeding | 1.5 (0.2, 3.5) | 0.2 (0.0, 0.5) | 1.7 (0.2, 4.1) |
| **Water, sanitation and biomass fuel use** | **2.8 (1.3, 4.4)** | **0.4 (0.2, 0.7)** | **3.2 (1.5, 5.4)** |
| Unimproved sanitation | 1.1 (0.4, 1.9) | 0.2 (0.1, 0.3) | 1.3 (0.4, 2.3) |
| Unimproved water | 0.4 (0.2, 0.7) | 0.1 (0.0, 0.1) | 0.5 (0.2, 0.9) |
| Use of biomass fuels | 1.3 (0.1, 2.8) | 0.2 (0.0, 0.4) | 1.5 (0.1, 3.3) |

**Fig 1: Stunting cases among 2-year olds attributable to individual risk factors**

Timore Leste

**Region: East Asia/Pacific; Sub-region: Asia, Southeast**

**Stunting prevalence among children age 2: 57.7%**

**Stunting cases among children age 2: 24,388**

**Population of children age 2: 42,288**

This country profile provides results for 2011 from the analysis presented in *Risk factors for childhood stunting in 137 developing countries: a comparative risk assessment analysis at global, regional, and country levels.*

**Table 1: Population attributable fraction (PAF in %), attributable stunting prevalence (percentage points) and number of stunting cases among 2-year olds attributable to individual risks and risk factor clusters (95% confidence intervals presented in parentheses)**

| **Description** | **PAF** | **Attributable stunting prevalence** | **Attributable stunting cases (thousands)** |
| --- | --- | --- | --- |
| **Maternal nutrition and infection** | **15.4 (14.1, 16.9)** | **8.9 (7.2, 10.7)** | **3.8 (3.1, 4.5)** |
| Maternal short stature | 12.6 (12.3, 12.9) | 7.3 (5.9, 8.5) | 3.1 (2.5, 3.6) |
| Maternal underweight | 2.0 (1.0, 3.2) | 1.2 (0.6, 1.9) | 0.5 (0.2, 0.8) |
| Maternal malaria | 0.0 (0.0, 0.0) | 0.0 (0.0, 0.0) | 0.0 (0.0, 0.0) |
| Maternal anemia | 1.3 (0.3, 2.3) | 0.7 (0.2, 1.4) | 0.3 (0.1, 0.6) |
| **Teenage motherhood and short birth intervals** | **1.8 (1.7, 1.9)** | **1.0 (0.8, 1.2)** | **0.4 (0.4, 0.5)** |
| Teenage motherhood | 0.4 (0.4, 0.5) | 0.2 (0.2, 0.3) | 0.1 (0.1, 0.1) |
| Short birth intervals | 1.4 (1.3, 1.5) | 0.8 (0.6, 0.9) | 0.3 (0.3, 0.4) |
| **Fetal growth restriction and preterm birth** | **31.0 (25.5, 35.9)** | **17.9 (13.9, 22.4)** | **7.6 (5.9, 9.5)** |
| Preterm, small-for-gestational age | 6.1 (0.8, 12.0) | 3.5 (0.4, 6.9) | 1.5 (0.2, 2.9) |
| Preterm, appropriate-for-gestational age | 5.7 (0.7, 11.0) | 3.3 (0.4, 6.4) | 1.4 (0.2, 2.7) |
| Term, small-for-gestational age | 22.1 (16.9, 27.5) | 12.7 (9.3, 16.9) | 5.4 (3.9, 7.1) |
| Low birth weight | 18.8 (15.8, 21.8) | 10.8 (8.3, 13.6) | 4.6 (3.5, 5.7) |
| **Child nutrition and infection** | **11.6 (4.4, 19.9)** | **6.7 (2.5, 11.6)** | **2.8 (1.1, 4.9)** |
| Childhood zinc deficiency | 0.5 (0.2, 0.8) | 0.3 (0.1, 0.5) | 0.1 (0.0, 0.2) |
| Childhood diarrhea | 11.3 (4.2, 19.6) | 6.5 (2.4, 11.5) | 2.8 (1.0, 4.9) |
| Non-exclusive breastfeeding | 1.1 (-1.0, 3.6) | 0.7 (-0.6, 2.1) | 0.3 (-0.3, 0.9) |
| Discontinued breastfeeding | 1.2 (0.1, 3.0) | 0.7 (0.1, 1.8) | 0.3 (0.0, 0.8) |
| HIV infection without HAART before 2 years | No data | No data | No data |
| **Water, sanitation and biomass fuel use** | **23.3 (19.9, 26.6)** | **13.4 (10.6, 16.7)** | **5.7 (4.5, 7.1)** |
| Unimproved sanitation | 17.1 (13.9, 20.4) | 9.9 (7.5, 12.6) | 4.2 (3.2, 5.3) |
| Unimproved water | 2.5 (1.5, 3.6) | 1.4 (0.8, 2.1) | 0.6 (0.3, 0.9) |
| Use of biomass fuels | 5.1 (3.7, 6.6) | 2.9 (2.0, 4.1) | 1.2 (0.9, 1.7) |

**Fig 1: Stunting cases among 2-year olds attributable to individual risk factors**

Togo

**Region: Sub-Saharan Africa; Sub-region: Sub-Saharan Africa, West**

**Stunting prevalence among children age 2: 34.6%**

**Stunting cases among children age 2: 82,907**

**Population of children age 2: 239,946**

This country profile provides results for 2011 from the analysis presented in *Risk factors for childhood stunting in 137 developing countries: a comparative risk assessment analysis at global, regional, and country levels.*

**Table 1: Population attributable fraction (PAF in %), attributable stunting prevalence (percentage points) and number of stunting cases among 2-year olds attributable to individual risks and risk factor clusters (95% confidence intervals presented in parentheses)**

| **Description** | **PAF** | **Attributable stunting prevalence** | **Attributable stunting cases (thousands)** |
| --- | --- | --- | --- |
| **Maternal nutrition and infection** | **11.7 (9.2, 14.3)** | **4.0 (3.0, 5.3)** | **9.7 (7.1, 12.7)** |
| Maternal short stature | 5.4 (5.2, 5.6) | 1.9 (1.6, 2.2) | 4.5 (3.7, 5.2) |
| Maternal underweight | 1.2 (0.5, 1.9) | 0.4 (0.2, 0.7) | 1.0 (0.4, 1.7) |
| Maternal malaria | 3.1 (1.2, 4.8) | 1.1 (0.4, 1.7) | 2.5 (1.0, 4.1) |
| Maternal anemia | 2.6 (0.8, 4.5) | 0.9 (0.3, 1.6) | 2.2 (0.6, 4.0) |
| **Teenage motherhood and short birth intervals** | **1.4 (1.3, 1.5)** | **0.5 (0.4, 0.6)** | **1.2 (1.0, 1.4)** |
| Teenage motherhood | 0.7 (0.7, 0.8) | 0.2 (0.2, 0.3) | 0.6 (0.5, 0.7) |
| Short birth intervals | 0.7 (0.6, 0.8) | 0.2 (0.2, 0.3) | 0.6 (0.5, 0.7) |
| **Fetal growth restriction and preterm birth** | **29.3 (24.0, 33.9)** | **10.1 (7.8, 12.6)** | **24.3 (18.6, 30.2)** |
| Preterm, small-for-gestational age | 5.0 (0.5, 9.8) | 1.7 (0.2, 3.4) | 4.2 (0.5, 8.2) |
| Preterm, appropriate-for-gestational age | 6.9 (0.8, 13.0) | 2.4 (0.3, 4.6) | 5.7 (0.6, 11.1) |
| Term, small-for-gestational age | 20.0 (16.3, 24.1) | 6.9 (5.3, 8.9) | 16.6 (12.6, 21.4) |
| Low birth weight | 18.1 (15.2, 21.1) | 6.3 (4.9, 7.9) | 15.0 (11.7, 18.9) |
| **Child nutrition and infection** | **13.7 (5.4, 23.7)** | **4.7 (1.8, 8.3)** | **11.4 (4.4, 19.9)** |
| Childhood zinc deficiency | 1.2 (0.5, 2.1) | 0.4 (0.2, 0.7) | 1.0 (0.4, 1.8) |
| Childhood diarrhea | 13.2 (5.0, 23.2) | 4.6 (1.7, 8.1) | 11.0 (4.1, 19.4) |
| Non-exclusive breastfeeding | 1.5 (-1.1, 4.5) | 0.5 (-0.4, 1.5) | 1.2 (-0.9, 3.7) |
| Discontinued breastfeeding | 0.4 (0.0, 1.1) | 0.1 (0.0, 0.4) | 0.3 (0.0, 1.0) |
| HIV infection without HAART before 2 years | 0.4 (0.2, 0.9) | 0.1 (0.1, 0.3) | 0.3 (0.1, 0.7) |
| **Water, sanitation and biomass fuel use** | **30.2 (27.8, 32.6)** | **10.4 (8.5, 12.5)** | **25.0 (20.5, 29.9)** |
| Unimproved sanitation | 24.1 (21.9, 26.3) | 8.3 (6.8, 10.0) | 20.0 (16.4, 24.0) |
| Unimproved water | 3.2 (1.8, 4.6) | 1.1 (0.6, 1.7) | 2.6 (1.4, 4.1) |
| Use of biomass fuels | 5.0 (3.6, 6.4) | 1.7 (1.2, 2.3) | 4.1 (2.9, 5.6) |

**Fig 1: Stunting cases among 2-year olds attributable to individual risk factors**

Tonga

**Region: East Asia/Pacific; Sub-region: Oceania**

**Stunting prevalence among children age 2: 16.1%**

**Stunting cases among children age 2: 451**

**Population of children age 2: 2,795**

This country profile provides results for 2011 from the analysis presented in *Risk factors for childhood stunting in 137 developing countries: a comparative risk assessment analysis at global, regional, and country levels.*

**Table 1: Population attributable fraction (PAF in %), attributable stunting prevalence (percentage points) and number of stunting cases among 2-year olds attributable to individual risks and risk factor clusters (95% confidence intervals presented in parentheses)**

| **Description** | **PAF** | **Attributable stunting prevalence** | **Attributable stunting cases (thousands)** |
| --- | --- | --- | --- |
| **Maternal nutrition and infection** | **1.8 (1.4, 2.3)** | **0.3 (0.0, 0.6)** | **0.0 (0.0, 0.0)** |
| Maternal short stature | 1.4 (1.3, 1.5) | 0.2 (0.0, 0.4) | 0.0 (0.0, 0.0) |
| Maternal underweight | 0.0 (0.0, 0.1) | 0.0 (0.0, 0.0) | 0.0 (0.0, 0.0) |
| Maternal malaria | 0.0 (0.0, 0.0) | 0.0 (0.0, 0.0) | 0.0 (0.0, 0.0) |
| Maternal anemia | 0.4 (0.0, 0.9) | 0.1 (0.0, 0.2) | 0.0 (0.0, 0.0) |
| **Teenage motherhood and short birth intervals** | **1.3 (1.2, 1.4)** | **0.2 (0.0, 0.4)** | **0.0 (0.0, 0.0)** |
| Teenage motherhood | 0.5 (0.4, 0.5) | 0.1 (0.0, 0.1) | 0.0 (0.0, 0.0) |
| Short birth intervals | 0.8 (0.8, 0.9) | 0.1 (0.0, 0.3) | 0.0 (0.0, 0.0) |
| **Fetal growth restriction and preterm birth** | **15.3 (9.3, 20.7)** | **2.5 (-0.3, 5.5)** | **0.1 (0.0, 0.2)** |
| Preterm, small-for-gestational age | 4.7 (0.8, 9.4) | 0.8 (-0.1, 2.1) | 0.0 (0.0, 0.1) |
| Preterm, appropriate-for-gestational age | 4.4 (0.6, 8.4) | 0.7 (-0.1, 2.0) | 0.0 (0.0, 0.1) |
| Term, small-for-gestational age | 7.0 (2.4, 11.6) | 1.1 (-0.1, 2.9) | 0.0 (0.0, 0.1) |
| Low birth weight | 5.5 (4.5, 6.5) | 0.9 (-0.1, 1.8) | 0.0 (0.0, 0.1) |
| **Child nutrition and infection** | **11.6 (4.6, 20.4)** | **1.9 (-0.2, 4.6)** | **0.1 (0.0, 0.1)** |
| Childhood zinc deficiency | 2.5 (0.4, 12.5) | 0.4 (0.0, 2.1) | 0.0 (0.0, 0.1) |
| Childhood diarrhea | 10.5 (4.1, 17.6) | 1.7 (-0.2, 4.1) | 0.0 (0.0, 0.1) |
| Non-exclusive breastfeeding | 1.2 (-1.1, 3.5) | 0.2 (-0.2, 0.8) | 0.0 (0.0, 0.0) |
| HIV infection without HAART before 2 years | No data | No data | No data |
| Discontinued breastfeeding | 0.9 (0.1, 2.4) | 0.1 (0.0, 0.5) | 0.0 (0.0, 0.0) |
| **Water, sanitation and biomass fuel use** | **6.7 (4.9, 8.6)** | **1.1 (-0.1, 2.3)** | **0.0 (0.0, 0.1)** |
| Unimproved sanitation | 5.9 (4.2, 7.7) | 1.0 (-0.1, 2.0) | 0.0 (0.0, 0.1) |
| Unimproved water | 0.1 (0.0, 0.1) | 0.0 (0.0, 0.0) | 0.0 (0.0, 0.0) |
| Use of biomass fuels | 0.8 (0.3, 1.4) | 0.1 (0.0, 0.3) | 0.0 (0.0, 0.0) |

**Fig 1: Stunting cases among 2-year olds attributable to individual risk factors**

Trinidad and Tobago

**Region: Latin America/Caribbean; Sub-region: Caribbean**

**Stunting prevalence among children age 2: 8.8%**

**Stunting cases among children age 2: 1,768**

**Population of children age 2: 19,974**

This country profile provides results for 2011 from the analysis presented in *Risk factors for childhood stunting in 137 developing countries: a comparative risk assessment analysis at global, regional, and country levels.*

**Table 1: Population attributable fraction (PAF in %), attributable stunting prevalence (percentage points) and number of stunting cases among 2-year olds attributable to individual risks and risk factor clusters (95% confidence intervals presented in parentheses)**

| **Description** | **PAF** | **Attributable stunting prevalence** | **Attributable stunting cases (thousands)** |
| --- | --- | --- | --- |
| **Maternal nutrition and infection** | **5.6 (4.4, 7.1)** | **0.5 (0.0, 1.0)** | **0.1 (0.0, 0.2)** |
| Maternal short stature | 4.0 (3.7, 4.3) | 0.4 (0.0, 0.7) | 0.1 (0.0, 0.1) |
| Maternal underweight | 0.3 (0.0, 0.8) | 0.0 (0.0, 0.1) | 0.0 (0.0, 0.0) |
| Maternal malaria | 0.0 (0.0, 0.0) | 0.0 (0.0, 0.0) | 0.0 (0.0, 0.0) |
| Maternal anemia | 1.3 (0.2, 2.9) | 0.1 (0.0, 0.3) | 0.0 (0.0, 0.1) |
| **Teenage motherhood and short birth intervals** | **2.3 (2.2, 2.4)** | **0.2 (0.0, 0.4)** | **0.0 (0.0, 0.1)** |
| Teenage motherhood | 1.3 (1.3, 1.4) | 0.1 (0.0, 0.2) | 0.0 (0.0, 0.0) |
| Short birth intervals | 0.9 (0.9, 1.0) | 0.1 (0.0, 0.2) | 0.0 (0.0, 0.0) |
| **Fetal growth restriction and preterm birth** | **23.4 (19.3, 27.4)** | **2.1 (0.0, 4.1)** | **0.4 (0.0, 0.8)** |
| Preterm, small-for-gestational age | 4.6 (1.9, 7.8) | 0.4 (0.0, 1.0) | 0.1 (0.0, 0.2) |
| Preterm, appropriate-for-gestational age | 4.5 (2.3, 7.4) | 0.4 (0.0, 0.9) | 0.1 (0.0, 0.2) |
| Term, small-for-gestational age | 15.9 (11.6, 20.1) | 1.4 (0.0, 2.9) | 0.3 (0.0, 0.6) |
| Low birth weight | 16.4 (13.7, 19.1) | 1.5 (0.0, 2.8) | 0.3 (0.0, 0.6) |
| **Child nutrition and infection** | **11.0 (3.6, 34.4)** | **1.0 (0.0, 3.5)** | **0.2 (0.0, 0.7)** |
| Childhood zinc deficiency | 8.6 (1.2, 43.6) | 0.8 (0.0, 4.5) | 0.2 (0.0, 0.9) |
| Childhood diarrhea | 7.2 (2.9, 11.8) | 0.6 (0.0, 1.5) | 0.1 (0.0, 0.3) |
| Non-exclusive breastfeeding | 1.4 (0.3, 3.0) | 0.1 (0.0, 0.4) | 0.0 (0.0, 0.1) |
| HIV infection without HAART before 2 years | No data | No data | No data |
| Discontinued breastfeeding | 1.2 (0.2, 2.7) | 0.1 (0.0, 0.3) | 0.0 (0.0, 0.1) |
| **Water, sanitation and biomass fuel use** | **3.0 (1.9, 4.1)** | **0.3 (0.0, 0.6)** | **0.1 (0.0, 0.1)** |
| Unimproved sanitation | 2.5 (1.5, 3.6) | 0.2 (0.0, 0.5) | 0.0 (0.0, 0.1) |
| Unimproved water | 0.4 (0.1, 0.8) | 0.0 (0.0, 0.1) | 0.0 (0.0, 0.0) |
| Use of biomass fuels | 0.0 (-0.2, 0.2) | 0.0 (0.0, 0.0) | 0.0 (0.0, 0.0) |

**Fig 1: Stunting cases among 2-year olds attributable to individual risk factors**

Tunisia

**Region: North Africa/Middle East; Sub-region: North Africa / Middle East**

**Stunting prevalence among children age 2: 10.0%**

**Stunting cases among children age 2: 18,930**

**Population of children age 2: 188,962**

This country profile provides results for 2011 from the analysis presented in *Risk factors for childhood stunting in 137 developing countries: a comparative risk assessment analysis at global, regional, and country levels.*

**Table 1: Population attributable fraction (PAF in %), attributable stunting prevalence (percentage points) and number of stunting cases among 2-year olds attributable to individual risks and risk factor clusters (95% confidence intervals presented in parentheses)**

| **Description** | **PAF** | **Attributable stunting prevalence** | **Attributable stunting cases (thousands)** |
| --- | --- | --- | --- |
| **Maternal nutrition and infection** | **6.5 (5.9, 7.4)** | **0.7 (0.4, 1.0)** | **1.2 (0.7, 1.8)** |
| Maternal short stature | 5.3 (5.1, 5.5) | 0.5 (0.3, 0.8) | 1.0 (0.6, 1.4) |
| Maternal underweight | 0.3 (0.1, 0.5) | 0.0 (0.0, 0.1) | 0.0 (0.0, 0.1) |
| Maternal malaria | 0.3 (0.1, 0.6) | 0.0 (0.0, 0.1) | 0.1 (0.0, 0.1) |
| Maternal anemia | 0.7 (0.2, 1.5) | 0.1 (0.0, 0.2) | 0.1 (0.0, 0.3) |
| **Teenage motherhood and short birth intervals** | **1.8 (1.7, 1.9)** | **0.2 (0.1, 0.3)** | **0.3 (0.2, 0.5)** |
| Teenage motherhood | 0.5 (0.5, 0.6) | 0.1 (0.0, 0.1) | 0.1 (0.1, 0.1) |
| Short birth intervals | 1.2 (1.1, 1.3) | 0.1 (0.1, 0.2) | 0.2 (0.1, 0.3) |
| **Fetal growth restriction and preterm birth** | **17.8 (12.9, 22.1)** | **1.8 (1.0, 2.8)** | **3.4 (1.9, 5.3)** |
| Preterm, small-for-gestational age | 4.0 (1.2, 6.9) | 0.4 (0.1, 0.8) | 0.7 (0.2, 1.5) |
| Preterm, appropriate-for-gestational age | 5.5 (1.8, 9.3) | 0.6 (0.1, 1.1) | 1.1 (0.2, 2.1) |
| Term, small-for-gestational age | 9.4 (5.8, 13.2) | 0.9 (0.5, 1.5) | 1.8 (0.9, 2.9) |
| Low birth weight | 9.4 (7.8, 11.1) | 0.9 (0.5, 1.4) | 1.8 (1.0, 2.7) |
| **Child nutrition and infection** | **13.2 (4.5, 28.2)** | **1.3 (0.4, 2.9)** | **2.5 (0.7, 5.4)** |
| Childhood zinc deficiency | 4.6 (0.7, 28.7) | 0.5 (0.1, 2.6) | 0.9 (0.1, 4.9) |
| Childhood diarrhea | 11.2 (3.5, 21.0) | 1.1 (0.3, 2.3) | 2.1 (0.6, 4.3) |
| Non-exclusive breastfeeding | 1.9 (0.2, 4.5) | 0.2 (0.0, 0.5) | 0.4 (0.0, 0.9) |
| Discontinued breastfeeding | 1.5 (0.2, 3.8) | 0.2 (0.0, 0.4) | 0.3 (0.0, 0.7) |
| HIV infection without HAART before 2 years | No data | No data | No data |
| **Water, sanitation and biomass fuel use** | **4.0 (2.7, 5.3)** | **0.4 (0.2, 0.6)** | **0.8 (0.4, 1.2)** |
| Unimproved sanitation | 3.6 (2.3, 4.8) | 0.4 (0.2, 0.6) | 0.7 (0.3, 1.1) |
| Unimproved water | 0.4 (0.2, 0.7) | 0.0 (0.0, 0.1) | 0.1 (0.0, 0.2) |
| Use of biomass fuels | 0.0 (-0.1, 0.1) | 0.0 (0.0, 0.0) | 0.0 (0.0, 0.0) |

**Fig 1: Stunting cases among 2-year olds attributable to individual risk factors**

Turkey

**Region: North Africa/Middle East; Sub-region: North Africa / Middle East**

**Stunting prevalence among children age 2: 20.3%**

**Stunting cases among children age 2: 265,613**

**Population of children age 2: 1,306,113**

This country profile provides results for 2011 from the analysis presented in *Risk factors for childhood stunting in 137 developing countries: a comparative risk assessment analysis at global, regional, and country levels.*

**Table 1: Population attributable fraction (PAF in %), attributable stunting prevalence (percentage points) and number of stunting cases among 2-year olds attributable to individual risks and risk factor clusters (95% confidence intervals presented in parentheses)**

| **Description** | **PAF** | **Attributable stunting prevalence** | **Attributable stunting cases (thousands)** |
| --- | --- | --- | --- |
| **Maternal nutrition and infection** | **7.0 (6.0, 8.4)** | **1.4 (0.8, 2.1)** | **18.6 (10.3, 28.0)** |
| Maternal short stature | 5.5 (5.4, 5.7) | 1.1 (0.6, 1.6) | 14.7 (8.2, 21.1) |
| Maternal underweight | 0.3 (0.1, 0.5) | 0.1 (0.0, 0.1) | 0.7 (0.2, 1.5) |
| Maternal malaria | 0.0 (0.0, 0.0) | 0.0 (0.0, 0.0) | 0.0 (0.0, 0.0) |
| Maternal anemia | 1.3 (0.3, 2.7) | 0.3 (0.0, 0.6) | 3.4 (0.6, 8.1) |
| **Teenage motherhood and short birth intervals** | **1.7 (1.6, 1.8)** | **0.3 (0.2, 0.5)** | **4.5 (2.5, 6.6)** |
| Teenage motherhood | 0.7 (0.6, 0.7) | 0.1 (0.1, 0.2) | 1.7 (1.0, 2.5) |
| Short birth intervals | 1.0 (1.0, 1.1) | 0.2 (0.1, 0.3) | 2.8 (1.6, 4.0) |
| **Fetal growth restriction and preterm birth** | **28.5 (24.0, 33.1)** | **5.8 (3.2, 8.5)** | **75.6 (42.1, 111)** |
| Preterm, small-for-gestational age | 6.6 (3.5, 10.3) | 1.3 (0.6, 2.4) | 17.6 (7.3, 31.9) |
| Preterm, appropriate-for-gestational age | 6.0 (3.3, 9.2) | 1.2 (0.5, 2.2) | 15.8 (7.0, 28.2) |
| Term, small-for-gestational age | 18.6 (13.6, 24.0) | 3.8 (2.0, 5.8) | 49.2 (25.6, 75.9) |
| Low birth weight | 17.5 (14.6, 20.3) | 3.6 (2.0, 5.2) | 46.4 (25.7, 67.5) |
| **Child nutrition and infection** | **19.3 (8.3, 30.7)** | **3.9 (1.4, 7.3)** | **51.3 (18.3, 95.3)** |
| Childhood zinc deficiency | 3.0 (0.7, 9.5) | 0.6 (0.1, 2.1) | 7.9 (1.6, 27.4) |
| Childhood diarrhea | 18.1 (7.1, 28.8) | 3.7 (1.2, 6.8) | 48.0 (16.3, 88.5) |
| Non-exclusive breastfeeding | 2.1 (-1.0, 5.4) | 0.4 (-0.2, 1.2) | 5.6 (-2.3, 15.7) |
| Discontinued breastfeeding | 2.3 (0.3, 5.4) | 0.5 (0.1, 1.1) | 6.1 (0.7, 14.6) |
| HIV infection without HAART before 2 years | No data | No data | No data |
| **Water, sanitation and biomass fuel use** | **3.8 (2.4, 5.1)** | **0.8 (0.4, 1.3)** | **10.0 (4.6, 16.5)** |
| Unimproved sanitation | 3.2 (1.9, 4.6) | 0.7 (0.3, 1.1) | 8.6 (3.9, 14.3) |
| Unimproved water | 0.3 (0.0, 0.6) | 0.1 (0.0, 0.1) | 0.7 (0.0, 1.7) |
| Use of biomass fuels | 0.3 (-0.1, 0.7) | 0.1 (0.0, 0.2) | 0.7 (-0.3, 2.1) |

**Fig 1: Stunting cases among 2-year olds attributable to individual risk factors**

Turkmenistan

**Region: Central Asia Eastern Europe/Central Asia; Sub-region: Asia, Central**

**Stunting prevalence among children age 2: 23.1%**

**Stunting cases among children age 2: 25,427**

**Population of children age 2: 109,968**

This country profile provides results for 2011 from the analysis presented in *Risk factors for childhood stunting in 137 developing countries: a comparative risk assessment analysis at global, regional, and country levels.*

**Table 1: Population attributable fraction (PAF in %), attributable stunting prevalence (percentage points) and number of stunting cases among 2-year olds attributable to individual risks and risk factor clusters (95% confidence intervals presented in parentheses)**

| **Description** | **PAF** | **Attributable stunting prevalence** | **Attributable stunting cases (thousands)** |
| --- | --- | --- | --- |
| **Maternal nutrition and infection** | **4.4 (3.7, 5.1)** | **1.0 (0.5, 1.5)** | **1.1 (0.5, 1.7)** |
| Maternal short stature | 3.4 (3.2, 3.7) | 0.8 (0.4, 1.2) | 0.9 (0.4, 1.3) |
| Maternal underweight | 0.3 (0.1, 0.5) | 0.1 (0.0, 0.1) | 0.1 (0.0, 0.2) |
| Maternal malaria | 0.0 (0.0, 0.0) | 0.0 (0.0, 0.0) | 0.0 (0.0, 0.0) |
| Maternal anemia | 0.7 (0.1, 1.5) | 0.2 (0.0, 0.4) | 0.2 (0.0, 0.4) |
| **Teenage motherhood and short birth intervals** | **1.6 (1.5, 1.7)** | **0.4 (0.2, 0.5)** | **0.4 (0.2, 0.6)** |
| Teenage motherhood | 0.5 (0.5, 0.6) | 0.1 (0.1, 0.2) | 0.1 (0.1, 0.2) |
| Short birth intervals | 1.0 (1.0, 1.1) | 0.2 (0.1, 0.4) | 0.3 (0.1, 0.4) |
| **Fetal growth restriction and preterm birth** | **22.0 (15.0, 28.2)** | **5.1 (2.2, 8.5)** | **5.6 (2.4, 9.3)** |
| Preterm, small-for-gestational age | 5.9 (1.1, 11.3) | 1.4 (0.2, 3.0) | 1.5 (0.2, 3.3) |
| Preterm, appropriate-for-gestational age | 5.3 (0.8, 9.9) | 1.2 (0.2, 2.8) | 1.4 (0.2, 3.0) |
| Term, small-for-gestational age | 12.4 (7.0, 17.7) | 2.9 (1.2, 5.1) | 3.2 (1.3, 5.6) |
| Low birth weight | 8.9 (7.4, 10.6) | 2.1 (1.0, 3.2) | 2.3 (1.1, 3.5) |
| **Child nutrition and infection** | **18.0 (7.5, 29.1)** | **4.2 (1.4, 7.6)** | **4.6 (1.5, 8.3)** |
| Childhood zinc deficiency | 0.4 (0.1, 1.2) | 0.1 (0.0, 0.3) | 0.1 (0.0, 0.3) |
| Childhood diarrhea | 17.8 (7.2, 28.7) | 4.1 (1.4, 7.5) | 4.5 (1.5, 8.3) |
| Non-exclusive breastfeeding | 3.2 (0.6, 6.6) | 0.7 (0.1, 1.7) | 0.8 (0.1, 1.9) |
| Discontinued breastfeeding | 2.5 (0.4, 5.5) | 0.6 (0.1, 1.4) | 0.6 (0.1, 1.6) |
| HIV infection without HAART before 2 years | No data | No data | No data |
| **Water, sanitation and biomass fuel use** | **3.1 (2.1, 4.2)** | **0.7 (0.3, 1.2)** | **0.8 (0.3, 1.3)** |
| Unimproved sanitation | 1.8 (1.4, 2.2) | 0.4 (0.2, 0.7) | 0.5 (0.2, 0.7) |
| Unimproved water | 1.3 (0.4, 2.4) | 0.3 (0.1, 0.6) | 0.3 (0.1, 0.7) |
| Use of biomass fuels | 0.0 (-0.1, 0.1) | 0.0 (0.0, 0.0) | 0.0 (0.0, 0.0) |

**Fig 1: Stunting cases among 2-year olds attributable to individual risk factors**

Uganda

**Region: Sub-Saharan Africa; Sub-region: Sub-Saharan Africa, East**

**Stunting prevalence among children age 2: 46.5%**

**Stunting cases among children age 2: 695,024**

**Population of children age 2: 1,493,681**

This country profile provides results for 2011 from the analysis presented in *Risk factors for childhood stunting in 137 developing countries: a comparative risk assessment analysis at global, regional, and country levels.*

**Table 1: Population attributable fraction (PAF in %), attributable stunting prevalence (percentage points) and number of stunting cases among 2-year olds attributable to individual risks and risk factor clusters (95% confidence intervals presented in parentheses)**

| **Description** | **PAF** | **Attributable stunting prevalence** | **Attributable stunting cases (thousands)** |
| --- | --- | --- | --- |
| **Maternal nutrition and infection** | **12.1 (9.9, 14.3)** | **5.6 (4.0, 7.5)** | **83.9 (59.7, 112.4)** |
| Maternal short stature | 6.5 (6.3, 6.7) | 3.0 (2.3, 3.8) | 45.1 (34.1, 56.5) |
| Maternal underweight | 1.4 (0.6, 2.4) | 0.7 (0.3, 1.2) | 9.9 (4.2, 17.4) |
| Maternal malaria | 2.7 (1.0, 4.4) | 1.2 (0.5, 2.2) | 18.6 (6.8, 32.1) |
| Maternal anemia | 2.0 (0.6, 3.5) | 0.9 (0.3, 1.7) | 13.9 (3.9, 25.9) |
| **Teenage motherhood and short birth intervals** | **2.2 (2.1, 2.3)** | **1.0 (0.8, 1.3)** | **15.2 (11.4, 19.6)** |
| Teenage motherhood | 1.0 (0.9, 1.1) | 0.5 (0.4, 0.6) | 7.1 (5.3, 9.1) |
| Short birth intervals | 1.2 (1.1, 1.3) | 0.5 (0.4, 0.7) | 8.2 (6.1, 10.6) |
| **Fetal growth restriction and preterm birth** | **30.6 (26.1, 34.8)** | **14.2 (10.4, 18.4)** | **212.7 (155.8, 274.6)** |
| Preterm, small-for-gestational age | 5.2 (1.7, 9.6) | 2.4 (0.8, 4.6) | 36.2 (11.3, 68.6) |
| Preterm, appropriate-for-gestational age | 7.1 (2.2, 11.8) | 3.3 (1.0, 5.9) | 49.0 (14.8, 88.7) |
| Term, small-for-gestational age | 21.2 (17.4, 25.4) | 9.9 (6.9, 13.1) | 147.4 (103.5, 196.2) |
| Low birth weight | 21.2 (17.9, 24.5) | 9.9 (7.0, 13.0) | 147.5 (104, 193.4) |
| **Child nutrition and infection** | **15.0 (5.7, 25.7)** | **7.0 (2.5, 12.5)** | **104.3 (36.8, 186.9)** |
| Childhood zinc deficiency | 0.7 (0.3, 1.2) | 0.3 (0.1, 0.6) | 4.8 (1.8, 8.9) |
| Childhood diarrhea | 14.7 (5.5, 25.3) | 6.8 (2.3, 12.4) | 102.2 (35.0, 185.7) |
| Non-exclusive breastfeeding | 1.4 (-1.2, 4.4) | 0.7 (-0.5, 2.2) | 9.8 (-8.0, 32.8) |
| Discontinued breastfeeding | 1.0 (0.1, 2.6) | 0.5 (0.0, 1.2) | 6.7 (0.6, 18.3) |
| HIV infection without HAART before 2 years | 0.5 (0.3, 0.8) | 0.2 (0.1, 0.4) | 3.3 (1.7, 6.0) |
| **Water, sanitation and biomass fuel use** | **25.2 (22.8, 27.6)** | **11.7 (8.7, 14.8)** | **174.8 (130.6, 221.8)** |
| Unimproved sanitation | 18.6 (16.6, 20.6) | 8.7 (6.5, 11.1) | 129.4 (97.7, 166.2) |
| Unimproved water | 2.2 (1.4, 3.2) | 1.0 (0.6, 1.6) | 15.6 (8.8, 24.1) |
| Use of biomass fuels | 5.9 (4.2, 7.6) | 2.8 (1.7, 3.9) | 41.1 (25.9, 58.2) |

**Fig 1: Stunting cases among 2-year olds attributable to individual risk factors**

United Arab Emirates

**Region: North Africa/Middle East; Sub-region: North Africa / Middle East**

**Stunting prevalence among children age 2: 10.2%**

**Stunting cases among children age 2: 9,090**

**Population of children age 2: 89,235**

This country profile provides results for 2011 from the analysis presented in *Risk factors for childhood stunting in 137 developing countries: a comparative risk assessment analysis at global, regional, and country levels.*

**Table 1: Population attributable fraction (PAF in %), attributable stunting prevalence (percentage points) and number of stunting cases among 2-year olds attributable to individual risks and risk factor clusters (95% confidence intervals presented in parentheses)**

| **Description** | **PAF** | **Attributable stunting prevalence** | **Attributable stunting cases (thousands)** |
| --- | --- | --- | --- |
| **Maternal nutrition and infection** | **9.7 (8.6, 10.5)** | **1.0 (0.1, 1.8)** | **0.9 (0.1, 1.6)** |
| Maternal short stature | 9.2 (8.2, 10.0) | 0.9 (0.1, 1.7) | 0.8 (0.1, 1.5) |
| Maternal underweight | 0.1 (0.0, 0.2) | 0.0 (0.0, 0.0) | 0.0 (0.0, 0.0) |
| Maternal malaria | 0.0 (0.0, 0.0) | 0.0 (0.0, 0.0) | 0.0 (0.0, 0.0) |
| Maternal anemia | 0.4 (0.1, 0.9) | 0.0 (0.0, 0.1) | 0.0 (0.0, 0.1) |
| **Teenage motherhood and short birth intervals** | **1.8 (1.7, 1.9)** | **0.2 (0.0, 0.3)** | **0.2 (0.0, 0.3)** |
| Teenage motherhood | 0.5 (0.5, 0.6) | 0.1 (0.0, 0.1) | 0.0 (0.0, 0.1) |
| Short birth intervals | 1.2 (1.1, 1.3) | 0.1 (0.0, 0.2) | 0.1 (0.0, 0.2) |
| **Fetal growth restriction and preterm birth** | **15.8 (11.3, 20.1)** | **1.6 (0.2, 3.0)** | **1.4 (0.2, 2.7)** |
| Preterm, small-for-gestational age | 5.1 (2.7, 8.1) | 0.5 (0.1, 1.1) | 0.5 (0.1, 1.0) |
| Preterm, appropriate-for-gestational age | 4.5 (2.5, 6.8) | 0.5 (0.1, 0.9) | 0.4 (0.1, 0.8) |
| Term, small-for-gestational age | 7.1 (2.4, 11.7) | 0.7 (0.1, 1.6) | 0.6 (0.1, 1.4) |
| Low birth weight | 5.8 (4.8, 6.9) | 0.6 (0.1, 1.1) | 0.5 (0.1, 1.0) |
| **Child nutrition and infection** | **9.9 (3.5, 25.0)** | **1.0 (0.1, 2.6)** | **0.9 (0.1, 2.3)** |
| Childhood zinc deficiency | 5.8 (0.8, 32.0) | 0.6 (0.0, 3.2) | 0.5 (0.0, 2.8) |
| Childhood diarrhea | 7.2 (2.9, 12.2) | 0.7 (0.1, 1.6) | 0.7 (0.1, 1.4) |
| Non-exclusive breastfeeding | 1.4 (0.3, 3.2) | 0.1 (0.0, 0.4) | 0.1 (0.0, 0.4) |
| HIV infection without HAART before 2 years | No data | No data | No data |
| Discontinued breastfeeding | 1.2 (0.2, 2.7) | 0.1 (0.0, 0.4) | 0.1 (0.0, 0.3) |
| **Water, sanitation and biomass fuel use** | **0.7 (0.5, 0.9)** | **0.1 (0.0, 0.1)** | **0.1 (0.0, 0.1)** |
| Unimproved sanitation | 0.7 (0.5, 0.9) | 0.1 (0.0, 0.1) | 0.1 (0.0, 0.1) |
| Unimproved water | 0.0 (0.0, 0.0) | 0.0 (0.0, 0.0) | 0.0 (0.0, 0.0) |
| Use of biomass fuels | 0.0 (-0.1, 0.1) | 0.0 (0.0, 0.0) | 0.0 (0.0, 0.0) |

**Fig 1: Stunting cases among 2-year olds attributable to individual risk factors**

United Republic of Tanzania

**Region: Sub-Saharan Africa; Sub-region: Sub-Saharan Africa, East**

**Stunting prevalence among children age 2: 54.3%**

**Stunting cases among children age 2: 1,017,812**

**Population of children age 2: 1,873,417**

This country profile provides results for 2011 from the analysis presented in *Risk factors for childhood stunting in 137 developing countries: a comparative risk assessment analysis at global, regional, and country levels.*

**Table 1: Population attributable fraction (PAF in %), attributable stunting prevalence (percentage points) and number of stunting cases among 2-year olds attributable to individual risks and risk factor clusters (95% confidence intervals presented in parentheses)**

| **Description** | **PAF** | **Attributable stunting prevalence** | **Attributable stunting cases (thousands)** |
| --- | --- | --- | --- |
| **Maternal nutrition and infection** | **11.1 (9.8, 12.7)** | **6.1 (4.9, 7.3)** | **113.4 (92.3, 137.1)** |
| Maternal short stature | 7.9 (7.7, 8.0) | 4.3 (3.7, 4.9) | 80.0 (69.0, 91.6) |
| Maternal underweight | 1.1 (0.5, 1.8) | 0.6 (0.3, 1.0) | 10.8 (5.4, 18.3) |
| Maternal malaria | 0.6 (0.2, 1.0) | 0.3 (0.1, 0.5) | 5.7 (2.0, 9.7) |
| Maternal anemia | 2.0 (0.6, 3.4) | 1.1 (0.3, 1.8) | 20.2 (6.2, 34.6) |
| **Teenage motherhood and short birth intervals** | **1.6 (1.5, 1.7)** | **0.9 (0.7, 1.0)** | **16.5 (14.0, 19.2)** |
| Teenage motherhood | 0.9 (0.8, 0.9) | 0.5 (0.4, 0.5) | 8.7 (7.3, 10.2) |
| Short birth intervals | 0.8 (0.7, 0.8) | 0.4 (0.4, 0.5) | 7.9 (6.6, 9.3) |
| **Fetal growth restriction and preterm birth** | **25.3 (20.1, 30.0)** | **13.8 (10.6, 17.2)** | **258 (198.3, 322.6)** |
| Preterm, small-for-gestational age | 4.7 (0.5, 9.0) | 2.5 (0.3, 5.1) | 47.5 (4.9, 95.5) |
| Preterm, appropriate-for-gestational age | 6.3 (1.0, 11.7) | 3.4 (0.5, 6.5) | 64.5 (10.1, 121.4) |
| Term, small-for-gestational age | 16.4 (12.6, 20.3) | 8.9 (6.7, 11.4) | 166.6 (125.7, 212.7) |
| Low birth weight | 15.5 (12.9, 18.1) | 8.4 (6.7, 10.3) | 157.3 (125.3, 193.4) |
| **Child nutrition and infection** | **14.1 (5.4, 24.6)** | **7.7 (2.9, 13.5)** | **143.7 (55.0, 252.7)** |
| Childhood zinc deficiency | 1.1 (0.4, 1.9) | 0.6 (0.2, 1.0) | 11.1 (4.5, 19.5) |
| Childhood diarrhea | 13.6 (4.9, 24.1) | 7.4 (2.7, 13.2) | 138.9 (50.9, 248) |
| Non-exclusive breastfeeding | 1.6 (-1.1, 4.8) | 0.9 (-0.5, 2.6) | 16.3 (-10.0, 48.5) |
| HIV infection without HAART before 2 years | 0.3 (0.2, 0.5) | 0.2 (0.1, 0.3) | 3.1 (1.8, 5.2) |
| Discontinued breastfeeding | 0.7 (0.0, 1.9) | 0.4 (0.0, 1.0) | 7.0 (0.5, 19.4) |
| **Water, sanitation and biomass fuel use** | **30.1 (27.9, 32.3)** | **16.3 (13.9, 19.0)** | **306.3 (259.9, 355.2)** |
| Unimproved sanitation | 24.1 (22.3, 26.1) | 13.1 (11.1, 15.4) | 245.8 (207.7, 288.9) |
| Unimproved water | 3.7 (2.3, 5.2) | 2.0 (1.2, 2.8) | 37.8 (23.3, 53.4) |
| Use of biomass fuels | 4.3 (3.1, 5.5) | 2.3 (1.6, 3.1) | 43.6 (30.4, 58.2) |

**Fig 1: Stunting cases among 2-year olds attributable to individual risk factors**

Uruguay

**Region: High Income Latin America/Caribbean; Sub-region: Latin America, Southern**

**Stunting prevalence among children age 2: 8.7%**

**Stunting cases among children age 2: 4,362**

**Population of children age 2: 49,885**

This country profile provides results for 2011 from the analysis presented in *Risk factors for childhood stunting in 137 developing countries: a comparative risk assessment analysis at global, regional, and country levels.*

**Table 1: Population attributable fraction (PAF in %), attributable stunting prevalence (percentage points) and number of stunting cases among 2-year olds attributable to individual risks and risk factor clusters (95% confidence intervals presented in parentheses)**

| **Description** | **PAF** | **Attributable stunting prevalence** | **Attributable stunting cases (thousands)** |
| --- | --- | --- | --- |
| **Maternal nutrition and infection** | **5.8 (4.8, 7.1)** | **0.5 (0.1, 0.9)** | **0.3 (0.1, 0.5)** |
| Maternal short stature | 4.5 (4.2, 4.7) | 0.4 (0.1, 0.7) | 0.2 (0.0, 0.3) |
| Maternal underweight | 0.2 (0.0, 0.5) | 0.0 (0.0, 0.0) | 0.0 (0.0, 0.0) |
| Maternal malaria | 0.0 (0.0, 0.0) | 0.0 (0.0, 0.0) | 0.0 (0.0, 0.0) |
| Maternal anemia | 1.2 (0.2, 2.5) | 0.1 (0.0, 0.3) | 0.0 (0.0, 0.1) |
| **Teenage motherhood and short birth intervals** | **2.3 (2.2, 2.4)** | **0.2 (0.0, 0.4)** | **0.1 (0.0, 0.2)** |
| Teenage motherhood | 1.4 (1.3, 1.4) | 0.1 (0.0, 0.2) | 0.1 (0.0, 0.1) |
| Short birth intervals | 1.0 (0.9, 1.0) | 0.1 (0.0, 0.2) | 0.0 (0.0, 0.1) |
| **Fetal growth restriction and preterm birth** | **22.4 (18.9, 26.1)** | **2.0 (0.4, 3.5)** | **1.0 (0.2, 1.7)** |
| Preterm, small-for-gestational age | 5.7 (3.9, 7.9) | 0.5 (0.1, 0.9) | 0.2 (0.1, 0.5) |
| Preterm, appropriate-for-gestational age | 5.6 (4.2, 7.0) | 0.5 (0.1, 0.9) | 0.2 (0.1, 0.5) |
| Term, small-for-gestational age | 12.8 (9.0, 17.3) | 1.1 (0.2, 2.1) | 0.6 (0.1, 1.1) |
| Low birth weight | 15.2 (12.7, 17.7) | 1.3 (0.3, 2.4) | 0.7 (0.1, 1.2) |
| **Child nutrition and infection** | **17.6 (7.4, 29.1)** | **1.5 (0.3, 3.3)** | **0.8 (0.2, 1.6)** |
| Childhood zinc deficiency | 2.1 (0.3, 11.5) | 0.2 (0.0, 0.8) | 0.1 (0.0, 0.4) |
| Childhood diarrhea | 16.8 (7.0, 27.1) | 1.5 (0.3, 3.1) | 0.7 (0.1, 1.6) |
| Non-exclusive breastfeeding | 1.5 (-1.8, 4.9) | 0.1 (-0.2, 0.5) | 0.1 (-0.1, 0.3) |
| Discontinued breastfeeding | 2.1 (0.3, 5.0) | 0.2 (0.0, 0.5) | 0.1 (0.0, 0.2) |
| HIV infection without HAART before 2 years | No data | No data | No data |
| **Water, sanitation and biomass fuel use** | **0.4 (0.0, 0.7)** | **0.0 (0.0, 0.1)** | **0.0 (0.0, 0.0)** |
| Unimproved sanitation | 0.4 (0.2, 0.6) | 0.0 (0.0, 0.1) | 0.0 (0.0, 0.0) |
| Unimproved water | 0.0 (0.0, 0.0) | 0.0 (0.0, 0.0) | 0.0 (0.0, 0.0) |
| Use of biomass fuels | 0.0 (-0.3, 0.3) | 0.0 (0.0, 0.0) | 0.0 (0.0, 0.0) |

**Fig 1: Stunting cases among 2-year olds attributable to individual risk factors**

Uzbekistan

**Region: Central Asia Eastern Europe/Central Asia; Sub-region: Asia, Central**

**Stunting prevalence among children age 2: 25.7%**

**Stunting cases among children age 2: 164,305**

**Population of children age 2: 639,487**

This country profile provides results for 2011 from the analysis presented in *Risk factors for childhood stunting in 137 developing countries: a comparative risk assessment analysis at global, regional, and country levels.*

**Table 1: Population attributable fraction (PAF in %), attributable stunting prevalence (percentage points) and number of stunting cases among 2-year olds attributable to individual risks and risk factor clusters (95% confidence intervals presented in parentheses)**

| **Description** | **PAF** | **Attributable stunting prevalence** | **Attributable stunting cases (thousands)** |
| --- | --- | --- | --- |
| **Maternal nutrition and infection** | **6.7 (6.0, 7.5)** | **1.7 (1.2, 2.3)** | **11.0 (7.4, 14.9)** |
| Maternal short stature | 5.6 (5.4, 5.8) | 1.4 (1.0, 1.9) | 9.3 (6.3, 12.4) |
| Maternal underweight | 0.3 (0.0, 0.6) | 0.1 (0.0, 0.2) | 0.5 (0.1, 1.1) |
| Maternal malaria | 0.0 (0.0, 0.0) | 0.0 (0.0, 0.0) | 0.0 (0.0, 0.0) |
| Maternal anemia | 0.8 (0.2, 1.6) | 0.2 (0.0, 0.4) | 1.3 (0.3, 2.8) |
| **Teenage motherhood and short birth intervals** | **1.6 (1.5, 1.7)** | **0.4 (0.3, 0.6)** | **2.6 (1.8, 3.5)** |
| Teenage motherhood | 0.5 (0.4, 0.5) | 0.1 (0.1, 0.2) | 0.8 (0.5, 1.0) |
| Short birth intervals | 1.1 (1.0, 1.3) | 0.3 (0.2, 0.4) | 1.9 (1.3, 2.6) |
| **Fetal growth restriction and preterm birth** | **20.5 (14.9, 25.5)** | **5.2 (3.3, 7.5)** | **33.6 (21.2, 47.8)** |
| Preterm, small-for-gestational age | 5.3 (1.6, 9.4) | 1.3 (0.4, 2.5) | 8.6 (2.4, 15.8) |
| Preterm, appropriate-for-gestational age | 4.9 (1.7, 8.4) | 1.2 (0.4, 2.3) | 8.0 (2.5, 14.6) |
| Term, small-for-gestational age | 11.7 (6.6, 17.0) | 3.0 (1.6, 4.9) | 19.2 (10.2, 31.3) |
| Low birth weight | 8.8 (7.2, 10.4) | 2.3 (1.4, 3.2) | 14.4 (9.2, 20.2) |
| **Child nutrition and infection** | **19.4 (8.4, 30.5)** | **5.0 (1.9, 8.5)** | **31.9 (12.4, 54.5)** |
| Childhood zinc deficiency | 0.7 (0.2, 1.8) | 0.2 (0.0, 0.5) | 1.1 (0.3, 3.1) |
| Childhood diarrhea | 19.1 (8.0, 30.1) | 4.9 (1.9, 8.4) | 31.4 (12.2, 53.6) |
| Non-exclusive breastfeeding | 2.4 (-0.7, 6.0) | 0.6 (-0.2, 1.6) | 3.9 (-1.3, 10.1) |
| HIV infection without HAART before 2 years | No data | No data | No data |
| Discontinued breastfeeding | 1.4 (0.1, 3.6) | 0.3 (0.0, 0.9) | 2.2 (0.2, 5.9) |
| **Water, sanitation and biomass fuel use** | **2.8 (1.2, 4.5)** | **0.7 (0.3, 1.2)** | **4.7 (1.8, 7.9)** |
| Unimproved sanitation | 1.8 (0.2, 3.3) | 0.5 (0.1, 0.9) | 2.9 (0.3, 5.7) |
| Unimproved water | 0.7 (0.2, 1.2) | 0.2 (0.1, 0.3) | 1.1 (0.4, 2.2) |
| Use of biomass fuels | 0.4 (0.1, 0.7) | 0.1 (0.0, 0.2) | 0.6 (0.1, 1.2) |

**Fig 1: Stunting cases among 2-year olds attributable to individual risk factors**

Vanuatu

**Region: East Asia/Pacific; Sub-region: Oceania**

**Stunting prevalence among children age 2: 31.3%**

**Stunting cases among children age 2: 2,037**

**Population of children age 2: 6,516**

This country profile provides results for 2011 from the analysis presented in *Risk factors for childhood stunting in 137 developing countries: a comparative risk assessment analysis at global, regional, and country levels.*

**Table 1: Population attributable fraction (PAF in %), attributable stunting prevalence (percentage points) and number of stunting cases among 2-year olds attributable to individual risks and risk factor clusters (95% confidence intervals presented in parentheses)**

| **Description** | **PAF** | **Attributable stunting prevalence** | **Attributable stunting cases (thousands)** |
| --- | --- | --- | --- |
| **Maternal nutrition and infection** | **7.4 (6.4, 8.7)** | **2.3 (1.6, 3.1)** | **0.2 (0.1, 0.2)** |
| Maternal short stature | 5.9 (5.7, 6.2) | 1.9 (1.4, 2.4) | 0.1 (0.1, 0.2) |
| Maternal underweight | 0.4 (0.1, 0.8) | 0.1 (0.0, 0.3) | 0.0 (0.0, 0.0) |
| Maternal malaria | 0.0 (0.0, 0.0) | 0.0 (0.0, 0.0) | 0.0 (0.0, 0.0) |
| Maternal anemia | 1.2 (0.3, 2.5) | 0.4 (0.1, 0.8) | 0.0 (0.0, 0.1) |
| **Teenage motherhood and short birth intervals** | **1.3 (1.2, 1.4)** | **0.4 (0.3, 0.5)** | **0.0 (0.0, 0.0)** |
| Teenage motherhood | 0.5 (0.4, 0.5) | 0.1 (0.1, 0.2) | 0.0 (0.0, 0.0) |
| Short birth intervals | 0.8 (0.8, 0.9) | 0.3 (0.2, 0.3) | 0.0 (0.0, 0.0) |
| **Fetal growth restriction and preterm birth** | **27.8 (21.3, 33.4)** | **8.7 (5.8, 11.8)** | **0.6 (0.4, 0.8)** |
| Preterm, small-for-gestational age | 7.1 (0.9, 13.6) | 2.2 (0.3, 4.5) | 0.1 (0.0, 0.3) |
| Preterm, appropriate-for-gestational age | 6.1 (0.6, 11.5) | 1.9 (0.2, 3.7) | 0.1 (0.0, 0.2) |
| Term, small-for-gestational age | 17.2 (12.1, 22.6) | 5.4 (3.3, 7.6) | 0.3 (0.2, 0.5) |
| Low birth weight | 16.4 (13.7, 19.1) | 5.1 (3.6, 6.8) | 0.3 (0.2, 0.4) |
| **Child nutrition and infection** | **10.8 (4.4, 18.1)** | **3.4 (1.3, 5.9)** | **0.2 (0.1, 0.4)** |
| Childhood zinc deficiency | 0.6 (0.2, 1.1) | 0.2 (0.1, 0.4) | 0.0 (0.0, 0.0) |
| Childhood diarrhea | 10.6 (4.1, 17.9) | 3.3 (1.2, 5.8) | 0.2 (0.1, 0.4) |
| Non-exclusive breastfeeding | 1.2 (-1.2, 3.5) | 0.4 (-0.4, 1.1) | 0.0 (0.0, 0.1) |
| HIV infection without HAART before 2 years | No data | No data | No data |
| Discontinued breastfeeding | 0.6 (0.0, 1.7) | 0.2 (0.0, 0.6) | 0.0 (0.0, 0.0) |
| **Water, sanitation and biomass fuel use** | **17.0 (13.7, 20.2)** | **5.3 (3.6, 7.1)** | **0.3 (0.2, 0.5)** |
| Unimproved sanitation | 12.5 (9.4, 15.5) | 3.9 (2.5, 5.4) | 0.3 (0.2, 0.4) |
| Unimproved water | 0.9 (0.5, 1.5) | 0.3 (0.1, 0.5) | 0.0 (0.0, 0.0) |
| Use of biomass fuels | 4.2 (2.9, 5.5) | 1.3 (0.8, 1.9) | 0.1 (0.1, 0.1) |

**Fig 1: Stunting cases among 2-year olds attributable to individual risk factors**

Venezuela

**Region: Latin America/Caribbean; Sub-region: Latin America, Central**

**Stunting prevalence among children age 2: 10.1%**

**Stunting cases among children age 2: 60,389**

**Population of children age 2: 598,649**

This country profile provides results for 2011 from the analysis presented in *Risk factors for childhood stunting in 137 developing countries: a comparative risk assessment analysis at global, regional, and country levels.*

**Table 1: Population attributable fraction (PAF in %), attributable stunting prevalence (percentage points) and number of stunting cases among 2-year olds attributable to individual risks and risk factor clusters (95% confidence intervals presented in parentheses)**

| **Description** | **PAF** | **Attributable stunting prevalence** | **Attributable stunting cases (thousands)** |
| --- | --- | --- | --- |
| **Maternal nutrition and infection** | **8.8 (7.9, 9.8)** | **0.9 (0.2, 1.7)** | **5.3 (1.0, 10.0)** |
| Maternal short stature | 7.7 (7.4, 7.9) | 0.8 (0.2, 1.4) | 4.6 (0.9, 8.5) |
| Maternal underweight | 0.2 (0.0, 0.4) | 0.0 (0.0, 0.0) | 0.1 (0.0, 0.3) |
| Maternal malaria | 0.0 (0.0, 0.0) | 0.0 (0.0, 0.0) | 0.0 (0.0, 0.0) |
| Maternal anemia | 1.0 (0.1, 2.2) | 0.1 (0.0, 0.3) | 0.6 (0.0, 1.7) |
| **Teenage motherhood and short birth intervals** | **2.5 (2.4, 2.6)** | **0.2 (0.0, 0.5)** | **1.5 (0.3, 2.7)** |
| Teenage motherhood | 1.5 (1.4, 1.6) | 0.2 (0.0, 0.3) | 0.9 (0.2, 1.6) |
| Short birth intervals | 1.0 (0.9, 1.1) | 0.1 (0.0, 0.2) | 0.6 (0.1, 1.1) |
| **Fetal growth restriction and preterm birth** | **20.9 (14.9, 26.4)** | **2.1 (0.4, 3.9)** | **12.6 (2.3, 23.6)** |
| Preterm, small-for-gestational age | 5.2 (-0.2, 10.8) | 0.5 (0.0, 1.4) | 3.1 (-0.3, 8.2) |
| Preterm, appropriate-for-gestational age | 5.0 (0.1, 10.4) | 0.5 (0.0, 1.3) | 3.0 (-0.1, 8.0) |
| Term, small-for-gestational age | 12.2 (8.4, 16.5) | 1.2 (0.2, 2.5) | 7.3 (1.4, 14.7) |
| Low birth weight | 13.6 (11.3, 16.0) | 1.4 (0.3, 2.6) | 8.2 (1.5, 15.6) |
| **Child nutrition and infection** | **18.4 (8.1, 30.0)** | **1.9 (0.3, 4.0)** | **11.1 (1.8, 24.0)** |
| Childhood zinc deficiency | 3.2 (0.6, 12.8) | 0.3 (0.0, 1.4) | 1.9 (0.1, 8.3) |
| Childhood diarrhea | 17.1 (7.1, 27.4) | 1.7 (0.3, 3.7) | 10.4 (1.7, 22.2) |
| Non-exclusive breastfeeding | 3.4 (0.7, 7.2) | 0.3 (0.0, 0.9) | 2.0 (0.2, 5.3) |
| HIV infection without HAART before 2 years | No data | No data | No data |
| Discontinued breastfeeding | 2.4 (0.4, 5.7) | 0.2 (0.0, 0.7) | 1.4 (0.0, 4.0) |
| **Water, sanitation and biomass fuel use** | **5.3 (3.3, 7.3)** | **0.5 (0.1, 1.1)** | **3.2 (0.6, 6.3)** |
| Unimproved sanitation | 4.9 (2.9, 6.9) | 0.5 (0.1, 1.0) | 3.0 (0.5, 6.0) |
| Unimproved water | 0.4 (0.1, 0.8) | 0.0 (0.0, 0.1) | 0.3 (0.0, 0.6) |
| Use of biomass fuels | 0.0 (-0.1, 0.1) | 0.0 (0.0, 0.0) | 0.0 (-0.1, 0.1) |

**Fig 1: Stunting cases among 2-year olds attributable to individual risk factors**

Viet Nam

**Region: East Asia/Pacific; Sub-region: Asia, Southeast**

**Stunting prevalence among children age 2: 33.5%**

**Stunting cases among children age 2: 514,735**

**Population of children age 2: 1,537,918**

This country profile provides results for 2011 from the analysis presented in *Risk factors for childhood stunting in 137 developing countries: a comparative risk assessment analysis at global, regional, and country levels.*

**Table 1: Population attributable fraction (PAF in %), attributable stunting prevalence (percentage points) and number of stunting cases among 2-year olds attributable to individual risks and risk factor clusters (95% confidence intervals presented in parentheses)**

| **Description** | **PAF** | **Attributable stunting prevalence** | **Attributable stunting cases (thousands)** |
| --- | --- | --- | --- |
| **Maternal nutrition and infection** | **12.3 (11.6, 13.0)** | **4.1 (3.4, 4.9)** | **63.2 (51.8, 74.9)** |
| Maternal short stature | 10.8 (10.6, 11.0) | 3.6 (3.0, 4.3) | 55.8 (46.4, 65.4) |
| Maternal underweight | 1.0 (0.6, 1.5) | 0.3 (0.2, 0.5) | 5.2 (2.8, 8.2) |
| Maternal malaria | 0.0 (0.0, 0.0) | 0.0 (0.0, 0.0) | 0.0 (0.0, 0.0) |
| Maternal anemia | 0.6 (0.1, 1.2) | 0.2 (0.0, 0.4) | 3.1 (0.7, 6.3) |
| **Teenage motherhood and short birth intervals** | **1.3 (1.2, 1.4)** | **0.4 (0.4, 0.5)** | **6.6 (5.5, 7.9)** |
| Teenage motherhood | 0.5 (0.4, 0.5) | 0.2 (0.1, 0.2) | 2.3 (1.9, 2.8) |
| Short birth intervals | 0.8 (0.8, 0.9) | 0.3 (0.2, 0.3) | 4.3 (3.6, 5.2) |
| **Fetal growth restriction and preterm birth** | **21.0 (15.0, 26.6)** | **7.0 (4.8, 9.6)** | **108.1 (73.6, 148.1)** |
| Preterm, small-for-gestational age | 5.7 (1.0, 11.0) | 1.9 (0.3, 3.8) | 29.5 (5.1, 58.1) |
| Preterm, appropriate-for-gestational age | 5.2 (0.7, 9.9) | 1.8 (0.2, 3.5) | 27.1 (3.3, 53.6) |
| Term, small-for-gestational age | 11.5 (6.6, 16.7) | 3.9 (2.2, 5.8) | 59.3 (33.1, 89.5) |
| Low birth weight | 9.3 (7.6, 11.0) | 3.1 (2.4, 4.0) | 47.7 (37.2, 60.8) |
| **Child nutrition and infection** | **10.4 (4.2, 17.4)** | **3.5 (1.4, 5.9)** | **53.7 (21.3, 90.6)** |
| Childhood zinc deficiency | 0.5 (0.2, 0.8) | 0.2 (0.1, 0.3) | 2.4 (0.9, 4.5) |
| Childhood diarrhea | 10.2 (3.9, 17.2) | 3.4 (1.3, 5.8) | 52.6 (20.6, 89.4) |
| Non-exclusive breastfeeding | 1.3 (-0.5, 3.3) | 0.4 (-0.2, 1.1) | 6.6 (-2.6, 17.6) |
| Discontinued breastfeeding | 0.6 (0.0, 1.6) | 0.2 (0.0, 0.5) | 3.0 (0.2, 8.4) |
| HIV infection without HAART before 2 years | No data | No data | No data |
| **Water, sanitation and biomass fuel use** | **10.5 (8.6, 12.4)** | **3.5 (2.7, 4.4)** | **54.0 (40.9, 68.2)** |
| Unimproved sanitation | 8.4 (6.6, 10.2) | 2.8 (2.1, 3.6) | 43.4 (31.8, 56.1) |
| Unimproved water | 0.7 (0.4, 1.1) | 0.2 (0.1, 0.4) | 3.6 (1.8, 5.8) |
| Use of biomass fuels | 1.6 (1.0, 2.2) | 0.5 (0.3, 0.7) | 8.1 (5.1, 11.5) |

**Fig 1: Stunting cases among 2-year olds attributable to individual risk factors**

Yemen

**Region: North Africa/Middle East; Sub-region: North Africa / Middle East**

**Stunting prevalence among children age 2: 62.6%**

**Stunting cases among children age 2: 508,831**

**Population of children age 2: 813,356**

This country profile provides results for 2011 from the analysis presented in *Risk factors for childhood stunting in 137 developing countries: a comparative risk assessment analysis at global, regional, and country levels.*

**Table 1: Population attributable fraction (PAF in %), attributable stunting prevalence (percentage points) and number of stunting cases among 2-year olds attributable to individual risks and risk factor clusters (95% confidence intervals presented in parentheses)**

| **Description** | **PAF** | **Attributable stunting prevalence** | **Attributable stunting cases (thousands)** |
| --- | --- | --- | --- |
| **Maternal nutrition and infection** | **14.0 (11.8, 16.8)** | **8.8 (6.2, 11.5)** | **71.3 (50.6, 93.7)** |
| Maternal short stature | 9.7 (9.5, 10.0) | 6.1 (4.7, 7.6) | 49.4 (38.0, 61.6) |
| Maternal underweight | 2.0 (0.8, 3.4) | 1.2 (0.5, 2.2) | 10.0 (4.2, 17.9) |
| Maternal malaria | 0.0 (0.0, 0.0) | 0.0 (0.0, 0.0) | 0.0 (0.0, 0.0) |
| Maternal anemia | 2.9 (0.7, 5.8) | 1.8 (0.4, 3.7) | 14.7 (3.5, 30.5) |
| **Teenage motherhood and short birth intervals** | **2.9 (2.7, 3.0)** | **1.8 (1.3, 2.2)** | **14.6 (11.0, 18.2)** |
| Teenage motherhood | 0.7 (0.6, 0.7) | 0.4 (0.3, 0.5) | 3.3 (2.5, 4.2) |
| Short birth intervals | 2.2 (2.0, 2.4) | 1.4 (1.0, 1.7) | 11.3 (8.4, 14.2) |
| **Fetal growth restriction and preterm birth** | **39.4 (35.3, 43.3)** | **24.6 (18.3, 31.4)** | **200.4 (148.7, 255.2)** |
| Preterm, small-for-gestational age | 5.9 (0.9, 11.1) | 3.7 (0.5, 7.0) | 30.0 (4.3, 57.1) |
| Preterm, appropriate-for-gestational age | 5.2 (0.2, 10.1) | 3.3 (0.1, 6.4) | 26.6 (1.1, 51.9) |
| Term, small-for-gestational age | 32.0 (27.3, 37.0) | 20.0 (14.7, 25.7) | 162.6 (119.3, 209.2) |
| Low birth weight | 30.7 (26.4, 34.8) | 19.2 (14.3, 24.7) | 155.9 (116.3, 200.5) |
| **Child nutrition and infection** | **13.5 (4.9, 23.9)** | **8.5 (2.9, 15.9)** | **68.9 (24.0, 128.9)** |
| Childhood zinc deficiency | 0.7 (0.3, 1.2) | 0.4 (0.2, 0.8) | 3.4 (1.2, 6.6) |
| Childhood diarrhea | 13.2 (4.6, 23.5) | 8.3 (2.7, 15.7) | 67.4 (22.3, 127.5) |
| Non-exclusive breastfeeding | 2.4 (0.5, 5.5) | 1.5 (0.3, 3.6) | 12.0 (2.5, 29.1) |
[truncated: 6,589 more chars]
