# Supplementary material for: Risk Factors for Childhood Stunting in 137 Developing Countries: A Comparative Risk Assessment Analysis at Global, Regional, and Country Levels
Source: PLoS Med. 2016 Nov 1;13(11):e1002164. doi: 10.1371/journal.pmed.1002164 (PMC5089547; doi:10.1371/journal.pmed.1002164)
Supplement: S6 Text — (DOCX) [file pmed.1002164.s017.docx]

**Objective 2: Quantify the effects of risk factors on early childhood development in national**

**populations**

*Activity 2.1: Estimating the etiological effects of individual risk factors on child development*

We will conduct systematic reviews and analyze primary data from epidemiological studies to obtain the

best current evidence on etiological effects of risk factors on developmental outcomes.

*Activity 2.1a: Systematic reviews and meta-analyses of existing studies*

A strength of our proposed project is the use of systematic reviews and meta-analyses of high-quality

epidemiologic studies to estimate etiological effect sizes. Meta-analytical estimates across all studies

increase the precision of the effect sizes and reduce the potential for bias. We will begin with systematic

reviews in previous publications, e.g. the *Lancet* Child Development Series [8], and either update them or,

when relevant, conduct new meta-analyses that allow parameterizing the impact models with the most

up-to-date etiological effects.

We will use study inclusion, exclusion, and quality criteria that help remove or minimize bias. In particular,

we will use randomized trials of exposure reduction; or observational studies that have (i) adjusted for

major potential confounding factors and (ii) where relevant, adjusted for the bias introduced by exposure

measurement error (the so-called regression dilution bias). For example, evidence from randomized trials

of zinc supplementation will be pooled to estimate the etiological effects of zinc deficiency on HAZ and

stunting, or those from iron supplementation for the effects of low maternal Hb and iron deficiency on

LBW, which will itself be related to stunting by pooling data from prospective cohort studies. We will also

incorporate evidence on the relationship between maternal Hb and birth weight from well-conducted

prospective studies, which are typically larger and allow us to examine the potential effect modification by

contextual factors (see below). We will use the study quality criteria to evaluate whether the effect sizes

vary based on study quality. The systematic review and meta-analyses will lead to new pooled estimates

of etiological effect sizes for each risk factor (**Milestone 2.1.1**). They will also provide an inventory of the

current evidence for various risk factors and will therefore help inform the design of future studies such as

the re-enrolment studies funded through the first Saving Brains RFA.

We will systematically evaluate whether the effects of risk factors on child development depend on

contextual variables, e.g. the effects of maternal Hb on birth weight and subsequent child development

may depend on malaria endemicity. Effect modification will be examined using meta-regression analyses.

We will use this knowledge for applying appropriate etiological effect sizes in our models, in Activity 2.2.

A particular focus of our meta-analyses of existing studies will be to assess and enhance comparability in

exposure and outcome metrics. For example, randomized trials of micronutrient supplementation have

used different formulations and doses [9]. Therefore, pooling the evidence across these trials requires

converting all effect estimates to a comparable dose. Issues of comparability will also inevitably arise

when epidemiological studies use different definitions and measures of child developmental. For

example, in the same context of micronutrient supplementation, studies report effects on the Bayley Scale

of development, on the Wechsler Scale of intelligence, receptive and expressive vocabulary, visual

evoked potentials, among others [9]. We have substantial experience in our work on nutritional and

metabolic risk factors in using data sources that have used multiple definitions of an exposure or outcome

to create “cross-walking” or conversion relationships among them [6,97,107]. In the proposed research,

we will use the studies with individual record data available to the consortium members, as well as the

relevant Saving Brains re-enrollment studies, that have used more than one metric of exposure and/or

outcome to convert the effect sizes to a common metric. This focus on the choice and comparability of

metrics of risk factor exposure and child development will also inform the design of subsequent studies in

a way that comparability can be enhanced.

*Activity 2.1b: Analysis of primary data from epidemiological studies*

To better estimate etiological effects of individual and multiple risk factors, we will supplement the metaanalyses of published epidemiological studies with analysis of primary records available to us, hence

generating new estimates of the etiological effects of risk factors on child development (**Milestone 2.1.2**).

We have access to data from several large prospective studies of maternal and childhood risk factors and

developmental outcomes from the work of the investigators of the consortium members. For example, we

have access to data from 12 randomized trials among approximately 50,000 mother-child pairs

implemented over the past 20 years or underway, as part of our collaboration in Tanzania led by Drs.

Fawzi, Masanja, and partners at multiple institutions in the country. The trials have largely examined the

efficacy of nutritional interventions (including vitamin A, zinc, iron, or multiple micronutrients) in the first

1,000 days among pregnant mothers or children on various outcomes that are relevant to this research

including pregnancy outcomes (LBW and prematurity), childhood infections (HIV, TB, malaria, diarrhea

and ALRI), and importantly on stunting. Three trials also included measures of child motor and cognitive

development. Data from these trials provide a rich set of variables as risk factors and covariates, including

for example, maternal education, antenatal and postnatal depression, and nutritional status, that can be

used for analysis. We also have access to individual-record epidemiological studies from our partner

projects. For example, our partner study CHERG which involves Professors Bhutta, Ezzati, and Fawzi,

has collated data from 18 prospective studies in developing countries (six in Africa, eight in Asia, and four in Latin America) that allow examining the effects of birth weight (including prematurity and IUGR) on

stunting at different ages. Other cohorts in CHERG will allow analyzing the effect of maternal stature on

prematurity and IUGR, which can then be linked to stunting based on the above cohorts.

We will also rely on effect sizes from the ongoing Saving Brains reenrollment studies – for example the

follow-up study of 3,700 children in Pakistan who received micronutrient supplementation in the first 1000

days of life and are currently 5-12 years old; as well as the re-enrollment of the children in the Pakistan

Early Child Development Scale-up trial that aims to evaluate the effect of cognitive stimulation and

responsive parenting on a large number of developmental indicators. Finally, we will use epidemiological

studies with publicly available data such as the Cebu Longitudinal Health and Nutrition Survey in the

Philippines that includes detailed data on feeding patterns as well as various social, economic and

environmental factors for mothers and children in 28,000 households in 17 urban and 16 rural

communities; and the Young Lives study, a large prospective study which enrolled 8,000 1-year olds in

2002, and has completed two rounds of follow-ups, one at age 5 (pre-school) and one at age 8 in four

countries (Ethiopia, India, Peru and Vietnam) and includes high-quality information on factors related to

childhood poverty and schooling outcomes.

Our consortium members also have the opportunity to include colleagues at Harvard and ICL who are

leading a set of prospective studies of children and birth cohorts in high-income countries with long-term

follow-ups and including data on various maternal, pregnancy and child measures of nutrition, pregnancy

outcomes, child growth and development. We acknowledge that the epidemiological, environmental and

health system characteristics is different in these studies compared with the low-income countries but we

believe that the very long term follow-up and the extensive data on various childhood and adolescent

characteristics is valuable in the analysis of the effects of various risk factors on schooling and economic

outcomes. These studies include the Northern Finland Birth Cohorts (12,231 people born in 1966 and

9,479 people born in 1985-86); The Danish National Birth Cohort (101,042 pregnant women enrolled in

1996-2002, leading to 96,986 children currently under follow up); and Project Viva including a birth cohort

of 1300 mother-infant pairs enrolled in 1998-2002 in Boston. The large sample sizes and long follow-up of

these prospective studies will particularly make them suitable for multivariate adjustment in analysis of

single risk factors as well as some of the joint risk analyses explained below. The PIs of these studies

have agreed to analysis of the data for this purpose (Professor Sjurdur Olsen of the Danish Epidemiology

Science Centre, and an Adjunct Professor of Nutrition at HSPH, is the PI of the Danish National Birth

Cohort; Professor Matthew Gillman is the Director of the Obesity Prevention Program in the Department

of Population Medicine at HMS and PI of Project Viva).

*Activity 2.2: Estimating the etiological effects of multiple risk factors on child development*

We will examine the joint effect of multiple risk factors on early childhood development both in the context

of previously published studies collated in Activity 2.1 and through re-analysis of primary data from

prospective studies that are available to us through various consortium members described above

(**Milestone 2.2.1**). Relying on primary data for joint etiological effects of risk factors removes the need for

strong assumptions regarding how multiple risk factors interact. As in Activity 1.3, risk factor combinations

will be based on consultation with the End-User and Stakeholder and Advisory Groups while also

considering the availability of interventions delivery platforms that cover more than one risk factor.

A specific form of the joint etiological effects of multiple risk factors is when the effects of one risk (e.g.

zinc deficiency) are partially mediated through another risk factor (e.g. diarrhea). In these cases, the joint

etiological effect of the distal and proximal risk factors together is a combination of the effect of the more

proximal risk (diarrhea) as well as the non-mediated part of the distal risk factor (the effect of zinc

deficiency growth, and subsequently schooling through other pathways). In such cases, we will use the

primary data available to the consortium with analytical methods for direct and mediated effects [105] to

estimate the non-mediated portion.

*Activity 2.3a: Estimating the impact of individual risk factors*

To estimate effects of individual risk factors, we will use one or both of the analytical approaches below,

guided by the empirical evidence from based from Activity 2.1:

Analytical approach 1: When risk factors increase the risk of impaired development (e.g. as measured by

stunting) in a proportional (or multiplicative) manner, the overall absolute effect depends, in a

multiplicative way, on the joint effect of multiple risk factors. For example, childhood infections may

increase the risk of subsequent stunting in a proportional manner [64,81,84,86 ], and hence have a larger

effect in low-birth-weight children than those with adequate fetal growth. In this case, the absolute effects

of a risk factor depend not only on its own exposure in the population, but also on the total (background)

level of stunting. If the epidemiological evidence indicates that risk factor effects are multiplicative, we will

implement an analytic model based on a proportional risk principle, accounting for dependency of

absolute effects on background rates (**Milestones 2.3.1**); we have used this modeling approach in

numerous previous national and global CRA analyses [49,95]. Our current work on comparable estimates

of stunting prevalence by country (Figure 2) will provide information on background prevalence.

Analytical approach 2: Risk factors may affect child development through an absolute effect on outcomes

such as HAZ. For example, a recent meta-analysis of over 30 studies indicated that zinc supplementation

improved HAZ by 0.19 [106]. In this case, the proportion of children who are mildly, moderately, or

severely stunted will depend on the presence of other risks that have also shifted the HAZ distribution. In

the presence of evidence for distributional shifts, we will use the absolute effect size in each country to

analyze the effect of risk factors on the population distribution of HAZ (**Milestones 2.3.1**), i.e. what the

distributions would have been like in the absence of the risk factor. Our current work on consistent and

comparable estimates of the full distribution of HAZ by country is a crucial input for this approach.

We will implement the calculations in a microsimulation platform (**Milestone 2.3.2**), described below, and

generate estimates of the impacts of current risk factor levels by country for each individual risk factor

(**Milestone 2.3.3**). Both of the above approaches avoid “double counting” risk factor effects, as they

analyze one risk factor at a time, controlling for factors upstream in the causal chain while allowing for

downstream factors to change as expected following the change in the risk factor of interest – e.g. the

total effect of zinc deficiency may be a combination of its effects through infections and those through

other pathways that affect growth and development. At the same time, the effects of individual risk factors

cannot be simply added to estimate the role of their combinations. Rather, the latter requires to explicitly

account for the joint distribution of risk factor exposures and their joint etiological effect, as below.

*Activity 2.3b: Estimating the impact of risk factor combinations*

A strength of our proposed research is the systematic analysis of the impacts of multiple risk factors in an

epidemiologically consistent framework (**Milestone 2.3.4**). The combined impacts of multiple risks,

compared to how they affect child development individually is not additive but is determined by (i) whether

their exposures in the population are independent or correlated and (ii) whether they interact multiplicatively or in some other form. Activities 1.3 and 2.2 will provide information on the joint

distributions of multiple risks and on their joint etiological effects.

To calculate the joint effects of a set of risk factors, we will repeatedly draw from their joint distributions –

each draw in this microsimulation approach can be considered “a child” with its own vector of exposures

to the risk factor combination. Each draw will be assigned an etiological effect size which will depend on

the levels of all risk factors. The etiological effect size will be a combination of those of individual risks,

with the functional form relating the two being based on the reviews and analyses in Activity 2.2. As

above, possible functional forms include multiplicative effects, additive shifts in HAZ, or other functions as

determined by the epidemiological evidence. We have substantial experience in implementing simulation based analysis of risk factor effects, e.g. in the US CRA [91] and NIMS, and will adapt the methods to suit the purposes of the current study.

We will incorporate these simulations along with other estimation parameters and methods of our

analyses in a web-based tool that will use the inputs to generate outcomes for a wide range of potential

interventions provided by the End-Users and Stakeholders. We will co-ordinate the development of the

new web-tool with our consortium members who are participating in the effort on Lives-Saved Tool (LiST)

which currently only provides estimates for child mortality.

*Activity 2.3c: Selecting policy-relevant exposure scenarios for risk and impact assessment*

The impacts of risk factors are (implicitly) measured by comparing child development status under current

population distribution of risk factor exposure with an alternative distribution [49]. To be informative for

both policies and programs, we will use a series of alternative exposure scenarios for national

populations. First, we will select a set of optimal distributions of risk factors which are theoretically feasible

and lead to the largest benefits for child development (e.g. the well-nourished population, a population

without malaria as has been achieved in other historically malaria-endemic regions, etc.) (**Milestone**

**2.3.5**). This optimal distribution will have the advantage of measuring the impact of all non-optimal levels

of risk factors, consistently and comparably across different risks. In other words, using the optimal

distribution measures the total magnitude of developmental loss associated with risk factor exposure.

Consistent with previous work, the optimal distributions for continuous risk factors will be based on the

levels to which epidemiologic studies have shown continued benefits, e.g. mean maternal Hb of 12 g/dL.

Other beneficial alternative distributions will lie between current exposure and the optimal distribution and

will be selected by users based on policies and interventions. For example, we will also use partial shifts

towards the optimal distribution, as evidenced by experiences of 10% or 20% best-performing countries

which demonstrate feasibility under specific set of interventions. The difference between the results of this

scenarios with that of the optimal scenario will demonstrate the “intervention gap” and can guide research

and development efforts to design and evaluate new interventions. For analysis of multiple risk factors,

we will select joint exposure scenarios for several risk factors that share intervention platforms, such as

multi-nutrient supplements for pregnant mothers and conditional cash transfer interventions that aim to

improve multiple risk factors simultaneously.
